# Supplementary material for: Public opinion on sharing data from health services for clinical and research purposes without explicit consent: an anonymous online survey in the UK
Source: BMJ Open. 2022 Apr 25;12(4):e057579. doi: 10.1136/bmjopen-2021-057579 (PMC9058801; doi:10.1136/bmjopen-2021-057579)
Supplement: Author's manuscript [file bmjopen-2021-057579.draft_revisions.pdf]

BMJ Open is committed to open peer review. As part of this commitment we make the peer review history of every article we publish publicly available.

When an article is published we post the peer reviewers' comments and the authors' responses online. We also post the versions of the paper that were used during peer review. These are the versions that the peer review comments apply to.

The versions of the paper that follow are the versions that were submitted during the peer review process. They are not the versions of record or the final published versions. They should not be cited or distributed as the published version of this manuscript.

BMJ Open is an open access journal and the full, final, typeset and author-corrected version of record of the manuscript is available on our site with no access controls, subscription charges or pay-per-view fees (http://bmjopen.bmj.com).

If you have any questions on BMJ Open's open peer review process please email info.bmjopen@bmj.com

BMJ Open

Public opinion on sharing data from UK health services for clinical and research purposes without explicit consent

Journal:	*BMJ Open*
Manuscript ID	bmjopen-2021-057579
Article Type:	Original research
Date Submitted by the Author:	23-Sep-2021
Complete List of Authors:	Jones, Linda; University of Cambridge, Department of Psychiatry<br>Nelder, Jenny; University of Cambridge, Department of Psychiatry<br>Fryer, Joseph; University of Cambridge, Department of Psychiatry<br>Alsop, Philip<br>Geary, Michael<br>Prince, Mark<br>Cardinal, Rudolf; University of Cambridge, Department of Psychiatry; Cambridgeshire and Peterborough NHS Foundation Trust, Liaison Psychiatry Service
Keywords:	Information management < BIOTECHNOLOGY & BIOINFORMATICS, Health informatics < BIOTECHNOLOGY & BIOINFORMATICS, Health policy < HEALTH SERVICES ADMINISTRATION & MANAGEMENT, MENTAL HEALTH

SCHOLARONE™
Manuscripts

I, the Submitting Author has the right to grant and does grant on behalf of all authors of the Work (as defined in the below author licence), an exclusive licence and/or a non-exclusive licence for contributions from authors who are: i) UK Crown employees; ii) where BMJ has agreed a CC-BY licence shall apply, and/or iii) in accordance with the terms applicable for US Federal Government officers or employees acting as part of their official duties; on a worldwide, perpetual, irrevocable, royalty-free basis to BMJ Publishing Group Ltd ("BMJ") its licensees and where the relevant Journal is co-owned by BMJ to the co-owners of the Journal, to publish the Work in this journal and any other BMJ products and to exploit all rights, as set out in our licence.

The Submitting Author accepts and understands that any supply made under these terms is made by BMJ to the Submitting Author unless you are acting as an employee on behalf of your employer or a postgraduate student of an affiliated institution which is paying any applicable article publishing charge ("APC") for Open Access articles. Where the Submitting Author wishes to make the Work available on an Open Access basis (and intends to pay the relevant APC), the terms of reuse of such Open Access shall be governed by a Creative Commons licence – details of these licences and which Creative Commons licence will apply to this Work are set out in our licence referred to above.

Other than as permitted in any relevant BMJ Author's Self Archiving Policies, I confirm this Work has not been accepted for publication elsewhere, is not being considered for publication elsewhere and does not duplicate material already published. I confirm all authors consent to publication of this Work and authorise the granting of this licence.

For peer review only

Public opinion on sharing data from UK health services for clinical and research purposes without explicit consent

- Linda A. Jones [1], *patient and public involvement lead*. ORCID 0000-0001-9347-5715.
- Jenny R. Nelder [1], *project manager*.
- Joseph M. Fryer [1], *data architect*.
- Philip H. Alsop [2], *research advisory group member*.
- Michael R. Geary [2], *research advisory group member*.
- Mark Prince [2], *research advisory group member*.
- Rudolf N. Cardinal [1,3,*] (rnc1001@cam.ac.uk), *university lecturer in clinical informatics and honorary consultant liaison psychiatrist*. ORCID 0000-0002-8751-5167.

[1] Department of Psychiatry, University of Cambridge, Clifford Albutt Building, Bay 13, Cambridge Biomedical Campus, Cambridge CB2 0AH, UK.

[2] Unaffiliated.

[3] Cambridgeshire and Peterborough NHS Foundation Trust, Liaison Psychiatry Service, Box 190, Addenbrooke's Hospital, Cambridge CB2 0QQ, UK.

[*] Corresponding author.

Version of: **21 Sep 2021**.

Abstract word count: **300**.

Text word count: **4566**.

For peer review only - http://bmjopen.bmj.com/site/about/guidelines.xhtml

1. ABSTRACT

OBJECTIVES. UK National Health Service (NHS/HSC) data is variably shared between healthcare organizations for direct care, and increasingly de-identified for research. Few large-scale studies have examined public opinion on sharing, including of mental health (MH) versus physical health (PH) data. We measured data sharing preferences.

DESIGN/SETTING/INTERVENTIONS/OUTCOMES. Pre-registered anonymous online survey, recruiting Feb–Sep 2020. Participants were randomized to one of three framing statements regarding MH versus PH data. Outcomes were expressed preferences.

PARTICIPANTS. Open to all UK residents. Participants numbered 29275; 40% had experienced a MH condition.

RESULTS. A majority supported identifiable data sharing for direct clinical care without explicit consent, but 20% opposed this. Preference for clinical/identifiable sharing decreased with distance and was slightly less for MH than PH data, with small framing effects. Preference for research/de-identified data sharing without explicit consent showed the same small PH/MH and framing effects, plus greater preference for sharing structured data than de-identified free text. There was net support for research sharing to the NHS, academic institutions, and national research charities, net ambivalence about sharing to profit-making companies researching treatments, and net opposition to sharing to other companies (similar to sharing publicly). De-identified linkage to non-health data was generally supported, except to data held by private companies. We report demographic influences on preference. A clear majority supported a single NHS mechanism to choose uses of their data. Support for data sharing increased during COVID-19.

CONCLUSIONS. Support for healthcare data sharing for direct care without explicit consent is broad but not universal. There is net support for the sharing of de-identified data for research to the NHS, academia, and the charitable sector, but not the commercial sector. A single national NHS-hosted system for patients to control the use of their NHS data for clinical purposes and for research would have broad support.

REGISTRATION. ISRCTN37444142.

2. STRENGTHS AND LIMITATIONS OF THIS STUDY

- Patient and public involvement in study design.
- Detailed questions measuring public opinion on health data sharing for clinical and research purposes.
- Large national sample giving high power, with quantitative analysis, sensitivity analyses to approximate known population demographics, and serendipitous examination of pandemic effects.
- Embedded randomized experiment to control and measure variation due to framing.
- The sample remained under-representative of some demographic groups despite weighting, with potential for unmeasured selection (including self-selection) bias reducing generalisability.

For peer review only - http://bmjopen.bmj.com/site/about/guidelines.xhtml

3. INTRODUCTION

In the United Kingdom (UK), health-related information is recorded routinely by health care professionals and patients within the National Health Service (NHS; England, Scotland, Wales) or Health and Social Care (HSC; Northern Ireland), henceforth "NHS" for brevity. When combined with personal identifiers such as names and addresses, the data represent "confidential patient information" (CPI),[1] used to provide care and managed according to standard principles.[2–7] It is "owned" legally and managed by the NHS organisation recording it.[5,8] De-identified or anonymised forms of the data may be used for research (**Figure 1**) without explicit consent,[5,6] as pledged by the NHS.[9] Identifiable data may be used for research with consent, or—under restricted circumstances—without.[1,5,6] "Fully" anonymised data are not subject to UK data protection legislation.[5,6] However, even supposedly anonymised data relating to individual people carries some risk of re-identification via "jigsaw" attacks.[10]

Our understanding of public wishes about data sharing is incomplete.[11] Information is sometimes not shared clinically when it should be,[3,12] and patients may be surprised and frustrated by failures to share in a "national" health service.[13,14] Previous work, whilst establishing themes in public views on data linkage and sharing for research,[11,15–21] has highlighted the very small scale of many studies, and the paucity of research about the views of minority groups and the acceptability of sharing some types of data, such as mental health (MH) data. Mental illnesses can carry significant stigma[22] but are associated with substantial loss of life expectancy,[23] necessitating improvements in research and care. Some research requires multi-source data, but linkage is complex and may involve transient use of identifiable information.[24] It is unclear to what extent the public supports such work, and whether support varies with the type of data to which health data is linked (e.g. education versus criminal justice); there is little prior research in this area.[18] Proposed national systems for NHS data research such as "care.data" have previously aroused public ire,[25] as have information governance (IG) breaches,[26] and there is current debate about the newest NHS data sharing proposal, General Practice Data for Planning and Research (GPDPR).[8,27]

What do patients and the public want now? We studied views on the sharing of identifiable health data (for clinical purposes) and de-identified health data (for research) within the UK. We examined data destinations ranging from local NHS services to public distribution. We distinguished MH and physical health (PH) data, and for research also structured versus free-text (narrative) data. We asked about data linkage for research. We superimposed a randomized experiment to quantify how opinions on sharing were affected by the "framing"[28] of risk versus benefit. We examined the effect of the COVID-19 pandemic upon preferences. We sought views on potential systems to govern NHS data sharing for clinical and research purposes and to offer direct participation in research.

4. METHODS

4.1. Patient and public involvement

The research team advertised and formed a research advisory group (RAG) comprising patients and carers, who designed the study with the research team. Patients, carers, and other members of the public participated in the study.

4.2. Approvals

We obtained NHS ethics approvals (East of Scotland Research Ethics Service, reference 19/ES/0144) and pre-registered the study (https://doi.org/10.1186/ISRCTN37444142).

For peer review only - http://bmjopen.bmj.com/site/about/guidelines.xhtml

4.3. Inclusion and exclusion criteria; sample size

The inclusion criteria were current residence in the UK and informed consent. The ability to take an online survey (alone or supported) was implicit. Participants under 16 required the permission of their parent or guardian to participate and were asked to report whether they had assistance. We sought a power of 0·9 to detect a "small" effect (Cohen's d=0·1) for the framing intervention (described below), with an estimated minimum n=433/group, but beyond that sought a large representative sample of the UK population.

4.4. Recruitment

Approvals covered public announcements and recruitment via health service sites, in person or through a variety of media. The study was adopted onto the National Institute for Health Research (NIHR) Clinical Research Network (CRN) portfolio; 216 general practice (GP) surgeries and 154 large healthcare organizations (e.g. acute care Trusts, MH Trusts, community hospitals, ambulance Trusts) supported recruitment. The study ran from 2020-02-07 to 2020-09-30.

4.5. Survey

Data were collected using REDCap.[29] The survey is reproduced in the **Supplementary Methods**. It asked for the respondent's views on current and desirable practice for sharing identifiable data for clinical care purposes; personal experience of MH/PH conditions and care; preference for sharing identifiable PH/MH data (for clinical care purposes) to a range of NHS "destinations"; preference for sharing de-identified structured PH/MH data (for research) to a range of potential research "destinations"; similarly for data including de-identified free-text notes; views about potential systems for managing data consent in the NHS; views about linkage for research to non-NHS data sources; and demographics.

4.6. Randomized framing intervention

We hypothesized that the context of questioning would affect willingness to share MH versus PH data, and sought to control and measure this effect. Before we asked about willingness to share different kinds of health data, we presented one of three framing statements: neutral, "concern" (about MH data being more sensitive), or "holistic" (about the importance of joined-up PH/MH care) (**Supplementary Methods**). Participants were randomized to one of the three statements.

4.7. Data processing

Where participants agreed to leave a postcode, this was converted to a larger Office for National Statistics (ONS) geographical area, to prevent inadvertent identification. The geographical area was linked to its known population and Index of Multiple Deprivation (IMD). If the participant provided sufficient information, the ONS National Statistics Socio-Economic Classification (NS-SEC) was also calculated. (See **Supplementary Methods**.)

4.8. Pandemic

By chance, our study spanned the UK onset of the COVID-19 pandemic. This had many consequences, including "lockdowns". Major changes were made to NHS data handling, including instructions to share CPI for public health purposes relating to the pandemic,[30] media reports of sharing of patient-level de-identified data with industry,[31] and guidance for GPs to include additional information in patients' Summary Care Record (SCR, England) unless they had previously opted out.[32] We examined whether the

For peer review only

pandemic was associated with changes in preference relating to data sharing, using 2020-03-23 (first UK "lockdown") as the split point (factor "pandemic": levels "before lockdown", "at/after lockdown").

4.9. Analysis

We analysed using R v3.6.3.[33] We analysed categorical associations via χ^2 tests, and effects upon ordinal Likert-type scales (phrased linguistically to approximate interval scales) via analysis of variance (ANOVA). With a large sample size, the central limit theorem means that the distribution of means and mean differences tends to normal even though the parent population is non-normal, and ANOVA is robust to non-normality,[34–36] permitting ANOVA of discrete dependent variables. Scales measuring likelihood were quantified as −2 very unlikely, −1 unlikely, 0 not sure, +1 likely, +2 very likely. Yes/no scales were quantified as −1 no, 0 not sure, +1 yes. Models involving within-subjects terms were analysed using the *lmer* and *lmerTest* packages, using type III sums of squares, and are expressed thus (~ "is predicted by"; A×B interaction; A*B denotes the inclusion of main effects A and B and their interactions). Statistics are shown to 3 significant figures and degrees of freedom are rounded to integers. We set α=0·05, and report "NS" for "not significant" and "VLP" for a very low *p* value, $p<2{\cdot}2\times10^{-16}$.

Opinions on sharing clinical/identifiable data were analysed using a model termed C1: *sharing~destination*nature*framing*pandemic+(1|subject)*. "Destination" had four levels (local, regional, national, UK-wide), "nature" had two (PH, MH), and framing had three (neutral, MH concern, holistic). We followed up nature×framing interactions by analysing MH and PH data separately using the simplified model C1B: *sharing~destination*framing+(1|subject)*.

To examine the effects of demographic factors and experience, we used a larger model, C2: *sharing~destination*nature*framing*pandemic+age+gender+ethnicity+education+sexuality+religion+nation+imd_quartile+nssec+mh_experience*nature+(1|subject)*. This was only possible for people who provided all necessary demographic information. Levels for demographic factors were as per **Supplementary Table 1**, plus sexuality (two levels: heterosexual/straight, LGBT+ [including homosexual/gay/lesbian, bisexual, other/self-described]) and NS-SEC (five levels).

Opinions on sharing de-identified data for research were analysed using model R1: *sharing~destination*nature*detail*framing*pandemic+(1|subject)*. "Destination" had six levels (NHS, academia, charities, companies conducting treatment research, other companies, publicly); "detail" had two levels (structured only, free text); other factors were as before. To examine nature×framing interactions, we used the simplified model R1B: *sharing~destination*framing+(1|subject)*. For demographic analysis we used model R2: *sharing~destination*nature*detail*framing*pandemic+age+gender+ethnicity+education+sexuality+religion+nation+imd_quartile+nssec+mh_experience*nature+(1|subject)*.

Sensitivity analyses were conducted by weighting to UK population demographic proportions. Effect size plots were created for key models. (See **Supplementary Methods**.)

Willingness for linkage to non-NHS data for research (data source, eight levels) was analysed for all participants using model L1: *willingness~source*pandemic+(1|subject)*. For demographic analysis we used model L2: *willingness~source*pandemic+age+gender+ethnicity+education+sexuality+religion+nation+imd_quartile+nssec+mh_experience+(1|subject)*.

A thematic analysis was performed on free-text comments (see **Supplementary Methods**).

For peer review only - http://bmjopen.bmj.com/site/about/guidelines.xhtml

5. RESULTS

5.1. Participants

Consenting participants numbered 29275. Recruitment is shown in **Supplementary Figure 1A–B**; 8019 participated before UK "lockdown" and 21256 on/after that date. Not everyone completed the survey: participation by stage is shown in **Supplementary Figure 1C**. Median completion time was 18·4 minutes. Participants were evenly distributed across framing conditions (neutral 9812, MH concern 9744, holistic 9719; χ^2_2=0.475, NS).

Demographics are shown in **Supplementary Figure 2A–J** (with free-text responses in **Supplementary Results**). Relative to the UK population (**Supplementary Table 1**), our sample under-represented the youngest and oldest age ranges, males, those of non-white ethnicity, those with less formal education, those professing a religion, residents of UK nations other than England, and people living in more deprived areas. Weighting yielded substantial though incomplete improvement. There was coverage of most UK local authority areas (**Supplementary Figure 2I**).

Forty percent of participants had experienced a MH condition (**Supplementary Figure 3**), primarily depression and anxiety disorders. Of them, 85% had used MH services, primarily their GP and NHS psychological therapy services. Eighty-eight percent of respondents had used PH services, primarily GP and outpatient services.

5.2. Sequence

We first report preferences for clinical data sharing based on multiple-choice questions (**Figure 2**), before examining in detail participants' likelihood of sharing data for clinical and research purposes (**Figure 3** and weighted equivalent in **Supplementary Figure 4**), preferences for data linkage (**Figure 4, Supplementary Figure 5**), and changes associated with the pandemic (**Figure 5**). We then report effect sizes for the analytical models, including for demographic factors (**Figure 6A–C, Supplementary Figure 6A–C**), and finally report views on a possible national consent system (**Figure 7A –F, Supplementary Figure 7A –F**).

5.3. Sharing identifiable data for clinical purposes

Understanding of current NHS practice regarding identifiable data sharing between care providers, without asking the patient each time, are shown in **Figure 2A**. In practice, sharing varies by area, e.g. depending on whether a local/regional shared care record is operative in part of England,[14] or according to limited national systems such as the Intra-NHS Scotland Information Sharing Accord[37] and Scottish Emergency Care Summary,[38] the Northern Ireland Electronic Care Record,[39] the English SCR,[40] and a variety of systems in Wales.[41] To our knowledge, there is no UK-wide sharing, but 30% of respondents thought that there was free sharing of identifiable data across the UK.

When asked preferences via a single multiple-choice question (**Figure 2B**), there was majority (55%) support for sharing identifiable data for direct care across the UK, without being asked first, and 76% support for sharing at least locally, but a substantial minority (20%) said that sharing should not occur without the patient being asked first.

5.4. Sharing mental and physical health data for clinical purposes and for research

Willingness to share health data without being asked every time is shown in **Figure 3** by purpose, type, and destination (with corresponding weighted data in **Supplementary Figure 4**).

For peer review only - http://bmjopen.bmj.com/site/about/guidelines.xhtml

For clinical purposes (with identifiable data), there was strong net willingness to share (**Figure 3A–C**). The most important determinant was destination, with stronger support the more local the sharing. People were slightly more willing to share PH than MH data. There were significant but very small effects of the framing statement, primarily that "MH concern" framing reduced willingness to share MH data. In the whole-sample analysis (model C1), there were highly significant effects of destination and nature, as well as interactions including nature×framing (**Figure 3C**, **Supplementary Figure 6A**). This interaction was driven primarily by a simple effect of "MH concern" framing to reduce sharing for MH data [model C1B: PH data, no effect of framing ($F_{2,24461}$=1·18, NS); MH data, effect of framing ($F_{2,24157}$=8·36, p=0·000234); pairwise comparison within MH data, MH concern versus neutral, p=0·00443]. Framing effects were also lessened for geographically broader destinations.

For research purposes (with de-identified data), destination was an extremely strong driver of preference (**Figure 3D–I**). On average, people expressed strong support for sharing to the NHS, academia, or national charities for research purposes. Support and opposition were approximately equally balanced for sharing to profit-making companies researching treatments. There was strong net opposition to sharing to other types of companies, approximately equal to that for sharing publicly. There was a small but significant preference for sharing PH (versus MH) data, and likewise higher preference for sharing structured-only versus free-text data. In the whole-sample analysis (model R1), there were highly significant effects of destination, nature, and detail, plus interactions including destination×nature×detail (**Supplementary Figure 6B**). Framing effects included nature×framing, though simple framing effects were not significant for PH or MH data separately (model R1B).

Sensitivity analyses weighted to UK population demographics (**Supplementary Results**) were consistent with the primary analysis.

5.5. Linkage to non-health data for research

We asked about linking of NHS data to non-health data sources for research. There was net support for all "state" sources and university-held data (**Figure 4**), but net opposition regarding private company data (**Figure 4**). Weighted responses were very similar (**Supplementary Figure 5**).

5.6. Changes related to the COVID-19 pandemic

Following "lockdown", willingness to share identifiable data for clinical purposes increased, with no significant change in the already high preference for local sharing, but progressive increases for sharing to more remote parts of the NHS (model C1, destination×pandemic, $F_{3,169348}$=26·6, VLP; **Figure 5A**).

Willingness to share de-identified data for research purposes generally increased for more-preferred destinations (NHS, academia, charities), except in the "MH concern" framing condition (model R1, destination×pandemic, $F_{5,535334}$=78·2, VLP; **Figure 5B**), but did not change for less-preferred destinations (commercial and public sharing).

Preference for linkage to university data increased (source×pandemic; **Figure 5C; Supplementary Figure 6C**). There was a less consistent decrease in preference for linkage to private data (**Figure 6C, Supplementary Figure 6C**) and police data (model L2; **Figure 6C**).

5.7. Effect sizes and influence of demographic factors

Preference varied according to demographic factors and experience of MH illness. For clinical purposes, there were several demographic effects (model C2, **Figure 6A**). Age was a significant factor, with the age bands most willing to share being 25–44 and 75+, and the 18–24 band being least willing. Males were more

For peer review only - http://bmjopen.bmj.com/site/about/guidelines.xhtml

willing to share data than females. Those of minority ethnicity were less willing to share than those of white ethnicity. Across educational levels, those of Level 3 were most willing and those of Level 4+ least willing. Those of minority religions were less willing to share. Those from the most-deprived IMD quartile were also less willing. There were no significant effects of sexuality ($F_{1,12334}$=2·21, NS), NS-SEC ($F_{4,12335}$=1·32, NS), or nation ($F_{3,12335}$=2·13, NS). Personal experience of MH illness specifically reduced willingness to share MH data for clinical purposes (nature×MH experience).

For research purposes, significant effects were similarly observed for age, gender, ethnicity, religion, and IMD quartile (model R2, **Figure 6B**). The age distribution was clearly U-shaped, with greater willingness to share among the youngest and oldest groups. As before, there was no effect of sexuality ($F_{1,12336}$=1·58, NS). There was no effect of education ($F_{4,12348}$=2·15, p=0·072), but there was an effect of nation (with people living in Wales more willing to share and those in Scotland less so, relative to England), and of socioeconomic status (NS-SEC; **Figure 6B**). People with MH experience were significantly *more* likely to share MH data for research purposes (nature×MH experience, $F_{1,296111}$=6·15, p=0·0132).

For linkage, the patterns were broadly as before (**Figure 6C**, **Supplementary Figure 6C**). Data source strongly influenced preference (education>universities≈police≈housing≈transport>social security>immigration≫private companies). There were also effects as before of age, ethnicity, education, and IMD quartile. There was no effect of gender ($F_{1,12338}$=2·32, NS), religion ($F_{2,12338}$=1·86, NS), nation (F<1), or MH experience (F<1), but there was now an effect of sexuality, with LGBT+ people being less willing for linkage.

5.8. A possible national consent system

We proposed varieties of a national system for patients to decide how their NHS data is used. Participants were most willing to sign up via a web site or in person (**Figure 7A,C**). Willingness was similar regardless of whether consent information was managed by the NHS centrally, a local NHS Trust, or the patient's GP (**Figure 7B**). Overall, 89% of people said they were "likely" or "very likely" to sign up to such a system (**Figure 7D**). Weighted responses were very similar (**Supplementary Figure 7**).

Most people wanted a single NHS system to sign up for participatory research (**Figure 7E; Supplementary Figure 7E**).

There was broad support for the draft consent form and for adding information about contact methods, data security, and management of the consent information (**Figure 7F**; **Supplementary Figure 7F**). Forty percent (unweighted) had been unaware of the NHS National Data Opt-Out.

Comment themes (n>100, **Supplementary Results**) included: the need for clarity around de-identification; the critical importance of healthcare data security; the desirability of data sharing; that opt-outs should be more prominent (or default) or linked to the NHS National Data Opt-Out; that profit-making use should not happen or that the NHS/patients should benefit from such profits; that clinical users should be specified in more detail; research users likewise; and that healthcare data should not be available to private or third-party companies without specific permission.

6. DISCUSSION

6.1. Summary

Many respondents believed that health data is shared UK-wide for clinical purposes without explicit consent, when sharing is usually more limited. A majority supported such sharing, though a significant minority

For peer review only - http://bmjopen.bmj.com/site/about/guidelines.xhtml

opposed it. Geographically broad sharing was endorsed, though with stronger support for more local destinations. People preferred to share PH (versus MH) data but this was less important than the destination.

For research, with de-identified data, there was strong net support for sharing without explicit consent to the NHS, academic research institutions, and research charities. There was net ambivalence regarding private companies researching treatments, and strong net opposition for sharing to other companies or publicly. There was a small preference for sharing PH over MH data (a smaller difference than for clinical purposes), and greater support for structured-only data over de-identified free text. There was net support for research linkage to state and university data sources, but opposition regarding data held by private companies.

Framing statements influenced MH/PH preferences, but only to a small degree. Age, gender, ethnicity, education, religion, and IMD were associated with willingness to have health data shared or linked, though not nearly as strongly as destination/source. Personal experience of MH conditions was associated with reduced willingness to share MH data for clinical purposes, but greater willingness to share it for research. After COVID-19 lockdown there was greater willingness regarding already-preferred destinations.

Respondents endorsed a suggested UK-wide system allowing patients to control the clinical/research uses of their data and to sign up for participatory research. They frequently emphasized the importance of data security and that NHS data should not be made available to private or third-party companies without specific permission.

6.2. Strengths and weaknesses

Strengths include patient/public involvement in the study design; the largest such study to date by 1–3 orders of magnitude,[15,17,19–21,42] giving high power; sensitivity analyses weighted to population demographics; detailed questions about data sharing for clinical/research purposes, including about the type of data and the destination, plus linkage to non-health data; a randomized framing experiment to control and measure this source of variation; quantitative analysis including of relative effect sizes; consultation on ways to improve the current situation; and serendipitous examination of the effects of COVID-19 on data sharing views.

The major weakness is that the sample remained under-representative of some groups despite weighting, with potential for unmeasured selection (including self-selection) bias, reducing generalisability.

6.3. Destination and purpose

The Caldicott framework (2013) and review (2016), regarding safe information sharing for direct clinical care, included the principle that the "duty to share information can be as important as the duty to protect patient confidentiality"[3] and noted that information was often not shared when it should be, for fear of inappropriate disclosure.[12] That was despite legislation creating a duty upon providers to share information with professionals when that is likely to facilitate the individual's health or social care, disclosure is in their best interests, and they do not or are not likely to object.[43] That is in essence an opt-out system. This legislation conflicted with some prior studies of public opinion.[44] The review noted low public understanding around how health information is used, but "an expectation that information is shared for direct care".[12] We observed net support for such sharing that varied with geographical destination and was by no means universal, but was nevertheless strong.

In relation to research and other non-clinical activities, the recommendation that people be able to opt out from personal confidential data being used beyond their own direct care[12] led to the NHS National Data Opt-Out.[45,46] This relates to the use of CPI (identifiable information) for purposes such as research, conducted under NHS Act Section 251 (s251) approvals.[1,47] It does not apply to direct clinical care, local

For peer review only - http://bmjopen.bmj.com/site/about/guidelines.xhtml

audit or service evaluation, or de-identified information.[45,46] Our study and others show it remains unknown to many.[42] Furthermore, it is not simple and we suspect many do not fully understand its scope. Conversely, from the researcher's perspective, s251 approval is often still required for linkage studies in which researchers never see identifiable information: there is no standardized "trusted third party" system for centralized linkage of identifiable information, and inconsistent adoption of de-identified linkage methods.

"Destination" was by far the strongest driver of preference for sharing and linkage. This pattern is established: willingness to allow researchers/clinicians access to health data, but far greater reservations about industry.[48,49] An important basis for this is mistrust of the security and/or motives of commercial organizations,[25,26,50] as our participants noted.

6.4. Demographic effects

A common demographic theme was that minority groups (of ethnicity, religion, and sexuality) and deprived groups were less willing to share. This might reflect experience of disadvantage to, or discrimination against, these groups.[51,52] Ethnicity has had mixed effects on preference for national electronic health record (EHR) systems.[19] In our study, age effects were generally biphasic, with higher willingness amount the youngest and eldest. Youth may be associated with familiarity with data and/or greater support for EHRs,[53] and older age with an increasing burden of illness, itself associated with support for national EHR systems.[53] Educational effects were relatively inconsistent. Males were slightly more willing to share than females. Similar results have been observed before,[42] but not always;[53,54] one reason might be gender-based healthcare discrimination.[55] Higher support for research sharing in Wales may relate to established national research systems there;[56,57] the reasons for reduced willingness in Scotland are unclear, but similar systems there are younger.[58] Those with personal experience of MH illness were less willing to share identifiable MH data for direct care. This may reflect experience of discrimination or stigma[59,60]—which can have disproportionate effects in subgroups.[60,61] However, the same people were more willing to share de-identified MH data for research, potentially reflecting increased prioritization of MH research.[62]

Demographic variations in preference may reflect differences in perception of current data rules or security practices, reasons for concern about uses of health data, or degree of concern. UK law prohibits variation of policy according to these factors.[63] Better understanding and public information may be required to address these groups' concerns,[11,64] but improvements in health equity are also required.[17,51] However, the effect sizes of these demographic predictors were not large enough to override the net support for data sharing, given the right destinations.

6.5. Framing and pandemic effects

We observed small but significant framing effects.[28] Our framing statements were true and non-alarmist, so real-world framing effects might sometimes be larger. Others have observed larger effects via "loss framing" (emphasizing the potential adverse consequences of not consenting over the potential benefits of consenting), and through other manipulations like the placement of framing statements.[65] Media coverage of health data sharing is influential.[66,67] Despite best intentions it is impossible to avoid framing effects entirely,[28] so those presenting information should be aware of these whilst presenting accurately the risks and benefits of data sharing/linkage.

During COVID-19, despite press coverage[31] of an enforced increase in sharing,[30,32] support for sharing/linkage increased—but only for some already-favoured destinations. Publicity regarding NHS care[68] and research regarding COVID-19[69,70] may have driven the increase in support for sharing with the NHS, universities, and research charities.

For peer review only - http://bmjopen.bmj.com/site/about/guidelines.xhtml

6.6. Conclusions

Participants supported a central system for patients to control the uses of their data, and likewise a single NHS mechanism to sign up for active research participation. There is a trade-off between the scientific desirability of everyone contributing de-identified data, including to avoid bias,[71,72] and the desirability of individual control over data use.[73] A reasonable balance might be a central system to opt out from identifiable clinical use, identifiable (s251) research use, or de-identified research use of one's data, and to opt in for participatory research. This would complement efforts to improve people's access to their own data.[74]

The majority support observed for clinical sharing without explicit consent perhaps makes such sharing reasonable as a default (opt-out) position, given the potential advantages for many people's own care, subject to strict IG rules (who has access, when). However, a significant minority opposed this, mandating at least a public information campaign about opt-outs if this were to occur.

There was strong net support for NHS, academic, and charity researchers accessing de-identified health data. A standard method is a trusted research environment (TRE).[24,56,75,76] Approved researchers come "into" the secure environment to interact with relevant data (e.g. pseudonymised; **Figure 1**). After analysis, aggregation, and other statistical disclosure control (SDC),[77] results go "out" for publication (**Figure 1**).

In contrast, respondents did not support research sharing to private companies. Some have suggested this is addressable in part by public education.[11] We suggest respecting public preference, and not giving commercial organisations direct access to patient-level NHS data for research, even de-identified, without consent. (This is distinct from the common NHS practice of employing companies, such as EHR software providers, to manage NHS data securely for clinical purposes.) We think that this does not rule out all industrial research uses of data, which could happen according to at least three methods. The first is via consent, as for commercial treatment trials. Secondly, companies could collaborate with NHS/academic researchers. For example, an artificial intelligence company could provide an untrained algorithm; NHS staff could train it on patient-level data; the company could receive a trained algorithm back whilst never having access to the data (assuming verification that the algorithm cannot "embed" detailed data features during training). Thirdly, methods exist whereby software queries come "in" to the TRE, and semi-automatic or automatic SDC occurs before results go "out".[78–80] This allows research to take place without researchers having access to patient-level data, and can also support "federated" queries across sites. Data that have undergone suitable SDC (e.g. aggregation) can be published, and are therefore suitable for industrial access if desired. Regardless, as our participants commented, the NHS might charge for such access,[11,81] and full transparency is essential. Formal, consultation-based standards governing this NHS–commercial interface would be desirable.

Governance of UK health data must be transparent and reflect the views of patients.[11,16] As the UK Government seeks to change data legislation[82] and emphasize health data in its science strategy,[83] we hope this study contributes to the conversation.

7. ACKNOWLEDGEMENTS

We thank all those who took part in the study and gave their views. We thank all the investigators, local collaborators, local NIHR CRN staff, and health care staff across the UK who supported the study. We thank also Elizabeth Rotherham (research advisory group member) for help with design; Nicola Gleadle for practical support; Jane Gaffa, Rachel Kyd, and Mary-Beth Sherwood for Research and Development support; Sally-Anne Hurford and Ruth Hudson for NIHR CRN advice; Chess Denman for investigator

For peer review only - http://bmjopen.bmj.com/site/about/guidelines.xhtml

approval; Laura Marshall for CPFT publicity; Hannah Clarke for helpful comments; REC and HRA staff for ethical and regulatory approvals; the Cambridge Autism Research Database for additional publicity.

8. DATA AVAILABILITY

After removal of all free text, anonymised data are available from the University of Cambridge Data Repository at https://doi.org/10.17863/CAM.75784, with participants' consent.[84]

9. AUTHOR CONTRIBUTIONS

RNC conceived the study. LAJ, JRN, PHA, MRG, MP and RNC designed it (with ER). LAJ coordinated its execution. JMF advised on REDCap implementation and execution. RNC, LAJ, and JMF had access to the original data; the final data set is public. RNC analysed the data. RNC and LAJ drafted the manuscript. All authors edited the manuscript and approved submission.

10. FUNDING

Supported by the UK Medical Research Council (Mental Health Data Pathfinder award MC_PC_17213 to RNC). Recruitment was supported by the NIHR CRN and the REDCap installation was supported in part by the NIHR Cambridge Biomedical Research Centre (BRC-1215-20014); the views expressed are those of the authors and not necessarily those of the NHS, the NIHR, or the Department of Health and Social Care.

11. COMPETING INTERESTS

The authors report no competing interests.

For peer review only

For peer review only - http://bmjopen.bmj.com/site/about/guidelines.xhtml

12. REFERENCES

1 UK. National Health Service Act 2006. 2006. https://www.legislation.gov.uk/ukpga/2006/41

2 UK General Medical Council. Confidentiality: good practice in handling patient information. 2017. http://www.gmc-uk.org/guidance/ethical_guidance/confidentiality.asp

3 Caldicott F. Information: To share or not to share? The Information Governance Review. 2013.https://www.gov.uk/government/publications/the-information-governance-review

4 UK Department of Health. Confidentiality: NHS Code of Practice. 2003.https://www.gov.uk/government/publications/confidentiality-nhs-code-of-practice

5 UK. Data Protection Act 2018. 2018. http://www.legislation.gov.uk/ukpga/2018/12/contents/enacted

6 European Parliament and Council. Regulation (EU) 2016/679 (General Data Protection Regulation). *Off J Eur Union* 2016;**L119**:1–88.

7 UK. Mental Capacity Act 2005. 2005. https://www.legislation.gov.uk/ukpga/2005/9/contents

8 Coulter A. Patient trust in plans to share primary care data. *BMJ* 2021;**373**:n1413. doi:10.1136/bmj.n1413

9 NHS England. The NHS Constitution for England. 2015.https://www.gov.uk/government/publications/the-nhs-constitution-for-england

10 Culnane C, Rubinstein BIP, Teague V. Health Data in an Open World. *ArXiv171205627 Cs* Published Online First: 15 December 2017.http://arxiv.org/abs/1712.05627

11 Ghafur S, Fontana G, Halligan J, *et al.* NHS Data: Maximising its impact on the health and wealth of the United Kingdom. Imperial College London 2020. https://doi.org/10.25561/76409

12 National Data Guardian for Health and Care. Review of Data Security, Consent and Opt-Outs. 2016.https://www.gov.uk/government/uploads/system/uploads/attachment_data/file/535024/data-security-review.PDF

13 NHS England. New local health and care partnerships could save lives. 2018.https://www.england.nhs.uk/2018/05/new-local-health-and-care-partnerships-could-save-lives/ (accessed 23 Aug 2019).

14 NHS England, Local Government Association. Local Health and Care Record Exemplars. 2018.https://www.england.nhs.uk/publication/local-health-and-care-record-exemplars/ (accessed 23 Aug 2019).

15 Aitken M, de St Jorre J, Pagliari C, *et al.* Public responses to the sharing and linkage of health data for research purposes: a systematic review and thematic synthesis of qualitative studies. *BMC Med Ethics* 2016;**17**:73. doi:10.1186/s12910-016-0153-x

16 Understanding Patient Data, Ada Lovelace Institute. Foundations of fairness: where next for NHS health data partnerships? 2020. https://understandingpatientdata.org.uk/sites/default/files/2020-03/Foundations%20of%20Fairness%20-%20Summary%20and%20Analysis.pdf (accessed 20 Apr 2020).

17 Hopkins H, Kinsella S, van Mil A. Foundations of fairness: views on uses of NHS patients' data and NHS operational data. Hopkins Van Mil: Creating Conditions Ltd 2020.

For peer review only

For peer review only - http://bmjopen.bmj.com/site/about/guidelines.xhtml

https://understandingpatientdata.org.uk/sites/default/files/2020-03/Foundations%20of%20Fairness%20-%20Full%20Research%20Report.pdf (accessed 20 Apr 2020).

18 Understanding Patient Data. Public attitudes to patient data use: a summary of existing research. 2018.https://understandingpatientdata.org.uk/sites/default/files/2019-05/Public%20attitudes%20key%20themes%200.pdf (accessed 23 Aug 2019).

19 Stockdale J, Cassell J, Ford E. 'Giving something back': A systematic review and ethical enquiry into public views on the use of patient data for research in the United Kingdom and the Republic of Ireland. *Wellcome Open Res* 2018;**3**:6. doi:10.12688/wellcomeopenres.13531.2

20 Aitken M, McAteer G, Davidson S, *et al.* Public Preferences regarding Data Linkage for Health Research: A Discrete Choice Experiment. *Int J Popul Data Sci* 2018;**3**:429. doi:10.23889/ijpds.v3i1.429

21 Ghafur S, Van Dael J, Leis M, *et al.* Public perceptions on data sharing: key insights from the UK and the USA. *Lancet Digit Health* 2020;**2**:e444–6. doi:10.1016/S2589-7500(20)30161-8

22 Henderson C, Evans-Lacko S, Thornicroft G. Mental Illness Stigma, Help Seeking, and Public Health Programs. *Am J Public Health* 2013;**103**:777–80. doi:10.2105/AJPH.2012.301056

23 Piotrowski P, Gondek TM, Królicka-Deręgowska A, *et al.* Causes of mortality in schizophrenia: An updated review of European studies. *Psychiatr Danub* 2017;**29**:108–20.

24 Downs JM, Ford T, Stewart R, *et al.* An approach to linking education, social care and electronic health records for children and young people in South London: a linkage study of child and adolescent mental health service data. *BMJ Open* 2019;**9**:e024355. doi:10.1136/bmjopen-2018-024355

25 Sterckx S, Rakic V, Cockbain J, *et al.* 'You hoped we would sleep walk into accepting the collection of our data': controversies surrounding the UK care.data scheme and their wider relevance for biomedical research. *Med Health Care Philos* Published Online First: 18 August 2015. doi:10.1007/s11019-015-9661-6

26 Powles J, Hodson H. Google DeepMind and healthcare in an age of algorithms. *Health Technol* 2017;:1–17. doi:10.1007/s12553-017-0179-1

27 Gayle D. GPs urged to refuse to hand over patient details. The Guardian. 2021.https://www.theguardian.com/society/2021/jun/01/gps-urged-to-refuse-to-hand-over-patient-details-to-nhs-digital (accessed 29 Jun 2021).

28 Tversky A, Kahneman D. The framing of decisions and the psychology of choice. *Science* 1981;**211**:453–8. doi:10.1126/science.7455683

29 Harris PA, Taylor R, Thielke R, *et al.* Research electronic data capture (REDCap)--a metadata-driven methodology and workflow process for providing translational research informatics support. *J Biomed Inform* 2009;**42**:377–81. doi:10.1016/j.jbi.2008.08.010

30 Department for Health and Social Care, Hancock M. Coronavirus (COVID-19): notification to organisations to share information. 2020.https://www.gov.uk/government/publications/coronavirus-covid-19-notification-of-data-controllers-to-share-information

31 Lewis P, Conn D, Pegg D. UK government using confidential patient data in coronavirus response. The Guardian. 2020.https://www.theguardian.com/world/2020/apr/12/uk-government-using-confidential-patient-data-in-coronavirus-response (accessed 12 Apr 2020).

For peer review only - http://bmjopen.bmj.com/site/about/guidelines.xhtml

32 NHS Digital. SCR coronavirus (COVID-19) supplementary privacy notice. NHS Digit. 2020.https://digital.nhs.uk/services/summary-care-records-scr/scr-coronavirus-covid-19-supplementary-privacy-notice (accessed 27 Jun 2021).

33 R Core Team. *R: A language and environment for statistical computing*. Vienna, Austria: : R Foundation for Statistical Computing 2019. http://www.R-project.org/

34 Cardinal RN, Aitken MRF. *ANOVA for the Behavioural Sciences Researcher*. Mahwah, N.J: : L. Erlbaum 2006.

35 Myers JL, Well AD. *Research design and statistical analysis*. Second. Mahwah, New Jersey: : Lawrence Erlbaum Associates 2003.

36 Maxwell SE, Delaney HD. *Designing experiments and analyzing data: a model comparison perspective*. Second. Mahwah, New Jersey: : Lawrence Erlbaum Associates 2004.

37 NHS Scotland. Intra NHS Scotland Information Sharing Accord. 2010.https://www.informationgovernance.scot.nhs.uk/wp-content/uploads/2020/06/2020-06-17-Intra-NHS-Scotland-Sharing-Accord-v2.0.pdf

38 Morris LMM, Brown C, Williamson M, *et al.* The Scottish Emergency Care Summary--an evaluation of a national shared record system aiming to improve patient care: technology report. *Inform Prim Care* 2012;**20**:41–9. doi:10.14236/jhi.v20i1.46

39 NIDirect. Northern Ireland Electronic Care Record (NIECR) | nidirect. 2015.https://www.nidirect.gov.uk/articles/northern-ireland-electronic-care-record-niecr (accessed 6 Jul 2021).

40 Greenhalgh T, Stramer K, Bratan T, *et al.* Adoption and non-adoption of a shared electronic summary record in England: a mixed-method case study. *BMJ* 2010;**340**:c3111. doi:10.1136/bmj.c3111

41 Auditor General for Wales. Informatics systems in NHS Wales. 2018.https://senedd.wales/laid%20documents/agr-ld11353/agr-ld11353-e.pdf

42 Atkin C, Crosby B, Dunn K, *et al.* Perceptions of anonymised data use and awareness of the NHS data opt-out amongst patients, carers and healthcare staff. *Res Involv Engagem* 2021;**7**:40. doi:10.1186/s40900-021-00281-2

43 UK. Health and Social Care (Safety and Quality) Act 2015. 2015. http://www.legislation.gov.uk/ukpga/2015/28/contents

44 nef (The New Economics Foundation), Centre for Science Education at Sheffield Hallam University. Who sees what: exploring public views on personal electronic health records. 2010. https://neweconomics.org/uploads/files/2cb17ab59382fe7c67_bfm6bdoas.pdf

45 NHS. National Data Opt-out: Factsheet 2 – When it applies. 2018.https://digital.nhs.uk/binaries/content/assets/website-assets/services/national-data-opt-out-programme/guidance-for-health-and-care-staff/nhs-factsheet-2-23052018.pdf (accessed 26 Jul 2019).

46 NHS Digital. Understanding the national data opt-out. 2019.https://web.archive.org/web/20200804083843/https://digital.nhs.uk/services/national-data-opt-out/understanding-the-national-data-opt-out

47 UK. The Health Service (Control of Patient Information) Regulations 2002. 2002. http://www.legislation.gov.uk/uksi/2002/1438/contents/made

For peer review only

For peer review only - http://bmjopen.bmj.com/site/about/guidelines.xhtml

48 Beange I, Kirkham EJ, Fletcher-Watson S, *et al.* Using a knowledge exchange event to assess study participants' attitudes to research in a rapidly evolving research context. *Wellcome Open Res* 2020;**5**:24. doi:10.12688/wellcomeopenres.15651.2

49 Chico V, Hunn A, Taylor M. Public views on sharing anonymised patient-level data where there is a mixed public and private benefit. Health Research Authority; University of Sheffield School of Law 2019.

50 Helm T. Revealed: how drugs giants can access your health records. *The Guardian* 2020.https://www.theguardian.com/technology/2020/feb/08/fears-over-sale-anonymous-nhs-patient-data (accessed 8 Feb 2020).

51 Gazard B, Chui Z, Harber-Aschan L, *et al.* Barrier or stressor? The role of discrimination experiences in health service use. *BMC Public Health* 2018;**18**:1354. doi:10.1186/s12889-018-6267-y

52 Wu Z, Schimmele CM. Perceived religious discrimination and mental health. *Ethn Health* 2019;:1–18. doi:10.1080/13557858.2019.1620176

53 Luchenski SA, Reed JE, Marston C, *et al.* Patient and public views on electronic health records and their uses in the United kingdom: cross-sectional survey. *J Med Internet Res* 2013;**15**:e160. doi:10.2196/jmir.2701

54 Haeusermann T, Greshake B, Blasimme A, *et al.* Open sharing of genomic data: Who does it and why? *PloS One* 2017;**12**:e0177158. doi:10.1371/journal.pone.0177158

55 SteelFisher GK, Findling MG, Bleich SN, *et al.* Gender discrimination in the United States: Experiences of women. *Health Serv Res* 2019;**54 Suppl 2**:1442–53. doi:10.1111/1475-6773.13217

56 Lyons RA, Jones KH, John G, *et al.* The SAIL databank: linking multiple health and social care datasets. *BMC Med Inform Decis Mak* 2009;**9**:3. doi:10.1186/1472-6947-9-3

57 Lyons J, Akbari A, Torabi F, *et al.* Understanding and responding to COVID-19 in Wales: protocol for a privacy-protecting data platform for enhanced epidemiology and evaluation of interventions. *BMJ Open* 2020;**10**:e043010. doi:10.1136/bmjopen-2020-043010

58 Hagger-Johnson G. Opportunities for longitudinal data linkage in Scotland. *Scott Med J* 2016;**61**:136–45. doi:10.1177/0036933015575214

59 Thornicroft G, Mehta N, Clement S, *et al.* Evidence for effective interventions to reduce mental-health-related stigma and discrimination. *Lancet Lond Engl* 2016;**387**:1123–32. doi:10.1016/S0140-6736(15)00298-6

60 Clement S, Schauman O, Graham T, *et al.* What is the impact of mental health-related stigma on help-seeking? A systematic review of quantitative and qualitative studies. *Psychol Med* 2015;**45**:11–27. doi:10.1017/S0033291714000129

61 Gary FA. Stigma: barrier to mental health care among ethnic minorities. *Issues Ment Health Nurs* 2005;**26**:979–99. doi:10.1080/01612840500280638

62 Sangill C, Buus N, Hybholt L, *et al.* Service user's actual involvement in mental health research practices: A scoping review. *Int J Ment Health Nurs* 2019;**28**:798–815. doi:10.1111/inm.12594

63 UK. Equality Act 2010. 2010.https://www.legislation.gov.uk/ukpga/2010/15/contents (accessed 5 Jul 2021).

For peer review only - http://bmjopen.bmj.com/site/about/guidelines.xhtml

64 Tully MP, Bozentko K, Clement S, *et al.* Investigating the Extent to Which Patients Should Control Access to Patient Records for Research: A Deliberative Process Using Citizens' Juries. *J Med Internet Res* 2018;**20**:e112. doi:10.2196/jmir.7763

65 Sakshaug JW, Schmucker A, Kreuter F, *et al.* The Effect of Framing and Placement on Linkage Consent. *Public Opin Q* 2019;**83**:289–308. doi:10.1093/poq/nfz018

66 Gollust SE, Fowler EF, Niederdeppe J. Television News Coverage of Public Health Issues and Implications for Public Health Policy and Practice. *Annu Rev Public Health* 2019;**40**:167–85. doi:10.1146/annurev-publhealth-040218-044017

67 Ford E, Kazempour Y, Cooper MJF, *et al.* Media content analysis of general practitioners' reactions to care.data expressed in the media: what lessons can be learned for future NHS data-sharing initiatives? *BMJ Open* 2020;**10**:e038006. doi:10.1136/bmjopen-2020-038006

68 Prime Minister's Office. PM address to the nation on coronavirus: 23 March 2020. 2020.https://www.gov.uk/government/speeches/pm-address-to-the-nation-on-coronavirus-23-march-2020 (accessed 23 Mar 2020).

69 RECOVERY Collaborative Group, Horby P, Lim WS, *et al.* Dexamethasone in Hospitalized Patients with Covid-19. *N Engl J Med* 2021;**384**:693–704. doi:10.1056/NEJMoa2021436

70 Horby P, Lim WS, Emberson J, *et al.* Effect of Dexamethasone in Hospitalized Patients with COVID-19 – Preliminary Report. *medRxiv* 2020;:2020.06.22.20137273. doi:10.1101/2020.06.22.20137273

71 Elwood JM, Marshall RJ, Tin ST, *et al.* Bias in survival estimates created by a requirement for consent to enter a clinical breast cancer registry. *Cancer Epidemiol* 2019;**58**:178–83. doi:10.1016/j.canep.2018.12.005

72 Morgan K, Page N, Brown R, *et al.* Sources of potential bias when combining routine data linkage and a national survey of secondary school-aged children: a record linkage study. *BMC Med Res Methodol* 2020;**20**:178. doi:10.1186/s12874-020-01064-1

73 Kariotis T, Ball MP, Greshake Tzovaras B, *et al.* Emerging Health Data Platforms: From Individual Control to Collective Data Governance. Published Online First: 2020. doi:10.1017/dap.2020.14

74 Wyatt D, Lampon S, McKevitt C. Delivering healthcare's 'triple aim': electronic health records and the health research participant in the UK National Health Service. *Sociol Health Illn* 2020;**42**:1312–27. doi:10.1111/1467-9566.13101

75 UK Office for National Statistics. ONS Secure Research Service (SRS). 2019.https://www.ons.gov.uk/aboutus/whatwedo/paidservices/virtualmicrodatalaboratoryvml (accessed 23 Jul 2019).

76 Stewart R, Soremekun M, Perera G, *et al.* The South London and Maudsley NHS Foundation Trust Biomedical Research Centre (SLAM BRC) case register: development and descriptive data. *BMC Psychiatry* 2009;**9**:51. doi:10.1186/1471-244X-9-51

77 Griffiths E, Greci C, Kotrotsios Y, *et al.* Handbook on Statistical Disclosure Control for Outputs. 2019.https://ukdataservice.ac.uk/media/622521/thf_datareport_aw_web.pdf

78 Williamson EJ, Walker AJ, Bhaskaran K, *et al.* Factors associated with COVID-19-related death using OpenSAFELY. *Nature* 2020;**584**:430–6. doi:10.1038/s41586-020-2521-4

For peer review only

79 Wolfson M, Wallace SE, Masca N, *et al.* DataSHIELD: resolving a conflict in contemporary bioscience--performing a pooled analysis of individual-level data without sharing the data. *Int J Epidemiol* 2010;**39**:1372–82. doi:10.1093/ije/dyq111

80 Gaye A, Marcon Y, Isaeva J, *et al.* DataSHIELD: taking the analysis to the data, not the data to the analysis. *Int J Epidemiol* 2014;**43**:1929–44. doi:10.1093/ije/dyu188

81 Fontana G, Ghafur S, Torne L, *et al.* Ensuring that the NHS realises fair financial value from its data. *Lancet Digit Health* 2020;**2**:e10–2. doi:10.1016/S2589-7500(19)30225-0

82 UK Department of Health and Social Care. Data saves lives: reshaping health and social care with data (draft). GOV.UK. 2021.https://www.gov.uk/government/publications/data-saves-lives-reshaping-health-and-social-care-with-data-draft (accessed 6 Jul 2021).

83 UK Department for Business, Energy & Industrial Strategy. Life Sciences Vision (policy paper). 2021.https://assets.publishing.service.gov.uk/government/uploads/system/uploads/attachment_data/file/1000030/life-sciences-vision.pdf

84 Higher Education Funding Council for England, Research Councils UK, Universities UK, *et al.* Concordat on Open Research Data. 2016.https://www.ukri.org/files/legacy/documents/concordatonopenresearchdata-pdf/

For peer review only

For peer review only - http://bmjopen.bmj.com/site/about/guidelines.xhtml

13. FIGURE LEGENDS

Figure 1. Classifying health data according to identifiability. At the "patient" level, the Venn diagram shows the overlaps between information that directly identifies a person, research identifiers (RIDs or pseudonyms), and health information, with simple examples. Anonymous health data may also existing in aggregated form, distinct from patient-level data; this aggregated form is the norm for public distribution. The level of identification risk and the research information governance requirements vary with the resulting categories of data, shown in the legend. All examples are fictional. (DOB, date of birth.)

Figure 2. (A) Understanding of how health data are shared identifiably without explicit consent for clinical purposes, and **(B)** preference as to what should happen. The denominator for percentages is the number of people who answered each question.

Figure 3. Participants' self-reported likelihood of sharing mental and physical health data without explicit consent for clinical purposes (identifiably) or research (de-identified), according to destination, nature (MH versus PH), framing statement, and for research purposes also the level of detail (structured only versus with free text). The denominator for percentages is the number of people who answered each question. In plots C, F, and H, the abscissa is the mean of responses coded as –2 very unlikely, –1 unlikely, 0 not sure, +1 likely, +2 very likely. Analyses were from models C1 and R1 as described in the Methods. (De-id., de-identified; MH, mental health; PH, physical health; #####, $p<10^{-5}$ for main effect of destination; †††††, $p<10^{-5}$ for main effect of nature, with bar length showing mean difference between MH and PH; ** $p<0.01$ and ***** $p<10^{-5}$ for framing×nature interaction, with bar showing the mean difference between "MH concern" and "neutral"; §§§§§, $p<10^{-5}$, main effect of detail, comparing panel F with panel I, with bar length showing the mean difference between structured and free-text conditions.) See **Supplementary Figure 4** for corresponding weighted analysis.

Figure 4. Participants' willingness for their NHS data to be linked to non-health data of different kinds for research. The denominator for percentages is the number of people who answered each question. See **Supplementary Figure 5** for corresponding weighted analysis.

Figure 5. Change in preference in relation to the COVID-19 pandemic. **(A)** Clinical/identifiable data sharing, by destination. Dependent variable (preference for sharing) as for **Figure 3C**, now shown on the ordinate (*y*) axis. **(B)** Research/de-identified data sharing, by detail (structured versus free text) and destination. Dependent variable as for **Figure 3F,H**. **(C)** Linkage for research, by non-NHS data source type. Dependent variable coded as –1 no, 0 not sure, +1 yes. (Error bars show ±1 SEM; ***** $p<10^{-5}$, **** $p<10^{-4}$, *** $p<10^{-3}$, ** $p<10^{-2}$, * $p<0.05$ by two-sample *t* test Šidák-corrected for multiple comparisons; NS, not significant; ##### $p \lll 10^{-5}$, destination×pandemic interaction.)

Figure 6. Effect sizes for **(A)** clinical data sharing via statistical model C2, **(B)** research data sharing via model R2, and **(C)** linkage via model L2. These models include only those participants who supplied full demographic information, to allow analysis by demographics; compare **Supplementary Figure 6** (all participants). Only those model terms with a significant *F* test are shown. Effect sizes with 95% confidence intervals are shown for each level as uncorrected pairwise comparisons to a reference category within each term (note the difference in what is being tested pairwise versus the omnibus *F* test for the term; see **Supplementary Methods**). ● $p<\alpha$; ○ NS.

Figure 7. Views on a national data sharing consent system. The denominator for percentages is the number of people who answered each question. See **Supplementary Figure 7** for corresponding weighted analysis.

For peer review only - http://bmjopen.bmj.com/site/about/guidelines.xhtml

Figure 1

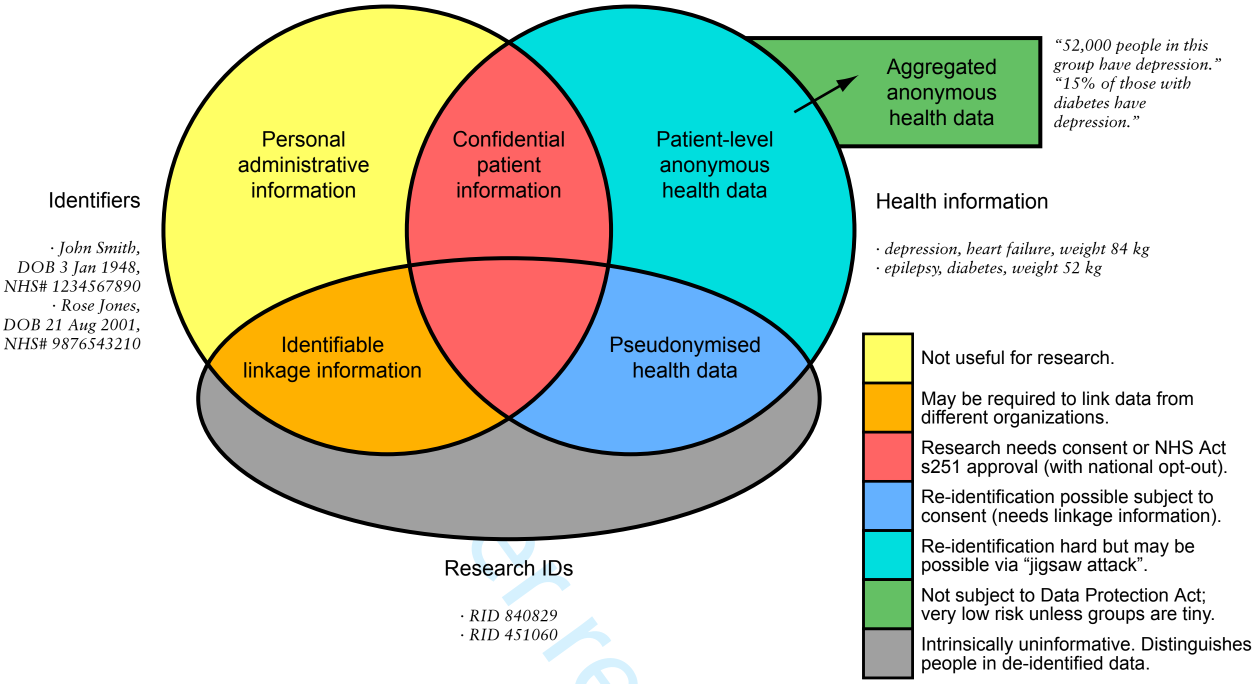
Personal administrative information
Confidential patient information
Patient-level anonymous health data
Aggregated anonymous health data
“52,000 people in this group have depression.”
“15% of those with diabetes have depression.”
Identifiers
· John Smith, DOB 3 Jan 1948, NHS# 1234567890
· Rose Jones, DOB 21 Aug 2001, NHS# 9876543210
Health information
· depression, heart failure, weight 84 kg
· epilepsy, diabetes, weight 52 kg
Identifiable linkage information
Pseudonymised health data
Research IDs
· RID 840829
· RID 451060
Not useful for research.
May be required to link data from different organizations.
Research needs consent or NHS Act s251 approval (with national opt-out).
Re-identification possible subject to consent (needs linkage information).
Re-identification hard but may be possible via “jigsaw attack”.
Not subject to Data Protection Act; very low risk unless groups are tiny.
Intrinsically uninformative. Distinguishes people in de-identified data.

Figure 2

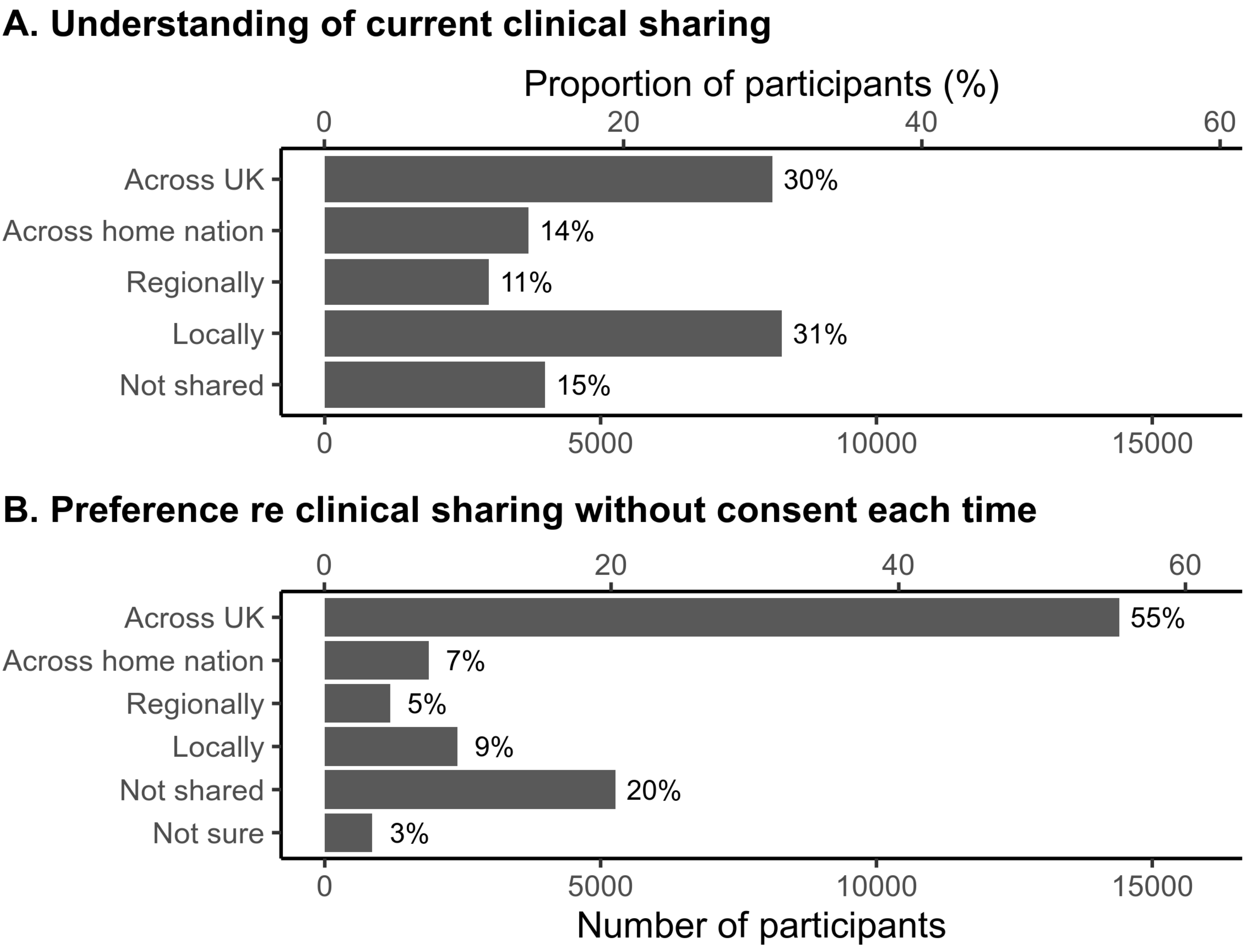
A. Understanding of current clinical sharing
Proportion of participants (%)
0
20
40
60
Across UK
Across home nation
Regionally
Locally
Not shared
30%
14%
11%
31%
15%
0
5000
10000
15000
B. Preference re clinical sharing without consent each time
0
20
40
60
Across UK
Across home nation
Regionally
Locally
Not shared
Not sure
55%
7%
5%
9%
20%
3%
0
5000
10000
15000
Number of participants

Figure 3

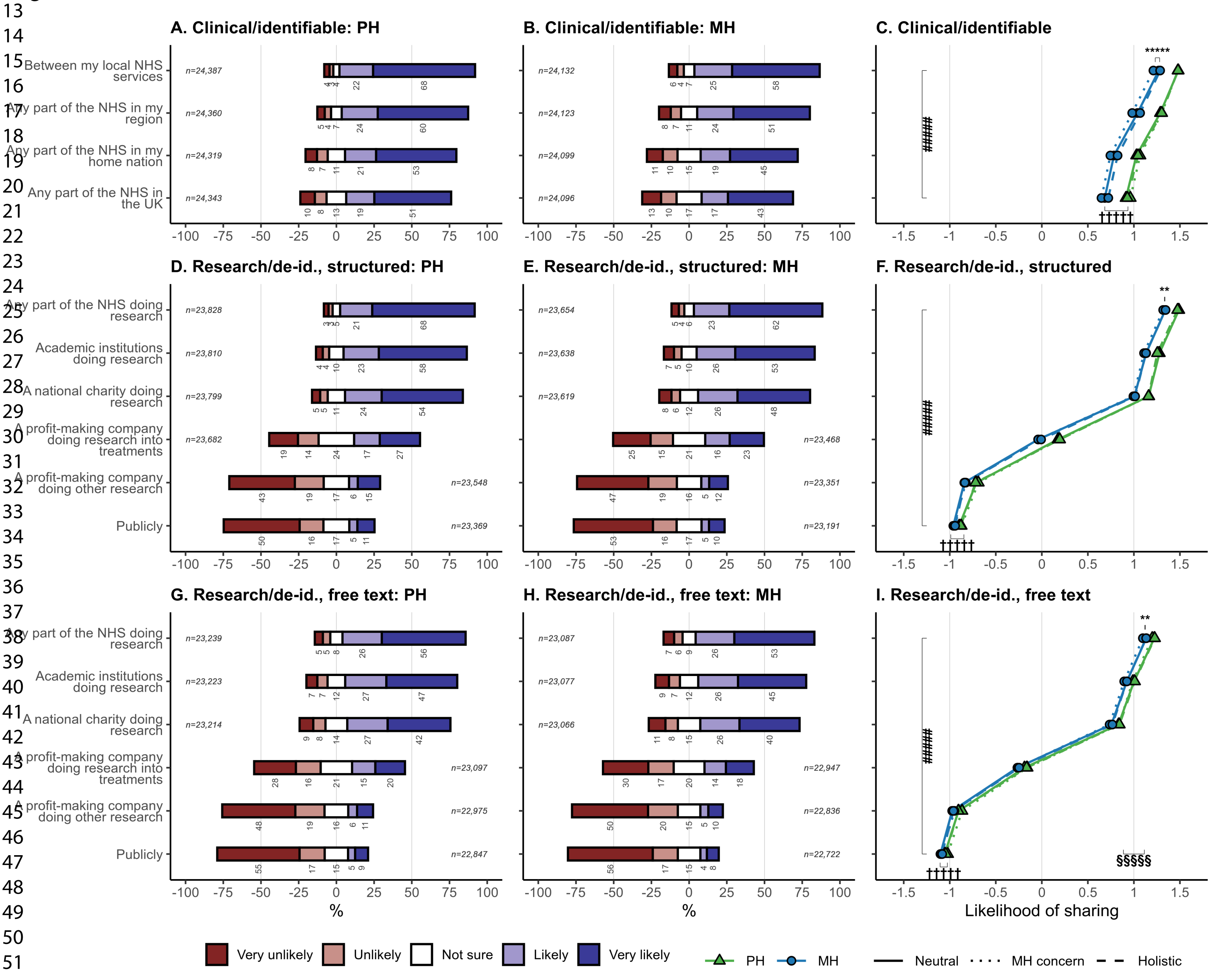
A. Clinical/identifiable: PH
B. Clinical/identifiable: MH
C. Clinical/identifiable
Between my local NHS services
Any part of the NHS in my region
Any part of the NHS in my home nation
Any part of the NHS in the UK
D. Research/de-id., structured: PH
E. Research/de-id., structured: MH
F. Research/de-id., structured
Any part of the NHS doing research
Academic institutions doing research
A national charity doing research
A profit-making company doing research into treatments
A profit-making company doing other research
Publicly
G. Research/de-id., free text: PH
H. Research/de-id., free text: MH
I. Research/de-id., free text
%
Likelihood of sharing
Very unlikely
Unlikely
Not sure
Likely
Very likely
PH
MH
Neutral
MH concern
Holistic

Figure 4

De-identified linkage to non-health data

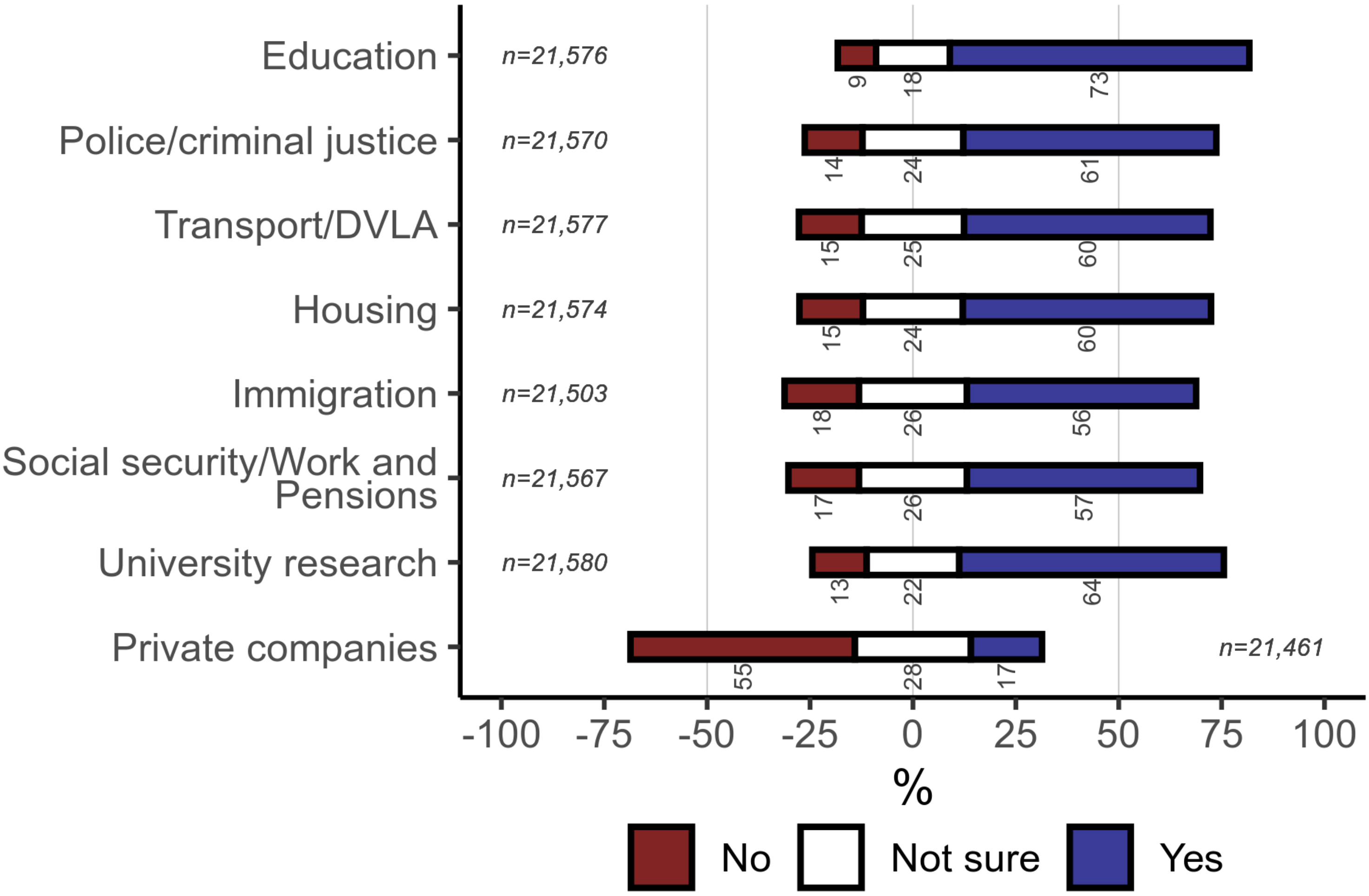

Figure 5

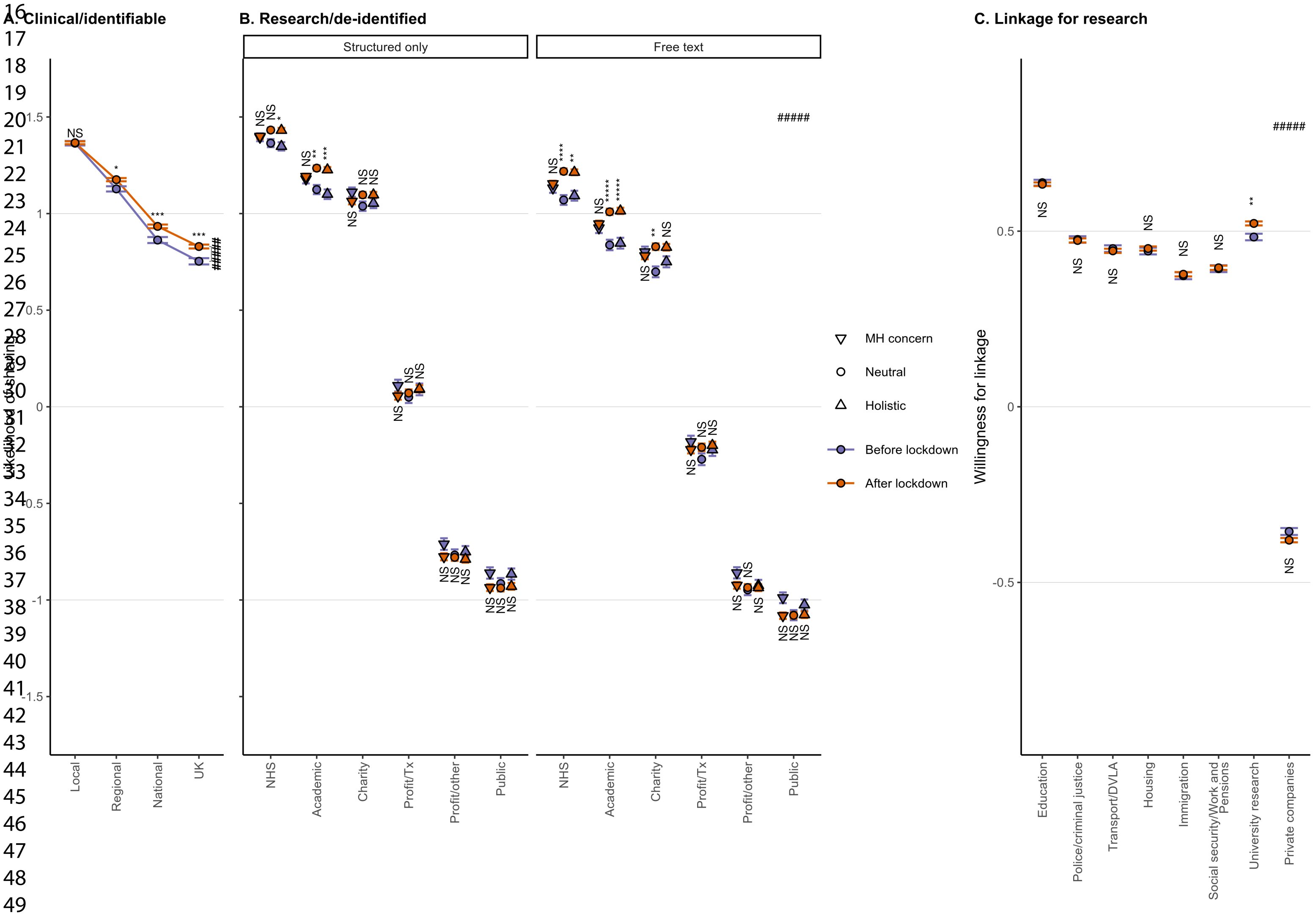
A. Clinical/identifiable
B. Research/de-identified
C. Linkage for research
Structured only
Free text
Likelihood of sharing
Willingness for linkage
Local
Regional
National
UK
NHS
Academic
Charity
Profit/Tx
Profit/other
Public
Education
Police/criminal justice
Transport/DVLA
Housing
Immigration
Social security/Work and Pensions
University research
Private companies
MH concern
Neutral
Holistic
Before lockdown
After lockdown
#####

Figure 6

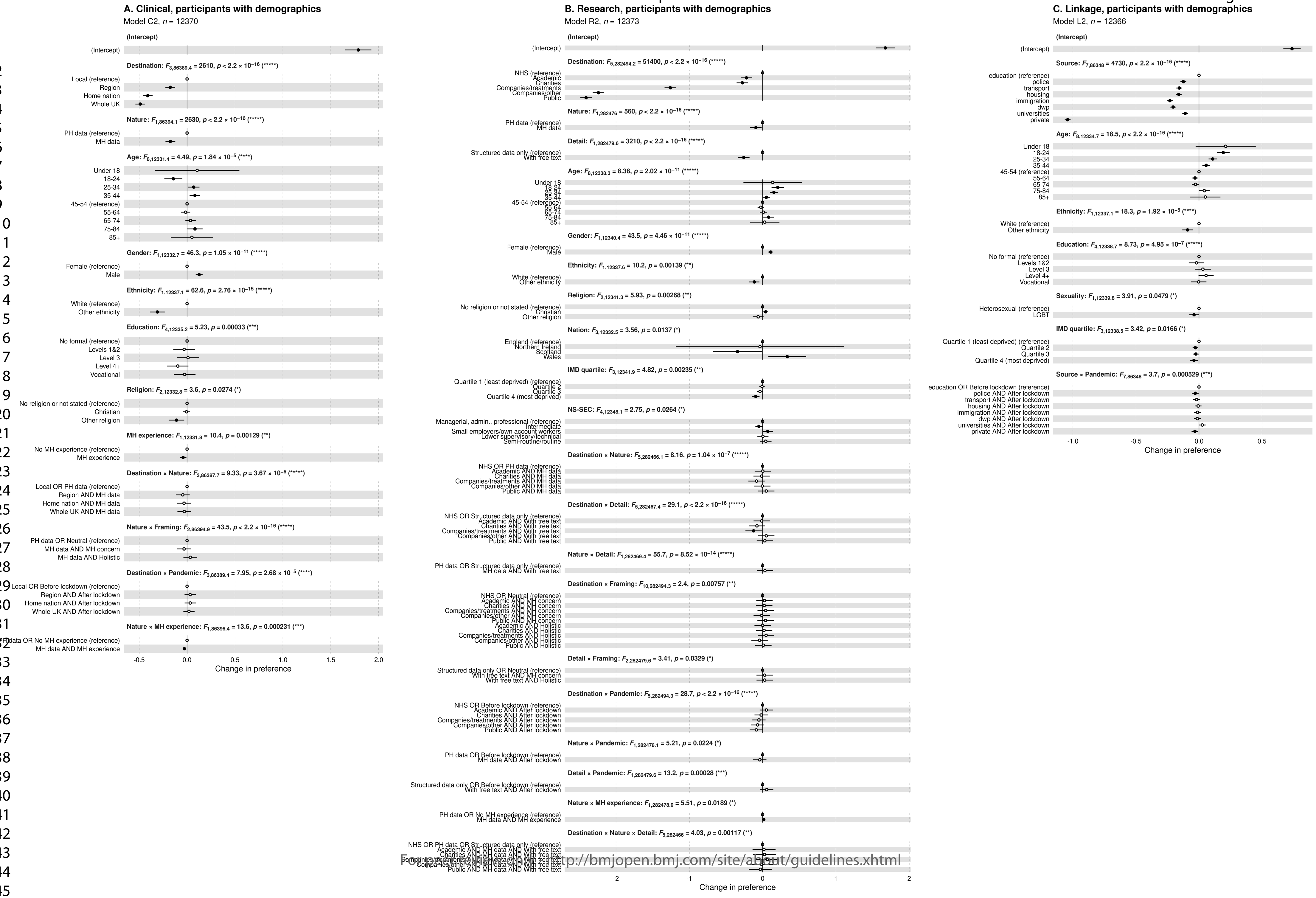
A. Clinical, participants with demographics
Model C2, n = 12370
(Intercept)
Destination: F3,86389.4 = 2610, p < 2.2 × 10−16 (*****)
Local (reference)
Region
Home nation
Whole UK
Nature: F1,86394.1 = 2630, p < 2.2 × 10−16 (*****)
PH data (reference)
MH data
Age: F8,12331.4 = 4.49, p = 1.84 × 10−5 (****)
Under 18
18-24
25-34
35-44
45-54 (reference)
55-64
65-74
75-84
85+
Gender: F1,12332.7 = 46.3, p = 1.05 × 10−11 (*****)
Female (reference)
Male
Ethnicity: F1,12337.1 = 62.6, p = 2.76 × 10−15 (*****)
White (reference)
Other ethnicity
Education: F4,12335.2 = 5.23, p = 0.00033 (***)
No formal (reference)
Levels 1&2
Level 3
Level 4+
Vocational
Religion: F2,12332.8 = 3.6, p = 0.0274 (*)
No religion or not stated (reference)
Christian
Other religion
MH experience: F1,12331.8 = 10.4, p = 0.00129 (**)
No MH experience (reference)
MH experience
Destination × Nature: F3,86387.7 = 9.33, p = 3.67 × 10−6 (*****)
Local OR PH data (reference)
Region AND MH data
Home nation AND MH data
Whole UK AND MH data
Nature × Framing: F2,86394.9 = 43.5, p < 2.2 × 10−16 (*****)
PH data OR Neutral (reference)
MH data AND MH concern
MH data AND Holistic
Destination × Pandemic: F3,86389.4 = 7.95, p = 2.68 × 10−5 (****)
Local OR Before lockdown (reference)
Region AND After lockdown
Home nation AND After lockdown
Whole UK AND After lockdown
Nature × MH experience: F1,86396.4 = 13.6, p = 0.000231 (***)
PH data OR No MH experience (reference)
MH data AND MH experience
-0.5
0.0
0.5
1.0
1.5
2.0
Change in preference
B. Research, participants with demographics
Model R2, n = 12373
(Intercept)
Destination: F5,282494.2 = 51400, p < 2.2 × 10−16 (*****)
NHS (reference)
Academic
Charities
Companies/treatments
Companies/other
Public
Nature: F1,282476 = 560, p < 2.2 × 10−16 (*****)
PH data (reference)
MH data
Detail: F1,282479.6 = 3210, p < 2.2 × 10−16 (*****)
Structured data only (reference)
With free text
Age: F8,12338.3 = 8.38, p = 2.02 × 10−11 (*****)
Under 18
18-24
25-34
35-44
45-54 (reference)
55-64
65-74
75-84
85+
Gender: F1,12340.4 = 43.5, p = 4.46 × 10−11 (*****)
Female (reference)
Male
Ethnicity: F1,12337.6 = 10.2, p = 0.00139 (**)
White (reference)
Other ethnicity
Religion: F2,12341.3 = 5.93, p = 0.00268 (**)
No religion or not stated (reference)
Christian
Other religion
Nation: F3,12332.5 = 3.56, p = 0.0137 (*)
England (reference)
Northern Ireland
Scotland
Wales
IMD quartile: F3,12341.9 = 4.82, p = 0.00235 (**)
Quartile 1 (least deprived) (reference)
Quartile 2
Quartile 3
Quartile 4 (most deprived)
NS-SEC: F4,12348.1 = 2.75, p = 0.0264 (*)
Managerial, admin., professional (reference)
Intermediate
Small employers/own account workers
Lower supervisory/technical
Semi-routine/routine
Destination × Nature: F5,282466.1 = 8.16, p = 1.04 × 10−7 (*****)
NHS OR PH data (reference)
Academic AND MH data
Charities AND MH data
Companies/treatments AND MH data
Companies/other AND MH data
Public AND MH data
Destination × Detail: F5,282467.4 = 29.1, p < 2.2 × 10−16 (*****)
NHS OR Structured data only (reference)
Academic AND With free text
Charities AND With free text
Companies/treatments AND With free text
Companies/other AND With free text
Public AND With free text
Nature × Detail: F1,282469.4 = 55.7, p = 8.52 × 10−14 (*****)
PH data OR Structured data only (reference)
MH data AND With free text
Destination × Framing: F10,282494.3 = 2.4, p = 0.00757 (**)
NHS OR Neutral (reference)
Academic AND MH concern
Charities AND MH concern
Companies/treatments AND MH concern
Companies/other AND MH concern
Public AND MH concern
Academic AND Holistic
Charities AND Holistic
Companies/treatments AND Holistic
Companies/other AND Holistic
Public AND Holistic
Detail × Framing: F2,282479.6 = 3.41, p = 0.0329 (*)
Structured data only OR Neutral (reference)
With free text AND MH concern
With free text AND Holistic
Destination × Pandemic: F5,282494.3 = 28.7, p < 2.2 × 10−16 (*****)
NHS OR Before lockdown (reference)
Academic AND After lockdown
Charities AND After lockdown
Companies/treatments AND After lockdown
Companies/other AND After lockdown
Public AND After lockdown
Nature × Pandemic: F1,282478.1 = 5.21, p = 0.0224 (*)
PH data OR Before lockdown (reference)
MH data AND After lockdown
Detail × Pandemic: F1,282479.6 = 13.2, p = 0.00028 (***)
Structured data only OR Before lockdown (reference)
With free text AND After lockdown
Nature × MH experience: F1,282478.9 = 5.51, p = 0.0189 (*)
PH data OR No MH experience (reference)
MH data AND MH experience
Destination × Nature × Detail: F5,282466 = 4.03, p = 0.00117 (**)
NHS OR PH data OR Structured data only (reference)
Academic AND MH data AND With free text
Charities AND MH data AND With free text
Companies/treatments AND MH data AND With free text
Companies/other AND MH data AND With free text
Public AND MH data AND With free text
-2
-1
0
1
2
Change in preference
C. Linkage, participants with demographics
Model L2, n = 12366
(Intercept)
Source: F7,86348 = 4730, p < 2.2 × 10−16 (*****)
education (reference)
police
transport
housing
immigration
dwp
universities
private
Age: F8,12334.7 = 18.5, p < 2.2 × 10−16 (*****)
Under 18
18-24
25-34
35-44
45-54 (reference)
55-64
65-74
75-84
85+
Ethnicity: F1,12337.1 = 18.3, p = 1.92 × 10−5 (****)
White (reference)
Other ethnicity
Education: F4,12338.7 = 8.73, p = 4.95 × 10−7 (*****)
No formal (reference)
Levels 1&2
Level 3
Level 4+
Vocational
Sexuality: F1,12339.8 = 3.91, p = 0.0479 (*)
Heterosexual (reference)
LGBT
IMD quartile: F3,12338.5 = 3.42, p = 0.0166 (*)
Quartile 1 (least deprived) (reference)
Quartile 2
Quartile 3
Quartile 4 (most deprived)
Source × Pandemic: F7,86348 = 3.7, p = 0.000529 (***)
education OR Before lockdown (reference)
police AND After lockdown
transport AND After lockdown
housing AND After lockdown
immigration AND After lockdown
dwp AND After lockdown
universities AND After lockdown
private AND After lockdown
-1.0
-0.5
0.0
0.5
Change in preference

Figure 7

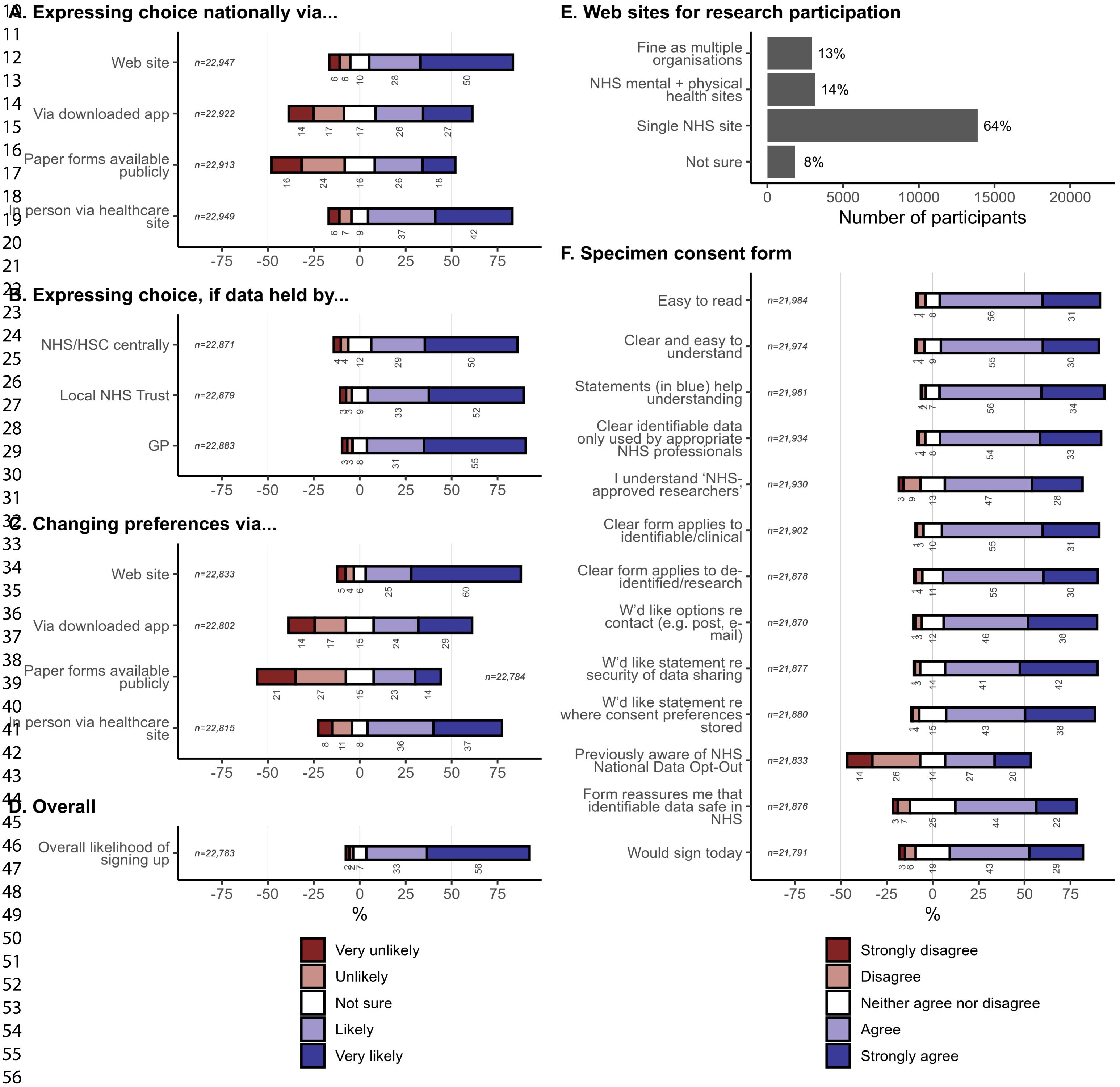
A. Expressing choice nationally via...
Web site
Via downloaded app
Paper forms available publicly
In person via healthcare site
B. Expressing choice, if data held by...
NHS/HSC centrally
Local NHS Trust
GP
C. Changing preferences via...
D. Overall
Overall likelihood of signing up
E. Web sites for research participation
Fine as multiple organisations
NHS mental + physical health sites
Single NHS site
Not sure
Number of participants
F. Specimen consent form
Easy to read
Clear and easy to understand
Statements (in blue) help understanding
Clear identifiable data only used by appropriate NHS professionals
I understand 'NHS-approved researchers'
Clear form applies to identifiable/clinical
Clear form applies to de-identified/research
W'd like options re contact (e.g. post, e-mail)
W'd like statement re security of data sharing
W'd like statement re where consent preferences stored
Previously aware of NHS National Data Opt-Out
Form reassures me that identifiable data safe in NHS
Would sign today
%
Very unlikely
Unlikely
Not sure
Likely
Very likely
Strongly disagree
Disagree
Neither agree nor disagree
Agree
Strongly agree

SUPPLEMENTARY MATERIALS

Public opinion on sharing data from UK health services for clinical and research purposes without explicit consent (Jones et al.)

Contents

S1. Supplementary Methods
- *S1.1. Survey design*
- *S1.2. Analysis of geography including Index of Multiple Deprivation*
- *S1.3. Sensitivity analysis via survey weighting*
- *S1.4. Effect size plots*
- *S1.5. Free-text values and comments*

S2. Supplementary Results
- *S2.1. Recruitment and completion*
- *S2.2. Demographics*
- *S2.3. Experience*
- *S2.4. Raking*
- *S2.5. Weighted analysis of sharing preferences*
- *S2.6. Weighted views on linkage to non-health data for research*
- *S2.7. Effect sizes*
- *S2.8. Weighted views on a national data sharing consent system*
- *S2.9. Free-text comments on the suggested consent form*
- *S2.10. Other free-text comments*

S3. Supplementary References
S4. Supplementary Tables
S5. List of appendices
S6. Supplementary Figure Legends
S7. Supplementary Figures
Appendix A: Underlying REDCap survey design
Appendix B: Survey
Appendix C: STROBE statement for cross-sectional studies

For peer review only

S1. SUPPLEMENTARY METHODS

S1.1. Survey design

The survey sequence is shown in full in the **Appendices.** In outline, it was as follows:

- Information, consent, and a question how the participant heard about the survey. (Participants were asked to indicate the health organization, if any, through which they had heard about the study and recruitment was attributed to those sites.)
- An explanation of "NHS" (used as shorthand for NHS/HSC), "health data", "identifiable health data", and "clinical care".
- Questions about **current and desired sharing of identifiable health data for clinical care purposes.**
  - A multiple-choice question on the respondent's understanding of how NHS clinical care providers *currently* share their identifiable data for clinical care purposes, without asking each time.
  - A statement that most NHS providers are separate and do not currently share identifiable data without asking, followed by a multiple-choice question as to if/how the NHS *should* share the respondent's identifiable data for clinical care purposes, without asking each time (with all stems ending "… without asking me first").
- Questions about **personal experience** of health conditions.
  - Whether the respondent had or hadn't experienced a mental health (MH) condition at some point (or a prefer-not-to-say option). If answered positively, categorical questions about what sort of condition that was, and whether it was recent or >5 years ago, and what sort of support had been obtained (e.g. from NHS or other sources).
  - Whether the respondent had ever used physical health services, and if so, which categories.
- Fictional examples of **identifiable health data** were presented. These included identifiers, diagnoses, and notes.
- A **framing statement** was presented, randomized to be one of the following:
  - *Neutral:* "We would like to find out your perspective on using information about your mental health and your physical health."
  - *MH concern:* "Previous surveys have found that people have more concerns about the use of their identifiable health data relating to their mental health than other aspects of their physical health care." This statement was based on previous findings.[1]
  - *Holistic:* "Mental and physical illnesses overlap, so holistic health care is important. Mental health problems have physical consequences, and physical illnesses have important consequences for mental health." This statement is also true (e.g. [2,3]).
- Likert-style questions about how likely the respondent would be to share their *identifiable physical health* data, for *clinical care* purposes, with a range of NHS "destinations", without being asked each time.
- Likert-style questions about how likely the respondent would be to share their *identifiable mental health* data, for *clinical care* purposes, with a range of NHS destinations, without being asked each time.
- Fictional examples of **de-identified structured health data** were presented (the same examples as before, now de-identified, and without any free text). These included alphanumeric research identifiers, "blurred" demographics (age, sex, geographical region), and diagnoses.
- Likert-style questions about how likely the respondent would be to share their *de-identified (structured) physical health* data with a range of research destinations, without giving consent each time.
- Likert-style questions about how likely the respondent would be to share their *de-identified (structured) mental health* data with a range of research destinations, without giving consent each time.
- Fictional examples of **de-identified free-text health data** were presented. These were the same de-identified data as before, but now with de-identified versions of the free-text notes. Explicit commentary was given that there was more information present, and a slightly higher risk of inadvertent identification. We gave the example of a hypothetical newspaper report that might enable someone to re-identify a patient.
- Likert-style questions about how likely the respondent would be to share their *de-identified (free text) physical health* data with a range of research destinations, without giving consent each time.
- Likert-style questions about how likely the respondent would be to share their *de-identified (free text) mental health* data with a range of research destinations, without giving consent each time.

For peer review only - http://bmjopen.bmj.com/site/about/guidelines.xhtml

- An explicit change of topic was noted.
- We asked Likert-style questions about how likely people would be to sign up to a **single system for controlling how one's NHS data is used** (clinically and for research), for a variety of types of system (e.g. online, in person), who should look after such consent-related data, what the respondent's preferred method would for changing their preferences, and overall how likely they would be to sign up to such a system.
- We showed a **specimen consent form** for such a system (see below for full details).
  - First, the specimen form asked about sharing data for direct health care purposes.
    - It defined "confidential patient information" [4], and set the context in terms of health care being provided directly to the respondent.
    - It offered a yes/no decision: "I agree that all NHS care providers and professionals may share my confidential patient information with each other for the purposes of my treatment and care."
  - Second, the specimen form asked about the use of de-identified data for research.
    - It provided brief information about the NHS's promises to use anonymised data for research (e.g. [5,6]). It said that research was conducted by the NHS and by NHS-approved researchers such as universities. It referred to strict security controls and NHS oversight.
    - It offered a yes/no decision: "I agree that all NHS care providers may share my confidential patient information with each other and de-identify it for the purpose of research."
    - We phrased the question in this way because it is already permitted, given NHS research ethics approvals, for NHS bodies to de-identify health data for research.[7,8] However, cross-site linkage within the NHS is more challenging; if linkage is conducted with direct identifiers such as NHS numbers, this is work involving confidential patient information for research, which requires either explicit consent or approvals (in England) under section 251 of the NHS Act 2006 [4] (as amended) and the Health Service (Control of Patient Information) Regulations 2002.[9] That is the case even if the data are de-identified subsequently and researchers never see identifiable information.
    - We said also that saying no would not prevent all uses of one's confidential information for research. At present, in England, the NHS National Data Opt-Out is the mechanism to opt out from uses of one's confidential personal information where that use is governed by section 251 of the NHS Act.[10,11]
  - Third, the specimen form asked about taking part in research.
    - It discussed briefly research involving direct participation, stating that the NHS promised to inform people of research studies for which they may be eligible,[5,6] and saying that there is never a commitment to take part.
    - It offered a yes/no decision: "I agree that NHS-approved researchers may learn my identity and contact me directly about research studies for which I may be eligible."
  - Having showed the respondent the specimen consent form, we asked Likert-style questions about its clarity, how the respondent understood its meaning, whether some additional aspects should be added, whether the respondent was previously aware of the NHS National Data Opt-Out, and whether the respondent would choose to sign such a form if it were available to them today.
- We asked, via a multiple-choice question, whether **sign-up portals for research** should be multiple (as they are now), single (across the NHS), or split by mental/physical health research.
- We asked about the respondents' preference for **linkage of their health data to other data sources** for research. We set out a basic method commonly used for identifiable linkage, in which special permissions are sought, trusted third-party linkage is conducted (using identifiable information), followed by de-identification for research. We asked whether the respondent would be happy (yes / not sure / no / prefer not to say) for their health data to be linked to a number of "state" sources (education, police/criminal justice, transport, housing, immigration, social security), giving simple examples of the potential research reasons for each linkage, plus universities (e.g. if the respondent had volunteered for research studies) and data held by private companies.
- Finally, we asked optional **demographic** questions: gender; age range; ethnicity; sexuality; religion; employment status, with conditional questions sufficient to determine the UK Office for National Statistics (ONS) National Statistics Socio-Economic Classification (NS-SEC)[12] via the "self-coded" method[13] if the respondent answered in full; UK nation of residence; and (if the respondent was willing) their postcode, to calculate a "blurred" geographical version as described below. We used ONS demographic categories where available. We offered the option to leave an e-mail address to receive a summary of results when available.
- The survey closed by thanking the participant (not shown in the Appendices).

The REDCap design for the survey is shown in **Appendix A,** and the resulting survey (including the specimen consent form) is shown in **Appendix B.**

For peer review only - http://bmjopen.bmj.com/site/about/guidelines.xhtml

S1.2. Analysis of geography including Index of Multiple Deprivation

Where participants agreed to leave a postcode, this was converted to a larger ONS geographical area, so that individuals could not inadvertently be identified. In turn, the larger geographical area was converted to an Index of Multiple Deprivation (IMD), a composite rank measure covering income, employment, education, health, crime, barriers to housing and services, and living environment.[14] A lower raw IMD indicates greater deprivation. For England and Wales, postcodes were converted to a Lower Layer Super Output Area; these have a minimum population of 1,000 and a mean population of 1,500.[15] For Scotland, the Data Zone was used; for Northern Ireland (NI), the Super Output Area (SOA). Here, we use the term "geographical area" (GA) for LSOA (England and Wales), DZ (Scotland), or SOA (NI). We used GAs from the 2011 Census.

GAs were converted to a UK-wide IMD score,[16,17] using the scale relative to England, in which high numbers represent greater deprivation. As this UK-wide data set uses 2001 DZs for Scotland, we mapped these to 2011 DZs.[18] Where multiple 2001 DZs mapped to one 2011 DZ, we took the mean of their UK-wide IMD scores, weighted by their population contribution to the 2011 DZ.[18]

Since GAs do not have equal populations, we corrected for population when calculating centile of deprivation. We used mid-2019 ONS estimates of GA population.[19–21] We calculated deprivation centile (100% meaning most deprived) by calculating, for each GA, "what percentage of the total UK population (66,796,806) live in a GA with a UK IMD score that is equal to or lower than that of this GA (i.e. in areas that are equally or less deprived than this GA)?". We calculated quartiles similarly.

To show the distribution of deprivation, we plotted the distribution of deprivation centile using a Gaussian kernel density estimate.

For map representations, the Apr 2019 ONS local authority district boundaries[22] (which exclude the Channel Islands) were used with the Nov 2019 ONS Postcode Directory.[23]

S1.3. Sensitivity analysis via survey weighting

As a sensitivity analysis to correct for unrepresentative demographic sampling during general linear modelling, we used raking,[24] specifically the American National Election Study (ANES) weighting algorithm[25] via the *anesrake* package.[26,27] We defined dimensions (classification variables) and categories (within dimensions) as shown in **Supplementary Table 1,** collapsing across some low-frequency categories in the survey. We included all such variables in the raking, and used the default weight cap of 5.

The algorithm does not alter weights for categories for which population expected proportions are not known (e.g. those identifying as neither male nor female, for which Census data are not available, or those answering "prefer not to say" for a given question). Such respondents are therefore assigned a weight of 1 in that category, representing them fairly in the absence of any other information with which to weight them.

Population values for some questions were not available; for example, sexual orientation was not part of the UK 2011 Census.[28]

We weighted all respondents once only, consistently (ignoring the potential for discrepant drop-out rates across the survey).

We used weighted versions of the statistical models (see **Methods**) that did not include demographic predictors, labelling these with "W" (e.g. model CW1 was a weighted version of model C1).

S1.4. Effect size plots

We show effect sizes with 95% confidence intervals from selected statistical models. We use the conventional language of "factor" (discrete predictor), "levels" (possible values of a factor), and "term" (individual predictive term in a GLM, such as "destination" or "destination × nature"). Effect sizes are shown as uncorrected pairwise comparison to a reference category within each term. For age, we used the central category (age range 45–54) as the reference category, because there were very few participants in the "under 18" category. For brevity, we restrict plots to those model terms with a significant *F* test. We show degrees of freedom to 1 decimal place and statistics to 3 significant figures.

F tests, as for the main results, were taken from *anova(model)* via *lmerTest::anova.lmerModLmerTest*, giving type III sums of squares via the Satterthwaite method for degrees of freedom.[29] Type III sums of squares test the effect of each term "over and above" others.[30] Pairwise comparisons were taken from *summary(model)* via *lmerTest::summary.lmerModLmerTest*, also using Satterthwaite's method.

We note the important difference between these pairwise contrasts, helpful for basic visual display, and the omnibus *F* test for the term.[30] In particular, we note firstly that for a single factor with three levels (A, B, and C with A as the reference level), the omnibus *F* test tests the null hypothesis A = B = C; if this null hypothesis is rejected, it remains possible that neither the pairwise hypothesis A = B nor the hypothesis A = C is rejected (for example, if the ordering is

For peer review only

B < A < C). Thus, failure to observe differences in the specific pairwise contrasts does not imply that there are no differences in the data. Secondly, a similar effect can be observed with interactions: pairwise contrasts within interaction components may sometimes be suboptimal compared to subgroup simple effects analyses.[30] Thus, we used simple effects analysis for detailed follow-up of significant interactions where appropriate.

S1.5. Free-text values and comments

Some respondents wrote in responses for "other" categories. Some provided free-text comments where invited to do so. A few e-mailed the study team separately. We provide narrative summaries of these answers, paraphrasing to avoid direct quotations and reporting "$n < 10$" where appropriate to mask small numbers.

For free-text comments on the proposed draft national consent form, we conducted a thematic analysis and report tallies by theme.

S2. SUPPLEMENTARY RESULTS

S2.1. Recruitment and completion

Supplementary Figure 1 shows recruitment sources, recruitment rates, and survey participation by stage.

S2.2. Demographics

A demographic breakdown is shown in **Supplementary Figure 2**.

Free-text values provided in response to "other, please specify" categories included:

- *Mental health conditions* (n = 274): many unique responses, with non-unique responses distinct from the options offered including: ADHD [attention-deficit/hyperactivity disorder], Asperger's syndrome/autism/autistic spectrum disorder, bereavement, body dysmorphic disorder, dissociative disorders, gambling disorders, insomnia, low mood, memory problems, menopause-associated symptoms, non-epileptic attack disorder, overdose, post-traumatic stress disorder, postnatal depression, suicidality, and stress.
- *Mental health services used* (n = 254): many unique responses, with non-unique responses including: counselling services, military services, specific named charities (including Mind and Samaritans), university mental health support services, and well-being services.
- *Physical health services used* (n = 258): many unique responses, with non-unique responses including: chiropractors, complementary therapists, dentistry, maternity services, NHS web sites, osteopaths, pharmacists, physiotherapists, and well-being services.
- *Gender* (n = 29): non-unique responses included: agender, gender fluid, non-binary, trans/transgender.
- *Ethnicity* (n = 150): a large number of responses more detailed than the ONS options on offer, with some critical of the premise.
- *Sexuality* (n = 65): a number of unique options and some critical of the premise or phrasing, plus some with multiple responses (e.g. asexual, panromantic, pansexual).
- *Religion* (n = 219): a range, some more closely specifying options offered (e.g. agnostic, atheist, Baptist, Catholic, Church of England, humanist, Jehovah's Witness, Methodist, Orthodox, Quaker) and some for beliefs not listed (e.g. Baha'i, druid, Jedi, pagan, pantheist, spiritualist, Wiccan), including a number of unique responses.
- *Geography* (England n = 43, Scotland $n < 10$, Wales n = 20, Northern Ireland $n < 10$): a range of geographical divisions not matching the categories offered.

S2.3. Experience

Participants' experience of mental health conditions/service and physical health services are shown in **Supplementary Figure 3**.

S2.4. Raking

The raking algorithm converged stably, and substantially improved the match to population marginal proportions, though was unable to make the resulting weights match the population marginals completely within the constraints specified. We show observed and raked proportions in **Supplementary Table 1**.

For peer review only - http://bmjopen.bmj.com/site/about/guidelines.xhtml

S2.5. Weighted analysis of sharing preferences

Weighted analyses (**Supplementary Figure 4**) were quantitatively similar to the primary results (compare **Figure 3**), and statistical analyses showed the same key patterns, although with somewhat weaker framing effects.

For clinical purposes (model CW1, n = 24497), there were effects including destination ($F_{3,181628}$ = 6310, VLP), nature ($F_{1,181756}$ = 4860, VLP), nature × framing ($F_{2,181756}$ = 21·3, p = 5·41 × 10^{-10}), destination × nature ($F_{3,181624}$ = 5·84, p = 0·000557), and destination × framing ($F_{6,181628}$ = 4·38, p = 0.000199). In sub-analyses of the framing effects (analysis of MH and PH data separately using model CW1B), there was no effect of framing for PH data (F < 1, NS) but this time the framing effect for MH data was not itself significant (framing, $F_{2,13607}$ = 1·74, NS; destination × framing, $F_{6,84700}$ = 1·91, p = 0.0748). As for the main analysis (model C1), the framing effects were slightly smaller for geographically broader (e.g. UK-wide) sharing, driving a destination × framing interaction via PH data.

For research purposes (model RW1, n = 23869), main effects included destination ($F_{5,547684}$ = 74700, VLP) nature ($F_{1,547914}$ = 1613, VLP), detail ($F_{1,548574}$ = 5270, VLP), and framing ($F_{2,13472}$ = 3·09, p = 0·045). Interactions included destination × nature × detail ($F_{5,547655}$ = 8·64, p = 8·64 × 10^{-8}) and nature × framing × pandemic ($F_{2,547914}$ = 5·25, p = 0·00536). In sub-analyses of the framing effects (analysis of MH and PH data separately using model RW1B), framing main effects were not independently significant, though there were destination × framing interactions as before.

S2.6. Weighted views on *linkage to non-health data for research*

Supplementary Figure 5 shows weighted views on linkage to non-health data for research (compare **Figure 4**).

S2.7. Effect sizes

Supplementary Figure 6 shows effect sizes for models C1, R1, and L1. These models include data from all participants who answered the relevant questions, including those who did not supply full demographic information (compare **Figure 6**).

S2.8. Weighted views on a national data sharing consent system

Supplementary Figure 7 shows weighted views on a national data consent system (compare **Figure 7**).

S2.9. Free-text comments on the suggested consent form

Themes of free-text comments in relation to the proposed consent form (n = 3112) included:

- Form design:
  - *Too long/complex* (n = 732). The form was too long and/or complex, or the text (or aspects of the text) should be clearer or simpler, including statements that the reader found it OK but felt that others (not specified in detail) would not.
  - *Visual style* (n = 395). Regarding visual clarity and style (e.g. typography, layout, colours, and the spelling of 'organization' versus 'organisation').[31]
  - *Clear/good* (n = 159). The form was satisfactory, good, clear, and/or concise.
  - *Extra information or accessible versions* (n = 271). There should be accessible forms, easy-read versions, alternative language versions, or accompanying explanations (e.g. leaflet, illustrations, video, explanations of the benefits and risks of data sharing).
  - *Too brief* (n = 18). It was too brief, and/or required more detail or explanation (in general).
  - *Biased* (n = 16). It was biased, coercive, or misleading in its questions.
- Method of completion:
  - *Digital* (n = 69). A digital or online version would be desirable or preferable (or in some cases that a paper copy would be desirable or necessary as well).
  - *Non-digital* (n = 26). A paper copy would be desirable or necessary, or that not everyone could use internet-based methods.
  - *One-to-one support* (n = 90). One-to-one support (e.g. face-to-face, e-mail, telephone) would be desirable or necessary (from a clinician or other unbiased person, including support for those who may lack capacity to decide, and for children).
  - *Copies* (n = 27). People should have copies of what they agreed to.

For peer review only

- ◦ *Changing preferences* (n = 96). It should be easy to revisit and update one's preferences and/or contact details (and/or clarity on what would happen to data previously shared under such circumstances).
- ◦ *Enough time* (n = 49). It is important people have time to complete the form and do not feel pressured to do so, and/or are not asked during times of personal medical crisis.

- Legal aspects:
  - ◦ *Legal* (n = 58). That the Data Protection Act and/or European Union (EU) General Data Protection Regulation (GDPR), Access to Health Records Act, Information Commissioner's Office, NHS privacy documents (e.g. privacy impact assessments, Caldicott Guardian framework), or mental capacity frameworks (e.g. Mental Capacity Act, Lasting Power of Attorney) should be referenced explicitly, or views that the proposed system/form would be (or that existing NHS information systems are) incompatible with one of these.
- Managing data related to consent:
  - ◦ *Data for the consent process* (n = 83). Relating to self-identification when completing the form: that asking for an NHS number is problematic as people may not know this, that some identifiers (e.g. full name, address, e-mail address) are unnecessary or sometimes unnecessary, or that some (e.g. telephone number, previous names and addresses) should be added, or concerns about the management of identity information used for the consent form.
- Management of healthcare data:
  - ◦ *De-identification* (n = 164). That aspects of de-identification/anonymisation/pseudonymisation needed to be clearer, including that asking for identity data whilst recording preferences about de-identified data use was confusing, or that any form of pseudonymisation (rather than full anonymisation) is undesirable, or that examples should be given of identifiable versus de-identified data.
  - ◦ *Data security* (n = 594). That the security and privacy of healthcare data is of paramount importance, including that more detail should be provided about data security/sharing or privacy controls, or that the respondent did not trust the NHS or the UK to manage data security properly.
  - ◦ *Centralization* (n = 30). Greater centralisation of data increases concern and risks.
  - ◦ *Sharing desirable* (n = 290). Data sharing is desirable in general, for clinical or research purposes or not specified.
  - ◦ *Sharing undesirable* (n = 74). Data sharing is undesirable in general, e.g. without case-by-case specific consent (± except in emergencies).
  - ◦ *Opt-outs* (n = 134). That opt-outs should be more prominent, the default, that the system should be linked with the NHS National Data Opt-Out (directly or by implication), or that the relationship to NHS Act Section 251 approval was unclear.
- Categories of healthcare data:
  - ◦ *Distinguish mental/physical health* (n = 45). Mental health and physical health data should be distinguished.
  - ◦ *Combine mental/physical health* (n < 10). Mental health and physical health data should not be distinguished.
  - ◦ *Distinguish health data in other ways* (n = 11). Further subtypes of health data (e.g. sexual health, drug/alcohol use) should be distinguished.
  - ◦ *Distinguish free text from structured data* (n = 23). Free/narrative text should be distinguished from structured data.
  - ◦ *Distinguish data by age* (n < 10). Data should be distinguished by its age (historical versus recent).
- Categories of use:
  - ◦ *Clinical versus research* (n = 44). Clinical and research uses of data should be more clearly distinguished (e.g. not included on the same form).
  - ◦ *Transparency of use* (n = 34). That people should be informed about, or be able to inspect, or choose regarding, each individual use of their data, and/or the results of research involving their data.
  - ◦ *Profit-making* (n = 198). That profit-making use should not happen, or people should be paid directly for providing data used for profit, or that the NHS should ensure that it profits from such data use.
- Data or use/users, not distinguished:
  - ◦ *More detail* (n = 74). More detailed options should be available (in general).
- End users:

For peer review only

- *Specify clinical users more* (n = 147). Permissions to share clinical data should not relate to the whole NHS, but be more detailed (e.g. local, regional, or regarding specific staff groups, or regarding private healthcare providers, or the relationship to social rather than health care).
- *Consent not needed* (n < 10). That consent (or at least explicit consent) is not required for aspects of data sharing, or should not be required.
- *Specify researchers more* (n = 268). That "NHS-approved researchers" is too vague, and that more detail about who such researchers are, or more detailed options to select from (up to and including case-by-case approval by the patient concerned), should be provided.
- *Third-party/private users* (n = 417). That healthcare data should not be available to private or third-party companies (or should require specific permission), including insurance companies and other profit-making organizations, or that later developments to provide them with data would be of concern.
- *Other state users* (n = 29). That use by other state/Government organizations would be of concern, or more detail would be required in that regard.
- *Overseas users* (n = 52). That use by overseas users (e.g. researchers) would be of concern, e.g. because other countries have different data regulations.

- Contact by researchers:
  - *Contact by researchers* (n = 69). That more detailed options should be available about consent for research, or that being contact directly would be of concern (including about frequency of contact, and preferences that contact is always via clinical teams rather than researchers being given identifiable information).
- Overall objectives:
  - *Good idea* (n = 128). The consent system or project was a good idea (in general).
  - *Bad idea* (n = 16). The consent system or project was a bad idea, or unnecessary.
- Other:
  - We did not tally comments relating to other aspects, such as the accuracy of health records themselves, patient access to health records (or holding the primary version of all their health data themselves), descriptions of an individual's care, details of how the respondent would themselves choose, comments on the relationship to consent for clinical treatment, expressed wishes that the research team be shot, comments that people never read forms anyway, views that an independent body should oversee such a process, or concerns about research bias relating to who would or would not consent to their data being used.

S2.10. Other free-text comments

Themes of the free-text comments sent to the research team separately included:

- That the respondent's preferences regarding mental versus physical health data had been driven by personal experience of information being misused (n < 10).
- That all research use of data should require informed consent, involving participants' knowledge of the nature of the research (n < 10).

For peer review only

S3. SUPPLEMENTARY REFERENCES

1 Aitken M, de St Jorre J, Pagliari C, Jepson R, Cunningham-Burley S. Public responses to the sharing and linkage of health data for research purposes: a systematic review and thematic synthesis of qualitative studies. *BMC Med Ethics* 2016; **17**: 73.

2 Grippo AJ, Johnson AK. Biological mechanisms in the relationship between depression and heart disease. *Neurosci Biobehav Rev* 2002; **26**: 941–62.

3 Royal College of Psychiatrists. CR183: Liaison psychiatry for every acute hospital. Integrated mental and physical healthcare. 2013. https://www.rcpsych.ac.uk/docs/default-source/members/faculties/liaison-psychiatry/cr183liaisonpsych-every-acute-hospital.pdf?sfvrsn=26c57d4_2.

4 UK. National Health Service Act 2006. 2006 https://www.legislation.gov.uk/ukpga/2006/41.

5 NHS England. The NHS Constitution for England. 2013; published online March 26. https://www.gov.uk/government/publications/the-nhs-constitution-for-england.

6 NHS England. The NHS Constitution for England. 2015; published online July 27. https://www.gov.uk/government/publications/the-nhs-constitution-for-england.

7 UK. Data Protection Act 2018. 2018 http://www.legislation.gov.uk/ukpga/2018/12/contents/enacted.

8 European Parliament and Council. Regulation (EU) 2016/679 (General Data Protection Regulation). *Off J Eur Union* 2016; **L119**: 1–88.

9 UK. The Health Service (Control of Patient Information) Regulations 2002. 2002 http://www.legislation.gov.uk/uksi/2002/1438/contents/made.

10 NHS. National Data Opt-out: Factsheet 2 – When it applies. 2018. https://digital.nhs.uk/binaries/content/assets/website-assets/services/national-data-opt-out-programme/guidance-for-health-and-care-staff/nhs-factsheet-2-23052018.pdf (accessed July 26, 2019).

11 NHS Digital. Understanding the national data opt-out. 2019. https://web.archive.org/web/20200804083843/https://digital.nhs.uk/services/national-data-opt-out/understanding-the-national-data-opt-out.

12 Office for National Statistics. Standard Occupational Classification 2010: Volume 3: The National Statistics Socio-economic Classification: (Rebased on the SOC2010) User Manual. 2010. https://www.ons.gov.uk/file?uri=/methodology/classificationsandstandards/standardoccupationalclassificationsoc/soc2010/soc2010volume3thenationalstatisticssocioeconomicclassificationnssecrebasedonsoc2010/soc2010vol31amendedjanuary2013tcm77179133.pdf (accessed Feb 13, 2020).

13 Office for National Statistics. Deriving the NS-SEC: self-coded method. 2012. https://www.ons.gov.uk/methodology/classificationsandstandards/standardoccupationalclassificationsoc/soc2010/soc2010volume3thenationalstatisticssocioeconomicclassificationnssecrebasedonsoc2010#deriving-the-ns-sec-self-coded-method (accessed Aug 23, 2019).

14 UK Ministry of Housing, Communities & Local Government. English indices of deprivation 2015. 2015. https://www.gov.uk/government/statistics/english-indices-of-deprivation-2015 (accessed May 15, 2018).

15 UK Office for National Statistics. Census geography: An overview of the various geographies used in the production of statistics collected via the UK census. 2011. https://www.ons.gov.uk/methodology/geography/ukgeographies/censusgeography (accessed May 15, 2018).

16 Abel GA, Barclay ME, Payne RA. Adjusted indices of multiple deprivation to enable comparisons within and between constituent countries of the UK including an illustration using mortality rates. *BMJ Open* 2016; **6**: e012750.

For peer review only

17 Abel GA, Payne RA, Barclay ME. UK Deprivation Indices. 2016; published online Nov 21. https://dx.doi.org/10.5523/bris.1ef3q32gybk001v77c1ifmty7x (accessed Feb 23, 2020).

18 Scottish Government. Data Zone Matching File (Area and Pop. based 2001 - 2011). 2015. https://www2.gov.scot/Resource/0048/00483037.xlsx.

19 Office for National Statistics. Lower layer Super Output Area population estimates (supporting information). 2019. https://www.ons.gov.uk/peoplepopulationandcommunity/populationandmigration/populationestimates/datasets/lowersuperoutputareamidyearpopulationestimates.

20 National Records of Scotland. Mid-2019 Small Area Population Estimates for 2011 Data Zones. 2020. https://www.nrscotland.gov.uk/statistics-and-data/statistics/statistics-by-theme/population/population-estimates/2011-based-special-area-population-estimates/small-area-population-estimates/mid-2019.

21 Northern Ireland Statistics and Research Agency. 2019 Mid Year Population Estimates for Northern Ireland. 2020. https://www.nisra.gov.uk/publications/2019-mid-year-population-estimates-northern-ireland.

22 Office for National Statistics. Local Authority Districts (April 2019) UK BFE. 2019. https://geoportal.statistics.gov.uk/datasets/b06ce126e1014409ac24c82739b2c130_0.

23 Office for National Statistics. ONS Postcode Directory (November 2019). 2019. https://geoportal.statistics.gov.uk/datasets/ons-postcode-directory-november-2019.

24 Kalton G, Flores-Cervantes I. Weighting methods. *J Off Stat* 2003; **19**: 81–97.

25 DeBell M, Krosnick JA. Computing Weights for American National Election Study Survey Data. Ann Arbor, MI; Palo Alto, CA: American National Election Studies, 2009 https://electionstudies.org/wp-content/uploads/2018/04/nes012427.pdf.

26 Pasek J. ANES weighting algorithm: a description. 2010; published online March 15. https://web.stanford.edu/group/iriss/cgi-bin/anesrake/resources/RakingDescription.pdf.

27 Pasek J. anesrake: ANES Raking Implementation. 2018. https://CRAN.R-project.org/package=anesrake (accessed June 4, 2021).

28 Office for National Statistics. 2011 Census Household Questionnaire. 2011. https://www.ons.gov.uk/file?uri=/census/censustransformationprogramme/consultations/the2021censusinitialviewoncontentforenglandandwales/2011censusquestionnaireenglandh1.pdf.

29 Satterthwaite FE. An approximate distribution of estimates of variance components. *Biometrics* 1946; **2**: 110–4.

30 Cardinal RN, Aitken MRF. ANOVA for the Behavioural Sciences Researcher. Mahwah, N.J: L. Erlbaum, 2006.

31 Oxford University Press. New Shorter Oxford English Dictionary. Oxford, UK: Oxford University Press, 1997.

32 Office for National Statistics. Estimates of the population for the UK, England and Wales, Scotland and Northern Ireland: mid-2019. 2020. https://www.ons.gov.uk/peoplepopulationandcommunity/populationandmigration/populationestimates/datasets/populationestimatesforukenglandandwalesscotlandandnorthernireland.

33 Office for National Statistics. Population estimates for the UK, England and Wales, Scotland and Northern Ireland: mid-2019. 2020; published online June 24. https://www.ons.gov.uk/peoplepopulationandcommunity/populationandmigration/populationestimates/bulletins/annualmidyearpopulationestimates/mid2019estimates (accessed Nov 6, 2020).

34 Office for National Statistics. UK population by ethnicity. 2018; published online Aug 1. https://www.ethnicity-facts-figures.service.gov.uk/uk-population-by-ethnicity (accessed Nov 6, 2020).

35 UK Data Service. Age by Highest level of qualification 2011. 2011. http://dx.doi.org/10.5257/census/aggregate-2011-2 (accessed Jan 19, 2021).

For peer review only

36 Office for National Statistics. Population characteristics research tables [2016 estimates]. 2019; published online Dec 4. https://www.ons.gov.uk/peoplepopulationandcommunity/populationandmigration/populationestimates/datasets/populationcharacteristicsresearchtables (accessed Jan 19, 2021).

For peer review only

S4. SUPPLEMENTARY TABLES

Supplementary Table 1. Demographic dimensions and categories used for weighting, with UK population marginal proportions (*e*), observed marginal proportions amongst respondents (*o*, ignoring those who did not answer or answered via a category not acknowledged by UK population statistics), and marginal proportions after raking (*r*, likewise). Proportions are shown to 3 decimal places; VLP, $p<2{\cdot}2\times10^{-16}$.

Variable (dimension)	Category	UK expected population proportion, *e*	Reference for *e*	Observed proportion (ignoring other/unknown), *o*	Sample versus population proportions	Raked proportion (ignoring other/unknown), *r*
Age	Under 18	0·211	[32,33]	0·005	χ^2_8=8860, VLP	0·024
	18–24	0·085		0·050		0·082
	25–34	0·135		0·139		0·167
	35–44	0·126		0·163		0·159
	45–54	0·136		0·218		0·177
	55–64	0·122		0·228		0·160
	65–74	0·100		0·150		0·133
	75–84	0·060		0·043		0·078
	85+	0·025		0·004		0·019
Gender	Female	0·506	[32]	0·673	χ^2_1=2390, VLP	0·471
	Male	0·494		0·327		0·529
Ethnicity	White	0·860	[34] (data only available for England and Wales)	0·910	χ^2_1=442, VLP	0·861
	Other	0·140		0·090		0·139
Education	No formal	0·232	[35]; data for over-16s only; vocational data absent for Scotland (ignored in calculating proportions)	0·025	χ^2_4=13800, VLP	0·121
	Level 1 and Level 2 (approximating "secondary")	0·292		0·139		0·250
	Level 3 (approximating "A-Level/equivalent")	0·121		0·122		0·146
	Level 4+ (approximating undergraduate, postgraduate, and professional)	0·270		0·589		0·364
	Vocational	0·084		0·126		0·119
Religion	No religion or not stated	0·332	[36] (Table B; data only available for England and Wales)	0·618	χ^2_2=10900, VLP	0·337
	Christian	0·565		0·338		0·563
	Other	0·102		0·043		0·100
Nation	England	0·843	[32] (Channel Islands not included)	0·994	χ^2_3=3660, VLP	0·968
	Northern Ireland	0·028		0·0002		0·001
	Scotland	0·082		0·002		0·010
	Wales	0·047		0·004		0·020
Index of multiple deprivation	First (least deprived) quartile	0·25	By definition	0·334	χ^2_3=1620, VLP	0·247
	Second	0·25		0·317		0·195
	Third	0·25		0·233		0·279
	Fourth (most deprived)	0·25		0·116		0·279

For peer review only

For peer review only - http://bmjopen.bmj.com/site/about/guidelines.xhtml

S5. LIST OF APPENDICES

Appendix A shows a screenshot of the full REDCap design for the survey.

Appendix B shows resulting screenshots of the survey running in a web browser. (Questions labelled 11, 12, and 13 internally, as per **Appendix A**, were the same question, but with differing accompanying framing statements; the "concern" framing is shown here. The last content page is shown twice, once in its starting state and once fully expanded after partial completion with hypothetical data.)

Appendix C is a STROBE statement for cross-sectional studies (page numbers for composite PDF including main manuscript and supplementary materials).

For peer review only

S6. SUPPLEMENTARY FIGURE LEGENDS

Supplementary Figure 1. Recruitment sources, participation over time (shading indicates weekends), and survey completion. The denominator for percentages is the total number of consented participants.

Supplementary Figure 2. Demographics of respondents. The denominator for percentages is the number of people who answered each question. For panels marked †, only participants who provided a postcode are included. ONS local authority map and deprivation centiles exclude the Channel Islands. See **Supplementary Methods** for details of geography and deprivation calculations.

Supplementary Figure 3. Respondents' experience of mental health conditions/services and physical health services. The denominator for percentages is the number of people who answered each question.

Supplementary Figure 4. Opinions about sharing mental and physical health data, weighted according to demographic variables. This is equivalent to **Figure 3** apart from the weighting (see **Supplementary Methods**), with identical conventions. "(W)": weighted.

Supplementary Figure 5. Views on linkage to non-health data for research, weighted according to demographic variables. This is equivalent to **Figure 4** apart from the weighting (see **Supplementary Methods**). "(W)": weighted.

Supplementary Figure 6. Effect sizes for predictors of willingness to **(A)** share data for clinical purposes (compare **Figure 3C**), **(B)** share data for research purposes (compare **Figure 3F,H**), or **(C)** support linkage to non-health data for research (compare **Figure 4**). Analysis via models C1, R1, and L1 respectively. These models include all participants, including those not supplying full demographic details, and therefore do not include the demographic predictors; compare **Figure 6**. Conventions as for **Figure 6**.

Supplementary Figure 7. Views on a national data sharing consent system, weighted according to demographic variables. This is equivalent to **Figure 7** apart from the weighting (see **Supplementary Methods**). "(W)": weighted.

For peer review only

Supplementary Figure 1

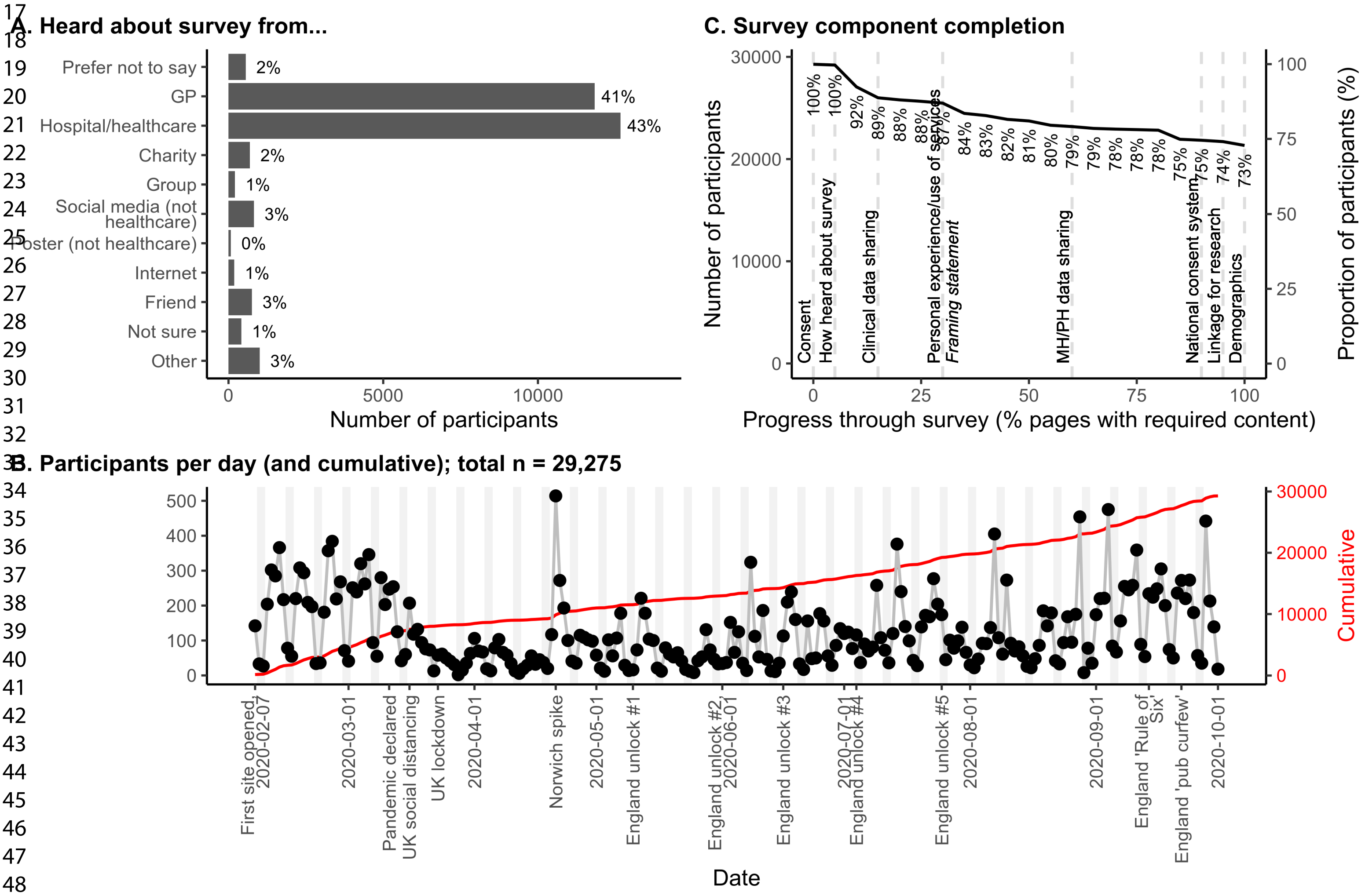

Supplementary Figure 2

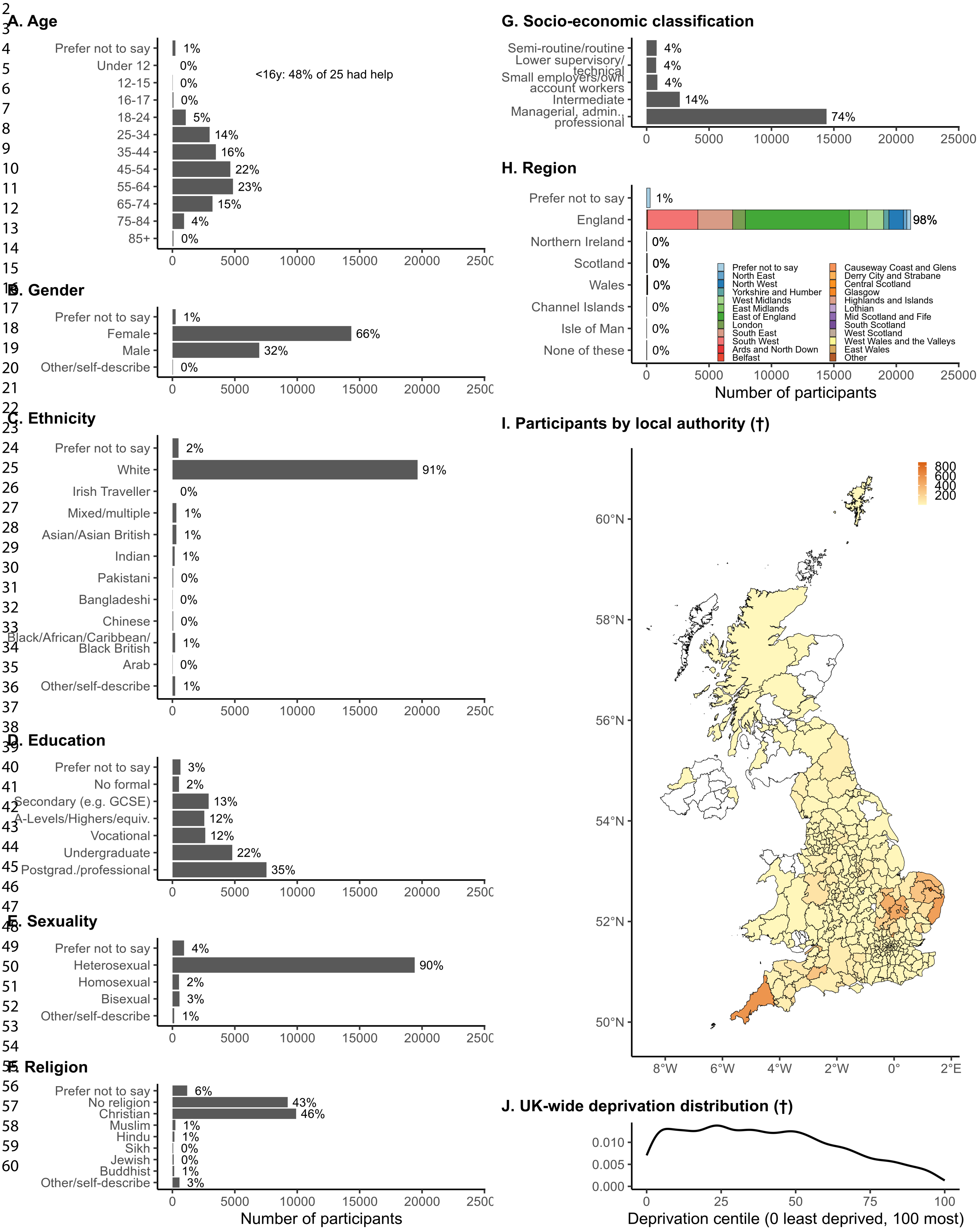

Supplementary Figure 3

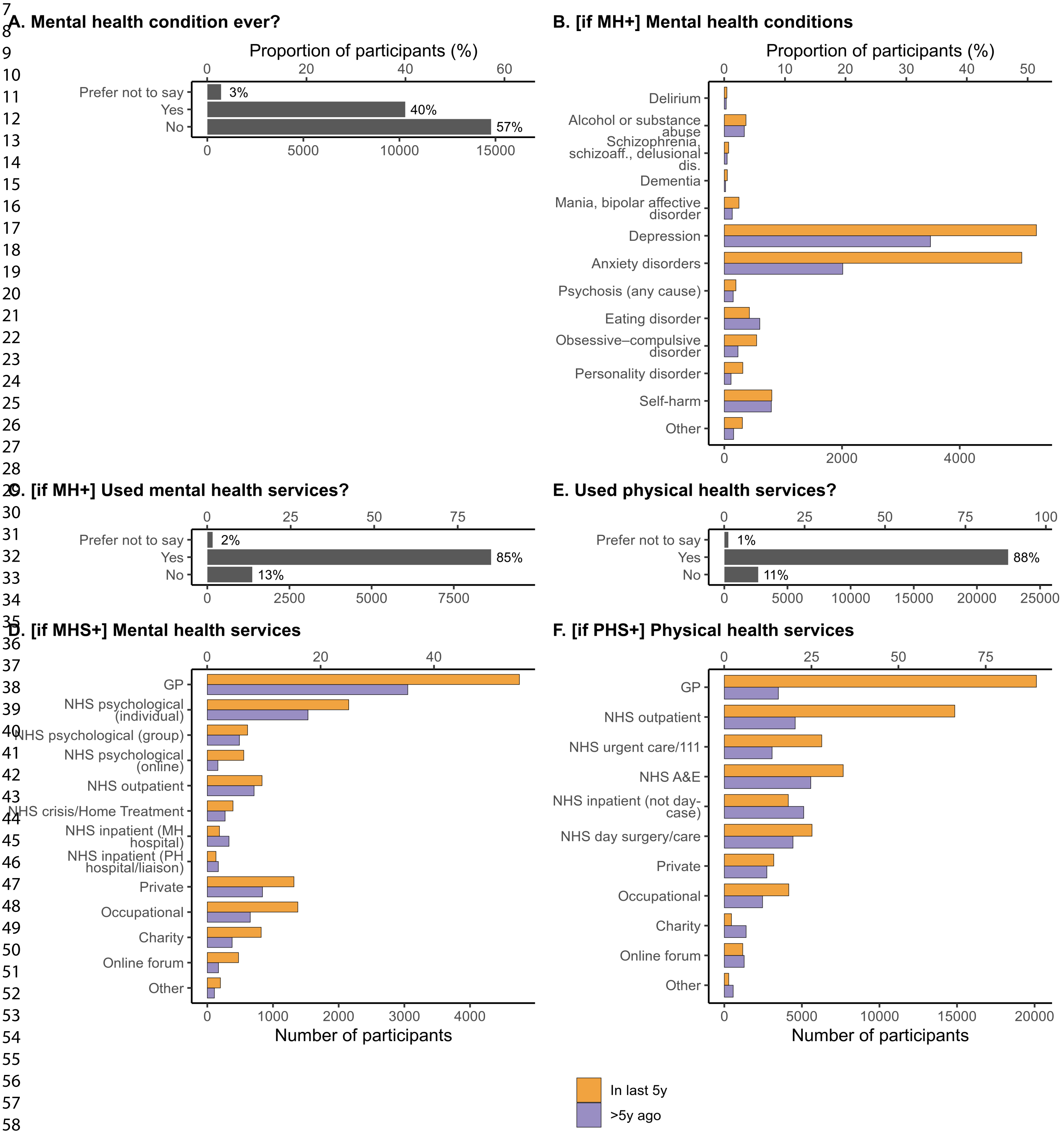

Supplementary Figure 4

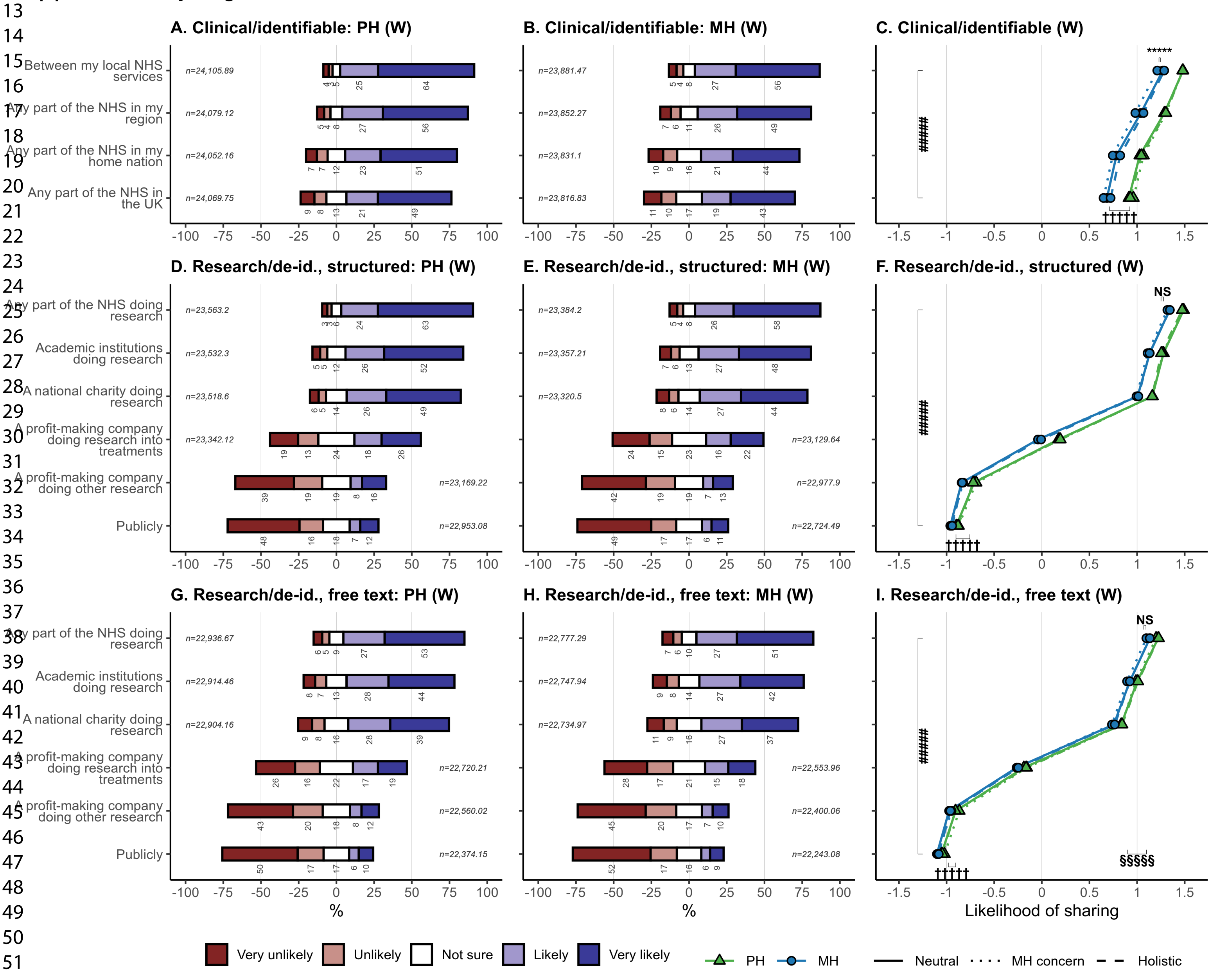

Supplementary Figure 5

De-identified linkage to non-health data (W)

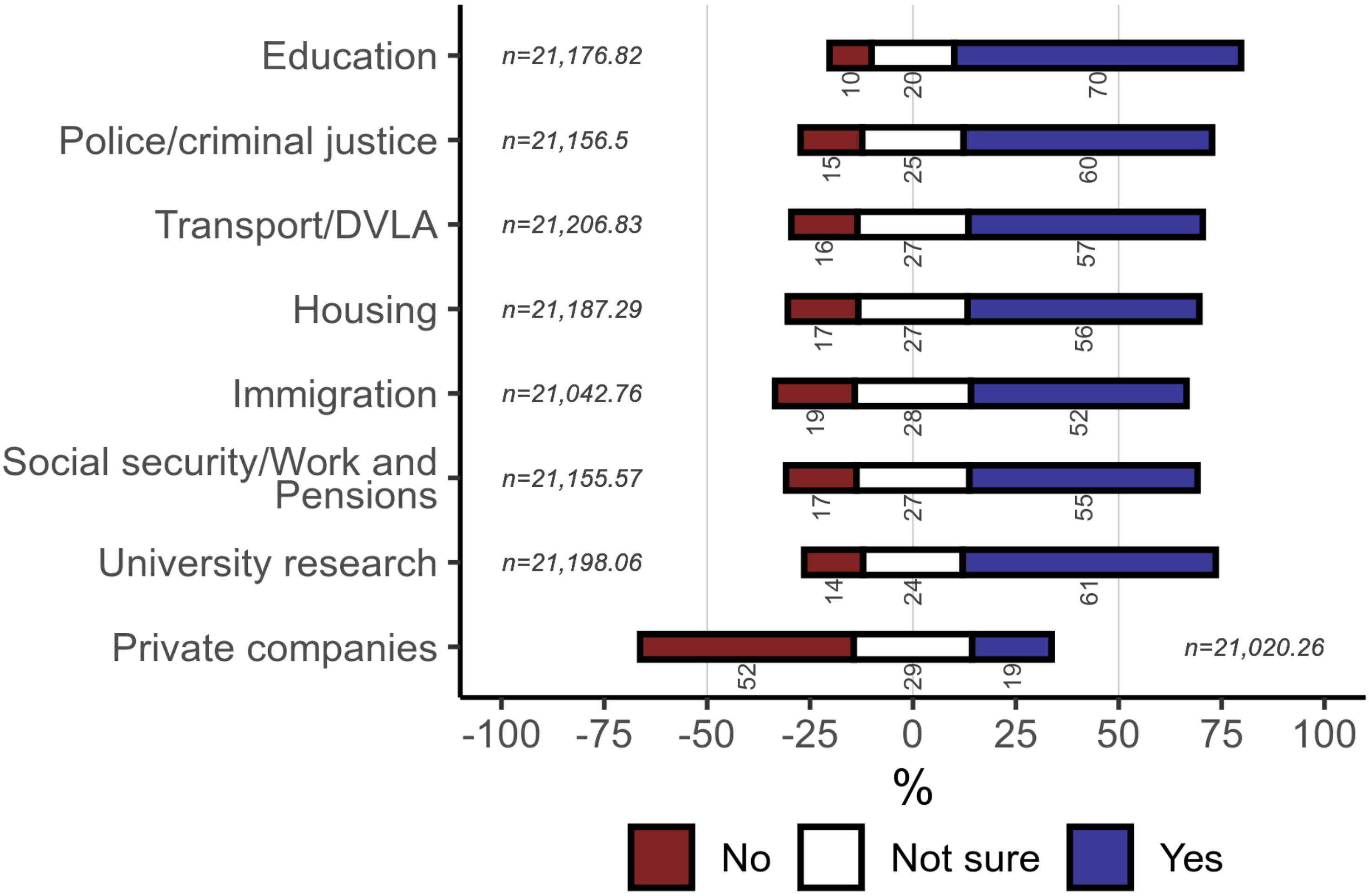

Supplementary Figure 6

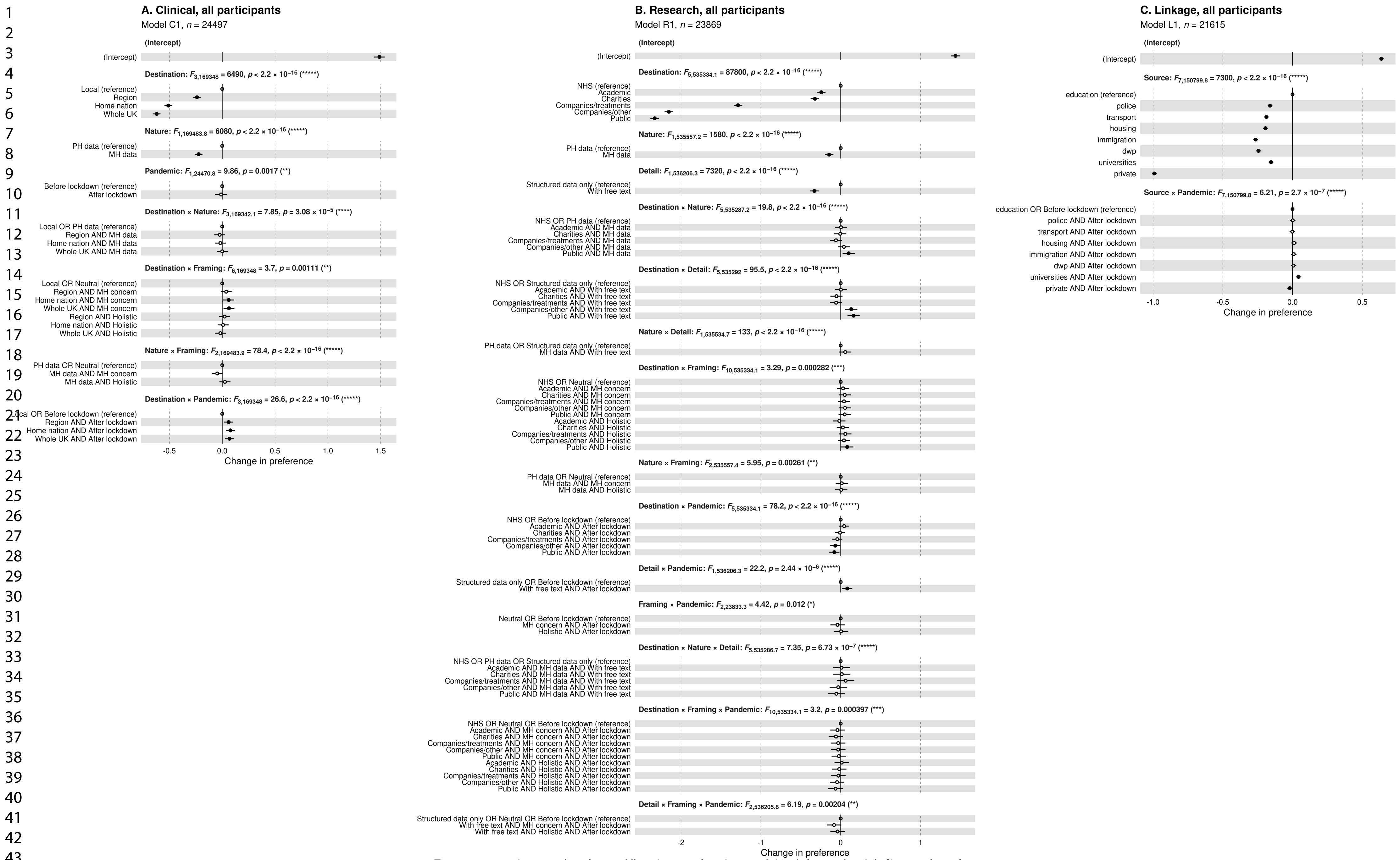

Supplementary Figure 7

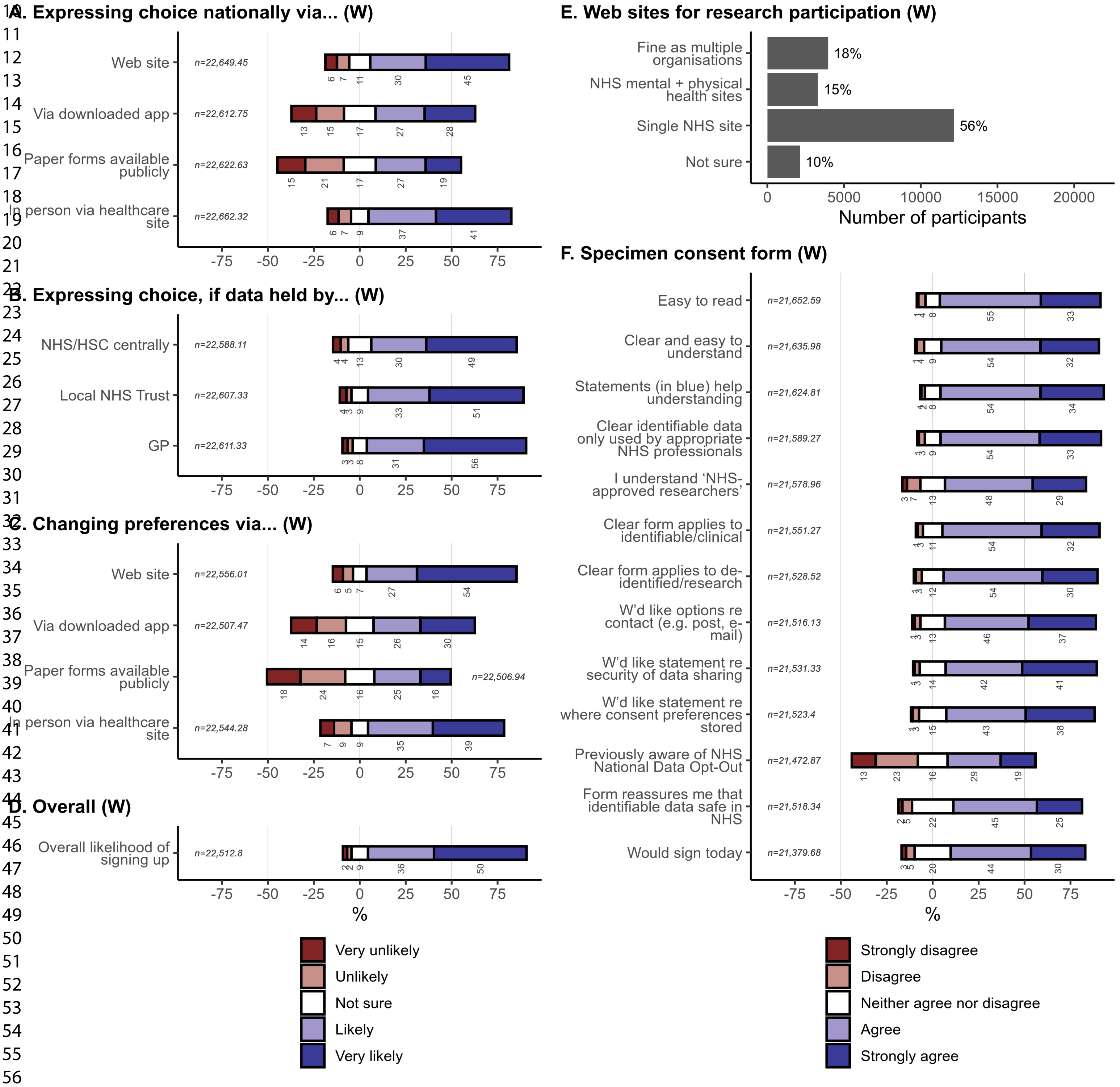

University of Cambridge NHS Health Data Consent Survey

Project Home | Project Setup | Online Designer | Data Dictionary | Codebook

Create snapshot of instruments   VIDEO: How to use this page

Last snapshot: 28/08/2019 10:22am ?

This page allows you to build and customize your data collection instruments one field at a time. You may add new fields or edit existing ones. New fields may be added by clicking the Add Field buttons. You can begin editing an existing field by clicking on the **Edit** icon. If you decide that you do not want to keep a field, you can simply delete it by clicking on the **Delete** icon. To reorder the fields, simply **drag and drop** a field to a different position within the form below. NOTE: While in development status, all field changes will take effect immediately in real time. Are you using Action Tags yet? If not, learn about Action Tags here.

Return to list of instruments   Survey settings

Current instrument: **NHS Data Consent Survey**   Preview instrument

Variable: record_id   * This field will NOT be displayed on the survey page.

Record ID

NOTE: The field above is the record ID field and thus cannot be deleted or moved. It can only be edited.

Add Field   Add Matrix of Fields

Variable: random_no_gen

Random number generator   View equation

Add Field   Add Matrix of Fields

Variable: random_no_min

Minimum random number

Add Field   Add Matrix of Fields

Variable: random_no_max

Maximum random number

Add Field   Add Matrix of Fields

Variable: random_no

Random number   View equation

Add Field   Add Matrix of Fields

Variable: date_time_1

Now   D-M-Y H:M

Add Field   Add Matrix of Fields

Matrix group: consent

Consent Form

Variable: consent_1

Please tick ALL boxes

I confirm I am aged 16 or over, or I am under 16 years of age but have the consent of my parent/guardian.
* must provide value

Variable: consent_2

I confirm that I am a resident of the UK.
* must provide value

Variable: consent_3

I understand I may quit the survey at any time but any data I have provided may still be used.
* must provide value

Variable: consent_4

I understand that the answers I give will be used for analysis and publication.
* must provide value

Variable: consent_5

I understand my answers will be recorded in a way that means I cannot be identified.
* must provide value

Variable: consent_6

I understand that not everyone will see the same questions or definitions.
* must provide value

Variable: consent_7

If I have any problems or questions with the survey, I understand I can contact Linda Jones, e-mail: laj28@cam.ac.uk, telephone: 01223 764670
* must provide value

Variable: consent_8

If I choose to leave an email address, I understand it will be removed from the survey and only used to send me the results of the survey
* must provide value

Variable: consent_9

I consent to take part in this survey.
* must provide value

Add Field Add Matrix of Fields

Variable: demo_advertising

We would like to know where you heard about this survey. It is especially important to know if you were given a leaflet (or letter) or saw a poster/text/social media from a 'healthcare provider' e.g. hospital, clinic, GP, ambulance service, healthcare community setting, or any other healthcare provider.

* must provide value

- Prefer not to say
- GP (via a poster, leaflet, from a staff member, via text, via GP Practice social media etc.)
- Hospital/Clinic/Ambulance/Healthcare community setting, other healthcare provider (via a poster, leaflet, from a staff member, text, healthcare provider's social media etc.)
- Group to which I belong
- Charity website/email/newsletter etc.
- Social media (other than from a healthcare provider)
- Poster (other than at a healthcare provider)
- Browsing the internet
- Friend
- Not sure
- Other

reset

Add Field | Add Matrix of Fields

Variable: healthcare_provider *Branching logic:* [demo_advertising] = '3'

Please could you tell us in which healthcare establishment you saw a poster or picked up/were given a leaflet? If you have the leaflet, the healthcare setting may be named on the back of it. This is really important to each healthcare provider. (Please type in a few letters and options will appear.)

* must provide value

Add Field | Add Matrix of Fields

Variable: gp *Branching logic:* [demo_advertising] = '2'

Please could you tell us in which GP Practice (surgery) you saw a poster or picked up/were given a leaflet? If you have the leaflet, the practice may be named on the back of it. This is really important to the GPs. (Please type in a few letters and options will appear.)

* must provide value

Add Field | Add Matrix of Fields

Before starting the survey please read the following definitions:

By "NHS" we mean all parts of the national health service in the UK: NHS England, NHS Scotland, NHS Wales, and Health and Social Care in Northern Ireland (HSC). We refer to all these as the NHS in this survey.

Health data means any information about a person's health such as might be collected by a nurse, doctor or other appropriate member of NHS staff. Health data includes things like your medical notes, blood test results, prescription information, scans, etc.

Identifiable health data means any health data (as described above) that can also identify a person -- such as any health data that also contains names, addresses, NHS numbers, etc.

Clinical care means providing health care directly to people -- for example, diagnosing illnesses or treating ill health.

Add Field | Add Matrix of Fields

Variable: understanding

We would like to know/gauge your understanding of how NHS clinical care providers currently share your identifiable health data for your clinical care, without asking you each time. If you are not sure, please have a "best guess" as to what you think might happen.

My understanding is that (please tick only one):

* must provide value

- My identifiable health data is currently shared freely between all NHS sites across the UK -- i.e. all acute hospital Trusts, my GP, all mental health Trusts, ambulance services, etc., in the whole UK.
- My identifiable health data is currently shared between all NHS sites but only in the home nation that I live in -- i.e. shared only in England, or Scotland, or Northern Ireland, or Wales, but not between them.
- My identifiable health data is currently only shared between NHS sites in my region (cluster of hospitals nearest to my local hospital).
- My identifiable health data is currently only shared between my GP, acute local hospital, local mental health Trust, and other local services.
- My identifiable health data is currently not shared between any NHS sites.

reset

Add Field | Add Matrix of Fields

MOST hospital Trusts, GPs, mental health Trusts are separate and currently DO NOT share identifiable health data without asking you, though they correspond and communicate with each other with your permission.

For peer review only - http://bmjopen.bmj.com/site/about/guidelines.xhtml

Add Field | Add Matrix of Fields

Variable: views_on_sharing

In view of the statement above, we would now like to ask you your views on sharing your own identifiable health data for YOUR CLINICAL CARE, without having to be asked each time (please tick only one):

* must provide value

- I would like my identifiable health data to be shared for clinical purposes with any part of the NHS in the UK, without asking me first.
- I would like my identifiable health data to be shared for clinical purposes with any part of the NHS in my home nation only (i.e. only in England, or only in Northern Ireland, or only in Scotland, or only in Wales) without asking me first.
- I would like my identifiable health data to be shared for clinical purposes with any part of my region (cluster of hospitals nearest to my local hospital), without asking me first.
- I would like my identifiable health data to be shared for clinical purposes only with my GP, local hospital, and local mental health services, without asking me first.
- No one in the NHS should share my identifiable health data without asking me first.
- Not sure.

reset

Add Field | Add Matrix of Fields

We would now like to ask you some questions about your own MENTAL and PHYSICAL health. You can "prefer not to say" if you wish.

Add Field | Add Matrix of Fields

Variable: mh_yes_or_no

We would like to ask if you have had a MENTAL health condition either recently or at some point in your life.

By mental health conditions we include things like delirium, self harm, substance abuse, dementia, mania, schizophrenia, depression, anxiety disorders, psychosis, eating disorders, OCD, personality disorders, etc.

* must provide value

- I prefer not to say.
- Yes, I have had a mental health condition either recently or at some point in my life.
- No, I have never had a mental health condition.

reset

Add Field | Add Matrix of Fields

Matrix group: mh_conditions

We would like to know if you would be willing to share what mental health condition you have had.

You may tick as many boxes as you wish.

If you prefer not to say, please use the "next page" button below to move on.

Variable: delirium *Branching logic:* [mh_yes_or_no] = '1'

	In the last 5 years	More than 5 years ago
**Delirium**	☐	☐

Variable: alcohol_substance *Branching logic:* [mh_yes_or_no] = '1'

**Alcohol or substance abuse**	☐	☐

Variable: schiz *Branching logic:* [mh_yes_or_no] = '1'

**Schizophrenia, schizoaffective disorder, or delusional disorder**	☐	☐

Variable: dementia *Branching logic:* [mh_yes_or_no] = '1'

**Dementia**	☐	☐

For peer review only - http://bmjopen.bmj.com/site/about/guidelines.xhtml

Variable: mania_bipolar *Branching logic:* [mh_yes_or_no] = '1'

Mania or bipolar affective disorder

Variable: depression *Branching logic:* [mh_yes_or_no] = '1'

Depression

Variable: anxiety *Branching logic:* [mh_yes_or_no] = '1'

Anxiety disorder (e.g. phobia, panic, generalized anxiety disorder, post-traumatic stress disorder)

Variable: psychosis *Branching logic:* [mh_yes_or_no] = '1'

Psychosis of any cause

Variable: eating_disorder *Branching logic:* [mh_yes_or_no] = '1'

Eating disorder (e.g. anorexia nervosa, bulimia)

Variable: ocd *Branching logic:* [mh_yes_or_no] = '1'

Obsessive-compulsive disorder

Variable: personality_disorder *Branching logic:* [mh_yes_or_no] = '1'

Personality disorder

Variable: self_harm *Branching logic:* [mh_yes_or_no] = '1'

Self harm

Variable: other_mh_illness *Branching logic:* [mh_yes_or_no] = '1'

Other

Add Field | Add Matrix of Fields

Variable: mh_other *Branching logic:* [other_mh_illness(1)] = '1' or [other_mh_illness(2)] = '1'

Other (please specify)

Add Field | Add Matrix of Fields

And also in relation to mental health:

Add Field | Add Matrix of Fields

Variable: mh_services_yes_no *Branching logic:* [mh_yes_or_no] = '1'

We would also like to know if you have accessed any mental health services for your own mental health condition.

These might include your GP, online services, mental health forums, psychological therapy services, crisis care, home treatments or visits, outpatient or inpatient care, services from the private sector, support from a mental health charity, etc.

* must provide value

- I prefer not to say.
- I have used mental health services.
- I have not used any mental health services (not even my GP or surgery) for my mental health condition.

reset

Add Field | Add Matrix of Fields

Matrix group: mh_services

Variable: mh_gp *Branching logic:* [mh_services_yes_no] = '1'

	In the last 5 years	More than 5 years ago
**Mental health support from your GP or surgery**		

Variable: mh_iapt_individual *Branching logic:* [mh_services_yes_no] = '1'

An NHS psychological therapy service, e.g. IAPT or other therapy service (INDIVIDUAL session)

Variable: mh_iapt_group *Branching logic:* [mh_services_yes_no] = '1'

An NHS psychological therapy service, e.g. IAPT or other therapy service (GROUP session)

Variable: mh_iapt_online *Branching logic:* [mh_services_yes_no] = '1'

An NHS psychological therapy service, e.g. IAPT or other therapy service (ONLINE)

Variable: mh_op *Branching logic:* [mh_services_yes_no] = '1'

NHS outpatient or community mental health services, or key worker

Variable: mh_crisis *Branching logic:* [mh_services_yes_no] = '1'

NHS crisis care/Home Treatment teams

Variable: mh_ip_mh *Branching logic:* [mh_services_yes_no] = '1'

NHS inpatient mental health services in a mental health hospital

Variable: mh_ip_general *Branching logic:* [mh_services_yes_no] = '1'

NHS inpatient mental health services in a general hospital (liaison psychiatry)

Variable: mh_private *Branching logic:* [mh_services_yes_no] = '1'

Private services

Variable: mh_oh *Branching logic:* [mh_services_yes_no] = '1'

Services in the workplace e.g. Occupational Health

Variable: mh_charity *Branching logic:* [mh_services_yes_no] = '1'

Charity providing mental health support

Variable: mh_forum *Branching logic:* [mh_services_yes_no] = '1'

Online forum

Variable: mh_other_service *Branching logic:* [mh_services_yes_no] = '1'

Other

Add Field Add Matrix of Fields

Variable: mh_service_other_detail *Branching logic:* [mh_other_service(1)] = '1' or [mh_other_service(2)] = '1'

Other (please specify)

Add Field Add Matrix of Fields

We are also interested to know if you have accessed any services regarding your own PHYSICAL health.

This could be services like your GP, outpatient hospital appointments, inpatient services, online support, Accident and Emergency, 111 service, private services, charities providing physical health support, etc.

Add Field | Add Matrix of Fields

Variable: ph_services_yes_no

Have you ever used any of the following services for your own PHYSICAL health?

* must provide value

- I prefer not to say
- I have used physical health services (this includes using your GP)
- I have not used any physical health services

reset

Add Field | Add Matrix of Fields

Matrix group: ph_services

And finally in this section, we would like to know if you would be willing to share what physical health services you have used for your own physical health.

You may tick as many boxes as you wish.

Please use the 'next page' button if you prefer not to answer.

	In the last 5 years	More than 5 years ago
Variable: ph_gp *Branching logic:* [ph_services_yes_no] = '1'		
**Physical health support from your GP or surgery**	☐	☐
Variable: ph_op *Branching logic:* [ph_services_yes_no] = '1'		
**NHS hospital outpatient services (outpatient clinics etc.)**	☐	☐
Variable: ph_urgent_care *Branching logic:* [ph_services_yes_no] = '1'		
**NHS Urgent Care Centre (not A&E) or NHS 111 helpline**	☐	☐
Variable: ph_accident *Branching logic:* [ph_services_yes_no] = '1'		
**NHS Accident and Emergency services (A&E)**	☐	☐
Variable: ph_ip *Branching logic:* [ph_services_yes_no] = '1'		
**NHS inpatient services (procedures not completed in 1 day)**	☐	☐
Variable: ph_day *Branching logic:* [ph_services_yes_no] = '1'		
**NHS Day Surgery/day care in a hospital**	☐	☐
Variable: ph_private *Branching logic:* [ph_services_yes_no] = '1'		
**Private services**	☐	☐
Variable: ph_oh *Branching logic:* [ph_services_yes_no] = '1'		
**Services in the workplace e.g. Occupational Health**	☐	☐
Variable: ph_charity *Branching logic:* [ph_services_yes_no] = '1'		
**Charity providing physical health support**	☐	☐
Variable: ph_online *Branching logic:* [ph_services_yes_no] = '1'		
**Online forum**	☐	☐
Variable: ph_other_services *Branching logic:* [ph_services_yes_no] = '1'		
**Other**	☐	☐

For peer review only - http://bmjopen.bmj.com/site/about/guidelines.xhtml

Add Field Add Matrix of Fields

Variable: ph_other *Branching logic:* [ph_other_services(1)] = '1' or [ph_other_services(2)] = '1'

Other (please specify)

Add Field Add Matrix of Fields

Moving on...

Add Field Add Matrix of Fields

Variable: identifiable_description

The table below shows what NHS identifiable health data may look like. This sort of data is used for health care by NHS staff. (Please note the examples shown are not real.)

NHS number	Title	Forename	Surname	Date of birth	Address	Diagnoses	Notes
9876543210	Mr	John	Smith	20 Jan 1950	1 The Street, Maidstone	pneumonia<br>high blood pressure	1 Apr 2019. Seen in clinic. Mr Smith has a fever and a cough. His chest sounds crackly. I think he has pneumonia. Sent to hospital.
8765432109	Miss	Alice	Jones	16 Jun 1994	15 The Road, Dundee	depression<br>carpal tunnel syndrome	2 Apr 2019. Alice rates her mood as 1/10 on average, she is more tired, and she is not looking forward to anything. Her depression has worsened. She wishes to restart citalopram.
7654321098	Mrs	Chloe	Williams	4 May 1930	5 Tree Close, Cardiff	broken humerus<br>anxiety	3 Apr 2019. Chloe fell after skidding on an oily patch while cycling and was hit by a car. Has an obvious fracture of her left upper arm. Needs X-ray.
6543210987	Mr	Pradeep	Agarwal	22 Sep 1973	27 The Mews, London	schizophrenia<br>diabetes	4 Apr 2019. Pradeep still worries that other people can hear his thoughts, but this is getting less common. He is attending cognitive–behavioural therapy sessions.

Add Field Add Matrix of Fields

Matrix group: share_ph_1

Previous surveys have found that people have more concerns about the use of their identifiable health data relating to their mental health than other aspects of their physical health care.

Regardless of whether you have a PHYSICAL health condition or have used PHYSICAL health services:

How likely are you to agree to share your identifiable PHYSICAL health data for your clinical care with the following, without being asked every time?

Variable: share_ph_local_1 *Branching logic:* [random_no] = '1'

	Very likely	Likely	Not sure	Unlikely	Very unlikely	Prefer not to say
**Between my local NHS services -- i.e. between my local physical and mental health hospitals, my GP, etc.** * must provide value	◯	◯	◯	◯	◯	◯

reset

Variable: share_ph_region_1 *Branching logic:* [random_no] = '1'

**Any part of the NHS in my region (cluster of hospitals nearest to my local hospital)** * must provide value	◯	◯	◯	◯	◯	◯

reset

Variable: share_ph_nation_1 *Branching logic:* [random_no] = '1'

**Any part of the NHS in my home nation only (i.e. only in England, or only in Northern Ireland, or only in Scotland, or only in Wales)** * must provide value	◯	◯	◯	◯	◯	◯

reset

Variable: share_ph_uk_1 *Branching logic:* [random_no] = '1'

**Any part of the NHS in the UK** * must provide value	◯	◯	◯	◯	◯	◯

reset

Add Field | Add Matrix of Fields

Matrix group: share_ph_2

We would like to find out your perspective on using information about your mental health and your physical health.

Regardless of whether you have a PHYSICAL health condition or have used PHYSICAL health services:

How likely are you to agree to share your identifiable PHYSICAL health data for your clinical care with the following, without being asked every time?

Variable: share_ph_local_2 *Branching logic:* [random_no] = '2'

	Very likely	Likely	Not sure	Unlikely	Very unlikely	Prefer not to say
**Between my local NHS services -- i.e. between my local physical and mental health hospitals, my GP, etc.** * must provide value	◯	◯	◯	◯	◯	◯

reset

Variable: share_ph_region_2 *Branching logic:* [random_no] = '2'

**Any part of the NHS in my region (cluster of hospitals nearest to my local hospital)** * must provide value	◯	◯	◯	◯	◯	◯

reset

Variable: share_ph_nation_2 *Branching logic:* [random_no] = '2'

**Any part of the NHS in my home nation only (i.e. only in England, or only in Northern Ireland, or only in Scotland, or only in Wales)** * must provide value	◯	◯	◯	◯	◯	◯

reset

Variable: share_ph_uk_2 *Branching logic:* [random_no] = '2'

**Any part of the NHS in the UK** * must provide value	◯	◯	◯	◯	◯	◯

reset

Add Field | Add Matrix of Fields

Matrix group: share_ph_3

Mental and physical illnesses overlap, so holistic health care is important. Mental health problems have physical consequences, and physical illnesses have important consequences for mental health.

Regardless of whether you have a PHYSICAL health condition or have used PHYSICAL health services:

How likely are you to agree to share your identifiable PHYSICAL health data for your clinical care with the following, without being asked every time?

Variable: share_ph_local_3 *Branching logic:* [random_no] = '3'

	Very likely	Likely	Not sure	Unlikely	Very unlikely	Prefer not to say
**Between my local NHS services -- i.e. between my local physical and mental health hospitals, my GP, etc.** * must provide value	◯	◯	◯	◯	◯	◯

reset

Variable: share_ph_region_3 *Branching logic:* [random_no] = '3'

**Any part of the NHS in my region (cluster of hospitals nearest to my local hospital)** * must provide value	◯	◯	◯	◯	◯	◯

reset

Variable: share_ph_nation_3 *Branching logic:* [random_no] = '3'

**Any part of the NHS in my home nation only (i.e. only in England, or only in Northern Ireland, or only in Scotland, or only in Wales)** * must provide value	◯	◯	◯	◯	◯	◯

reset

Variable: share_ph_uk_3 *Branching logic:* [random_no] = '3'

**Any part of the NHS in the UK** * must provide value	◯	◯	◯	◯	◯	◯

reset

Add Field | Add Matrix of Fields

Matrix group: share_mh

Regardless of whether you have a MENTAL health condition or have used MENTAL health services:

How likely are you to agree to share your identifiable MENTAL health data for your clinical care with the following, without being asked every time?

Variable: share_mh_local

	Very likely	Likely	Not sure	Unlikely	Very unlikely	Prefer not to say
**Between my local NHS services -- i.e. between my local physical and mental health hospitals, my GP, etc.** * must provide value	◯	◯	◯	◯	◯	◯

reset

Variable: share_mh_region

**Any part of the NHS in my region (cluster of hospitals nearest to my local hospital)** * must provide value	◯	◯	◯	◯	◯	◯

reset

For peer review only - http://bmjopen.bmj.com/site/about/guidelines.xhtml

Variable: share_mh_nation

Any part of the NHS in my home nation only (i.e. only in England, or only in Northern Ireland, or only in Scotland, or only in Wales)
* must provide value

reset

Variable: share_mh_uk

Any part of the NHS in the UK
* must provide value

reset

Add Field Add Matrix of Fields

In the next two questions we would like to ask you about sharing your NHS data for research purposes. Research is used to improve overall healthcare for everyone.

Add Field Add Matrix of Fields

Variable: deidentified_description

Firstly, please look at some of the NHS health care data that you saw previously, which has now been de-identified.

Research ID	Age	Sex	Region	Diagnoses
5X62V	69	M	Kent	pneumonia<br>high blood pressure
597PT	24	F	Dundee	depression<br>carpal tunnel syndrome
8HG7S	89	F	Glamorganshire	broken humerus<br>anxiety
BA6A9	45	M	London	schizophrenia<br>diabetes

Add Field Add Matrix of Fields

Matrix group: ph_non_ident_research

How likely would you be to share your de-identified PHYSICAL health data with the following, without giving consent every time?

Variable: ph_non_id_nhs

	Very likely	Likely	Not sure	Unlikely	Very unlikely	Prefer not to say
**Any part of the NHS doing research** * must provide value						

reset

Variable: ph_non_id_academic

Academic institutions doing research (e.g. universities)
* must provide value

reset

Variable: ph_non_id_charity

A national charity doing research (e.g. MIND, Cancer Research UK, British Heart Foundation)
* must provide value

reset

Variable: ph_non_id_profit_tx

A profit-making company doing research into treatments (e.g. a pharmaceutical company, health technology company)
* must provide value

reset

Variable: ph_non_id_profit_other

A profit-making company doing other research (e.g. an insurance company, broadband provider)
* must provide value

reset

Variable: ph_non_id_public

Publicly
* must provide value

reset

Add Field | Add Matrix of Fields

Matrix group: mh_non_id_research

How likely would you be to share your de-identified MENTAL health data with the following, without giving consent every time:

Variable: mh_non_id_nhs

	Very likely	Likely	Not sure	Unlikely	Very unlikely	Prefer not to say
**Any part of the NHS doing research** * must provide value						
**Academic institutions doing research (e.g. universities)** * must provide value						
**A national charity doing research (e.g. MIND, Cancer Research UK, British Heart Foundation)** * must provide value						
**A profit-making company doing research into treatments (e.g. pharmaceutical company, health technology company)** * must provide value						
**A profit-making company doing other research (e.g. insurance company, broadband provider)** * must provide value						
**Publicly** * must provide value						

Variables: mh_non_id_nhs, mh_non_id_academic, mh_non_id_charity, mh_non_id_profit_tx, mh_non_id_profit_other, mh_non_id_public (each with reset)

Add Field | Add Matrix of Fields

The final question in this section relates to sharing de-identified "free text" data for research.

Add Field | Add Matrix of Fields

Variable: deidentified_ft_descript

Here is the table of de-identified health care data you saw earlier, but this time a column labelled "Notes" has been added. This is known as "free text" data.

Research ID	Age	Sex	Region	Diagnoses	Notes
5X62V	69	M	Kent	pneumonia<br>high blood pressure	1st April 2019. XXX has a fever and cough. His chest sounds crackly. I think he has pneumonia. Sent to hospital.
597PT	24	F	Dundee	depression<br>carpal tunnel syndrome	2nd April 2019. XXX rates her mood as 1/10 on average, she is more tired, and she is not looking forward to anything. Her depression has worsened. She wishes to restart citalopram.
8HG7S	89	F	Glamorganshire	broken humerus<br>anxiety	3rd April 2019. XXX fell after skidding on an oily patch while cycling and was hit by a car. She has an obvious fracture of her left upper arm. She needs an X-ray.
BA6A9	45	M	London	schizophrenia<br>diabetes	4th April 2019. XXX still worries that other people can hear his thoughts, but this is getting less common. He is attending cognitive-behavioural therapy sessions.

Like the last example the data remains de-identified but gives the researchers more information. As there is more information there is a slightly increased risk of someone being identified from the data.

For example, in the table above, an "89-year-old cyclist skidding on oil and being hit by a car" may be reported in a local newspaper. Whilst the lady is not named in the data above, she would almost certainly be named in the newspaper and it may therefore be possible for researchers to work out who she is.

Add Field | Add Matrix of Fields

Matrix group: ph_free_text

How likely would you be to share your FREE TEXT de-identified PHYSICAL health data with the following, without giving consent every time:

Variable: ph_ft_nhs

	Very likely	Likely	Not sure	Unlikely	Very unlikely	Prefer not to say
**Any part of the NHS doing research**<br>* must provide value	○	○	○	○	○	○

reset

Variable: ph_ft_academic

**Academic institutions doing research (e.g. universities)**<br>* must provide value	○	○	○	○	○	○

reset

Variable: ph_ft_charity

**A national charity doing research (e.g MIND, Cancer Research UK, British Heart Foundation)**<br>* must provide value	○	○	○	○	○	○

reset

Variable: ph_ft_profit_tx

**A profit-making company doing research into treatments (e.g. pharmaceutical company, health technology company)**<br>* must provide value	○	○	○	○	○	○

reset

Variable: ph_ft_profit_other

A profit-making company doing other research (e.g. insurance company, broadband provider)
* must provide value

reset

Variable: ph_ft_public

Publicly
* must provide value

reset

Add Field | Add Matrix of Fields

Matrix group: mh_free_text

How likely would you be to share your FREE TEXT de-identified MENTAL health data with the following, without giving consent each time:

Variable: mh_ft_nhs

	Very likely	Likely	Not sure	Unlikely	Very unlikely	Prefer not to say
**Any part of the NHS doing research** * must provide value	◯	◯	◯	◯	◯	◯

reset

Variable: mh_ft_academic

Academic institutions doing research (e.g. universities)
* must provide value

reset

Variable: mh_ft_charity

A national charity doing research (e.g MIND, Cancer Research UK, British Heart Foundation)
* must provide value

reset

Variable: mh_ft_profit_tx

A profit-making company doing research into treatments (e.g. pharmaceutical company, health technology company)
* must provide value

reset

Variable: mh_ft_profit_other

A profit-making company doing other research (e.g. insurance company, broadband provider)
* must provide value

reset

Variable: mh_ft_public

Publicly
* must provide value

reset

Add Field | Add Matrix of Fields

Matrix group: consent_signup_place

On a slightly different topic...

Imagine that there was a single place where you could securely sign up to choose how your NHS identifiable health data is used. This would cover how your confidential patient information is managed across all UK NHS services, and how your NHS de-identified data could (or could not) be used for research.

It would be in the form of a standardised UK-wide CONSENT FORM that all NHS services could use.

How likely would you be to sign up if you were asked to sign:

Variable: signup_online

	Very likely	Likely	Not sure	Unlikely	Very unlikely	Prefer not to say
**Online (via a website)** * must provide value						

reset

Variable: signup_app

**By downloading an app** * must provide value						

reset

Variable: signup_paper

**On paper forms available from pharmacies, the Post Office, etc. (and sent by post)** * must provide value						

reset

Variable: signup_in_person

**In person (e.g. when attending your GP, at a hospital clinic, etc.)** * must provide value						

reset

Add Field Add Matrix of Fields

Matrix group: consent_stored

Would you be likely to sign up if your consent form were then stored and managed securely by the following institutions?

Variable: stored_centrally

	Very likely	Likely	Not sure	Unlikely	Very unlikely	Prefer not to say
**Centrally by NHS England/NHS Scotland/NHS Wales/HSC Northern Ireland** * must provide value						

reset

Variable: stored_locally

**Your local NHS Trust** * must provide value						

reset

Variable: stored_gp

**Your GP** * must provide value						

reset

Add Field Add Matrix of Fields

Matrix group: consent_change

If you wanted to change your preferences, how likely would you be to use the following methods?

Variable: change_online

	Very likely	Likely	Not sure	Unlikely	Very unlikely	Prefer not to say
**Online (via a website)** * must provide value						

reset

Variable: change_app

**By downloading an app** * must provide value						

reset

For peer review only - http://bmjopen.bmj.com/site/about/guidelines.xhtml

Variable: change_paper

**On paper forms available from pharmacies, Post Office, etc. (and sent by post)** * must provide value	◯	◯	◯	◯	◯	◯

reset

Variable: change_in_person

**In person (e.g. when attending your GP, at a hospital clinic, etc.)** * must provide value	◯	◯	◯	◯	◯	◯

reset

Add Field | Add Matrix of Fields

Matrix group: likelihood_signup

Assume that you were able to sign up and change your choices in a way you preferred, and that the consent form was stored securely in a place where you felt comfortable.

How likely would you be to use this system to choose how your NHS health data is managed?

Variable: likelihood_signup

	Very likely	Likely	Not sure	Unlikely	Very unlikely	Prefer not to say
**Overall likelihood that you would sign up** * must provide value	◯	◯	◯	◯	◯	◯

reset

Add Field | Add Matrix of Fields

The last part of the survey (before we ask a few questions to ensure we have reached a broad section of the UK) examines what a consent form to share our health data could look like.

By completing one consent form it would then be possible for all UK NHS professionals to access their patient's identifiable health data for their clinical care, saving time for both patients and health care professionals.

Please take a look at the consent form below:

Add Field Add Matrix of Fields

Variable: consent_form_descript

National NHS/HSC data sharing consent form

Full name ______________________
Date of birth ___________
NHS number ______________________
Address ________________________________
____
E-mail address ______________________

I confirm I have read the information sheet 'Sharing My Health Data' version XXX dated XXX. I have had the opportunity to consider the information and ask questions. I understand that my participation is voluntary, and that I am free to change or withdraw my consent at any time, without giving a reason and without my medical care or rights being affected. Yes [ ]

1. Providing health care to you

Confidential patient information is information that can identify you and that says something about your health care or treatment. Information about you might be held by several NHS organizations (such as GP surgeries and hospitals). May they share your information with each other when providing health care to you?

I agree that all NHS care providers and professionals may share my confidential patient information with each other for the purpose of my treatment and care. Yes [ ] No [ ]

2. Using your de-identified data to help others

The NHS promises to **anonymise** the information collected during the course of your treatment and use it to support research and improve care for others. Research is conducted by the NHS and by NHS-approved researchers in organizations such as universities. Strict security controls apply to the use of NHS data, even after information that might identify you (such as names and addresses) has been removed. All research involving NHS data must be approved by the NHS.

Saying no doesn't prevent all uses of your confidential information for research. To do that, use the national data opt-out at https://www.nhs.uk/your-nhs-data-matters.

I agree that all NHS care providers may share my confidential patient information with each other and de-identify it for the purpose of research. Yes [ ] No [ ]

3. Taking part in research

Some research involves **direct participation.** This ranges from questionnaires to trials of new treatments. The NHS promises to inform you of research studies in which you may be eligible to participate. There is never a commitment to take part.

I agree that NHS-approved researchers may learn my identity and contact me directly about research studies for which I may be eligible. Yes [ ] No [ ]

Signature: ______________________

Date: ___________

Add Field Add Matrix of Fields

Matrix group: consent_form

Having looked at the consent form, please agree or disagree with the following statements.

(If you would like to take another look at the consent form at any time while answering this question, you can use the 'previous' button at the bottom of this page to look back. This will not affect answers already given.)

For peer review only - http://bmjopen.bmj.com/site/about/guidelines.xhtml

	Strongly agree	Agree	Neither agree nor disagree	Disagree	Strongly disagree	Prefer not to say
Variable: consent_read						
**The consent form is easy to read.** * must provide value						reset
Variable: consent_clear						
**The consent form is clear and easy to understand.** * must provide value						reset
Variable: consent_help						
**The statements (in blue) help me to understand what I am consenting to.** * must provide value						reset
Variable: consent_professionals						
**The consent form is clear that my identifiable health data would only be used by appropriate NHS health care professionals.** * must provide value						reset
Variable: consent_researchers						
**I understand the term 'NHS-approved researchers' and which individuals this may refer to.** * must provide value						reset
Variable: consent_identifiable_care						
**The consent form makes clear that it would be used for my preferences about sharing my IDENTIFIABLE health data within the NHS for my CLINICAL CARE.** * must provide value						reset
Variable: consent_non_id_research						
**The consent form makes clear that it would also be used for my preferences about sharing my DE-IDENTIFIED health data for RESEARCH purposes.** * must provide value						reset
Variable: consent_add_contact						
**I would like the consent form to give specific options about how researchers can contact me (e.g. by post only, e-mail).** * must provide value						reset
Variable: consent_add_security						
**I would like to see a statement added about the security of data sharing.** * must provide value						reset
Variable: consent_add_storage						
**I would like to see a statement added about where my consent preferences will be stored.** * must provide value						reset

Variable: consent_opt_out

Before seeing this consent form I was aware of the NHS National Data Opt-Out.
* must provide value

reset

Variable: consent_nhs_secure

The consent form reassures me that my identifiable health data is safe in the NHS.
* must provide value

reset

Variable: consent_sign

If this consent form were put in front of me today, I would sign it.
* must provide value

reset

Add Field Add Matrix of Fields

Variable: consent_comments

If you would like to make any comments about the consent form, please do so here:

Expand

Add Field Add Matrix of Fields

There are currently many individual websites where people can sign up to take part in research. Examples include charity websites, the national "Join Dementia Research" register, and the National Institute for Health Research (NIHR) "be part of research" campaign.

Add Field Add Matrix of Fields

Variable: national_sign_up

An alternative might be a national sign-up portal, on a web site, where people could register their preferences about taking part in all NHS research.

Choose the statement you agree with most:

* must provide value

- Everything is fine as it is; leave it to individuals to sign up with the various organisations.
- Have two national NHS research websites, one where people can sign up for mental health research, and a second website where people can sign up for physical health research.
- Have a national NHS research website where people can sign up for all conditions.
- Not sure.

reset

Add Field | Add Matrix of Fields

Matrix group: linked_data

Finally, it can be very valuable for research to link health (NHS) data to other data sources.

For example, causes of death (from death certificates) are held by the UK Office of National Statistics (ONS), rather than the NHS -- so to find out more about the reasons people die, NHS and ONS data must be linked.

Usually, this is done as follows:
a) Research teams seek special permission for the process.
b) The relevant information from each organization, plus a small amount of identifiable information, is given to a "trusted third party" (e.g. an NHS Trust, the Office of National Statistics).
c) The trusted third party links the information, then removes any details that can identify anyone.
d) Researchers are then given access to the de-identified information only, under special controls.

We are interested to hear whether you would be happy for your health data to be linked, in this way, to:

Variable	Item	Yes	Not sure	No	Prefer not to say
link_education	**Educational data (e.g. to study impact of illness on education)** * must provide value	○	○	○	○
link_police	**Police/Criminal Justice data (e.g. to study health in the victims of crime)** * must provide value	○	○	○	○
link_transport	**Transport/DVLA data (e.g. to study health and pollution)** * must provide value	○	○	○	○
link_housing	**Housing data (e.g. impact of social housing on health)** * must provide value	○	○	○	○
link_immigration	**Immigration data (e.g. health in immigrants)** * must provide value	○	○	○	○
link_dwp	**Social security/Work and Pensions (e.g. health and financial insecurity)** * must provide value	○	○	○	○
link_universities	**Data held about you for research by universities (e.g. if you have volunteered for research studies)** * must provide value	○	○	○	○
link_private	**Data held about you by private companies** * must provide value	○	○	○	○

reset

Add Field | Add Matrix of Fields

And finally, to ensure we have surveyed a wide range of the population (please note, all questions have a "prefer not to say" option and have been written to apply to all of the home nations):

Add Field | Add Matrix of Fields

Variable: demo_gender

Do you consider yourself to be:

* must provide value

- Prefer not to say
- Female
- Male
- Prefer to self describe

reset

Add Field | Add Matrix of Fields

Variable: demo_gender_self *Branching logic:* [demo_gender] = '4'

Prefer to self describe (please specify)

Add Field | Add Matrix of Fields

Variable: demo_age

May we know which age range you fit in?

* must provide value

- Prefer not to say
- Under 12
- 12-15
- 16-17
- 18-24
- 25-34
- 35-44
- 45-54
- 55-64
- 65-74
- 75-84
- 85+

reset

Add Field | Add Matrix of Fields

Variable: under_16_help *Branching logic:* [demo_age] = '2' or [demo_age] = '3'

Please tick which of the two options applies:

* must provide value

- I completed the survey by myself
- I had help to complete the survey

reset

Add Field | Add Matrix of Fields

Variable: demo_ethnicity

What do you consider your ethnicity to be?

* must provide value

- Prefer not to say
- White
- Irish Traveller
- Mixed/Multiple ethnic groups
- Asian or Asian British
- Indian
- Pakistani
- Bangladeshi
- Chinese
- Black/African/Caribbean/Black British
- Arab
- Other or prefer to self describe

reset

Add Field | Add Matrix of Fields

Variable: demo_ethnicity_other *Branching logic:* [demo_ethnicity] = '12'

Other or prefer to self describe (please specify)

Add Field | Add Matrix of Fields

Variable: demo_education

What is the highest qualification you have achieved?

* must provide value

- Prefer not to say
- No formal qualifications
- Secondary school level qualifications e.g. CSE, GCSE, O-Levels, Nationals, or equivalent
- A-Levels, Highers, or equivalent
- Vocational qualification or equivalent
- Undergraduate degree or equivalent
- Postgraduate or professional qualification or equivalent

reset

Add Field | Add Matrix of Fields

Variable: demo_sexuality

Do you consider yourself to be

* must provide value

- Prefer not to say
- Heterosexual (straight)
- Homosexual (gay or lesbian)
- Bisexual
- Other or prefer to self describe

reset

Add Field | Add Matrix of Fields

Variable: demo_sexuality_other *Branching logic:* [demo_sexuality] = '5'

Other or prefer to self describe (Please specify)

Add Field | Add Matrix of Fields

Variable: demo_religion

What is your religion, if any?

* must provide value

- Prefer not to say
- No religion
- Christian
- Muslim
- Hindu
- Sikh
- Jewish
- Buddhist
- Other or prefer to self describe

reset

Add Field | Add Matrix of Fields

Variable: demo_religion_other *Branching logic:* [demo_religion] = '9'

Other or prefer to self describe (please specify)

Add Field | Add Matrix of Fields

Variable: demo_se_1

Thinking about your current (or last) main job or occupation. Do (did) you work as an employee or are (were) you self-employed?

* must provide value

- Prefer not to say
- Never worked
- Employee
- Self-employed with employees
- Self-employed/freelance without employees

reset

Add Field | Add Matrix of Fields

Variable: demo_se_2 *Branching logic:* [demo_se_1] = '3'

How many people work (worked) for your employer at the place where you work (worked)?

- Prefer not to say
- 1-24
- 25 or more

reset

Add Field | Add Matrix of Fields

Variable: demo_se_3 *Branching logic:* [demo_se_1] = '4'

How many people do (did) you employ?

- Prefer not to say
- 1-24
- 25 or more

reset

Add Field | Add Matrix of Fields

Variable: demo_se_4 *Branching logic:* ([demo_se_1] = '3' and ([demo_se_2] = '2' or [demo_se_2] = '3'...

Do (did) you supervise any other employees on a day to day basis?

- Prefer not to say
- Yes
- No

reset

Add Field | Add Matrix of Fields

Variable: demo_se_5 *Branching logic:* ([demo_se_1] = '3' and ([demo_se_2] = '2' or [demo_se_2] = '3'...

And which best describes the work you do (did)?

- Prefer not to say
- Modern professional occupations such as: teacher - nurse - physiotherapist - social worker - welfare officer - artist - musician - police office (sergeant or above) - software designer
- Clerical and intermediate occupations such as: secretary - personal assistant - clerical worker - office clerk - call centre agent - nursing auxiliary - nursery nurse
- Senior managers or administrators (responsible for planning, organising, and co-coordinating work and for finance) such as: finance manager - chief executive
- Technical and craft occupations such as: motor mechanic - fitter - inspector - plumber - printer - tool maker - electrician - gardener - train driver
- Semi-routine manual and service occupations such as: postal worker - machine operative - security guard - caretaker - farm worker - catering assistant - receptionist - sales assistant
- Routine manual and service occupations such as: HGV driver - van driver - cleaner - porter - packer - sewing machinist - messenger - labourer - waiter/waitress - bar staff
- Middle or junior managers such as: office manager - retail manager - bank manager - restaurant manager - warehouse manager - publican
- Traditional professional occupations such as: accountant - solicitor - medical practitioner - scientist - civil/mechanical engineer

reset

Add Field | Add Matrix of Fields

Variable: demo_country

And where in the UK do you currently live?

* must provide value

- Prefer not to say
- England
- Northern Ireland
- Scotland
- Wales
- Channel Islands
- Isle of Man
- None of these

reset

For peer review only - http://bmjopen.bmj.com/site/about/guidelines.xhtml

Add Field | Add Matrix of Fields

Variable: demo_eng_region *Branching logic:* [demo_country] = '2'

Please specify the region you live in

* must provide value

- Prefer not to say
- North East
- North West
- Yorkshire and Humber
- West Midlands
- East Midlands
- East of England
- London
- South East
- South West
- Other

reset

Add Field | Add Matrix of Fields

Variable: demo_sco_region *Branching logic:* [demo_country] = '4'

Please specify the region you live in

* must provide value

- Prefer not to say
- Central Scotland
- Glasgow
- Highlands and Islands
- Lothian
- Mid Scotland and Fife
- North East Scotland
- South Scotland
- West Scotland
- Other

reset

Add Field | Add Matrix of Fields

Variable: demo_wales_region *Branching logic:* [demo_country] = '5'

Please specify the region you live in

* must provide value

- Prefer not to say
- West Wales and the Valleys
- East Wales
- Other

reset

Add Field | Add Matrix of Fields

Variable: demo_ni_region *Branching logic:* [demo_country] = '3'

Please specify the region you live in

* must provide value

- Prefer not to say
- Antrim and Newtownabbey
- Ards and North Down
- Armagh City, Banbridge and Craigavon
- Belfast
- Causeway Coast and Glens
- Derry City and Strabane
- Fermanagh and Omagh
- Lisburn and Castlereagh
- Mid and East Antrim
- Mid Ulster
- Newry Mourne and Down
- Other

reset

Add Field | Add Matrix of Fields

Variable: demo_sco_region_other *Branching logic:* [demo_sco_region] = '10'

Other (Please specify)

Add Field | Add Matrix of Fields

Variable: demo_eng_regio_other *Branching logic:* [demo_eng_region] = '11'

Other (please specify)

Add Field | Add Matrix of Fields

Variable: demo_wales_region_other *Branching logic:* [demo_wales_region] = '4'

Other (please specify)

Add Field | Add Matrix of Fields

Variable: demo_ni_region_other *Branching logic:* [demo_ni_region] = '13'

Other (please specify)

Add Field | Add Matrix of Fields

Variable: demo_postcode

We would like to know your postcode so that we can analyse our results by "geography", such as urban versus rural areas. You do not have to give this. However if you do, we will never disclose it or identify you from it.

* must provide value

- I prefer not to give my postcode
- I am happy to give my postcode

reset

Add Field | Add Matrix of Fields

Variable: demo_postcode_actual *Branching logic:* [demo_postcode] = '2'

Postcode

Add Field | Add Matrix of Fields

Variable: email

Before you submit your answers there is the option to leave an e-mail address should you wish to have the final report of the survey results personally e-mailed to you.

Please note: all e-mail addresses will be removed from the survey answers so that you cannot be identified, and will be held securely on password-protected computers at the University of Cambridge until the results are available, as described in the information sheet at the start of the survey.

If you wish to leave an e-mail address, please do so in the box below (and then submit your answers). Otherwise, please submit your answers now.

Optional

Add Field | Add Matrix of Fields

Resize font: | Returning?

NHS Data Consent Survey

What am I being invited to do?

You are being invited to take part in an online survey about the UK's views on sharing National Health Service (NHS) (or Health and Social Care in Northern Ireland) health data, for both clinical and research purposes. This survey is open to anyone living in the UK over 16 years of age (or under 16 with parental permission). If you are under 16, please show your parent/guardian this page and ask if they are happy for you to take this survey. You will need to confirm you have their permission before you start the survey.

What will taking part involve?

If you agree to take part, after filling in a consent form, you will be asked a series of questions about your views on sharing your NHS/HSC health data for both clinical and research purposes. By 'health data' we mean any information collected about a person's health including things like medical notes, blood test results, prescription information etc. collected by an appropriate health care professional. As well as asking about sharing health data, we will also ask you some questions about your physical and mental health and any health services you may have used, as well as some questions about you, for example your age and gender. The survey does not ask for any identifiable personal information, and at any time you may click the "prefer not to answer" box if you wish.

The survey has been tested and will take approximately 18-25 minutes to complete. The survey does not need to be completed at one sitting.

Your answers won't affect the way your own health information is managed.

What are the benefits or disadvantages of taking part in the survey?

There are no direct benefits or disadvantages to taking part in this survey nor are there any payments. However, the study team hopes the results of this survey can be used to inform and improve the way the NHS/HSC manage data for clinical and research purposes.

Do I have to take part?

No, taking part in this survey is entirely up to you. The consent form at the start of the survey asks for your permission to use your data, even if you decide not to complete all of the survey. Unfortunately, once data has been submitted it will not be possible for the study team to find and delete your answers as we are not asking for any personal details, making it impossible for us to retrieve and delete your individual answers.

How will the information I give be kept confidential? Where can I find out more about how your information is used?

No information that can identify you is being collected in this study. There is an option to leave an email address, should you wish to personally receive the results of the survey. Email addresses will be removed from the survey responses and stored on password protected computers at the University of Cambridge. Access to these email addresses will be by the study team only and will only be used to send you the final results of the survey. Once you have received the results you will be asked if you would like any further updates, for example any publications or posters that arise from the results of the study. If you decide you do not want any further information, we will delete your email address. If you would like further updates, we will keep your email address securely until we have sent you the final publications from the study, at which time point your email address will then be deleted from our records.

There is also an option to leave your postcode (or not) in the survey. The study team would like to look at whether there are any effects of geographical area on answers to the survey. Once the survey has closed the study team will convert all postcodes to a nationally available 'code' of the area you live in, and then delete your postcode. By doing this it will never be possible to identify you or the road that you live in, as one code is given to multiple postcodes in the same area. Whether or not you agree to give us your post code is entirely up to you.

Once the study team have finished using the de-identified data, they will make it freely available for public use via the University of Cambridge Research Data Repository (https://www.data.cam.ac.uk/repository). Please be assured that, as in the information above, any email addresses or postcodes given will have been deleted before this happens. There will be no way that anyone will be able to identify you from the data, but we hope that others may use the data to further understand the sharing of health data with the aim to improve mental and physical health.

You can find out more about how we use your information:

- at www.hra.nhs.uk/information-about-patients
- our leaflet from http://www.hra.nhs.uk/patientdataandresearch
- by sending an email to the University of Cambridge Data Protection officer at dpo@admin.cam.ac.uk
- by asking one of the research team

Who is conducting this survey?

This survey is being conducted by the CLIMB project study team based at the University of Cambridge in conjunction with a patient and public research advisory group. Funding for the study team is from the Medical Research Council's (MRC) Mental Health Data Pathfinder awards.

For peer review only - http://bmjopen.bmj.com/site/about/guidelines.xhtml

Who has reviewed this study?

This study has been reviewed and given a favourable opinion by members of the East of Scotland Research Ethics Service.

Where can I find the results of this survey?

Once the study has concluded and the results have been analysed, a report will be made available. If you have chosen to leave an email address this report will be sent directly to you. Results will also be published on the following websites: https://www.climbproject.org.uk/ and www.psychiatry.cam.ac.uk. You may like to note these down now before you move on or, alternatively, they will be displayed again at the end of the survey.

What if I have any concerns about this study?

The study team can be contacted in the following ways:

Mrs Linda Jones

Email: laj28@cam.ac.uk

Telephone (01223) 764670

Further contact details are available at: https://www.climbproject.org.uk/

Alternatively you may like to contact the CPFT's Patient Advice and Liaison Service (PALS) on 0800 376 0775 or email pals@cpft.nhs.uk. Please note PALS will not be able to give advice about technical queries with the study.

Thank you very much for reading the information sheet about the survey. If you would now like to take part in the survey, please read the following statements and tick the boxes to show that you agree. Please only take the survey one time (although you do not have to complete it in one go).

Page 1 of 29

Next Page >>

Save & Return Later

For peer review only - http://bmjopen.bmj.com/site/about/guidelines.xhtml

NHS Data Consent Survey

Resize font:

Page 2 of 29

Consent Form

Please tick ALL boxes

I confirm I am aged 16 or over, or I am under 16 years of age but have the consent of my parent/guardian. * must provide value	☑
I confirm that I am a resident of the UK. * must provide value	☑
I understand I may quit the survey at any time but any data I have provided may still be used. * must provide value	☑
I understand that the answers I give will be used for analysis and publication. * must provide value	☑
I understand my answers will be recorded in a way that means I cannot be identified. * must provide value	☑
I understand that not everyone will see the same questions or definitions. * must provide value	☑
If I have any problems or questions with the survey, I understand I can contact Linda Jones, e-mail: laj28@cam.ac.uk, telephone: 01223 764670 * must provide value	☑
If I choose to leave an email address, I understand it will be removed from the survey and only used to send me the results of the survey * must provide value	☑
I consent to take part in this survey. * must provide value	☑

We would like to know where you heard about this survey. It is especially important to know if you were given a leaflet (or letter) or saw a poster/text/social media from a 'healthcare provider' e.g. hospital, clinic, GP, ambulance service, healthcare community setting, or any other healthcare provider.

* must provide value

- ○ Prefer not to say
- ○ GP (via a poster, leaflet, from a staff member, via text, via GP Practice social media etc.)
- ● Hospital/Clinic/Ambulance/Healthcare community setting, other healthcare provider (via a poster, leaflet, from a staff member, text, healthcare provider's social media etc.)
- ○ Group to which I belong
- ○ Charity website/email/newsletter etc.
- ○ Social media (other than from a healthcare provider)
- ○ Poster (other than at a healthcare provider)
- ○ Browsing the internet
- ○ Friend
- ○ Not sure
- ○ Other

reset

For peer review only - http://bmjopen.bmj.com/site/about/guidelines.xhtml

Please could you tell us in which healthcare establishment you saw a poster or picked up/were given a leaflet? If you have the leaflet, the healthcare setting may be named on the back of it. This is really important to each healthcare provider. (Please type in a few letters and options will appear.)

* must provide value

Cambridgeshire and Peterborough NHS Foundation Trust (CPFT)

<< Previous Page

Next Page >>

Save & Return Later

For peer review only

NHS Data Consent Survey

Resize font:

Page 3 of 29

Before starting the survey please read the following definitions:

By "NHS" we mean all parts of the national health service in the UK: NHS England, NHS Scotland, NHS Wales, and Health and Social Care in Northern Ireland (HSC). We refer to all these as the NHS in this survey.

Health data means any information about a person's health such as might be collected by a nurse, doctor or other appropriate member of NHS staff. Health data includes things like your medical notes, blood test results, prescription information, scans, etc.

Identifiable health data means any health data (as described above) that can also identify a person -- such as any health data that also contains names, addresses, NHS numbers, etc.

Clinical care means providing health care directly to people -- for example, diagnosing illnesses or treating ill health.

We would like to know/gauge your understanding of how NHS clinical care providers currently share your identifiable health data for your clinical care, without asking you each time. If you are not sure, please have a "best guess" as to what you think might happen.

My understanding is that (please tick only one):

* must provide value

- My identifiable health data is currently shared freely between all NHS sites across the UK -- i.e. all acute hospital Trusts, my GP, all mental health Trusts, ambulance services, etc., in the whole UK.
- My identifiable health data is currently shared between all NHS sites but only in the home nation that I live in -- i.e. shared only in England, or Scotland, or Northern Ireland, or Wales, but not between them.
- My identifiable health data is currently only shared between NHS sites in my region (cluster of hospitals nearest to my local hospital).
- My identifiable health data is currently only shared between my GP, acute local hospital, local mental health Trust, and other local services.
- My identifiable health data is currently not shared between any NHS sites.

reset

<< Previous Page

Next Page >>

Save & Return Later

For peer review only - http://bmjopen.bmj.com/site/about/guidelines.xhtml

NHS Data Consent Survey

Resize font:

Page 4 of 29

MOST hospital Trusts, GPs, mental health Trusts are separate and currently DO NOT share identifiable health data without asking you, though they correspond and communicate with each other with your permission.

In view of the statement above, we would now like to ask you your views on sharing your own identifiable health data for YOUR CLINICAL CARE, without having to be asked each time (please tick only one):

* must provide value

- ○ I would like my identifiable health data to be shared for clinical purposes with any part of the NHS in the UK, without asking me first.
- ○ I would like my identifiable health data to be shared for clinical purposes with any part of the NHS in my home nation only (i.e. only in England, or only in Northern Ireland, or only in Scotland, or only in Wales) without asking me first.
- ○ I would like my identifiable health data to be shared for clinical purposes with any part of my region (cluster of hospitals nearest to my local hospital), without asking me first.
- ○ I would like my identifiable health data to be shared for clinical purposes only with my GP, local hospital, and local mental health services, without asking me first.
- ○ No one in the NHS should share my identifiable health data without asking me first.
- ○ Not sure.

reset

<< Previous Page

Next Page >>

Save & Return Later

For peer review only - http://bmjopen.bmj.com/site/about/guidelines.xhtml

NHS Data Consent Survey

Resize font:

Page 5 of 29

We would now like to ask you some questions about your own MENTAL and PHYSICAL health. You can "prefer not to say" if you wish.

We would like to ask if you have had a MENTAL health condition either recently or at some point in your life.

By mental health conditions we include things like delirium, self harm, substance abuse, dementia, mania, schizophrenia, depression, anxiety disorders, psychosis, eating disorders, OCD, personality disorders, etc.

* must provide value

- I prefer not to say.
- Yes, I have had a mental health condition either recently or at some point in my life.
- No, I have never had a mental health condition.

reset

<< Previous Page

Next Page >>

Save & Return Later

For peer review only - http://bmjopen.bmj.com/site/about/guidelines.xhtml

NHS Data Consent Survey

Resize font:

Page 6 of 29

We would like to know if you would be willing to share what mental health condition you have had.

You may tick as many boxes as you wish.

If you prefer not to say, please use the "next page" button below to move on.

	In the last 5 years	More than 5 years ago
Delirium	☐	☐
Alcohol or substance abuse	☐	☐
Schizophrenia, schizoaffective disorder, or delusional disorder	☐	☐
Dementia	☐	☐
Mania or bipolar affective disorder	☐	☐
Depression	☐	☐
Anxiety disorder (e.g. phobia, panic, generalized anxiety disorder, post-traumatic stress disorder)	☐	☐
Psychosis of any cause	☐	☐
Eating disorder (e.g. anorexia nervosa, bulimia)	☐	☐
Obsessive-compulsive disorder	☐	☐
Personality disorder	☐	☐
Self harm	☐	☐
Other	☐	☐

<< Previous Page

Next Page >>

Save & Return Later

For peer review only - http://bmjopen.bmj.com/site/about/guidelines.xhtml

NHS Data Consent Survey

Resize font:

Page 7 of 29

And also in relation to mental health:

We would also like to know if you have accessed any mental health services for your own mental health condition.

These might include your GP, online services, mental health forums, psychological therapy services, crisis care, home treatments or visits, outpatient or inpatient care, services from the private sector, support from a mental health charity, etc.

* must provide value

- ○ I prefer not to say.
- ● I have used mental health services.
- ○ I have not used any mental health services (not even my GP or surgery) for my mental health condition.

reset

	In the last 5 years	More than 5 years ago
**Mental health support from your GP or surgery**	☐	☐
**An NHS psychological therapy service, e.g. IAPT or other therapy service (INDIVIDUAL session)**	☐	☐
**An NHS psychological therapy service, e.g. IAPT or other therapy service (GROUP session)**	☐	☐
**An NHS psychological therapy service, e.g. IAPT or other therapy service (ONLINE)**	☐	☐
**NHS outpatient or community mental health services, or key worker**	☐	☐
**NHS crisis care/Home Treatment teams**	☐	☐
**NHS inpatient mental health services in a mental health hospital**	☐	☐
**NHS inpatient mental health services in a general hospital (liaison psychiatry)**	☐	☐
**Private services**	☐	☐
**Services in the workplace e.g. Occupational Health**	☐	☐
**Charity providing mental health support**	☐	☐
**Online forum**	☐	☐
**Other**	☐	☐

<< Previous Page

Next Page >>

Save & Return Later

For peer review only - http://bmjopen.bmj.com/site/about/guidelines.xhtml

NHS Data Consent Survey

Resize font:

Page 8 of 29

We are also interested to know if you have accessed any services regarding your own PHYSICAL health.

This could be services like your GP, outpatient hospital appointments, inpatient services, online support, Accident and Emergency, 111 service, private services, charities providing physical health support, etc.

Have you ever used any of the following services for your own PHYSICAL health?

* must provide value

- ○ I prefer not to say
- ◉ I have used physical health services (this includes using your GP)
- ○ I have not used any physical health services

reset

<< Previous Page

Next Page >>

Save & Return Later

For peer review only - http://bmjopen.bmj.com/site/about/guidelines.xhtml

NHS Data Consent Survey

Resize font: ⊞ | ⊟

Page 9 of 29

And finally in this section, we would like to know if you would be willing to share what physical health services you have used for your own physical health.

You may tick as many boxes as you wish.

Please use the 'next page' button if you prefer not to answer.

	In the last 5 years	More than 5 years ago
Physical health support from your GP or surgery	☐	☐
NHS hospital outpatient services (outpatient clinics etc.)	☐	☐
NHS Urgent Care Centre (not A&E) or NHS 111 helpline	☐	☐
NHS Accident and Emergency services (A&E)	☐	☐
NHS inpatient services (procedures not completed in 1 day)	☐	☐
NHS Day Surgery/day care in a hospital	☐	☐
Private services	☐	☐
Services in the workplace e.g. Occupational Health	☐	☐
Charity providing physical health support	☐	☐
Online forum	☐	☐
Other	☐	☐

<< Previous Page

Next Page >>

Save & Return Later

For peer review only - http://bmjopen.bmj.com/site/about/guidelines.xhtml

NHS Data Consent Survey

Resize font:

Page 10 of 29

Moving on...

The table below shows what NHS identifiable health data may look like. This sort of data is used for health care by NHS staff. (Please note the examples shown are not real.)

NHS number	Title	Forename	Surname	Date of birth	Address	Diagnoses	Notes
9876543210	Mr	John	Smith	20 Jan 1950	1 The Street, Maidstone	pneumonia<br>high blood pressure	1 Apr 2019. Seen in clinic. Mr Smith has a fever and a cough. His chest sounds crackly. I think he has pneumonia. Sent to hospital.
8765432109	Miss	Alice	Jones	16 Jun 1994	15 The Road, Dundee	depression<br>carpal tunnel syndrome	2 Apr 2019. Alice rates her mood as 1/10 on average, she is more tired, and she is not looking forward to anything. Her depression has worsened. She wishes to restart citalopram.
7654321098	Mrs	Chloe	Williams	4 May 1930	5 Tree Close, Cardiff	broken humerus<br>anxiety	3 Apr 2019. Chloe fell after skidding on an oily patch while cycling and was hit by a car. Has an obvious fracture of her left upper arm. Needs X-ray.
6543210987	Mr	Pradeep	Agarwal	22 Sep 1973	27 The Mews, London	schizophrenia<br>diabetes	4 Apr 2019. Pradeep still worries that other people can hear his thoughts, but this is getting less common. He is attending cognitive–behavioural therapy sessions.

<< Previous Page

Next Page >>

Save & Return Later

For peer review only - http://bmjopen.bmj.com/site/about/guidelines.xhtml

NHS Data Consent Survey

Resize font:

Page 11 of 29

Previous surveys have found that people have more concerns about the use of their identifiable health data relating to their mental health than other aspects of their physical health care.

Regardless of whether you have a PHYSICAL health condition or have used PHYSICAL health services:

How likely are you to agree to share your identifiable PHYSICAL health data for your clinical care with the following, without being asked every time?

	Very likely	Likely	Not sure	Unlikely	Very unlikely	Prefer not to say
**Between my local NHS services -- i.e. between my local physical and mental health hospitals, my GP, etc.** * must provide value	○	○	○	○	○	○
**Any part of the NHS in my region (cluster of hospitals nearest to my local hospital)** * must provide value	○	○	○	○	○	○
**Any part of the NHS in my home nation only (i.e. only in England, or only in Northern Ireland, or only in Scotland, or only in Wales)** * must provide value	○	○	○	○	○	○
**Any part of the NHS in the UK** * must provide value	○	○	○	○	○	○

reset

<< Previous Page

Next Page >>

Save & Return Later

For peer review only - http://bmjopen.bmj.com/site/about/guidelines.xhtml

NHS Data Consent Survey

Resize font:

Page 14 of 29

Regardless of whether you have a MENTAL health condition or have used MENTAL health services:

How likely are you to agree to share your identifiable MENTAL health data for your clinical care with the following, without being asked every time?

	Very likely	Likely	Not sure	Unlikely	Very unlikely	Prefer not to say
**Between my local NHS services -- i.e. between my local physical and mental health hospitals, my GP, etc.** * must provide value	○	○	○	○	○	○ reset
**Any part of the NHS in my region (cluster of hospitals nearest to my local hospital)** * must provide value	○	○	○	○	○	○ reset
**Any part of the NHS in my home nation only (i.e. only in England, or only in Northern Ireland, or only in Scotland, or only in Wales)** * must provide value	○	○	○	○	○	○ reset
**Any part of the NHS in the UK** * must provide value	○	○	○	○	○	○ reset

<< Previous Page

Next Page >>

Save & Return Later

For peer review only - http://bmjopen.bmj.com/site/about/guidelines.xhtml

NHS Data Consent Survey

Resize font:

Page 15 of 29

In the next two questions we would like to ask you about sharing your NHS data for research purposes. Research is used to improve overall healthcare for everyone.

Firstly, please look at some of the NHS health care data that you saw previously, which has now been de-identified.

Research ID	Age	Sex	Region	Diagnoses
5X62V	69	M	Kent	pneumonia<br>high blood pressure
597PT	24	F	Dundee	depression<br>carpal tunnel syndrome
8HG7S	89	F	Glamorganshire	broken humerus<br>anxiety
BA6A9	45	M	London	schizophrenia<br>diabetes

<< Previous Page

Next Page >>

Save & Return Later

For peer review only - http://bmjopen.bmj.com/site/about/guidelines.xhtml

NHS Data Consent Survey

Resize font:

Page 16 of 29

How likely would you be to share your de-identified PHYSICAL health data with the following, without giving consent every time?

	Very likely	Likely	Not sure	Unlikely	Very unlikely	Prefer not to say
**Any part of the NHS doing research** * must provide value						reset
**Academic institutions doing research (e.g. universities)** * must provide value						reset
**A national charity doing research (e.g. MIND, Cancer Research UK, British Heart Foundation)** * must provide value						reset
**A profit-making company doing research into treatments (e.g. a pharmaceutical company, health technology company)** * must provide value						reset
**A profit-making company doing other research (e.g. an insurance company, broadband provider)** * must provide value						reset
**Publicly** * must provide value						reset

<< Previous Page

Next Page >>

Save & Return Later

For peer review only - http://bmjopen.bmj.com/site/about/guidelines.xhtml

Resize font:

NHS Data Consent Survey

Page 17 of 29

How likely would you be to share your de-identified MENTAL health data with the following, without giving consent every time:

	Very likely	Likely	Not sure	Unlikely	Very unlikely	Prefer not to say
**Any part of the NHS doing research** * must provide value	○	○	○	○	○	○ reset
**Academic institutions doing research (e.g. universities)** * must provide value	○	○	○	○	○	○ reset
**A national charity doing research (e.g. MIND, Cancer Research UK, British Heart Foundation)** * must provide value	○	○	○	○	○	○ reset
**A profit-making company doing research into treatments (e.g. pharmaceutical company, health technology company)** * must provide value	○	○	○	○	○	○ reset
**A profit-making company doing other research (e.g. insurance company, broadband provider)** * must provide value	○	○	○	○	○	○ reset
**Publicly** * must provide value	○	○	○	○	○	○ reset

<< Previous Page

Next Page >>

Save & Return Later

For peer review only - http://bmjopen.bmj.com/site/about/guidelines.xhtml

NHS Data Consent Survey

Resize font:

Page 18 of 29

The final question in this section relates to sharing de-identified "free text" data for research.

Here is the table of de-identified health care data you saw earlier, but this time a column labelled "Notes" has been added. This is known as "free text" data.

Research ID	Age	Sex	Region	Diagnoses	Notes
5X62V	69	M	Kent	pneumonia<br>high blood pressure	1st April 2019. XXX has a fever and cough. His chest sounds crackly. I think he has pneumonia. Sent to hospital.
597PT	24	F	Dundee	depression<br>carpal tunnel syndrome	2nd April 2019. XXX rates her mood as 1/10 on average, she is more tired, and she is not looking forward to anything. Her depression has worsened. She wishes to restart citalopram.
8HG7S	89	F	Glamorganshire	broken humerus<br>anxiety	3rd April 2019. XXX fell after skidding on an oily patch while cycling and was hit by a car. She has an obvious fracture of her left upper arm. She needs an X-ray.
BA6A9	45	M	London	schizophrenia<br>diabetes	4th April 2019. XXX still worries that other people can hear his thoughts, but this is getting less common. He is attending cognitive-behavioural therapy sessions.

Like the last example the data remains de-identified but gives the researchers more information. As there is more information there is a slightly increased risk of someone being identified from the data.

For example, in the table above, an "89-year-old cyclist skidding on oil and being hit by a car" may be reported in a local newspaper. Whilst the lady is not named in the data above, she would almost certainly be named in the newspaper and it may therefore be possible for researchers to work out who she is.

<< Previous Page

Next Page >>

Save & Return Later

For peer review only - http://bmjopen.bmj.com/site/about/guidelines.xhtml

NHS Data Consent Survey

Resize font:

Page 19 of 29

How likely would you be to share your FREE TEXT de-identified PHYSICAL health data with the following, without giving consent every time:

	Very likely	Likely	Not sure	Unlikely	Very unlikely	Prefer not to say	
**Any part of the NHS doing research** * must provide value	○	○	○	○	●	○	reset
**Academic institutions doing research (e.g. universities)** * must provide value	○	○	○	○	●	○	reset
**A national charity doing research (e.g MIND, Cancer Research UK, British Heart Foundation)** * must provide value	○	○	○	○	●	○	reset
**A profit-making company doing research into treatments (e.g. pharmaceutical company, health technology company)** * must provide value	○	○	○	○	●	○	reset
**A profit-making company doing other research (e.g. insurance company, broadband provider)** * must provide value	○	○	○	○	●	○	reset
**Publicly** * must provide value	○	○	○	○	●	○	reset

<< Previous Page

Next Page >>

Save & Return Later

For peer review only - http://bmjopen.bmj.com/site/about/guidelines.xhtml

NHS Data Consent Survey

Resize font:

Page 20 of 29

How likely would you be to share your FREE TEXT de-identified MENTAL health data with the following, without giving consent each time:

	Very likely	Likely	Not sure	Unlikely	Very unlikely	Prefer not to say	
**Any part of the NHS doing research** * must provide value	○	○	○	○	○	○	reset
**Academic institutions doing research (e.g. universities)** * must provide value	○	○	○	○	○	○	reset
**A national charity doing research (e.g MIND, Cancer Research UK, British Heart Foundation)** * must provide value	○	○	○	○	○	○	reset
**A profit-making company doing research into treatments (e.g. pharmaceutical company, health technology company)** * must provide value	○	○	○	○	○	○	reset
**A profit-making company doing other research (e.g. insurance company, broadband provider)** * must provide value	○	○	○	○	○	○	reset
**Publicly** * must provide value	○	○	○	○	○	○	reset

<< Previous Page

Next Page >>

Save & Return Later

For peer review only - http://bmjopen.bmj.com/site/about/guidelines.xhtml

NHS Data Consent Survey

Resize font:

Page 21 of 29

On a slightly different topic...

Imagine that there was a single place where you could securely sign up to choose how your NHS identifiable health data is used. This would cover how your confidential patient information is managed across all UK NHS services, and how your NHS de-identified data could (or could not) be used for research.

It would be in the form of a standardised UK-wide CONSENT FORM that all NHS services could use.

How likely would you be to sign up if you were asked to sign:

	Very likely	Likely	Not sure	Unlikely	Very unlikely	Prefer not to say
**Online (via a website)** * must provide value	○	○	○	○	○	○ reset
**By downloading an app** * must provide value	○	○	○	○	○	○ reset
**On paper forms available from pharmacies, the Post Office, etc. (and sent by post)** * must provide value	○	○	○	○	○	○ reset
**In person (e.g. when attending your GP, at a hospital clinic, etc.)** * must provide value	○	○	○	○	○	○ reset

<< Previous Page

Next Page >>

Save & Return Later

For peer review only - http://bmjopen.bmj.com/site/about/guidelines.xhtml

NHS Data Consent Survey

Resize font:

Page 22 of 29

Would you be likely to sign up if your consent form were then stored and managed securely by the following institutions?

	Very likely	Likely	Not sure	Unlikely	Very unlikely	Prefer not to say
**Centrally by NHS England/NHS Scotland/NHS Wales/HSC Northern Ireland** * must provide value	○	○	○	○	○	○
**Your local NHS Trust** * must provide value	○	○	○	○	○	○
**Your GP** * must provide value	○	○	○	○	○	○

reset

<< Previous Page

Next Page >>

Save & Return Later

For peer review only - http://bmjopen.bmj.com/site/about/guidelines.xhtml

NHS Data Consent Survey

Resize font:

Page 23 of 29

If you wanted to change your preferences, how likely would you be to use the following methods?

	Very likely	Likely	Not sure	Unlikely	Very unlikely	Prefer not to say
**Online (via a website)** * must provide value	○	○	○	○	○	○ reset
**By downloading an app** * must provide value	○	○	○	○	○	○ reset
**On paper forms available from pharmacies, Post Office, etc. (and sent by post)** * must provide value	○	○	○	○	○	○ reset
**In person (e.g. when attending your GP, at a hospital clinic, etc.)** * must provide value	○	○	○	○	○	○ reset

<< Previous Page

Next Page >>

Save & Return Later

For peer review only - http://bmjopen.bmj.com/site/about/guidelines.xhtml

Resize font:

NHS Data Consent Survey

Page 24 of 29

Assume that you were able to sign up and change your choices in a way you preferred, and that the consent form was stored securely in a place where you felt comfortable.

How likely would you be to use this system to choose how your NHS health data is managed?

	Very likely	Likely	Not sure	Unlikely	Very unlikely	Prefer not to say
**Overall likelihood that you would sign up** * must provide value	○	○	○	○	○	○

reset

<< Previous Page

Next Page >>

Save & Return Later

For peer review only - http://bmjopen.bmj.com/site/about/guidelines.xhtml

NHS Data Consent Survey

Resize font:

Page 25 of 29

The last part of the survey (before we ask a few questions to ensure we have reached a broad section of the UK) examines what a consent form to share our health data could look like.

By completing one consent form it would then be possible for all UK NHS professionals to access their patient's identifiable health data for their clinical care, saving time for both patients and health care professionals.

Please take a look at the consent form below:

National NHS/HSC data sharing consent form

HSC Health and Social Care NHS

Full name ____________________
Date of birth __________
NHS number ____________________
Address ______________________________
____
E-mail address ____________________

I confirm I have read the information sheet 'Sharing My Health Data' version XXX dated XXX. I have had the opportunity to consider the information and ask questions. I understand that my participation is voluntary, and that I am free to change or withdraw my consent at any time, without giving a reason and without my medical care or rights being affected. Yes [ ]

1. Providing health care to you

Confidential patient information is information that can identify you and that says something about your health care or treatment. Information about you might be held by several NHS organizations (such as GP surgeries and hospitals). May they share your information with each other when providing health care to you?

I agree that all NHS care providers and professionals may share my confidential patient information with each other for the purpose of my treatment and care. Yes [ ] No [ ]

2. Using your de-identified data to help others

The NHS promises to **anonymise** the information collected during the course of your treatment and use it to support research and improve care for others. Research is conducted by the NHS and by NHS-approved researchers in organizations such as universities. Strict security controls apply to the use of NHS data, even after information that might identify you (such as names and addresses) has been removed. All research involving NHS data must be approved by the NHS.

Saying no doesn't prevent all uses of your confidential information for research. To do that, use the national data opt-out at https://www.nhs.uk/your-nhs-data-matters.

I agree that all NHS care providers may share my confidential patient information with each other and de-identify it for the purpose of research. Yes [ ] No [ ]

3. Taking part in research

Some research involves **direct participation.** This ranges from questionnaires to trials of new treatments. The NHS promises to inform you of research studies in which you may be eligible to participate. There is never a commitment to take part.

I agree that NHS-approved researchers may learn my identity and contact me directly about research studies for which I may be eligible. Yes [ ] No [ ]

Signature: ____________________

Date: __________

For peer review only - http://bmjopen.bmj.com/site/about/guidelines.xhtml

<< Previous Page

Next Page >>

Save & Return Later

For peer review only

NHS Data Consent Survey

Resize font:

Page 26 of 29

Having looked at the consent form, please agree or disagree with the following statements.

(If you would like to take another look at the consent form at any time while answering this question, you can use the 'previous' button at the bottom of this page to look back. This will not affect answers already given.)

	Strongly agree	Agree	Neither agree nor disagree	Disagree	Strongly disagree	Prefer not to say
The consent form is easy to read. * must provide value	○	○	○	○	○	○ reset
The consent form is clear and easy to understand. * must provide value	○	○	○	○	○	○ reset
The statements (in blue) help me to understand what I am consenting to. * must provide value	○	○	○	○	○	○ reset
The consent form is clear that my identifiable health data would only be used by appropriate NHS health care professionals. * must provide value	○	○	○	○	○	○ reset
I understand the term 'NHS-approved researchers' and which individuals this may refer to. * must provide value	○	○	○	○	○	○ reset
The consent form makes clear that it would be used for my preferences about sharing my IDENTIFIABLE health data within the NHS for my CLINICAL CARE. * must provide value	○	○	○	○	○	○ reset
The consent form makes clear that it would also be used for my preferences about sharing my DE-IDENTIFIED health data for RESEARCH purposes. * must provide value	○	○	○	○	○	○ reset
I would like the consent form to give specific options about how researchers can contact me (e.g. by post only, e-mail). * must provide value	○	○	○	○	○	○ reset
I would like to see a statement added about the security of data sharing. * must provide value	○	○	○	○	○	○ reset
I would like to see a statement added about where my consent preferences will be stored. * must provide value	○	○	○	○	○	○ reset
Before seeing this consent form I was aware of the NHS National Data Opt-Out. * must provide value	○	○	○	○	○	○ reset
The consent form reassures me that my identifiable health data is safe in the NHS. * must provide value	○	○	○	○	○	○ reset
If this consent form were put in front of me today, I would sign it. * must provide value	○	○	○	○	○	○ reset

If you would like to make any comments about the consent form, please do so here:

Expand

<< Previous Page

Next Page >>

Save & Return Later

For peer review only

For peer review only - http://bmjopen.bmj.com/site/about/guidelines.xhtml

NHS Data Consent Survey

Resize font:

Page 27 of 29

There are currently many individual websites where people can sign up to take part in research. Examples include charity websites, the national "Join Dementia Research" register, and the National Institute for Health Research (NIHR) "be part of research" campaign.

An alternative might be a national sign-up portal, on a web site, where people could register their preferences about taking part in all NHS research.

Choose the statement you agree with most:

* must provide value

- Everything is fine as it is; leave it to individuals to sign up with the various organisations.
- Have two national NHS research websites, one where people can sign up for mental health research, and a second website where people can sign up for physical health research.
- Have a national NHS research website where people can sign up for all conditions.
- Not sure.

reset

<< Previous Page

Next Page >>

Save & Return Later

For peer review only - http://bmjopen.bmj.com/site/about/guidelines.xhtml

NHS Data Consent Survey

Resize font:

Page 28 of 29

Finally, it can be very valuable for research to link health (NHS) data to other data sources.

For example, causes of death (from death certificates) are held by the UK Office of National Statistics (ONS), rather than the NHS -- so to find out more about the reasons people die, NHS and ONS data must be linked.

Usually, this is done as follows:
a) Research teams seek special permission for the process.
b) The relevant information from each organization, plus a small amount of identifiable information, is given to a "trusted third party" (e.g. an NHS Trust, the Office of National Statistics).
c) The trusted third party links the information, then removes any details that can identify anyone.
d) Researchers are then given access to the de-identified information only, under special controls.

We are interested to hear whether you would be happy for your health data to be linked, in this way, to:

	Yes	Not sure	No	Prefer not to say	
**Educational data (e.g. to study impact of illness on education)** * must provide value	○	○	○	○	reset
**Police/Criminal Justice data (e.g. to study health in the victims of crime)** * must provide value	○	○	○	○	reset
**Transport/DVLA data (e.g. to study health and pollution)** * must provide value	○	○	○	○	reset
**Housing data (e.g. impact of social housing on health)** * must provide value	○	○	○	○	reset
**Immigration data (e.g. health in immigrants)** * must provide value	○	○	○	○	reset
**Social security/Work and Pensions (e.g. health and financial insecurity)** * must provide value	○	○	○	○	reset
**Data held about you for research by universities (e.g. if you have volunteered for research studies)** * must provide value	○	○	○	○	reset
**Data held about you by private companies** * must provide value	○	○	○	○	reset

<< Previous Page

Next Page >>

Save & Return Later

For peer review only - http://bmjopen.bmj.com/site/about/guidelines.xhtml

NHS Data Consent Survey

Resize font:

Page 29 of 29

And finally, to ensure we have surveyed a wide range of the population (please note, all questions have a "prefer not to say" option and have been written to apply to all of the home nations):

Do you consider yourself to be:

* must provide value

- Prefer not to say
- Female
- Male
- Prefer to self describe

reset

May we know which age range you fit in?

* must provide value

- Prefer not to say
- Under 12
- 12-15
- 16-17
- 18-24
- 25-34
- 35-44
- 45-54
- 55-64
- 65-74
- 75-84
- 85+

reset

What do you consider your ethnicity to be?

* must provide value

- Prefer not to say
- White
- Irish Traveller
- Mixed/Multiple ethnic groups
- Asian or Asian British
- Indian
- Pakistani
- Bangladeshi
- Chinese
- Black/African/Caribbean/Black British
- Arab
- Other or prefer to self describe

reset

For peer review only - http://bmjopen.bmj.com/site/about/guidelines.xhtml

What is the highest qualification you have achieved?

* must provide value

- Prefer not to say
- No formal qualifications
- Secondary school level qualifications e.g. CSE, GCSE, O-Levels, Nationals, or equivalent
- A-Levels, Highers, or equivalent
- Vocational qualification or equivalent
- Undergraduate degree or equivalent
- Postgraduate or professional qualification or equivalent

reset

Do you consider yourself to be

* must provide value

- Prefer not to say
- Heterosexual (straight)
- Homosexual (gay or lesbian)
- Bisexual
- Other or prefer to self describe

reset

What is your religion, if any?

* must provide value

- Prefer not to say
- No religion
- Christian
- Muslim
- Hindu
- Sikh
- Jewish
- Buddhist
- Other or prefer to self describe

reset

Thinking about your current (or last) main job or occupation. Do (did) you work as an employee or are (were) you self-employed?

* must provide value

- Prefer not to say
- Never worked
- Employee
- Self-employed with employees
- Self-employed/freelance without employees

reset

And where in the UK do you currently live?

* must provide value

- Prefer not to say
- England
- Northern Ireland
- Scotland
- Wales
- Channel Islands
- Isle of Man
- None of these

reset

For peer review only - http://bmjopen.bmj.com/site/about/guidelines.xhtml

We would like to know your postcode so that we can analyse our results by "geography", such as urban versus rural areas. You do not have to give this. However if you do, we will never disclose it or identify you from it.

* must provide value

- I prefer not to give my postcode
- I am happy to give my postcode

reset

Before you submit your answers there is the option to leave an e-mail address should you wish to have the final report of the survey results personally e-mailed to you.

Please note: all e-mail addresses will be removed from the survey answers so that you cannot be identified, and will be held securely on password-protected computers at the University of Cambridge until the results are available, as described in the information sheet at the start of the survey.

If you wish to leave an e-mail address, please do so in the box below (and then submit your answers). Otherwise, please submit your answers now.

Optional

<< Previous Page

Submit

Save & Return Later

For peer review only

NHS Data Consent Survey

Resize font:

Page 29 of 29

And finally, to ensure we have surveyed a wide range of the population (please note, all questions have a "prefer not to say" option and have been written to apply to all of the home nations):

Do you consider yourself to be:

* must provide value

- ( ) Prefer not to say
- ( ) Female
- ( ) Male
- (•) Prefer to self describe

reset

Prefer to self describe (please specify)

May we know which age range you fit in?

* must provide value

- ( ) Prefer not to say
- (•) Under 12
- ( ) 12-15
- ( ) 16-17
- ( ) 18-24
- ( ) 25-34
- ( ) 35-44
- ( ) 45-54
- ( ) 55-64
- ( ) 65-74
- ( ) 75-84
- ( ) 85+

reset

Please tick which of the two options applies:

* must provide value

- ( ) I completed the survey by myself
- ( ) I had help to complete the survey

reset

What do you consider your ethnicity to be?

* must provide value

- ( ) Prefer not to say
- ( ) White
- ( ) Irish Traveller
- ( ) Mixed/Multiple ethnic groups
- ( ) Asian or Asian British
- ( ) Indian
- ( ) Pakistani
- ( ) Bangladeshi
- ( ) Chinese
- ( ) Black/African/Caribbean/Black British
- ( ) Arab
- (•) Other or prefer to self describe

reset

For peer review only - http://bmjopen.bmj.com/site/about/guidelines.xhtml

Other or prefer to self describe (please specify)

What is the highest qualification you have achieved?

* must provide value

- ○ Prefer not to say
- ○ No formal qualifications
- ○ Secondary school level qualifications e.g. CSE, GCSE, O-Levels, Nationals, or equivalent
- ○ A-Levels, Highers, or equivalent
- ○ Vocational qualification or equivalent
- ○ Undergraduate degree or equivalent
- ○ Postgraduate or professional qualification or equivalent

reset

Do you consider yourself to be

* must provide value

- ○ Prefer not to say
- ○ Heterosexual (straight)
- ○ Homosexual (gay or lesbian)
- ○ Bisexual
- ◉ Other or prefer to self describe

reset

Other or prefer to self describe (Please specify)

What is your religion, if any?

* must provide value

- ○ Prefer not to say
- ○ No religion
- ○ Christian
- ○ Muslim
- ○ Hindu
- ○ Sikh
- ○ Jewish
- ○ Buddhist
- ◉ Other or prefer to self describe

reset

Other or prefer to self describe (please specify)

Thinking about your current (or last) main job or occupation. Do (did) you work as an employee or are (were) you self-employed?

* must provide value

- ○ Prefer not to say
- ○ Never worked
- ◉ Employee
- ○ Self-employed with employees
- ○ Self-employed/freelance without employees

reset

How many people work (worked) for your employer at the place where you work (worked)?

- ◯ Prefer not to say
- ◯ 1-24
- ◉ 25 or more

reset

Do (did) you supervise any other employees on a day to day basis?

- ◯ Prefer not to say
- ◉ Yes
- ◯ No

reset

And which best describes the work you do (did)?

- ◯ Prefer not to say
- ◯ Modern professional occupations such as: teacher - nurse - physiotherapist - social worker - welfare officer - artist - musician - police office (sergeant or above) - software designer
- ◯ Clerical and intermediate occupations such as: secretary - personal assistant - clerical worker - office clerk - call centre agent - nursing auxiliary - nursery nurse
- ◯ Senior managers or administrators (responsible for planning, organising, and co-coordinating work and for finance) such as: finance manager - chief executive
- ◯ Technical and craft occupations such as: motor mechanic - fitter - inspector - plumber - printer - tool maker - electrician - gardener - train driver
- ◯ Semi-routine manual and service occupations such as: postal worker - machine operative - security guard - caretaker - farm worker - catering assistant - receptionist - sales assistant
- ◯ Routine manual and service occupations such as: HGV driver - van driver - cleaner - porter - packer - sewing machinist - messenger - labourer - waiter/waitress - bar staff
- ◯ Middle or junior managers such as: office manager - retail manager - bank manager - restaurant manager - warehouse manager - publican
- ◯ Traditional professional occupations such as: accountant - solicitor - medical practitioner - scientist - civil/mechanical engineer

reset

And where in the UK do you currently live?

* must provide value

- ◯ Prefer not to say
- ◉ England
- ◯ Northern Ireland
- ◯ Scotland
- ◯ Wales
- ◯ Channel Islands
- ◯ Isle of Man
- ◯ None of these

reset

Please specify the region you live in

* must provide value

- ○ Prefer not to say
- ○ North East
- ○ North West
- ○ Yorkshire and Humber
- ○ West Midlands
- ○ East Midlands
- ○ East of England
- ○ London
- ○ South East
- ○ South West
- ◉ Other

reset

Other (please specify)

We would like to know your postcode so that we can analyse our results by "geography", such as urban versus rural areas. You do not have to give this. However if you do, we will never disclose it or identify you from it.

* must provide value

- ○ I prefer not to give my postcode
- ◉ I am happy to give my postcode

reset

Postcode

Before you submit your answers there is the option to leave an e-mail address should you wish to have the final report of the survey results personally e-mailed to you.

Please note: all e-mail addresses will be removed from the survey answers so that you cannot be identified, and will be held securely on password-protected computers at the University of Cambridge until the results are available, as described in the information sheet at the start of the survey.

If you wish to leave an e-mail address, please do so in the box below (and then submit your answers). Otherwise, please submit your answers now.

Optional

<< Previous Page

Submit

Save & Return Later

For peer review only - http://bmjopen.bmj.com/site/about/guidelines.xhtml

STROBE Statement—Checklist of items that should be included in reports of ***cross-sectional studies***

Jones et al.: Public opinion on sharing data from UK health services for clinical and research purposes without explicit consent, 2021

	Item No	Recommendation	Section (§, main text section; S, supplementary; F, figure; T, table)	Page number in submitted manuscript (S, supplementary)
**Title and abstract**	1	(*a*) Indicate the study's design with a commonly used term in the title or the abstract	§1	2
		(*b*) Provide in the abstract an informative and balanced summary of what was done and what was found	§1	2
**Introduction**				
Background/ rationale	2	Explain the scientific background and rationale for the investigation being reported	§3	3
Objectives	3	State specific objectives, including any prespecified hypotheses	§3	3
**Methods**				
Study design	4	Present key elements of study design early in the paper	§3, §4	3–4
Setting	5	Describe the setting, locations, and relevant dates, including periods of recruitment, exposure, follow-up, and data collection	§4.3–4.5	4
Participants	6	(*a*) Give the eligibility criteria, and the sources and methods of selection of participants	§4.3	4
Variables	7	Clearly define all outcomes, exposures, predictors, potential confounders, and effect modifiers. Give diagnostic criteria, if applicable	§4.5–4.9, S1.2	4–5, S4
Data sources/ measurement	8*	For each variable of interest, give sources of data and details of methods of assessment (measurement). Describe comparability of assessment methods if there is more than one group	§4.5	5–6
Bias	9	Describe any efforts to address potential sources of bias	§4.9, S1.3	5, S4
Study size	10	Explain how the study size was arrived at	§4.3	4
Quantitative	11	Explain how quantitative variables were handled	§4.9, S1.2–S1.4	5–6, S4–S5

For peer review only - http://bmjopen.bmj.com/site/about/guidelines.xhtml

variables		in the analyses. If applicable, describe which groupings were chosen and why		
Statistical methods	12	(*a*) Describe all statistical methods, including those used to control for confounding	§4.9, S1.3–S1.4	5–6, S4–S5
		(*b*) Describe any methods used to examine subgroups and interactions	§4.9, S1.4	5–6, S4–S5
		(*c*) Explain how missing data were addressed	§4.9	5–6
		(*d*) If applicable, describe analytical methods taking account of sampling strategy	§4.8–4.9, S1.3–S1.4	5–6, S4–S5
		(*e*) Describe any sensitivity analyses	S1.3, ST1	S4, S12
**Results**				
Participants	13*	(a) Report numbers of individuals at each stage of study—eg numbers potentially eligible, examined for eligibility, confirmed eligible, included in the study, completing follow-up, and analysed	§5.1, S2.5, SF1	6, S6, S14/S15
		(b) Give reasons for non-participation at each stage	SF1	S14/S15
		(c) Consider use of a flow diagram	SF1	S14/S15
Descriptive data	14*	(a) Give characteristics of study participants (eg demographic, clinical, social) and information on exposures and potential confounders	§5.1, SF2, SF3, ST1	6, S14/S16, S14/S17, S12
		(b) Indicate number of participants with missing data for each variable of interest	F2–F4, F6–F7, SF1–SF7	Figures (as left); S14–S21
Outcome data	15*	Report numbers of outcome events or summary measures	N/A	N/A
Main results	16	(*a*) Give unadjusted estimates and, if applicable, confounder-adjusted estimates and their precision (eg, 95% confidence interval). Make clear which confounders were adjusted for and why they were included	F2–F7, SF4–SF7	Figures (as left); S14/S18–S21
		(*b*) Report category boundaries when continuous variables were categorized	S1.2	S2
		(*c*) If relevant, consider translating estimates of relative risk into absolute risk for a meaningful time period	N/A	N/A
Other analyses	17	Report other analyses done—eg analyses of subgroups and interactions, and sensitivity	§4.9, §5.4–5.7, S1.4, S2.2–S2.10,	5–8, S4–S8, S12,

For peer review only - http://bmjopen.bmj.com/site/about/guidelines.xhtml

		analyses	ST1, SF4–SF7	S14/S18–S21.
**Discussion**				
Key results	18	Summarise key results with reference to study objectives	§6.1	9
Limitations	19	Discuss limitations of the study, taking into account sources of potential bias or imprecision. Discuss both direction and magnitude of any potential bias	§6.2	9
Interpretation	20	Give a cautious overall interpretation of results considering objectives, limitations, multiplicity of analyses, results from similar studies, and other relevant evidence	§6.3–6.6	10–11
Generalisability	21	Discuss the generalisability (external validity) of the study results	§6.2	9
**Other information**				
Funding	22	Give the source of funding and the role of the funders for the present study and, if applicable, for the original study on which the present article is based	§10	12

*Give information separately for exposed and unexposed groups.

Note: An Explanation and Elaboration article discusses each checklist item and gives methodological background and published examples of transparent reporting. The STROBE checklist is best used in conjunction with this article (freely available on the Web sites of PLoS Medicine at http://www.plosmedicine.org/, Annals of Internal Medicine at http://www.annals.org/, and Epidemiology at http://www.epidem.com/). Information on the STROBE Initiative is available at www.strobe-statement.org.

For peer review only

For peer review only - http://bmjopen.bmj.com/site/about/guidelines.xhtml

BMJ Open

Public opinion on sharing data from health services for clinical and research purposes without explicit consent: an anonymous online survey in the UK

Journal:	*BMJ Open*
Manuscript ID	bmjopen-2021-057579.R1
Article Type:	Original research
Date Submitted by the Author:	15-Feb-2022
Complete List of Authors:	Jones, Linda; University of Cambridge, Department of Psychiatry<br>Nelder, Jenny; University of Cambridge, Department of Psychiatry<br>Fryer, Joseph; University of Cambridge, Department of Psychiatry<br>Alsop, Philip; Unaffiliated<br>Geary, Michael; Unaffiliated<br>Prince, Mark; Unaffiliated<br>Cardinal, Rudolf; University of Cambridge, Department of Psychiatry; Cambridgeshire and Peterborough NHS Foundation Trust, Liaison Psychiatry Service
**Primary Subject Heading**:	Health policy
Secondary Subject Heading:	Health policy, Mental health
Keywords:	Information management < BIOTECHNOLOGY & BIOINFORMATICS, Health informatics < BIOTECHNOLOGY & BIOINFORMATICS, Health policy < HEALTH SERVICES ADMINISTRATION & MANAGEMENT, MENTAL HEALTH

SCHOLARONE™
Manuscripts

I, the Submitting Author has the right to grant and does grant on behalf of all authors of the Work (as defined in the below author licence), an exclusive licence and/or a non-exclusive licence for contributions from authors who are: i) UK Crown employees; ii) where BMJ has agreed a CC-BY licence shall apply, and/or iii) in accordance with the terms applicable for US Federal Government officers or employees acting as part of their official duties; on a worldwide, perpetual, irrevocable, royalty-free basis to BMJ Publishing Group Ltd ("BMJ") its licensees and where the relevant Journal is co-owned by BMJ to the co-owners of the Journal, to publish the Work in this journal and any other BMJ products and to exploit all rights, as set out in our licence.

The Submitting Author accepts and understands that any supply made under these terms is made by BMJ to the Submitting Author unless you are acting as an employee on behalf of your employer or a postgraduate student of an affiliated institution which is paying any applicable article publishing charge ("APC") for Open Access articles. Where the Submitting Author wishes to make the Work available on an Open Access basis (and intends to pay the relevant APC), the terms of reuse of such Open Access shall be governed by a Creative Commons licence – details of these licences and which Creative Commons licence will apply to this Work are set out in our licence referred to above.

Other than as permitted in any relevant BMJ Author's Self Archiving Policies, I confirm this Work has not been accepted for publication elsewhere, is not being considered for publication elsewhere and does not duplicate material already published. I confirm all authors consent to publication of this Work and authorise the granting of this licence.

For peer review only

Public opinion on sharing data from health services for clinical and research purposes without explicit consent: an anonymous online survey in the UK

- Linda A. Jones [1], *patient and public involvement lead*. ORCID 0000-0001-9347-5715.
- Jenny R. Nelder [1], *project manager*.
- Joseph M. Fryer [1], *data architect*.
- Philip H. Alsop [2], *research advisory group member*.
- Michael R. Geary [2], *research advisory group member*.
- Mark Prince [2], *research advisory group member*.
- Rudolf N. Cardinal [1,3,*] (rnc1001@cam.ac.uk), *associate professor of clinical informatics and honorary consultant liaison psychiatrist*. ORCID 0000-0002-8751-5167.

[1] Department of Psychiatry, University of Cambridge, Clifford Albutt Building, Bay 13, Cambridge Biomedical Campus, Cambridge CB2 0AH, UK.

[2] Unaffiliated.

[3] Cambridgeshire and Peterborough NHS Foundation Trust, Liaison Psychiatry Service, Box 190, Addenbrooke's Hospital, Cambridge CB2 0QQ, UK.

[*] Corresponding author.

Version of: **13 Feb 2022**.

Abstract word count: **300 (max 300 words)**.

Text word count: **5,565**.

For peer review only

For peer review only - http://bmjopen.bmj.com/site/about/guidelines.xhtml

1. ABSTRACT

OBJECTIVES. UK National Health Service (NHS/HSC) data is variably shared between healthcare organizations for direct care, and increasingly de-identified for research. Few large-scale studies have examined public opinion on sharing, including of mental health (MH) versus physical health (PH) data. We measured data sharing preferences.

DESIGN/SETTING/INTERVENTIONS/OUTCOMES. Pre-registered anonymous online survey, measuring expressed preferences, recruiting Feb–Sep 2020. Participants were randomized to one of three framing statements regarding MH versus PH data.

PARTICIPANTS. Open to all UK residents. Participants numbered 29275; 40% had experienced a MH condition.

RESULTS. Most (76%) supported identifiable data sharing for direct clinical care without explicit consent, but 20% opposed this. Preference for clinical/identifiable sharing decreased with geographical distance and was slightly less for MH than PH data, with small framing effects. Preference for research/de-identified data sharing without explicit consent showed the same small PH/MH and framing effects, plus greater preference for sharing structured data than de-identified free text. There was net support for research sharing to the NHS, academic institutions, and national research charities, net ambivalence about sharing to profit-making companies researching treatments, and net opposition to sharing to other companies (similar to sharing publicly). De-identified linkage to non-health data was generally supported, except to data held by private companies. We report demographic influences on preference. A majority (89%) supported a single NHS mechanism to choose uses of their data. Support for data sharing increased during COVID-19.

CONCLUSIONS. Support for healthcare data sharing for direct care without explicit consent is broad but not universal. There is net support for the sharing of de-identified data for research to the NHS, academia, and the charitable sector, but not the commercial sector. A single national NHS-hosted system for patients to control the use of their NHS data for clinical purposes and for research would have broad support.

REGISTRATION. ISRCTN37444142.

2. STRENGTHS AND LIMITATIONS OF THIS STUDY

- Patient and public involvement in study design.
- Detailed questions measuring public opinion on health data sharing for clinical and research purposes.
- Large national sample giving high power, with quantitative analysis, sensitivity analyses to approximate known population demographics, and serendipitious examination of pandemic effects.
- Embedded randomized experiment to control and measure variation due to framing.
- The sample remained under-representative of some demographic groups despite weighting, with potential for unmeasured selection (including self-selection) bias reducing generalisability.

For peer review only - http://bmjopen.bmj.com/site/about/guidelines.xhtml

3. INTRODUCTION

In the United Kingdom (UK), health-related information is recorded routinely by health care professionals and patients within the National Health Service (NHS; England, Scotland, Wales) or Health and Social Care (HSC; Northern Ireland), henceforth "NHS" for brevity. When combined with personal identifiers such as names and addresses, the data represent "confidential patient information" (CPI),[1] used to provide care and managed according to standard principles.[2–7] It is "owned" legally and managed by the NHS organisation recording it.[5,8] De-identified or anonymised forms of the data may be used for research (**Figure 1**) without explicit consent,[5,6] as pledged by the NHS.[9] Identifiable data may be used for research with consent, or—under restricted circumstances—without.[1,5,6] "Fully" anonymised data are not subject to UK data protection legislation.[5,6] However, even supposedly anonymised data relating to individual people carries some risk of re-identification via "jigsaw" attacks.[10]

Our understanding of public wishes about data sharing is incomplete.[11] Information is sometimes not shared clinically when it should be,[3,12] and patients may be surprised and frustrated by failures to share in a "national" health service.[13,14] Previous work, whilst establishing themes in public views on data linkage and sharing for research,[11,15–21] has highlighted the very small scale of many studies, and the paucity of research about the views of minority groups and the acceptability of sharing some types of data, such as mental health (MH) data. Mental illnesses can carry significant stigma[22] but are associated with substantial loss of life expectancy,[23] necessitating improvements in research and care. Some research requires multi-source data, but linkage is complex and may involve transient use of identifiable information.[24] It is unclear to what extent the public supports such work, and whether support varies with the type of data to which health data is linked (e.g. education versus criminal justice); there is little prior research in this area.[18] Proposed national systems for NHS data research such as "care.data" have previously aroused public opposition and ire,[25] as have information governance (IG) breaches,[26] and there is current debate about the newest NHS data sharing proposal, General Practice Data for Planning and Research (GPDPR).[8,27]

What do patients and the public want now? We studied views on the sharing of identifiable health data (for clinical purposes) and de-identified health data (for research) within the UK. We examined data destinations ranging from local NHS services to public distribution. We distinguished MH and physical health (PH) data, and for research also structured data (coded information) versus free-text data (e.g. narrative information typed by clinicians).[28] We asked about data linkage for research. We examined the effects of decision "framing", a term describing a decision-maker's conception of the possibilities and contingencies involved in a choice: since choices can be affected materially by the way in which they are formulated and presented,[29] we superimposed a randomized experiment to quantify how opinions on sharing were affected by the framing of risk versus benefit. We examined the effect of the COVID-19 pandemic upon preferences. We sought views on potential systems to govern NHS data sharing for clinical and research purposes and to offer direct participation in research.

4. METHODS

4.1. Patient and public involvement

The research team advertised and formed a research advisory group (RAG) comprising patients and carers, who designed and co-produced the study and questionnaire with the research team (see **Supplementary Methods,** S1.1). Patients, carers, and other members of the public participated in the study. Some members of the RAG co-wrote this paper.

For peer review only - http://bmjopen.bmj.com/site/about/guidelines.xhtml

4.2. Approvals

We obtained NHS ethics approvals (East of Scotland Research Ethics Service, reference 19/ES/0144) and pre-registered the study (https://doi.org/10.1186/ISRCTN37444142).

4.3. Inclusion and exclusion criteria; sample size

The inclusion criteria were current residence in the UK and informed consent. The ability to take an online survey (alone or supported) was implicit. Participants under 16 required the permission of their parent or guardian to participate and were asked to report whether they had assistance. We sought a power of 0·9 to detect a "small" effect (Cohen's d=0·1) for the framing intervention (described below), with an estimated minimum n=433/group, but beyond that sought a large sample of the UK population.

4.4. Recruitment

Approvals covered public announcements and recruitment via health service sites, in person or through a variety of media. The study was adopted onto the National Institute for Health Research (NIHR) Clinical Research Network (CRN) portfolio; 216 general practice (GP) surgeries and 154 large healthcare organizations (e.g. acute care Trusts, MH Trusts, community hospitals, ambulance Trusts) supported recruitment. The study ran from 2020-02-07 to 2020-09-30.

4.5. Survey

Data were collected using REDCap.[30] The survey is reproduced in the **Supplementary Methods** (S1.2). It asked for the respondent's views on current and desirable practice for sharing identifiable data for clinical care purposes; personal experience of MH/PH conditions and care; preference for sharing identifiable PH/MH data (for clinical care purposes) to a range of NHS "destinations"; preference for sharing de-identified structured PH/MH data (for research) to a range of potential research "destinations"; similarly for data including de-identified free-text notes; views about potential systems for managing data consent in the NHS; views about linkage for research to non-NHS data sources; and demographics.

4.6. Randomized framing intervention

We hypothesized that the context of questioning would affect willingness to share MH versus PH data, and sought to control and measure this effect. Before we asked about willingness to share different kinds of health data, we presented one of three framing statements: neutral, "concern" (about MH data being more sensitive), or "holistic" (about the importance of joined-up PH/MH care) (**Supplementary Methods**, S1.2). Participants were randomized to one of the three statements.

4.7. Data processing

Where participants agreed to leave a postcode, this was converted to a larger Office for National Statistics (ONS) geographical area, to prevent inadvertent identification. The geographical area was linked to its known population and Index of Multiple Deprivation (IMD). If the participant provided sufficient information, the ONS National Statistics Socio-Economic Classification (NS-SEC) was also calculated. (See **Supplementary Methods,** S1.3.)

4.8. Pandemic

By chance, our study spanned the UK onset of the COVID-19 pandemic. This had many consequences, including "lockdowns". Major changes were made to NHS data handling, including instructions to share CPI

For peer review only - http://bmjopen.bmj.com/site/about/guidelines.xhtml

for public health purposes relating to the pandemic,[31] media reports of sharing of patient-level de-identified data with industry,[32] and guidance for GPs to include additional information in patients' Summary Care Record (SCR, England) unless they had previously opted out.[33] We examined whether the pandemic was associated with changes in preference relating to data sharing, using 2020-03-23 (first UK "lockdown") as the split point (factor "pandemic": levels "before lockdown", "at/after lockdown").

4.9. Analysis

We analysed using R v3.6.3.[34] We analysed categorical associations via χ^2 tests, and effects upon ordinal Likert-type scales (phrased linguistically to approximate interval scales) via analysis of variance (ANOVA). With a large sample size, the central limit theorem means that the distribution of means and mean differences tends to normal even though the parent population is non-normal, and ANOVA is robust to non-normality,[35–37] permitting ANOVA of discrete dependent variables. Scales measuring likelihood were quantified as −2 very unlikely, −1 unlikely, 0 not sure, +1 likely, +2 very likely. Yes/no scales were quantified as −1 no, 0 not sure, +1 yes. Models involving within-subjects terms were analysed using the *lmer* and *lmerTest* packages, using type III sums of squares, and are expressed thus (~ "is predicted by"; A×B interaction; A*B denotes the inclusion of main effects A and B and their interactions). Statistics are shown to 3 significant figures (or as integers for percentages reported as annotations on figures or in the abstract/discussion) and degrees of freedom are rounded to integers. We set α=0·05, and report "NS" for "not significant" and "VLP" for a very low p value, $p<2{\cdot}2\times10^{-16}$.

Opinions on sharing clinical/identifiable data were analysed using a model termed C1: *sharing~destination*nature*framing*pandemic+(1|subject)*. "Destination" had four levels (local, regional, national, UK-wide), "nature" had two (PH, MH), and framing had three (neutral, MH concern, holistic). We followed up nature×framing interactions by analysing MH and PH data separately using the simplified model C1B: *sharing~destination*framing+(1|subject)*.

To examine the effects of demographic factors and experience, we used a larger model, C2: *sharing~destination*nature*framing*pandemic+age+gender+ethnicity+education+sexuality+religion+nation+imd_quartile+nssec+mh_experience*nature+(1|subject)*. This was only possible for people who provided all necessary demographic information. Levels for demographic factors were as per **Supplementary Table 1**, plus sexuality (two levels: heterosexual/straight, LGBT+ [including homosexual/gay/lesbian, bisexual, other/self-described]) and NS-SEC (five levels). We did not include all interaction terms for demographic factors (as formal tests of "intersectionality" effects) because of the combinatorial explosion this would entail; instead, this model tests main effects of demographic factors plus the specific hypothesis that MH experience affects sharing of MH/PH data differentially.

Opinions on sharing de-identified data for research were analysed using model R1: *sharing~destination*nature*detail*framing*pandemic+(1|subject)*. "Destination" had six levels (NHS, academia, charities, companies conducting treatment research, other companies, publicly); "detail" had two levels (structured only, free text); other factors were as before. To examine nature×framing interactions, we used the simplified model R1B: *sharing~destination*framing+(1|subject)*. For demographic analysis we used model R2: *sharing~destination*nature*detail*framing*pandemic+age+gender+ethnicity+education+sexuality+religion+nation+imd_quartile+nssec+mh_experience*nature+(1|subject)*.

Sensitivity analyses were conducted by weighting to UK population demographic proportions. Effect size plots were created for key models. (See **Supplementary Methods,** S1.4–S1.5.)

For peer review only - http://bmjopen.bmj.com/site/about/guidelines.xhtml

Willingness for linkage to non-NHS data for research (data source, eight levels) was analysed for all participants using model L1: *willingness~source*pandemic+(1|subject)*. For demographic analysis we used model L2: *willingness~source*pandemic+age+gender+ethnicity+education+sexuality+religion+nation+imd_quartile +nssec+mh_experience+(1|subject)*.

A thematic analysis was performed on free-text comments (see **Supplementary Methods,** S1.6).

5. RESULTS

5.1. Participants

Consenting participants numbered 29275. Recruitment is shown in **Supplementary Figure 1A–B;** 8019 participated before UK "lockdown" and 21256 on/after that date. Not everyone completed the survey: participation by stage is shown in **Supplementary Figure 1C,** with 73·6% completing all stages. Median completion time was 18·4 minutes. Participants were evenly distributed across framing conditions (neutral 9812, MH concern 9744, holistic 9719; χ^2_2=0.475, NS).

Demographics are shown in **Supplementary Figure 2A–J** (with free-text responses in **Supplementary Results,** S2.2). Relative to the UK population (**Supplementary Table 1**), our sample under-represented the youngest and oldest age ranges, males, those of non-white ethnicity, those with less formal education, those professing a religion, residents of UK nations other than England, and people living in more deprived areas. Weighting yielded substantial though incomplete improvement (**Supplementary Results,** S2.4). There was coverage of most UK local authority areas (**Supplementary Figure 2I**).

A MH condition had been experienced by 40·0% of participants (**Supplementary Figure 3**), primarily depression and anxiety disorders (of people who had experienced a MH condition, 93·8% reported having had depression or anxiety at some point). Of participants who had experienced a MH condition, 84·9% had used MH services, primarily their GP and NHS psychological therapy services (**Supplementary Figure 3**). PH services had been used by 88·2% of respondents, primarily GP and outpatient services (**Supplementary Figure 3**).

5.2. Sequence

We first report preferences for clinical data sharing based on multiple-choice questions (**Figure 2**), before examining in detail participants' likelihood of sharing data for clinical and research purposes (**Figure 3,** with weighted equivalent in **Supplementary Figure 4**), preferences for data linkage (**Figure 4, Supplementary Figure 5**), and changes associated with the pandemic (**Figure 5**). We then report effect sizes for the analytical models, including for demographic factors (**Figure 6A–C, Supplementary Figure 6A–C**), and finally report views on a possible national consent system (**Figure 7A–F, Supplementary Figure 7A–F**).

5.3. Sharing identifiable data for clinical purposes

Understanding of current NHS practice regarding identifiable data sharing between care providers, without asking the patient each time, are shown in **Figure 2A**. In practice, sharing varies by area, e.g. depending on whether a local/regional shared care record is operative in part of England,[14] or according to limited national systems such as the Intra-NHS Scotland Information Sharing Accord[38] and Scottish Emergency Care Summary,[39] the Northern Ireland Electronic Care Record,[40] the English SCR,[41] and a variety of systems in Wales.[42] To our knowledge, there is no UK-wide sharing, but 30·0% of respondents thought that there was free sharing of identifiable data across the UK.

For peer review only - http://bmjopen.bmj.com/site/about/guidelines.xhtml

When asked preferences via a single multiple-choice question (**Figure 2B**), there was majority (55·4%) support for sharing identifiable data for direct care across the UK, without being asked first, and 76·4% supported sharing at least locally, but a substantial minority (20·3%) said that sharing should not occur without the patient being asked first.

5.4. Sharing mental and physical health data for clinical purposes and for research

Willingness to share health data without being asked every time is shown in **Figure 3** by purpose, nature, and destination (with corresponding weighted data in **Supplementary Figure 4**).

For clinical purposes (with identifiable data), there was strong net willingness to share (**Figure 3A–C**), with 89·9% (PH data) or 83·1% (MH data) rating themselves "likely" or "very likely" to share to local NHS services. The most important determinant was destination, with stronger support the more local the sharing (that is, preference decreased with geographical distance). This monotonic effect, when participants were asked to rate each destination and nature separately, had not been evident in the one-from-many question about health data in general, asked previously (**Figure 2B**). People were slightly more willing to share PH than MH data. There were significant but very small effects of the framing statement, primarily that "MH concern" framing reduced willingness to share MH data. In the whole-sample analysis (model C1), there were highly significant effects of destination ($F_{3,169348}$=6490, VLP) and nature ($F_{1,169484}$=6080, VLP), as well as interactions including nature×framing ($F_{2,169484}$=78·4, VLP) (**Figure 3C**, **Supplementary Figure 6A**). This interaction was driven primarily by a simple effect of "MH concern" framing to reduce sharing for MH data [model C1B: PH data, no effect of framing ($F_{2,24461}$=1·18, NS); MH data, effect of framing ($F_{2,24157}$=8·36, p=0·000234); pairwise comparison within MH data, MH concern versus neutral, p=0·00443]. Framing effects were also lessened for geographically broader destinations. Effect sizes are reported further below.

For research purposes (with de-identified data), destination was an extremely strong driver of preference ($F_{5,535334}$=87800, VLP) (**Figure 3D–I**). On average, people expressed strong support for sharing to the NHS, academia, or national charities for research purposes. For NHS sharing, the most popular destination, 89·2% (PH data, structured only), 85·2% (MH data, structured), 81·6% (PH data, free text), and 78·7% (MH data, free text) of participants rated themselves "likely" or "very likely" to share. Support and opposition were approximately equally balanced for sharing to profit-making companies researching treatments. There was strong net opposition to sharing to other types of companies, approximately equal to that for sharing publicly. Only 20·6% (PH data, structured only), 17·6% (MH data, structured), 16·4% (PH data, free text), and 14·9% (MH data, free text) rated themselves "likely" or "very likely" to share to other types of companies. There was a small but significant preference for sharing PH (versus MH) data, and likewise higher preference for sharing structured-only versus free-text data. In the whole-sample analysis (model R1), there were highly significant effects of destination, nature, and detail, plus interactions including destination×nature×detail (**Supplementary Figure 6B**). Framing effects included nature×framing, though simple framing effects were not significant for PH or MH data separately (model R1B). Effect sizes are reported further below.

Sensitivity analyses weighted to UK population demographics (**Supplementary Results,** S2.5) were consistent with the primary analysis.

5.5. Linkage to non-health data for research

We asked about linking of NHS data to non-health data sources for research, ultimately with de-identified data. There was net support for all "state" sources and university-held data (**Figure 4**), ranging from 72·8% support for education data to 56·6% support for social security/Work and Pensions data, but net opposition

For peer review only - http://bmjopen.bmj.com/site/about/guidelines.xhtml

regarding private company data (**Figure 4**), for which only 17·3% were supportive. Weighted responses were very similar (**Supplementary Figure 5**).

5.6. Changes related to the COVID-19 pandemic

Following "lockdown", willingness to share identifiable data for clinical purposes increased, with no significant change in the already high preference for local sharing, but progressive increases for sharing to more remote parts of the NHS (model C1, destination×pandemic, $F_{3,169348}$=26·6, VLP; **Figure 5A**).

Willingness to share de-identified data for research purposes generally increased for more-preferred destinations (NHS, academia, charities), except in the "MH concern" framing condition (model R1, destination×pandemic, $F_{5,535334}$=78·2, VLP; **Figure 5B**), but did not change for less-preferred destinations (commercial and public sharing).

Preference for linkage to university data increased (source×pandemic; **Figure 5C; Supplementary Figure 6C**). There was a less consistent decrease in preference for linkage to private data (**Figure 6C, Supplementary Figure 6C**) and police data (model L2; **Figure 6C**).

5.7. Effect sizes and influence of demographic factors

Preference varied according to demographic factors and experience of MH illness. For clinical purposes, there were several demographic effects (model C2; **Figure 6A** shows significant terms with effect sizes). Age was a significant factor, with the age bands most willing to share being 25–44 and 75+, and the 18–24 band being least willing. Males were more willing to share data than females. Those of minority ethnicity were less willing to share than those of white ethnicity. There was a main effect of education, and across educational levels, those of Level 3 were most willing and those of Level 4+ least willing. Those of minority religions were less willing to share. Those from the most-deprived IMD quartile were also less willing. There were no significant effects of sexuality ($F_{1,12334}$=2·21, NS), NS-SEC ($F_{4,12335}$=1·32, NS), or nation ($F_{3,12335}$=2·13, NS). Personal experience of MH illness specifically reduced willingness to share MH data for clinical purposes (nature×MH experience).

For research purposes, significant effects were similarly observed for age, gender, ethnicity, religion, and IMD quartile (model R2, **Figure 6B**). The age distribution was clearly U-shaped, with greater willingness to share among the youngest and oldest groups. As before, there was no effect of sexuality ($F_{1,12336}$=1·58, NS). There was no effect of education ($F_{4,12348}$=2·15, p=0·072), but there was an effect of nation (with people living in Wales more willing to share and those in Scotland less so, relative to England), and of socioeconomic status (NS-SEC; **Figure 6B**). People with MH experience were significantly *more* likely to share MH data for research purposes (nature×MH experience, $F_{1,296111}$=6·15, p=0·0132).

For linkage, the patterns were broadly as before (**Figure 6C**, **Supplementary Figure 6C**). Data source strongly influenced preference (education>universities≈police≈housing≈transport>social security>immigration≫private companies). There were also effects as before of age, ethnicity, education, and IMD quartile. There was no effect of gender ($F_{1,12338}$=2·32, NS), religion ($F_{2,12338}$=1·86, NS), nation (F<1), or MH experience (F<1), but there was now an effect of sexuality, with LGBT+ people being less willing for linkage.

5.8. A possible national consent system

We proposed varieties of a national system for patients to decide how their NHS data is used. Participants were most willing to sign up via a web site or in person (**Figure 7A,C**). Willingness was similar regardless of whether consent information was managed by the NHS centrally, a local NHS Trust, or the patient's GP

For peer review only - http://bmjopen.bmj.com/site/about/guidelines.xhtml

(**Figure 7B**). Overall, 88·8% of people said they were "likely" or "very likely" to sign up to such a system (**Figure 7D**). Weighted responses were very similar (**Supplementary Figure 7**).

Most people (63·6%) wanted a single NHS system to sign up for participatory research (**Figure 7E; Supplementary Figure 7E**).

There was broad support for the draft consent form and for adding information about contact methods, data security, and management of the consent information, with 66·1%–89·8% answering "agree" or "strongly agree" to all questions except about awareness of the NHS National Data Opt-Out (NDO) (**Figure 7F**; **Supplementary Figure 7F**); 46·7% (unweighted) agreed that they had been aware of the NDO.

Common subthemes from the thematic analysis (*n*>100, **Supplementary Results,** S2.9–S2.10) included: the need for clarity around de-identification (*n*=164); the critical importance of healthcare data security (*n*=594); the desirability of data sharing (*n*=290); that opt-outs should be more prominent (or default) or linked to the NDO (*n*=134); that profit-making use should not happen or that the NHS/patients should benefit from such profits (*n*=198); that clinical users should be specified in more detail (*n*=147); research users likewise (*n*=268); and that healthcare data should not be available to private or third-party companies without specific permission (*n*=417). For full theme/subtheme descriptions and tallies, see **Supplementary Results** (S2.9).

6. DISCUSSION

6.1. Summary

Many respondents believed that health data is shared UK-wide for clinical purposes without explicit consent, when sharing is usually more limited. A majority (76%) supported such sharing, though a significant minority (20%) opposed it. Geographically broad sharing was endorsed, though with stronger support for more local destinations. People preferred to share PH (versus MH) data but this was less important than the destination.

For research, with de-identified data, there was strong net support for sharing without explicit consent to the NHS, academic research institutions, and research charities. There was net ambivalence regarding private companies researching treatments, and strong net opposition for sharing to other companies or publicly. There was a small preference for sharing PH over MH data (a smaller difference than for clinical purposes), and greater support for structured-only data over de-identified free text. There was net support for research linkage to state and university data sources, but opposition regarding data held by private companies.

Framing statements influenced MH/PH preferences, but only to a small degree. Age, gender, ethnicity, education, religion, and IMD were associated with willingness to have health data shared or linked, though not nearly as strongly as destination/source. Personal experience of MH conditions was associated with reduced willingness to share MH data for clinical purposes, but greater willingness to share it for research. After COVID-19 lockdown there was greater willingness regarding already-preferred destinations.

Respondents endorsed a suggested UK-wide system allowing patients to control the clinical/research uses of their data and to sign up for participatory research, with 89% saying they would be likely or very likely to use such a system. In commenting, respondents frequently emphasized the importance of data security and that NHS data should not be made available to private or third-party companies without specific permission. Comment themes reflected the tensions previously noted in this area, including about healthcare and research benefits, security, governance, trust, and vulnerable groups.[43–45]

For peer review only - http://bmjopen.bmj.com/site/about/guidelines.xhtml

6.2. Strengths and weaknesses

Strengths include patient/public involvement in the study design; the largest such study to date by 1–3 orders of magnitude,[15,17,19–21,46] giving high power; sensitivity analyses weighted to population demographics; detailed questions about data sharing for clinical/research purposes, including about the type of data and the destination, plus linkage to non-health data; a randomized framing experiment to control and measure this source of variation; quantitative analysis including of relative effect sizes; consultation on ways to improve the current situation; and serendipitous examination of the effects of COVID-19 on data sharing views.

The major weakness is that the sample remained under-representative of some groups despite weighting, with potential for unmeasured selection (including self-selection) bias, reducing generalisability. We consider the reasons for the bias, and potential routes to improving representativeness in future work, in the **Supplementary Discussion** (S3.1).

6.3. Destination and purpose

The Caldicott framework (1997)[47] and reviews (2013, 2016),[3,12] regarding safe information sharing for direct clinical care, included the principle that the "duty to share information can be as important as the duty to protect patient confidentiality"[3] and noted that information was often not shared when it should be, for fear of inappropriate disclosure.[12] That was despite legislation creating a duty upon providers to share information with professionals when that is likely to facilitate the individual's health or social care, disclosure is in their best interests, and they do not or are not likely to object.[48] That is in essence an opt-out system. This legislation conflicted with some prior studies of public opinion.[49] We provide more background on relevant legislation in the **Supplementary Discussion** (S3.2). The 2016 Caldicott review noted low public understanding around how health information is used, but "an expectation that information is shared for direct care".[12] We observed net support for such sharing that varied with geographical destination and was by no means universal, but was nevertheless strong.

In relation to research and other non-clinical activities, the recommendation that people be able to opt out from personal confidential data being used beyond their own direct care[12] led to the NHS National Data Opt-Out.[50,51] This relates to the use of CPI (identifiable information) for purposes such as research, conducted under NHS Act Section 251 (§251) approvals.[1,52] It does not apply to direct clinical care, local audit or service evaluation, or de-identified information.[50,51] Our study and others show it remains unknown to many.[46] Furthermore, it is not simple (see **Supplementary Discussion,** S3.2) and we suspect many do not fully understand its scope. Conversely, from the researcher's perspective, §251 approval is often still required for linkage studies in which researchers never see identifiable information: there is no standardized "trusted third party" system for centralized linkage of identifiable information, and inconsistent adoption of de-identified linkage methods.

"Destination" was by far the strongest driver of preference for sharing and linkage. This pattern is established: willingness to allow researchers/clinicians access to health data, but far greater reservations about industry.[53,54] An important basis for this is mistrust of the security and/or motives of commercial organizations,[25,26,55] as our participants noted.

6.4. Demographic effects

A common demographic theme was that minority groups (of ethnicity, religion, and sexuality) and deprived groups were less willing to share. This might reflect experience of disadvantage to, or discrimination against, these groups.[56,57] Ethnicity has had mixed effects on preference for national electronic health record

For peer review only - http://bmjopen.bmj.com/site/about/guidelines.xhtml

(EHR) systems.[19] In our study, age effects were generally biphasic, with higher willingness amount the youngest and eldest. Youth may be associated with familiarity with data and/or greater support for EHRs,[58] and older age with an increasing burden of illness, itself associated with support for national EHR systems.[58] Educational effects were relatively inconsistent, being small and present for clinical and linkage preferences though in quantitatively different ways, and not being significant for research preferences. Males were slightly more willing to share than females. Similar results have been observed before,[46] but not always;[58,59] one reason might be gender-based healthcare discrimination.[60] Higher support for research sharing in Wales may relate to established national research systems there;[61,62] the reasons for reduced willingness in Scotland are unclear, but similar systems there are younger.[63] Those with personal experience of MH illness were less willing to share identifiable MH data for direct care. This may reflect experience of discrimination or stigma[64,65]—which can have disproportionate effects in subgroups.[65,66] However, the same people were more willing to share de-identified MH data for research, potentially reflecting increased prioritization of MH research.[67]

Demographic variations in preference may reflect differences in perception of current data rules or security practices, reasons for concern about uses of health data, or degree of concern. UK law prohibits variation of policy according to these factors.[68] Better understanding and public information may be required to address these groups' concerns,[11,69] but improvements in health equity are also required.[17,56] However, the effect sizes of these demographic predictors were not large enough to override the net support for data sharing, given the right destinations.

6.5. Framing and pandemic effects

We observed small but significant framing effects.[29] Our framing statements were true and non-alarmist, so real-world framing effects might sometimes be larger. Others have observed larger effects via "loss framing" (emphasizing the potential adverse consequences of not consenting over the potential benefits of consenting), and through other manipulations like the placement of framing statements.[70] Media coverage of health data sharing is influential.[71,72] Despite best intentions it is impossible to avoid framing effects entirely,[29] so those presenting information should be aware of these whilst presenting accurately the risks and benefits of data sharing/linkage.

During COVID-19, despite press coverage[32] of an enforced increase in NHS data sharing for public health purposes,[31,33] support for sharing/linkage increased—but only for some already-favoured destinations. Publicity regarding NHS care[73] and research regarding COVID-19[74,75] may have driven the increase in support for sharing with the NHS, universities, and research charities. We did not analyse the pandemic-related trajectory of responses beyond examining the change at/after the first UK "lockdown", and public views may have changed further after the conclusion of our study.

6.6. Conclusions

Participants supported a central system for patients to control the uses of their data, and likewise a single NHS mechanism to sign up for active research participation. There is a trade-off between the scientific desirability of everyone contributing de-identified data, including to avoid bias,[76,77] and the desirability of individual control over data use.[78] As we suggest below, a reasonable balance might be a central system to opt out from identifiable clinical use, identifiable (§251) research use, or de-identified research use of one's data, and to opt in for participatory research. This would complement efforts to improve people's access to their own data.[79]

The majority support that we observed for clinical sharing without explicit consent perhaps makes such sharing reasonable as a default (opt-out) position, given the potential advantages for many people's own

For peer review only - http://bmjopen.bmj.com/site/about/guidelines.xhtml

care, subject to strict IG rules (who has access, and when). Under UK data protection laws, the legal basis for NHS organizations to hold patient data is not consent (see **Supplementary Discussion,** S3.2). The Caldicott principles require relevant information sharing for direct care under many circumstances,[3,12] and NHSX have set out the information governance requirements for regional shared care records (ShCRs), several of which are already in operation (and may use opt-outs), and for cross-ShCR sharing.[80] However, a significant minority of participants in our study opposed clinical sharing without explicit consent, mandating (we suggest) at least a public information campaign about opt-outs, sufficiently targeted to reach groups most concerned about data sharing, if broader sharing were to occur, to conform to the Caldicott principle of "no surprises".[3]

There was strong net support for NHS, academic, and charity researchers accessing de-identified health data. Opt-outs are often offered, even if they are not legally required, for research using de-identified data (see **Supplementary Discussion,** S3.2). A standard method for conducting such research is via a trusted research environment (TRE).[24,61,81–83][24,61,81–83] Approved researchers come "into" the secure environment, which can be highly controlled, to interact with relevant data (e.g. pseudonymised; **Figure 1**). After analysis, aggregation, and other statistical disclosure control (SDC),[84] results go "out" for publication (**Figure 1**). The principle is of the "five safes": safe people, safe projects, a safe setting, safe data, and safe outputs.[83] The lower preference for sharing de-identified free-text data versus de-identified structured data is congruent with the increased sensitivity of free-text data, and the fact that technical methods for de-identifying free text remain imperfect;[85,86] additional safeguards surrounding that kind of data are justified.

We did not examine preferences regarding research uses of identifiable data, except about being contacted for direct research participation and about awareness of the NDO. The NDO only applies to work using identifiable data without consent (**Supplementary Discussion,** S3.2). Separately, some argue that it should be easier to gain permissions to conduct research using identifiable or potentially identifiable data, as part of an ethical duty to participate in research.[87] We provide no evidence to suggest changes to the operation of this process, but we found strong support for a central mechanism to control the uses of one's data and to sign up for direct research participation.

In contrast to the support for NHS/academic/charity research, respondents did not support research sharing to private companies. Some have suggested this is addressable in part by public education.[11] We suggest respecting public preference, and not giving commercial organisations direct access to patient-level NHS data for research, even de-identified, without consent. (This is distinct from the common NHS practice of employing companies, such as EHR software providers, to manage NHS data securely for clinical purposes.) We think that this does not rule out all industrial research uses of data, which could happen according to at least three methods. The first is via consent, as for commercial treatment trials. Secondly, companies could collaborate with NHS/academic researchers. For example, an artificial intelligence company could provide an untrained algorithm; NHS staff could train it on patient-level data; the company could receive a trained algorithm back whilst never having access to the data (assuming verification that the algorithm cannot "embed" detailed data features during training). Thirdly, methods exist whereby software queries come "in" to the TRE, and semi-automatic or automatic SDC occurs before results go "out".[88–90][88–90] This allows research to take place without researchers having access to patient-level data, and can also support "federated" queries across sites. Data that have undergone suitable SDC (e.g. aggregation) can be published, and are therefore suitable for industrial access if desired. Regardless, as our participants commented, the NHS might charge for such access,[11,91] and full transparency is essential. Formal, consultation-based standards governing this NHS–commercial interface would be desirable.

Some noted the need to control the nature of information sharing in greater detail, and this would need further detailed consultation. Our data may help to frame this. There is likely to be a trade-off between the

For peer review only - http://bmjopen.bmj.com/site/about/guidelines.xhtml

level of fine-grained control offered and a need for simplicity in a nationwide system; our effect sizes (**Figure 6B**) suggest, for example, that "destination", and whether free text or only structured data is involved, should be prioritized over other factors such as mental versus physical health data.

Governance of UK health data must be transparent and reflect the views of patients.[11,16] Regardless of legal authority, it is important that health data is processed in ways that have a "social licence".[92] As the UK Government seeks to change data legislation[93] and emphasize health data in its science strategy,[94] we hope this study contributes to the conversation.

7. ACKNOWLEDGEMENTS

We thank all those who took part in the study and gave their views. We thank all the investigators, local collaborators, local NIHR CRN staff, and health care staff across the UK who supported the study. We thank also Elizabeth Rotherham (research advisory group member) for help with design; Nicola Gleadle for practical support; Jane Gaffa, Rachel Kyd, and Mary-Beth Sherwood for Research and Development support; Sally-Anne Hurford and Ruth Hudson for NIHR CRN advice; Chess Denman for investigator approval; Laura Marshall for CPFT publicity; Hannah Clarke for helpful comments; REC and HRA staff for ethical and regulatory approvals; the Cambridge Autism Research Database for additional publicity; and Artur Arikainen, Andres Colubri, Lisa Eckstein, Julia Ivanova, and David Wyatt for helpful comments during open peer review.

8. DATA AVAILABILITY

After removal of all free text, anonymised data are available[95] from the University of Cambridge Data Repository at https://doi.org/10.17863/CAM.75784, with participants' consent.[96]

9. AUTHOR CONTRIBUTIONS

RNC conceived the study. LAJ, JRN, PHA, MRG, MP and RNC designed it (with ER). LAJ coordinated its execution. JMF advised on REDCap implementation and execution. RNC, LAJ, and JMF had access to the original data; the final data set is public. RNC analysed the data. RNC and LAJ drafted the manuscript. All authors edited the manuscript and approved submission.

10. FUNDING

Supported by a UK Medical Research Council (MRC) Mental Health Data Pathfinder award (MC_PC_17213 to RNC), and MRC grant MR/W014386/1 (Health Data Research UK Mental Health Data Hub, DATAMIND). Recruitment was supported by the NIHR CRN and the REDCap installation was supported in part by the NIHR Cambridge Biomedical Research Centre (BRC-1215-20014); the views expressed are those of the authors and not necessarily those of the NHS, the NIHR, or the Department of Health and Social Care.

11. COMPETING INTERESTS

The authors report no competing interests.

For peer review only - http://bmjopen.bmj.com/site/about/guidelines.xhtml

12. REFERENCES

1 UK. National Health Service Act 2006. 2006. https://www.legislation.gov.uk/ukpga/2006/41

2 UK General Medical Council. Confidentiality: good practice in handling patient information. 2017. http://www.gmc-uk.org/guidance/ethical_guidance/confidentiality.asp

3 Caldicott F. Information: To share or not to share? The Information Governance Review. 2013.https://www.gov.uk/government/publications/the-information-governance-review

4 UK Department of Health. Confidentiality: NHS Code of Practice. 2003.https://www.gov.uk/government/publications/confidentiality-nhs-code-of-practice

5 UK. Data Protection Act 2018. 2018. http://www.legislation.gov.uk/ukpga/2018/12/contents/enacted

6 European Parliament and Council. Regulation (EU) 2016/679 (General Data Protection Regulation). *Off J Eur Union* 2016;**L119**:1–88.

7 UK. Mental Capacity Act 2005. 2005. https://www.legislation.gov.uk/ukpga/2005/9/contents

8 Coulter A. Patient trust in plans to share primary care data. *BMJ* 2021;**373**:n1413. doi:10.1136/bmj.n1413

9 NHS England. The NHS Constitution for England. 2015.https://www.gov.uk/government/publications/the-nhs-constitution-for-england

10 Culnane C, Rubinstein BIP, Teague V. Health Data in an Open World. *ArXiv171205627 Cs* Published Online First: 15 December 2017.http://arxiv.org/abs/1712.05627

11 Ghafur S, Fontana G, Halligan J, *et al.* NHS Data: Maximising its impact on the health and wealth of the United Kingdom. Imperial College London 2020. https://doi.org/10.25561/76409

12 National Data Guardian for Health and Care. Review of Data Security, Consent and Opt-Outs. 2016.https://www.gov.uk/government/uploads/system/uploads/attachment_data/file/535024/data-security-review.PDF

13 NHS England. New local health and care partnerships could save lives. 2018.https://www.england.nhs.uk/2018/05/new-local-health-and-care-partnerships-could-save-lives/ (accessed 23 Aug 2019).

14 NHS England, Local Government Association. Local Health and Care Record Exemplars. 2018.https://www.england.nhs.uk/publication/local-health-and-care-record-exemplars/ (accessed 23 Aug 2019).

15 Aitken M, de St Jorre J, Pagliari C, *et al.* Public responses to the sharing and linkage of health data for research purposes: a systematic review and thematic synthesis of qualitative studies. *BMC Med Ethics* 2016;**17**:73. doi:10.1186/s12910-016-0153-x

16 Understanding Patient Data, Ada Lovelace Institute. Foundations of fairness: where next for NHS health data partnerships? 2020. https://understandingpatientdata.org.uk/sites/default/files/2020-03/Foundations%20of%20Fairness%20-%20Summary%20and%20Analysis.pdf (accessed 20 Apr 2020).

17 Hopkins H, Kinsella S, van Mil A. Foundations of fairness: views on uses of NHS patients' data and NHS operational data. Hopkins Van Mil: Creating Conditions Ltd 2020.

For peer review only - http://bmjopen.bmj.com/site/about/guidelines.xhtml

https://understandingpatientdata.org.uk/sites/default/files/2020-03/Foundations%20of%20Fairness%20-%20Full%20Research%20Report.pdf (accessed 20 Apr 2020).

18 Understanding Patient Data. Public attitudes to patient data use: a summary of existing research. 2018.https://understandingpatientdata.org.uk/sites/default/files/2019-05/Public%20attitudes%20key%20themes%200.pdf (accessed 23 Aug 2019).

19 Stockdale J, Cassell J, Ford E. 'Giving something back': A systematic review and ethical enquiry into public views on the use of patient data for research in the United Kingdom and the Republic of Ireland. *Wellcome Open Res* 2018;**3**:6. doi:10.12688/wellcomeopenres.13531.2

20 Aitken M, McAteer G, Davidson S, *et al.* Public Preferences regarding Data Linkage for Health Research: A Discrete Choice Experiment. *Int J Popul Data Sci* 2018;**3**:429. doi:10.23889/ijpds.v3i1.429

21 Ghafur S, Van Dael J, Leis M, *et al.* Public perceptions on data sharing: key insights from the UK and the USA. *Lancet Digit Health* 2020;**2**:e444–6. doi:10.1016/S2589-7500(20)30161-8

22 Henderson C, Evans-Lacko S, Thornicroft G. Mental Illness Stigma, Help Seeking, and Public Health Programs. *Am J Public Health* 2013;**103**:777–80. doi:10.2105/AJPH.2012.301056

23 Piotrowski P, Gondek TM, Królicka-Deręgowska A, *et al.* Causes of mortality in schizophrenia: An updated review of European studies. *Psychiatr Danub* 2017;**29**:108–20.

24 Downs JM, Ford T, Stewart R, *et al.* An approach to linking education, social care and electronic health records for children and young people in South London: a linkage study of child and adolescent mental health service data. *BMJ Open* 2019;**9**:e024355. doi:10.1136/bmjopen-2018-024355

25 Sterckx S, Rakic V, Cockbain J, *et al.* 'You hoped we would sleep walk into accepting the collection of our data': controversies surrounding the UK care.data scheme and their wider relevance for biomedical research. *Med Health Care Philos* Published Online First: 18 August 2015. doi:10.1007/s11019-015-9661-6

26 Powles J, Hodson H. Google DeepMind and healthcare in an age of algorithms. *Health Technol* 2017;:1–17. doi:10.1007/s12553-017-0179-1

27 Gayle D. GPs urged to refuse to hand over patient details. The Guardian. 2021.https://www.theguardian.com/society/2021/jun/01/gps-urged-to-refuse-to-hand-over-patient-details-to-nhs-digital (accessed 29 Jun 2021).

28 Ford E, Oswald M, Hassan L, *et al.* Should free-text data in electronic medical records be shared for research? A citizens' jury study in the UK. *J Med Ethics* 2020;**46**:367–77. doi:10.1136/medethics-2019-105472

29 Tversky A, Kahneman D. The framing of decisions and the psychology of choice. *Science* 1981;**211**:453–8. doi:10.1126/science.7455683

30 Harris PA, Taylor R, Thielke R, *et al.* Research electronic data capture (REDCap)--a metadata-driven methodology and workflow process for providing translational research informatics support. *J Biomed Inform* 2009;**42**:377–81. doi:10.1016/j.jbi.2008.08.010

31 Department for Health and Social Care, Hancock M. Coronavirus (COVID-19): notification to organisations to share information. 2020.https://www.gov.uk/government/publications/coronavirus-covid-19-notification-of-data-controllers-to-share-information

For peer review only - http://bmjopen.bmj.com/site/about/guidelines.xhtml

32 Lewis P, Conn D, Pegg D. UK government using confidential patient data in coronavirus response. The Guardian. 2020.https://www.theguardian.com/world/2020/apr/12/uk-government-using-confidential-patient-data-in-coronavirus-response (accessed 12 Apr 2020).

33 NHS Digital. SCR coronavirus (COVID-19) supplementary privacy notice. NHS Digit. 2020.https://digital.nhs.uk/services/summary-care-records-scr/scr-coronavirus-covid-19-supplementary-privacy-notice (accessed 27 Jun 2021).

34 R Core Team. *R: A language and environment for statistical computing*. Vienna, Austria: : R Foundation for Statistical Computing 2019. http://www.R-project.org/

35 Cardinal RN, Aitken MRF. *ANOVA for the Behavioural Sciences Researcher*. Mahwah, N.J: : L. Erlbaum 2006.

36 Myers JL, Well AD. *Research design and statistical analysis*. Second. Mahwah, New Jersey: : Lawrence Erlbaum Associates 2003.

37 Maxwell SE, Delaney HD. *Designing experiments and analyzing data: a model comparison perspective*. Second. Mahwah, New Jersey: : Lawrence Erlbaum Associates 2004.

38 NHS Scotland. Intra NHS Scotland Information Sharing Accord. 2010.https://www.informationgovernance.scot.nhs.uk/wp-content/uploads/2020/06/2020-06-17-Intra-NHS-Scotland-Sharing-Accord-v2.0.pdf

39 Morris LMM, Brown C, Williamson M, *et al.* The Scottish Emergency Care Summary--an evaluation of a national shared record system aiming to improve patient care: technology report. *Inform Prim Care* 2012;**20**:41–9. doi:10.14236/jhi.v20i1.46

40 NIDirect. Northern Ireland Electronic Care Record (NIECR) | nidirect. 2015.https://www.nidirect.gov.uk/articles/northern-ireland-electronic-care-record-niecr (accessed 6 Jul 2021).

41 Greenhalgh T, Stramer K, Bratan T, *et al.* Adoption and non-adoption of a shared electronic summary record in England: a mixed-method case study. *BMJ* 2010;**340**:c3111. doi:10.1136/bmj.c3111

42 Auditor General for Wales. Informatics systems in NHS Wales. 2018.https://senedd.wales/laid%20documents/agr-ld11353/agr-ld11353-e.pdf

43 Greenhalgh T, Wood GW, Bratan T, *et al.* Patients' attitudes to the summary care record and HealthSpace: qualitative study. *BMJ* 2008;**336**:1290–5. doi:10.1136/bmj.a114

44 Haddow G, Bruce A, Sathanandam S, *et al.* 'Nothing is really safe': a focus group study on the processes of anonymizing and sharing of health data for research purposes. *J Eval Clin Pract* 2011;**17**:1140–6. doi:10.1111/j.1365-2753.2010.01488.x

45 Stevenson F. The use of electronic patient records for medical research: conflicts and contradictions. *BMC Health Serv Res* 2015;**15**:124. doi:10.1186/s12913-015-0783-6

46 Atkin C, Crosby B, Dunn K, *et al.* Perceptions of anonymised data use and awareness of the NHS data opt-out amongst patients, carers and healthcare staff. *Res Involv Engagem* 2021;**7**:40. doi:10.1186/s40900-021-00281-2

47 The Caldicott Committee. Report on the Review of Patient-Identifiable Information. 1997.https://webarchive.nationalarchives.gov.uk/20130124064947/http://www.dh.gov.uk/prod_consum_dh/groups/dh_digitalassets/@dh/@en/documents/digitalasset/dh_4068404.pdf

For peer review only - http://bmjopen.bmj.com/site/about/guidelines.xhtml

48 UK. Health and Social Care (Safety and Quality) Act 2015. 2015. http://www.legislation.gov.uk/ukpga/2015/28/contents

49 nef (The New Economics Foundation), Centre for Science Education at Sheffield Hallam University. Who sees what: exploring public views on personal electronic health records. 2010. https://neweconomics.org/uploads/files/2cb17ab59382fe7c67_bfm6bdoas.pdf

50 NHS. National Data Opt-out: Factsheet 2 – When it applies. 2018.https://digital.nhs.uk/binaries/content/assets/website-assets/services/national-data-opt-out-programme/guidance-for-health-and-care-staff/nhs-factsheet-2-23052018.pdf (accessed 26 Jul 2019).

51 NHS Digital. Understanding the national data opt-out. 2019.https://web.archive.org/web/20200804083843/https://digital.nhs.uk/services/national-data-opt-out/understanding-the-national-data-opt-out

52 UK. The Health Service (Control of Patient Information) Regulations 2002. 2002. http://www.legislation.gov.uk/uksi/2002/1438/contents/made

53 Beange I, Kirkham EJ, Fletcher-Watson S, *et al.* Using a knowledge exchange event to assess study participants' attitudes to research in a rapidly evolving research context. *Wellcome Open Res* 2020;**5**:24. doi:10.12688/wellcomeopenres.15651.2

54 Chico V, Hunn A, Taylor M. Public views on sharing anonymised patient-level data where there is a mixed public and private benefit. Health Research Authority; University of Sheffield School of Law 2019.

55 Helm T. Revealed: how drugs giants can access your health records. *The Guardian* 2020.https://www.theguardian.com/technology/2020/feb/08/fears-over-sale-anonymous-nhs-patient-data (accessed 8 Feb 2020).

56 Gazard B, Chui Z, Harber-Aschan L, *et al.* Barrier or stressor? The role of discrimination experiences in health service use. *BMC Public Health* 2018;**18**:1354. doi:10.1186/s12889-018-6267-y

57 Wu Z, Schimmele CM. Perceived religious discrimination and mental health. *Ethn Health* 2019;:1–18. doi:10.1080/13557858.2019.1620176

58 Luchenski SA, Reed JE, Marston C, *et al.* Patient and public views on electronic health records and their uses in the United kingdom: cross-sectional survey. *J Med Internet Res* 2013;**15**:e160. doi:10.2196/jmir.2701

59 Haeusermann T, Greshake B, Blasimme A, *et al.* Open sharing of genomic data: Who does it and why? *PloS One* 2017;**12**:e0177158. doi:10.1371/journal.pone.0177158

60 SteelFisher GK, Findling MG, Bleich SN, *et al.* Gender discrimination in the United States: Experiences of women. *Health Serv Res* 2019;**54 Suppl 2**:1442–53. doi:10.1111/1475-6773.13217

61 Lyons RA, Jones KH, John G, *et al.* The SAIL databank: linking multiple health and social care datasets. *BMC Med Inform Decis Mak* 2009;**9**:3. doi:10.1186/1472-6947-9-3

62 Lyons J, Akbari A, Torabi F, *et al.* Understanding and responding to COVID-19 in Wales: protocol for a privacy-protecting data platform for enhanced epidemiology and evaluation of interventions. *BMJ Open* 2020;**10**:e043010. doi:10.1136/bmjopen-2020-043010

63 Hagger-Johnson G. Opportunities for longitudinal data linkage in Scotland. *Scott Med J* 2016;**61**:136–45. doi:10.1177/0036933015575214

For peer review only

For peer review only - http://bmjopen.bmj.com/site/about/guidelines.xhtml

64 Thornicroft G, Mehta N, Clement S, *et al.* Evidence for effective interventions to reduce mental-health-related stigma and discrimination. *Lancet Lond Engl* 2016;**387**:1123–32. doi:10.1016/S0140-6736(15)00298-6

65 Clement S, Schauman O, Graham T, *et al.* What is the impact of mental health-related stigma on help-seeking? A systematic review of quantitative and qualitative studies. *Psychol Med* 2015;**45**:11–27. doi:10.1017/S0033291714000129

66 Gary FA. Stigma: barrier to mental health care among ethnic minorities. *Issues Ment Health Nurs* 2005;**26**:979–99. doi:10.1080/01612840500280638

67 Sangill C, Buus N, Hybholt L, *et al.* Service user's actual involvement in mental health research practices: A scoping review. *Int J Ment Health Nurs* 2019;**28**:798–815. doi:10.1111/inm.12594

68 UK. Equality Act 2010. 2010.https://www.legislation.gov.uk/ukpga/2010/15/contents (accessed 5 Jul 2021).

69 Tully MP, Bozentko K, Clement S, *et al.* Investigating the Extent to Which Patients Should Control Access to Patient Records for Research: A Deliberative Process Using Citizens' Juries. *J Med Internet Res* 2018;**20**:e112. doi:10.2196/jmir.7763

70 Sakshaug JW, Schmucker A, Kreuter F, *et al.* The Effect of Framing and Placement on Linkage Consent. *Public Opin Q* 2019;**83**:289–308. doi:10.1093/poq/nfz018

71 Gollust SE, Fowler EF, Niederdeppe J. Television News Coverage of Public Health Issues and Implications for Public Health Policy and Practice. *Annu Rev Public Health* 2019;**40**:167–85. doi:10.1146/annurev-publhealth-040218-044017

72 Ford E, Kazempour Y, Cooper MJF, *et al.* Media content analysis of general practitioners' reactions to care.data expressed in the media: what lessons can be learned for future NHS data-sharing initiatives? *BMJ Open* 2020;**10**:e038006. doi:10.1136/bmjopen-2020-038006

73 Prime Minister's Office. PM address to the nation on coronavirus: 23 March 2020. 2020.https://www.gov.uk/government/speeches/pm-address-to-the-nation-on-coronavirus-23-march-2020 (accessed 23 Mar 2020).

74 RECOVERY Collaborative Group, Horby P, Lim WS, *et al.* Dexamethasone in Hospitalized Patients with Covid-19. *N Engl J Med* 2021;**384**:693–704. doi:10.1056/NEJMoa2021436

75 Horby P, Lim WS, Emberson J, *et al.* Effect of Dexamethasone in Hospitalized Patients with COVID-19 – Preliminary Report. *medRxiv* 2020;:2020.06.22.20137273. doi:10.1101/2020.06.22.20137273

76 Elwood JM, Marshall RJ, Tin ST, *et al.* Bias in survival estimates created by a requirement for consent to enter a clinical breast cancer registry. *Cancer Epidemiol* 2019;**58**:178–83. doi:10.1016/j.canep.2018.12.005

77 Morgan K, Page N, Brown R, *et al.* Sources of potential bias when combining routine data linkage and a national survey of secondary school-aged children: a record linkage study. *BMC Med Res Methodol* 2020;**20**:178. doi:10.1186/s12874-020-01064-1

78 Kariotis T, Ball MP, Greshake Tzovaras B, *et al.* Emerging Health Data Platforms: From Individual Control to Collective Data Governance. Published Online First: 2020. doi:10.1017/dap.2020.14

For peer review only - http://bmjopen.bmj.com/site/about/guidelines.xhtml

79 Wyatt D, Lampon S, McKevitt C. Delivering healthcare's 'triple aim': electronic health records and the health research participant in the UK National Health Service. *Sociol Health Illn* 2020;**42**:1312–27. doi:10.1111/1467-9566.13101

80 NHSX. Information Governance Framework for Integrated Health and Care: Shared Care Records. 2021.https://www.nhsx.nhs.uk/media/documents/NHSX_IG_Framework_V6.pdf (accessed 5 Feb 2022).

81 UK Office for National Statistics. ONS Secure Research Service (SRS). 2019.https://www.ons.gov.uk/aboutus/whatwedo/paidservices/virtualmicrodatalaboratoryvml (accessed 23 Jul 2019).

82 Stewart R, Soremekun M, Perera G, *et al.* The South London and Maudsley NHS Foundation Trust Biomedical Research Centre (SLAM BRC) case register: development and descriptive data. *BMC Psychiatry* 2009;**9**:51. doi:10.1186/1471-244X-9-51

83 UK Health Data Research Alliance. Trusted research environments (TRE): A strategy to build public trust and meet changing health data science needs. 2020.https://ukhealthdata.org/wp-content/uploads/2020/07/200723-Alliance-Board_Paper-E_TRE-Green-Paper.pdf (accessed 4 Feb 2022).

84 Griffiths E, Greci C, Kotrotsios Y, *et al.* Handbook on Statistical Disclosure Control for Outputs. 2019.https://ukdataservice.ac.uk/media/622521/thf_datareport_aw_web.pdf

85 Fernandes AC, Cloete D, Broadbent MTM, *et al.* Development and evaluation of a de-identification procedure for a case register sourced from mental health electronic records. *BMC Med Inform Decis Mak* 2013;**13**:71. doi:10.1186/1472-6947-13-71

86 Cardinal RN. Clinical records anonymisation and text extraction (CRATE): an open-source software system. *BMC Med Inform Decis Mak* 2017;**17**:50. doi:10.1186/s12911-017-0437-1

87 Ballantyne A, Schaefer GO. Consent and the ethical duty to participate in health data research. *J Med Ethics* 2018;**44**:392–6. doi:10.1136/medethics-2017-104550

88 Williamson EJ, Walker AJ, Bhaskaran K, *et al.* Factors associated with COVID-19-related death using OpenSAFELY. *Nature* 2020;**584**:430–6. doi:10.1038/s41586-020-2521-4

89 Wolfson M, Wallace SE, Masca N, *et al.* DataSHIELD: resolving a conflict in contemporary bioscience--performing a pooled analysis of individual-level data without sharing the data. *Int J Epidemiol* 2010;**39**:1372–82. doi:10.1093/ije/dyq111

90 Gaye A, Marcon Y, Isaeva J, *et al.* DataSHIELD: taking the analysis to the data, not the data to the analysis. *Int J Epidemiol* 2014;**43**:1929–44. doi:10.1093/ije/dyu188

91 Fontana G, Ghafur S, Torne L, *et al.* Ensuring that the NHS realises fair financial value from its data. *Lancet Digit Health* 2020;**2**:e10–2. doi:10.1016/S2589-7500(19)30225-0

92 Carter P, Laurie GT, Dixon-Woods M. The social licence for research: why care.data ran into trouble. *J Med Ethics* 2015;**41**:404–9. doi:10.1136/medethics-2014-102374

93 UK Department of Health and Social Care. Data saves lives: reshaping health and social care with data (draft). GOV.UK. 2021.https://www.gov.uk/government/publications/data-saves-lives-reshaping-health-and-social-care-with-data-draft (accessed 6 Jul 2021).

For peer review only - http://bmjopen.bmj.com/site/about/guidelines.xhtml

94 UK Department for Business, Energy & Industrial Strategy. Life Sciences Vision (policy paper). 2021.https://assets.publishing.service.gov.uk/government/uploads/system/uploads/attachment_data/file/1000030/life-sciences-vision.pdf

95 Higher Education Funding Council for England, Research Councils UK, Universities UK, *et al.* Concordat on Open Research Data. 2016.https://www.ukri.org/files/legacy/documents/concordatonopenresearchdata-pdf/

[dataset] 96 Jones LA, Nelder JR, Fryer JM, *et al.* Data for: Public opinion on sharing data from health services for clinical and research purposes without explicit consent: an anonymous online survey in the UK. University of Cambridge Data Repository, 16 July 2021. doi:10.17863/CAM.75784

For peer review only

13. FIGURE LEGENDS

Figure 1. Classifying health data according to identifiability. At the "patient" level, the Venn diagram shows the overlaps between information that directly identifies a person, research identifiers (RIDs or pseudonyms), and health information, with simple examples. Anonymous health data may also existing in aggregated form, distinct from patient-level data; this aggregated form is the norm for public distribution. The level of identification risk and the research information governance requirements vary with the resulting categories of data, shown in the legend. All examples are fictional. (DOB, date of birth.)

Figure 2. (A) Understanding of how health data are shared identifiably without explicit consent for clinical purposes, and **(B)** preference as to what should happen. The denominator for percentages is the number of people who answered each question, shown at the bottom right of each panel.

Figure 3. Participants' self-reported likelihood of sharing mental and physical health data without explicit consent for clinical purposes (identifiably) or research (de-identified), according to destination, nature (MH versus PH), framing statement, and for research purposes also the level of detail (structured only versus with free text). The denominator for percentages is the number of people who answered each question. In plots C, F, and H, the abscissa is the mean of responses coded as –2 very unlikely, –1 unlikely, 0 not sure, +1 likely, +2 very likely. Analyses were from models C1 and R1 as described in the Methods. (De-id., de-identified; MH, mental health; PH, physical health; #####, $p<10^{-5}$ for main effect of destination; †††††, $p<10^{-5}$ for main effect of nature, with bar length showing mean difference between MH and PH; ** $p<0.01$ and ***** $p<10^{-5}$ for framing×nature interaction, with bar showing the mean difference between "MH concern" and "neutral"; §§§§§, $p<10^{-5}$, main effect of detail, comparing panel F with panel I, with bar length showing the mean difference between structured and free-text conditions.) See **Supplementary Figure 4** for corresponding weighted analysis.

Figure 4. Participants' willingness for their NHS data to be linked to non-health data of different kinds for research. The denominator for percentages is the number of people who answered each question. See **Supplementary Figure 5** for corresponding weighted analysis.

Figure 5. Change in preference in relation to the COVID-19 pandemic. **(A)** Clinical/identifiable data sharing, by destination. Dependent variable (preference for sharing) as for **Figure 3C**, now shown on the ordinate (y) axis. **(B)** Research/de-identified data sharing, by detail (structured versus free text) and destination. Dependent variable as for **Figure 3F,H**. **(C)** Linkage for research, by non-NHS data source type. Dependent variable coded as –1 no, 0 not sure, +1 yes. (Error bars show ±1 SEM; ***** $p<10^{-5}$, **** $p<10^{-4}$, *** $p<10^{-3}$, ** $p<10^{-2}$, * $p<0.05$ by two-sample t test Šidák-corrected for multiple comparisons; NS, not significant; ##### $p \lll 10^{-5}$, destination×pandemic interaction.)

Figure 6. Effect sizes for **(A)** clinical data sharing via statistical model C2, **(B)** research data sharing via model R2, and **(C)** linkage via model L2. These models include only those participants who supplied full demographic information, to allow analysis by demographics; compare **Supplementary Figure 6** (all participants). Only those model terms with a significant F test are shown. Effect sizes with 95% confidence intervals are shown for each level as uncorrected pairwise comparisons to a reference category within each term (note the difference in what is being tested pairwise versus the omnibus F test for the term; see **Supplementary Methods**). ● $p<\alpha$; ○ NS.

Figure 7. Views on a national data sharing consent system. The denominator for percentages is the number of people who answered each question. See **Supplementary Figure 7** for corresponding weighted analysis.

For peer review only

For peer review only - http://bmjopen.bmj.com/site/about/guidelines.xhtml

Figure 1

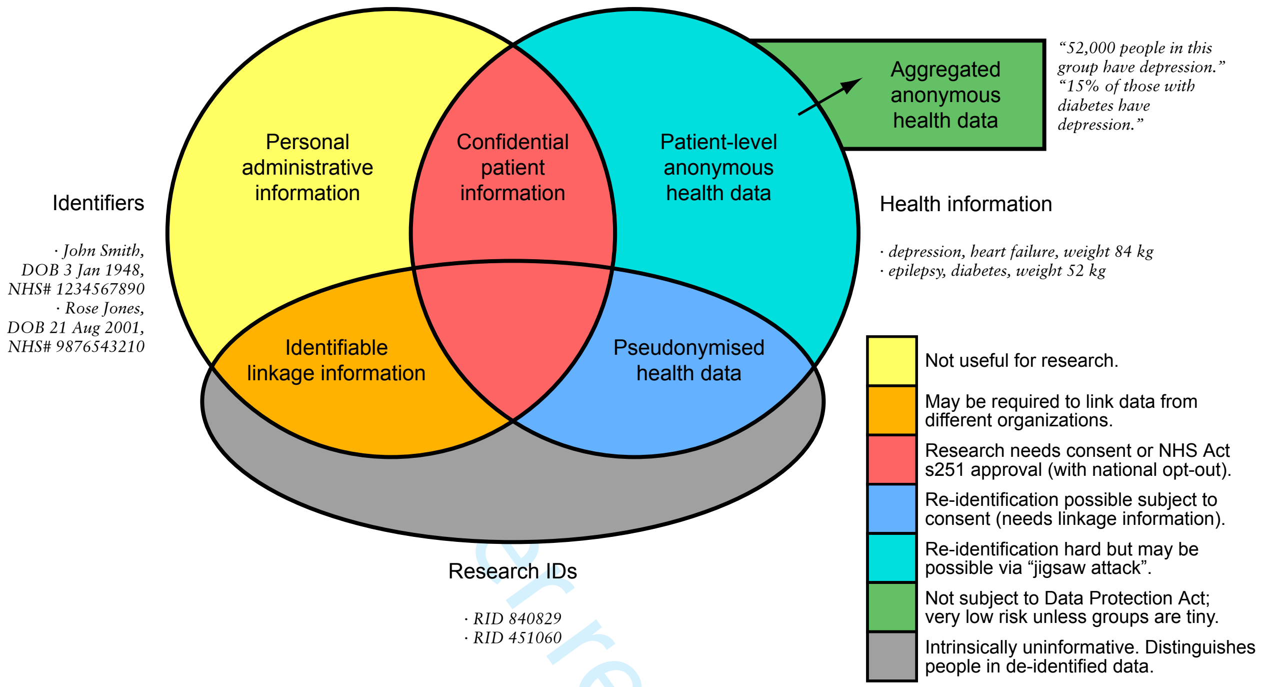
Personal administrative information
Confidential patient information
Patient-level anonymous health data
Aggregated anonymous health data
"52,000 people in this group have depression." "15% of those with diabetes have depression."
Identifiers
· John Smith, DOB 3 Jan 1948, NHS# 1234567890
· Rose Jones, DOB 21 Aug 2001, NHS# 9876543210
Health information
· depression, heart failure, weight 84 kg
· epilepsy, diabetes, weight 52 kg
Identifiable linkage information
Pseudonymised health data
Research IDs
· RID 840829
· RID 451060
Not useful for research.
May be required to link data from different organizations.
Research needs consent or NHS Act s251 approval (with national opt-out).
Re-identification possible subject to consent (needs linkage information).
Re-identification hard but may be possible via "jigsaw attack".
Not subject to Data Protection Act; very low risk unless groups are tiny.
Intrinsically uninformative. Distinguishes people in de-identified data.

Figure 2

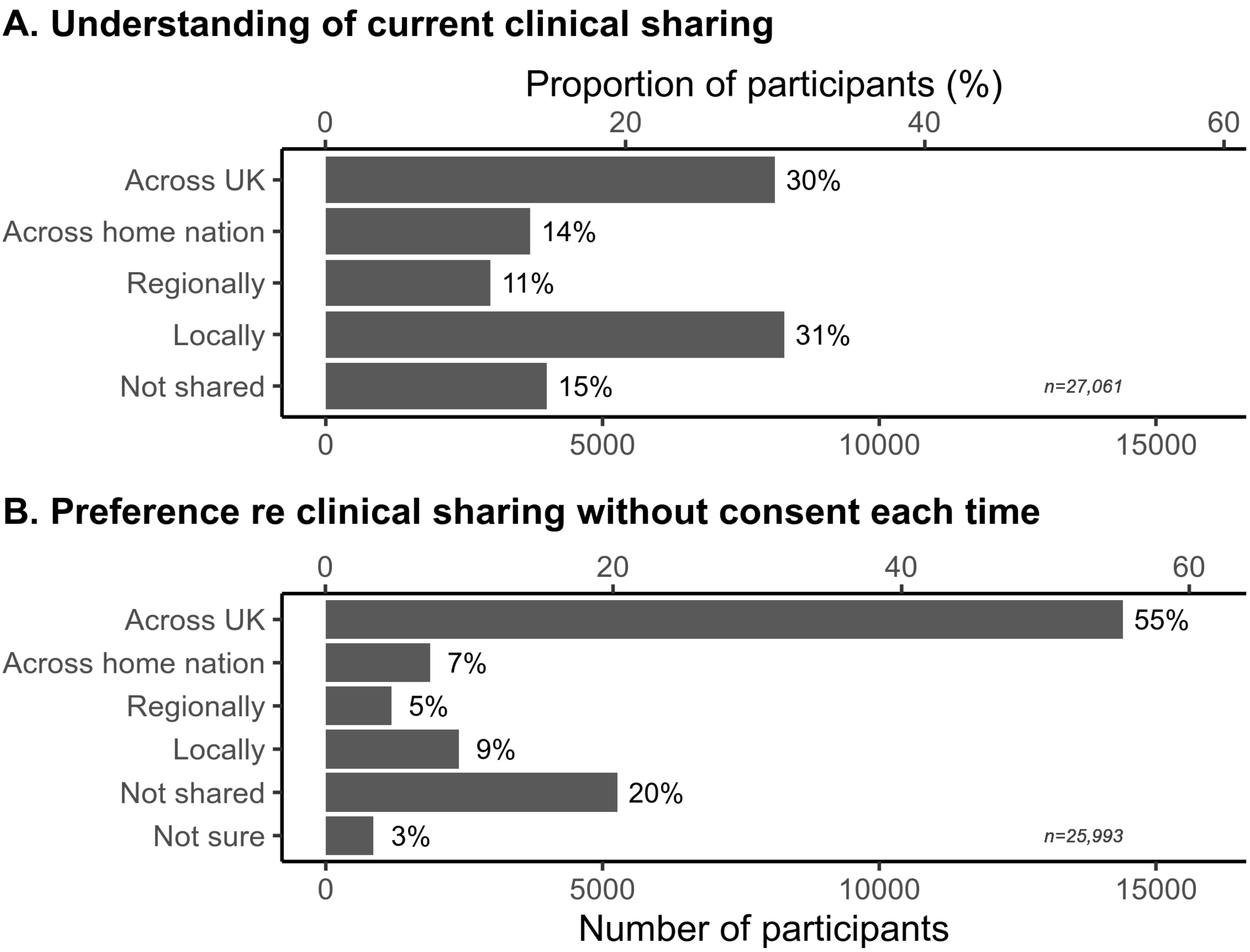
A. Understanding of current clinical sharing
Proportion of participants (%)
0
20
40
60
Across UK
Across home nation
Regionally
Locally
Not shared
30%
14%
11%
31%
15%
n=27,061
0
5000
10000
15000
B. Preference re clinical sharing without consent each time
0
20
40
60
Across UK
Across home nation
Regionally
Locally
Not shared
Not sure
55%
7%
5%
9%
20%
3%
n=25,993
0
5000
10000
15000
Number of participants

Figure 3

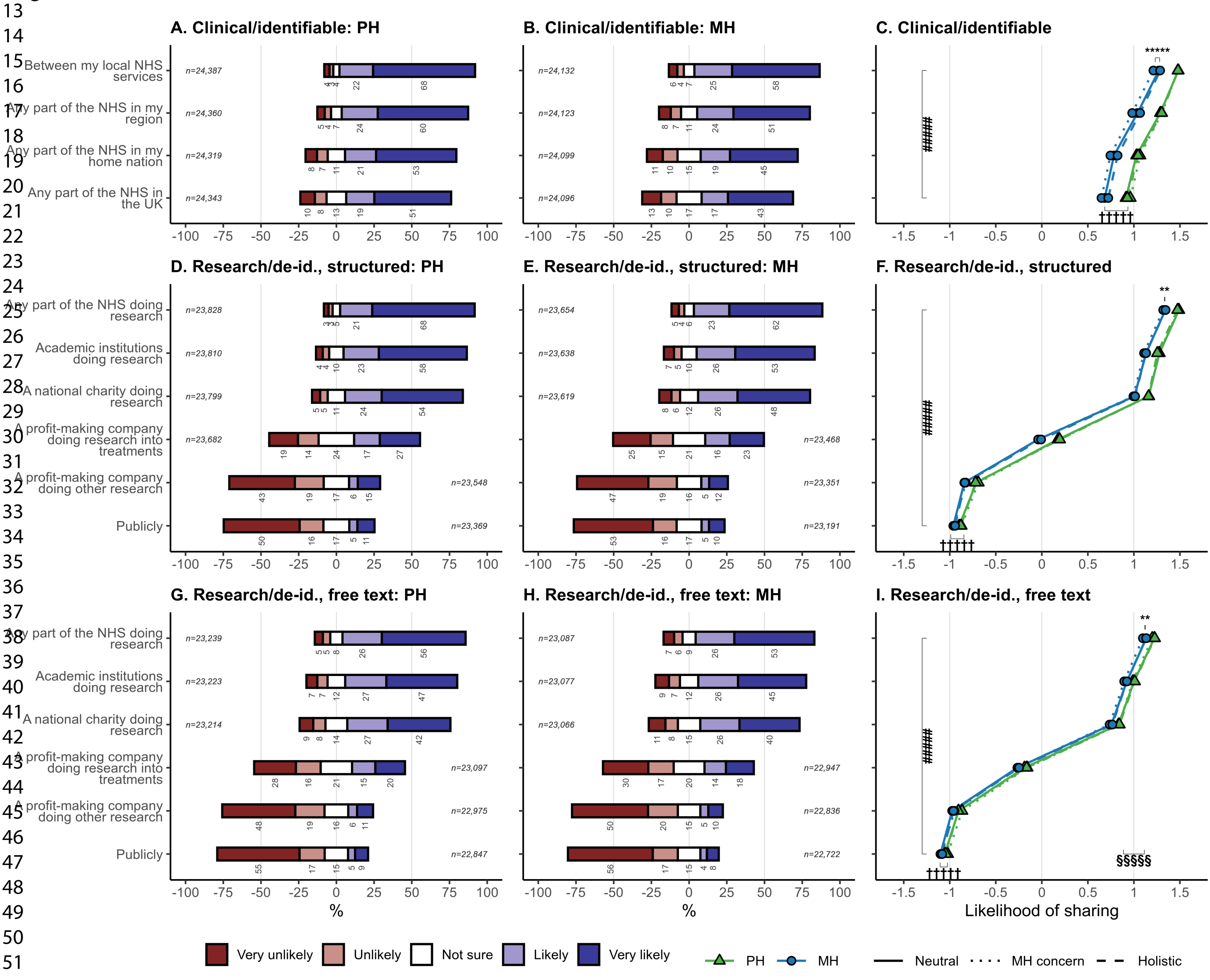
A. Clinical/identifiable: PH
B. Clinical/identifiable: MH
C. Clinical/identifiable
D. Research/de-id., structured: PH
E. Research/de-id., structured: MH
F. Research/de-id., structured
G. Research/de-id., free text: PH
H. Research/de-id., free text: MH
I. Research/de-id., free text
Between my local NHS services
Any part of the NHS in my region
Any part of the NHS in my home nation
Any part of the NHS in the UK
Any part of the NHS doing research
Academic institutions doing research
A national charity doing research
A profit-making company doing research into treatments
A profit-making company doing other research
Publicly
%
Likelihood of sharing
Very unlikely
Unlikely
Not sure
Likely
Very likely
PH
MH
Neutral
MH concern
Holistic

Figure 4

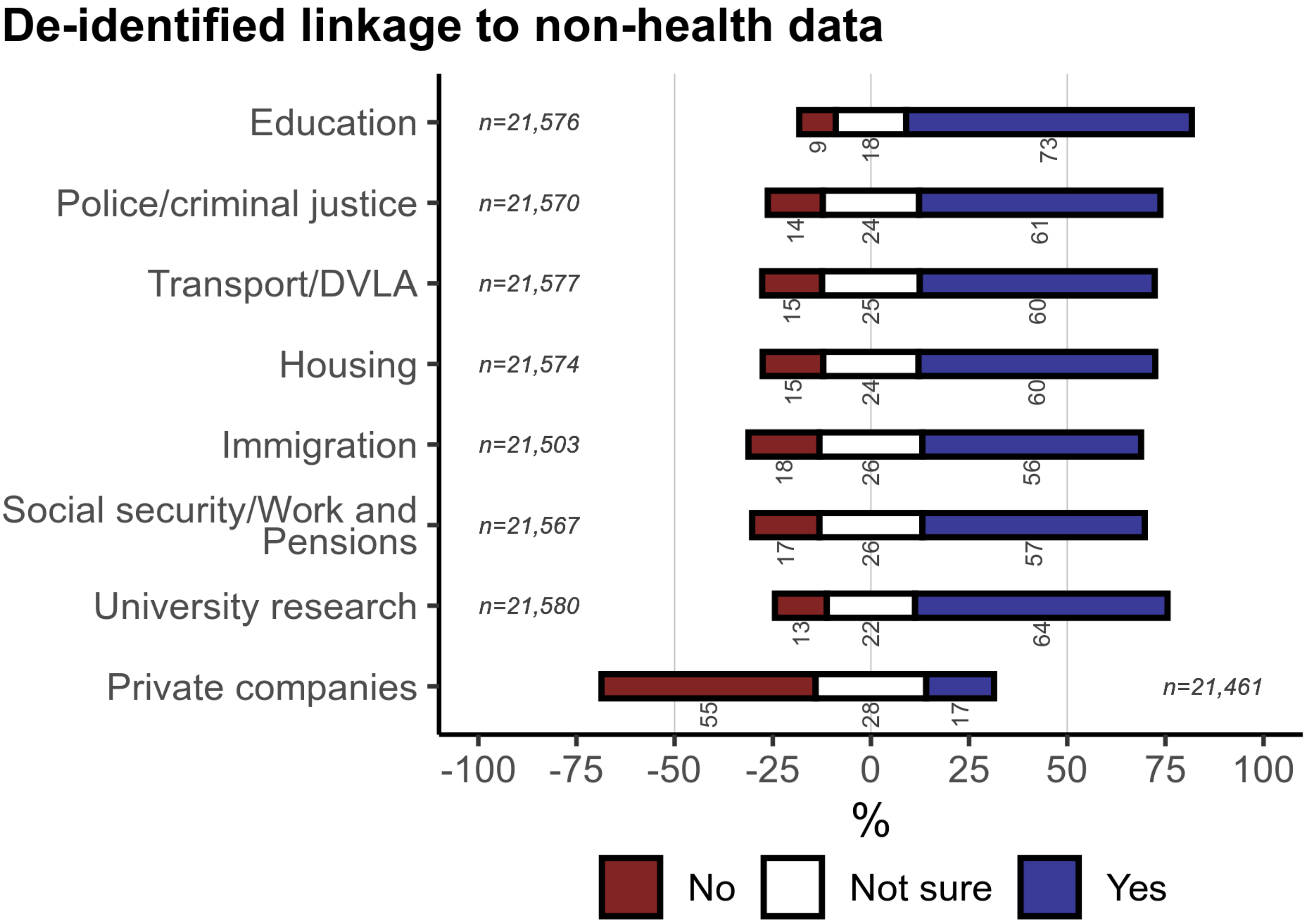
De-identified linkage to non-health data
Education
n=21,576
9
18
73
Police/criminal justice
n=21,570
14
24
61
Transport/DVLA
n=21,577
15
25
60
Housing
n=21,574
15
24
60
Immigration
n=21,503
18
26
56
Social security/Work and Pensions
n=21,567
17
26
57
University research
n=21,580
13
22
64
Private companies
n=21,461
55
28
17
-100
-75
-50
-25
0
25
50
75
100
%
No
Not sure
Yes

Figure 5

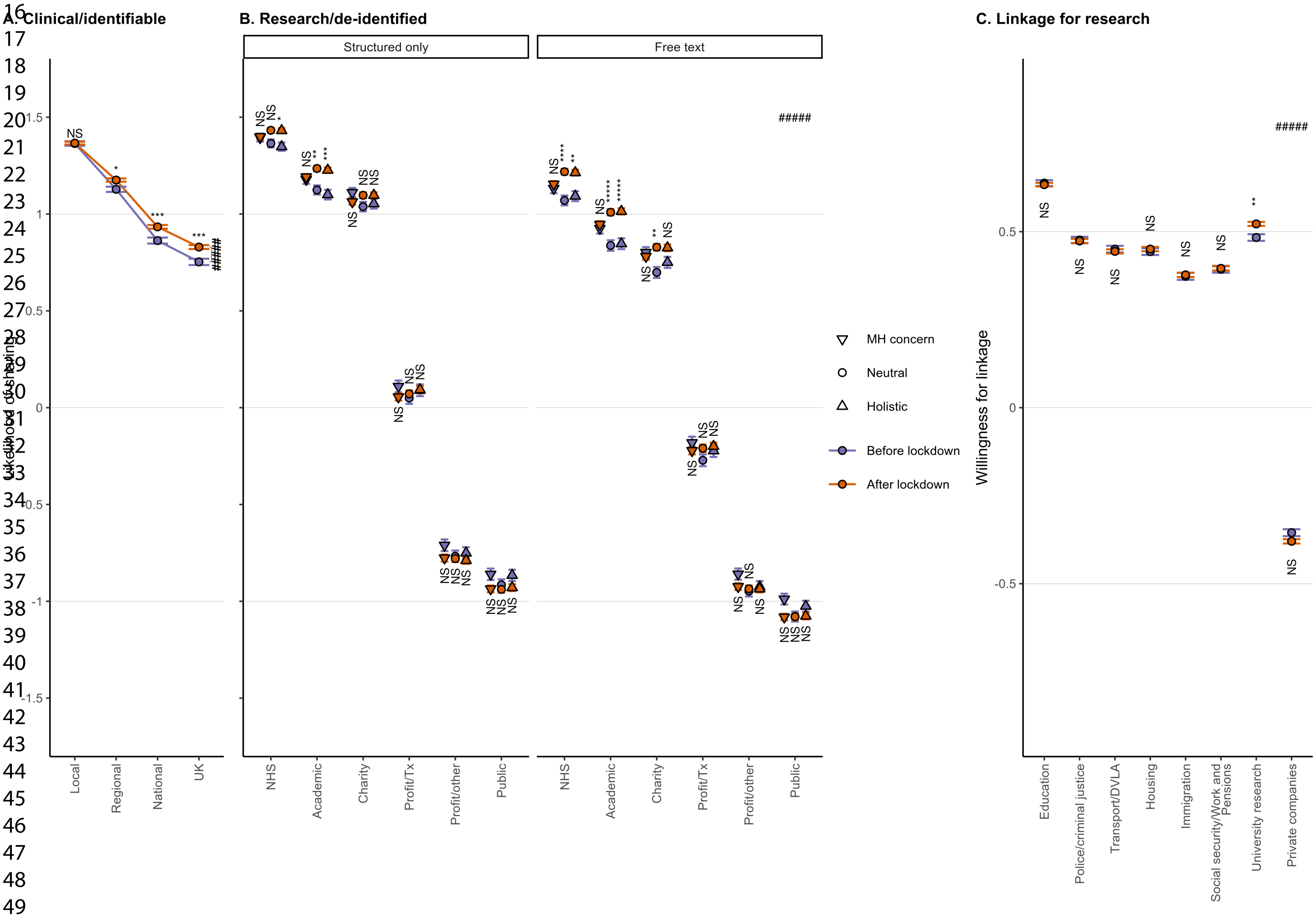
A. Clinical/identifiable
B. Research/de-identified
C. Linkage for research
Structured only
Free text
Likelihood of sharing
Willingness for linkage
Local
Regional
National
UK
NHS
Academic
Charity
Profit/Tx
Profit/other
Public
Education
Police/criminal justice
Transport/DVLA
Housing
Immigration
Social security/Work and Pensions
University research
Private companies
MH concern
Neutral
Holistic
Before lockdown
After lockdown
#####

Figure 6

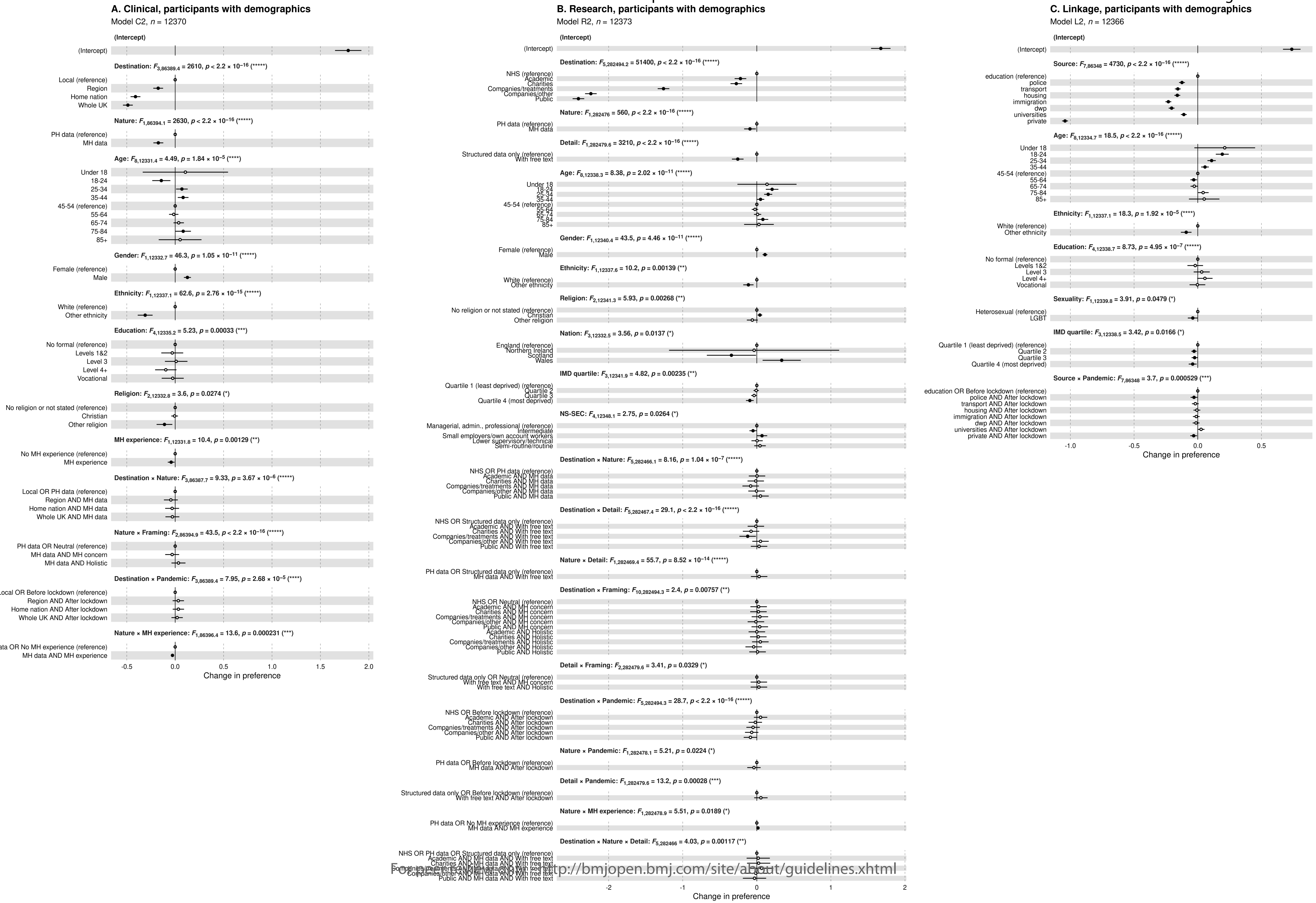

For peer review only - http://bmjopen.bmj.com/site/about/guidelines.xhtml

Figure 7

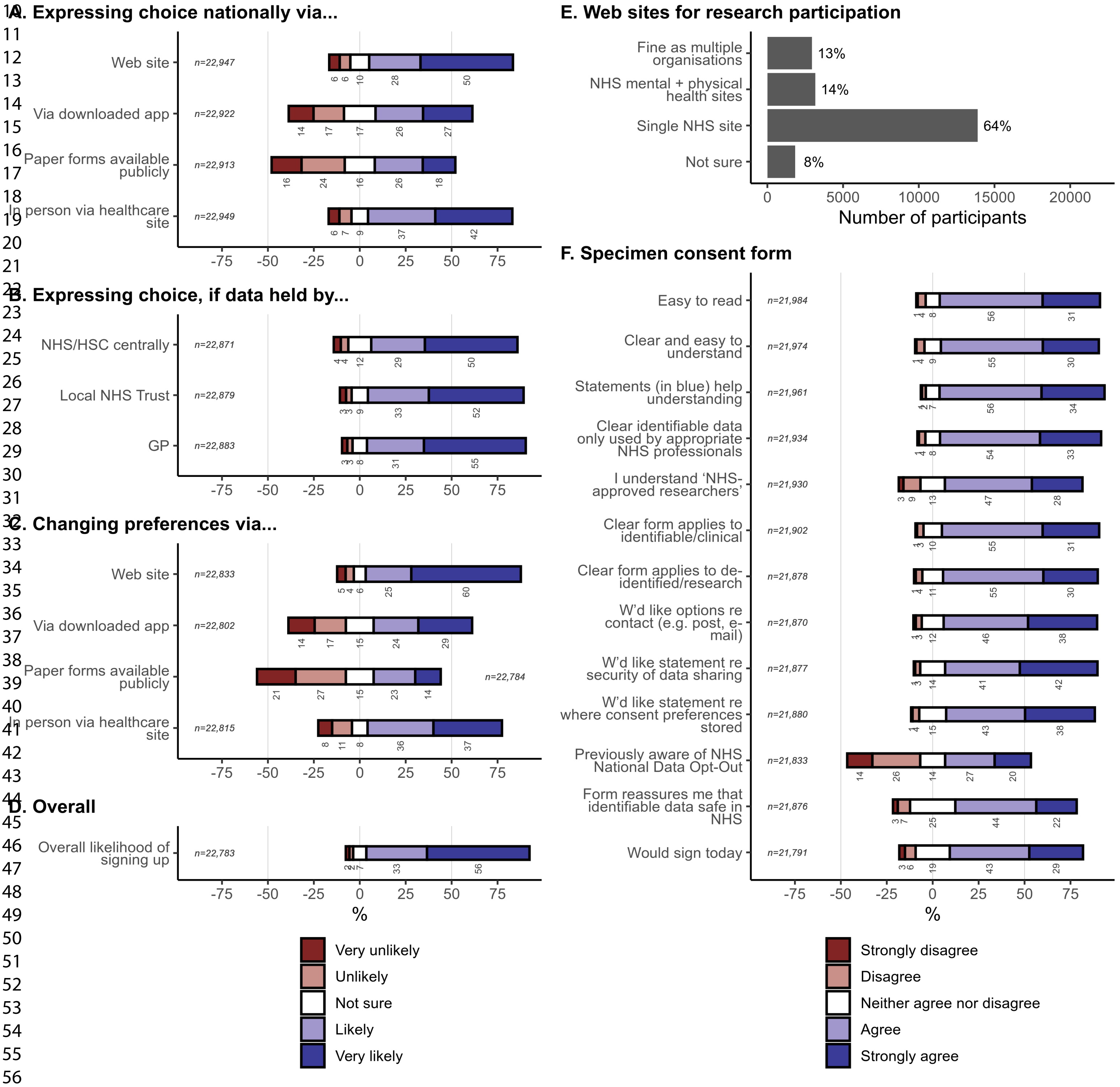
A. Expressing choice nationally via...
Web site
n=22,947
Via downloaded app
n=22,922
Paper forms available publicly
n=22,913
In person via healthcare site
n=22,949
B. Expressing choice, if data held by...
NHS/HSC centrally
n=22,871
Local NHS Trust
n=22,879
GP
n=22,883
C. Changing preferences via...
Web site
n=22,833
Via downloaded app
n=22,802
Paper forms available publicly
n=22,784
In person via healthcare site
n=22,815
D. Overall
Overall likelihood of signing up
n=22,783
-75 -50 -25 0 25 50 75
%
Very unlikely
Unlikely
Not sure
Likely
Very likely
E. Web sites for research participation
Fine as multiple organisations
13%
NHS mental + physical health sites
14%
Single NHS site
64%
Not sure
8%
0 5000 10000 15000 20000
Number of participants
F. Specimen consent form
Easy to read
n=21,984
Clear and easy to understand
n=21,974
Statements (in blue) help understanding
n=21,961
Clear identifiable data only used by appropriate NHS professionals
n=21,934
I understand 'NHS-approved researchers'
n=21,930
Clear form applies to identifiable/clinical
n=21,902
Clear form applies to de-identified/research
n=21,878
W'd like options re contact (e.g. post, e-mail)
n=21,870
W'd like statement re security of data sharing
n=21,877
W'd like statement re where consent preferences stored
n=21,880
Previously aware of NHS National Data Opt-Out
n=21,833
Form reassures me that identifiable data safe in NHS
n=21,876
Would sign today
n=21,791
-75 -50 -25 0 25 50 75
%
Strongly disagree
Disagree
Neither agree nor disagree
Agree
Strongly agree

SUPPLEMENTARY MATERIALS

Public opinion on sharing data from health services for clinical and research purposes without explicit consent: an anonymous online survey in the UK

Jones et al.

Contents

S1. Supplementary Methods 2
S1.1. Patient and public involvement in the study
S1.2. Survey content
S1.3. Analysis of geography including Index of Multiple Deprivation
S1.4. Sensitivity analysis via survey weighting
S1.5. Effect size plots
S1.6. Free-text values and comments
S2. Supplementary Results 6
S2.1. Recruitment and completion
S2.2. Demographics
S2.3. Experience
S2.4. Raking
S2.5. Weighted analysis of sharing preferences
S2.6. Weighted views on linkage to non-health data for research
S2.7. Effect sizes
S2.8. Weighted views on a national data sharing consent system
S2.9. Free-text comments on the suggested consent form
S2.10. Other free-text comments
S3. Supplementary Discussion 10
S3.1. Under-represented groups
S3.2. UK legal basis for using NHS data for clinical or research purposes
S4. Supplementary Tables 15
S5. Supplementary References 16
S6. List of appendices 22
S7. Supplementary Figures
Appendix A: Underlying REDCap survey design
Appendix B: Survey
Appendix C: STROBE statement for cross-sectional studies

bmjopen-2021-057579; version of 2022-02-13

For peer review only

S1. SUPPLEMENTARY METHODS

S1.1. Patient and public involvement in the study

Recruitment and operation of a research advisory group (RAG). The research team designed an advertisement explaining the project, and a role profile, which were circulated through our local longstanding patient/public involvement (PPI) groups across our region. There were no specific requirements other than to have had personal experience of a mental health condition or experience of caring for someone who had, and an interest in the project. After receiving applications, the PPI lead met all applicants to discuss further and to answer any questions. Of nine original applicants, five became involved as part of the RAG in person and one was involved remotely providing advice. The RAG were invited to a meeting at which the research team presented the broad scope of the clinical informatics work in which they were involved (known as the Clinical Informatics for Mind and Brain Health [CLIMB] project, funded via Medical Research Council [MRC] Mental Health Data Pathfinder grant MC_PC_17213). Subsequently, the research team and RAG continued their association through meetings, e-mails, and phone calls, though in-person meetings were curtailed after the COVID-19 pandemic arrived in the UK.

Survey design and piloting. The RAG and research team jointly produced the questions for the survey project at face-to-face meetings for this purpose. The research team explained their objectives and the RAG contributed their own, helped formulate the questions needed, and helped to design the survey content. This took several group meetings plus additional one-to-one meetings and e-mail contacts. RAG members added questions and removed some suggested by the research team that they disliked, particularly revising the survey sections on personal characteristics and the suggested/draft consent form. The RAG spent considerable time helping with revisions to the language of the project, as they felt the initial drafts were too verbose and "scientifically" phrased. Once the first draft was ready and implemented electronically, the RAG were instrumental in testing it themselves and via their family, friends, and colleagues, providing feedback that the research team incorporated to shape the final survey. Once finalized, the research team and RAG piloted the survey again to estimate the time it would take to complete (estimated at 18–25 minutes, the figure used subsequently as part of the research ethics application). The RAG also helped to design and revise the materials used to publicize the study.

Survey participation. Patients, carers, and other members of the public participated in the survey.

Dissemination. After initial analysis, the research team briefed the RAG on the results. The research team and RAG co-wrote the lay summary of the results, which was sent to all participants who had left an e-mail address for this purpose. The research team drafted the present manuscript and some members of the RAG chose to participate as authors, editing and critically revising the content.

S1.2. Survey content

The survey sequence is shown in full in the **Appendix A** and **Appendix B.** In outline, it was as follows:

- Information, consent, and a question how the participant heard about the survey. (Participants were asked to indicate the health organization, if any, through which they had heard about the study and recruitment was attributed to those sites.)
- An explanation of "NHS" (used as shorthand for NHS/HSC), "health data", "identifiable health data", and "clinical care".
- Questions about **current and desired sharing of identifiable health data for clinical care purposes.**
  - A multiple-choice question on the respondent's understanding of how NHS clinical care providers *currently* share their identifiable data for clinical care purposes, without asking each time.
  - A statement that most NHS providers are separate and do not currently share identifiable data without asking, followed by a multiple-choice question as to if/how the NHS *should* share the respondent's identifiable data for clinical care purposes, without asking each time (with all stems ending "… without asking me first").
- Questions about **personal experience** of health conditions.
  - Whether the respondent had or hadn't experienced a mental health (MH) condition at some point (or a prefer-not-to-say option). If answered positively, categorical questions about what sort of condition that was, and whether it was recent or >5 years ago, and what sort of support had been obtained (e.g. from NHS or other sources).
  - Whether the respondent had ever used physical health services, and if so, which categories.
- Fictional examples of **identifiable health data** were presented. These included identifiers, diagnoses, and notes.

For peer review only

- A **framing statement** was presented, randomized to be one of the following:
  - *Neutral:* "We would like to find out your perspective on using information about your mental health and your physical health."
  - *MH concern:* "Previous surveys have found that people have more concerns about the use of their identifiable health data relating to their mental health than other aspects of their physical health care." This statement was based on previous findings.[1]
  - *Holistic:* "Mental and physical illnesses overlap, so holistic health care is important. Mental health problems have physical consequences, and physical illnesses have important consequences for mental health." This statement is also true (e.g. [2,3]).
- Likert-style questions about how likely the respondent would be to share their *identifiable physical health* data, for *clinical care* purposes, with a range of NHS "destinations", without being asked each time.
- Likert-style questions about how likely the respondent would be to share their *identifiable mental health* data, for *clinical care* purposes, with a range of NHS destinations, without being asked each time.
- Fictional examples of **de-identified structured health data** were presented (the same examples as before, now de-identified, and without any free text). These included alphanumeric research identifiers, "blurred" demographics (age, sex, geographical region), and diagnoses.
- Likert-style questions about how likely the respondent would be to share their *de-identified (structured) physical health* data with a range of research destinations, without giving consent each time.
- Likert-style questions about how likely the respondent would be to share their *de-identified (structured) mental health* data with a range of research destinations, without giving consent each time.
- Fictional examples of **de-identified free-text health data** were presented. These were the same de-identified data as before, but now with de-identified versions of the free-text notes. Explicit commentary was given that there was more information present, and a slightly higher risk of inadvertent identification. We gave the example of a hypothetical newspaper report that might enable someone to re-identify a patient.
- Likert-style questions about how likely the respondent would be to share their *de-identified (free text) physical health* data with a range of research destinations, without giving consent each time.
- Likert-style questions about how likely the respondent would be to share their *de-identified (free text) mental health* data with a range of research destinations, without giving consent each time.
- An explicit change of topic was noted.
- We asked Likert-style questions about how likely people would be to sign up to a **single system for controlling how one's NHS data is used** (clinically and for research), for a variety of types of system (e.g. online, in person), who should look after such consent-related data, what the respondent's preferred method would for changing their preferences, and overall how likely they would be to sign up to such a system.
- We showed a **specimen consent form** for such a system (see below for full details).
  - First, the specimen form asked about sharing data for direct health care purposes.
    - It defined "confidential patient information" [4], and set the context in terms of health care being provided directly to the respondent.
    - It offered a yes/no decision: "I agree that all NHS care providers and professionals may share my confidential patient information with each other for the purposes of my treatment and care."
  - Second, the specimen form asked about the use of de-identified data for research.
    - It provided brief information about the NHS's promises to use anonymised data for research (e.g. [5,6]). It said that research was conducted by the NHS and by NHS-approved researchers such as universities. It referred to strict security controls and NHS oversight.
    - It offered a yes/no decision: "I agree that all NHS care providers may share my confidential patient information with each other and de-identify it for the purpose of research."
    - We phrased the question in this way because it is already permitted, given NHS research ethics approvals, for NHS bodies to de-identify health data for research.[7,8] However, cross-site linkage within the NHS is more challenging; if linkage is conducted with direct identifiers such as NHS numbers, this is work involving confidential patient information for research, which requires either explicit consent or approvals (in England) under section 251 of the NHS Act 2006 [4] (as amended) and the Health Service (Control of Patient Information) Regulations 2002.[9] That is the case even if the data are de-identified subsequently and researchers never see identifiable information.

For peer review only

- We said also that saying no would not prevent all uses of one's confidential information for research. At present, in England, the NHS National Data Opt-Out is the mechanism to opt out from uses of one's confidential personal information where that use is governed by section 251 of the NHS Act.[10,11]
    - Third, the specimen form asked about taking part in research.
        - It discussed briefly research involving direct participation, stating that the NHS promised to inform people of research studies for which they may be eligible,[5,6] and saying that there is never a commitment to take part.
        - It offered a yes/no decision: "I agree that NHS-approved researchers may learn my identity and contact me directly about research studies for which I may be eligible."
    - Having showed the respondent the specimen consent form, we asked Likert-style questions about its clarity, how the respondent understood its meaning, whether some additional aspects should be added, whether the respondent was previously aware of the NHS National Data Opt-Out, and whether the respondent would choose to sign such a form if it were available to them today.
- We asked, via a multiple-choice question, whether **sign-up portals for research** should be multiple (as they are now), single (across the NHS), or split by mental/physical health research.
- We asked about the respondents' preference for **linkage of their health data to other data sources** for research. We set out a basic method commonly used for identifiable linkage, in which special permissions are sought, trusted third-party linkage is conducted (using identifiable information), followed by de-identification for research. We asked whether the respondent would be happy (yes / not sure / no / prefer not to say) for their health data to be linked to a number of "state" sources (education, police/criminal justice, transport, housing, immigration, social security), giving simple examples of the potential research reasons for each linkage, plus universities (e.g. if the respondent had volunteered for research studies) and data held by private companies.
- Finally, we asked optional **demographic** questions: gender; age range; ethnicity; sexuality; religion; employment status, with conditional questions sufficient to determine the UK Office for National Statistics (ONS) National Statistics Socio-Economic Classification (NS-SEC)[12] via the "self-coded" method[13] if the respondent answered in full; UK nation of residence; and (if the respondent was willing) their postcode, to calculate a "blurred" geographical version as described below. We used ONS demographic categories where available. We offered the option to leave an e-mail address to receive a summary of results when available.
- The survey closed by thanking the participant (not shown in the Appendices).

The REDCap design for the survey is shown in **Appendix A,** and the resulting survey (including the specimen consent form) is shown in **Appendix B.**

S1.3. Analysis of geography including Index of Multiple Deprivation

Where participants agreed to leave a postcode, this was converted to a larger ONS geographical area, so that individuals could not inadvertently be identified. In turn, the larger geographical area was converted to an Index of Multiple Deprivation (IMD), a composite rank measure covering income, employment, education, health, crime, barriers to housing and services, and living environment.[14] A lower raw IMD indicates greater deprivation. For England and Wales, postcodes were converted to a Lower Layer Super Output Area; these have a minimum population of 1,000 and a mean population of 1,500.[15] For Scotland, the Data Zone was used; for Northern Ireland (NI), the Super Output Area (SOA). Here, we use the term "geographical area" (GA) for LSOA (England and Wales), DZ (Scotland), or SOA (NI). We used GAs from the 2011 Census.

GAs were converted to a UK-wide IMD score,[16,17] using the scale relative to England, in which high numbers represent greater deprivation. As this UK-wide data set uses 2001 DZs for Scotland, we mapped these to 2011 DZs.[18] Where multiple 2001 DZs mapped to one 2011 DZ, we took the mean of their UK-wide IMD scores, weighted by their population contribution to the 2011 DZ.[18]

Since GAs do not have equal populations, we corrected for population when calculating centile of deprivation. We used mid-2019 ONS estimates of GA population.[19–21] We calculated deprivation centile (100% meaning most deprived) by calculating, for each GA, "what percentage of the total UK population (66,796,806) live in a GA with a UK IMD score that is equal to or lower than that of this GA (i.e. in areas that are equally or less deprived than this GA)?". We calculated quartiles similarly.

To show the distribution of deprivation, we plotted the distribution of deprivation centile using a Gaussian kernel density estimate.

For map representations, the Apr 2019 ONS local authority district boundaries[22] (which exclude the Channel Islands) were used with the Nov 2019 ONS Postcode Directory.[23]

For peer review only - http://bmjopen.bmj.com/site/about/guidelines.xhtml

S1.4. Sensitivity analysis via survey weighting

As a sensitivity analysis to correct for unrepresentative demographic sampling during general linear modelling, we used raking,[24] specifically the American National Election Study (ANES) weighting algorithm[25] via the *anesrake* package.[26,27] We defined dimensions (classification variables) and categories (within dimensions) as shown in **Supplementary Table 1,** collapsing across some low-frequency categories in the survey. We included all such variables in the raking, and used the default weight cap of 5.

The algorithm does not alter weights for categories for which population expected proportions are not known (e.g. those identifying as neither male nor female, for which Census data are not available, or those answering "prefer not to say" for a given question). Such respondents are therefore assigned a weight of 1 in that category, representing them fairly in the absence of any other information with which to weight them.

Population values for some questions were not available; for example, sexual orientation was not part of the UK 2011 Census.[28]

We weighted all respondents once only, consistently (ignoring the potential for discrepant drop-out rates across the survey).

We used weighted versions of the statistical models (see **Methods**) that did not include demographic predictors, labelling these with "W" (e.g. model CW1 was a weighted version of model C1).

S1.5. Effect size plots

We show effect sizes with 95% confidence intervals from selected statistical models. We use the conventional language of "factor" (discrete predictor), "levels" (possible values of a factor), and "term" (individual predictive term in a GLM, such as "destination" or "destination × nature"). Effect sizes are shown as uncorrected pairwise comparisons to a reference category within each term. For age, we used the central category (age range 45–54) as the reference category, because there were very few participants in the "under 18" category. For brevity, we restrict plots to those model terms with a significant F test. We show degrees of freedom to 1 decimal place and statistics to 3 significant figures.

F tests, as for the main results, were taken from *anova(model)* via *lmerTest::anova.lmerModLmerTest*, giving type III sums of squares via the Satterthwaite method for degrees of freedom.[29] Type III sums of squares test the effect of each term "over and above" others.[30] Pairwise comparisons were taken from *summary(model)* via *lmerTest::summary.lmerModLmerTest*, also using Satterthwaite's method.

We note the important difference between these pairwise contrasts, helpful for basic visual display, and the omnibus F test for the term.[30] In particular, we note firstly that for a single factor with three levels (A, B, and C with A as the reference level), the omnibus F test tests the null hypothesis A = B = C; if this null hypothesis is rejected, it remains possible that neither the pairwise hypothesis A = B nor the hypothesis A = C is rejected (for example, if the ordering is B < A < C). Thus, failure to observe differences in the specific pairwise contrasts does not imply that there are no differences in the data. Secondly, a similar effect can be observed with interactions: pairwise contrasts within interaction components may sometimes be suboptimal compared to subgroup simple effects analyses.[30] Thus, we used simple effects analysis for detailed follow-up of significant interactions where appropriate.

S1.6. Free-text values and comments

Some respondents wrote in responses for "other" categories. Some provided free-text comments where invited to do so. A few e-mailed the study team separately. We provide narrative summaries of these answers, paraphrasing to avoid direct quotations and reporting "$n < 10$" where appropriate to mask small numbers.

For free-text comments on the proposed draft national consent form, we conducted a thematic analysis via the inductive approach[31] as follows. Comments were imported into a MySQL database table[32] whose primary key was the REDCap source record identifier. The unit of analysis (row) was a free-text comment from a single respondent. Analysis was performed in MySQL Workbench[33] by a single rater. All comments were read individually in an initial familiarization phase (phase 1).[31] Initial topics (subthemes) were drafted (phase 2) and collated into themes (phase 3).[31] Boolean columns representing each topic were created directly via Structured Query Language (SQL).[34] All comments were read sequentially and classified as matching each topic or not; one comment could be classified against multiple topics. Comments were invited in relation to the consent form specifically, but many respondents made broader comments, and we incorporated all topics, except topics unrelated to the study specifically (which we summarize in narrative form separately for completeness, but did not tally). Where new topics were identified in the coding run, these were added as columns, themes reviewed and revised, and classification performed again for that and all preceding comments (phase 4). Themes were collated into a narrative and topic comments described (phase 5). We report tallies by theme and by topic within each theme (subtheme) (phase 6).[31]

For peer review only

S2. SUPPLEMENTARY RESULTS

S2.1. Recruitment and completion

Supplementary Figure 1 shows recruitment sources, recruitment rates, and survey participation by stage.

S2.2. Demographics

A demographic breakdown is shown in **Supplementary Figure 2**.

Free-text values provided in response to "other, please specify" categories included:

- *Mental health conditions* (n = 274): many unique responses, with non-unique responses distinct from the options offered including: ADHD [attention-deficit/hyperactivity disorder], Asperger's syndrome/autism/autistic spectrum disorder, bereavement, body dysmorphic disorder, dissociative disorders, gambling disorders, insomnia, low mood, memory problems, menopause-associated symptoms, non-epileptic attack disorder, overdose, post-traumatic stress disorder, postnatal depression, suicidality, and stress.
- *Mental health services used* (n = 254): many unique responses, with non-unique responses including: counselling services, military services, specific named charities (including Mind and Samaritans), university mental health support services, and well-being services.
- *Physical health services used* (n = 258): many unique responses, with non-unique responses including: chiropractors, complementary therapists, dentistry, maternity services, NHS web sites, osteopaths, pharmacists, physiotherapists, and well-being services.
- *Gender* (n = 29): non-unique responses included: agender, gender fluid, non-binary, trans/transgender.
- *Ethnicity* (n = 150): a large number of responses more detailed than the ONS options on offer, with some critical of the premise.
- *Sexuality* (n = 65): a number of unique options and some critical of the premise or phrasing, plus some with multiple responses (e.g. asexual, panromantic, pansexual).
- *Religion* (n = 219): a range, some more closely specifying options offered (e.g. agnostic, atheist, Baptist, Catholic, Church of England, humanist, Jehovah's Witness, Methodist, Orthodox, Quaker) and some for beliefs not listed (e.g. Baha'i, druid, Jedi, pagan, pantheist, spiritualist, Wiccan), including a number of unique responses.
- *Geography* (England n = 43, Scotland n < 10, Wales n = 20, Northern Ireland n < 10): a range of geographical divisions not matching the categories offered.

S2.3. Experience

Participants' experience of mental health conditions/service and physical health services are shown in **Supplementary Figure 3**.

S2.4. Raking

The raking algorithm converged stably, and substantially improved the match to population marginal proportions, though was unable to make the resulting weights match the population marginals completely within the constraints specified. We show observed and raked proportions in **Supplementary Table 1**.

S2.5. Weighted analysis of sharing preferences

Weighted analyses (**Supplementary Figure 4**) were quantitatively similar to the primary results (compare **Figure 3**), and statistical analyses showed the same key patterns, although with somewhat weaker framing effects.

For clinical purposes (model CW1, n = 24497), there were effects including destination ($F_{3,181628}$ = 6310, VLP), nature ($F_{1,181756}$ = 4860, VLP), nature × framing ($F_{2,181756}$ = 21·3, p = 5·41 × 10^{-10}), destination × nature ($F_{3,181624}$ = 5·84, p = 0·000557), and destination × framing ($F_{6,181628}$ = 4·38, p = 0.000199). In sub-analyses of the framing effects (analysis of MH and PH data separately using model CW1B), there was no effect of framing for PH data (F < 1, NS) but this time the framing effect for MH data was not itself significant (framing, $F_{2,13607}$ = 1·74, NS; destination × framing, $F_{6,84700}$ = 1·91, p = 0.0748). As for the main analysis (model C1), the framing effects were slightly smaller for geographically broader (e.g. UK-wide) sharing, driving a destination × framing interaction via PH data.

For peer review only - http://bmjopen.bmj.com/site/about/guidelines.xhtml

For research purposes (model RW1, $n = 23869$), main effects included destination ($F_{5,547684} = 74700$, VLP) nature ($F_{1,547914} = 1613$, VLP), detail ($F_{1,548574} = 5270$, VLP), and framing ($F_{2,13472} = 3{\cdot}09$, $p = 0{\cdot}045$). Interactions included destination × nature × detail ($F_{5,547655} = 8{\cdot}64$, $p = 8{\cdot}64 \times 10^{-8}$) and nature × framing × pandemic ($F_{2,547914} = 5{\cdot}25$, $p = 0{\cdot}00536$). In sub-analyses of the framing effects (analysis of MH and PH data separately using model RW1B), framing main effects were not independently significant, though there were destination × framing interactions as before.

S2.6. Weighted views on linkage to non-health data for research

Supplementary Figure 5 shows weighted views on linkage to non-health data for research (compare **Figure 4**).

S2.7. Effect sizes

Supplementary Figure 6 shows effect sizes for models C1, R1, and L1. These models include data from all participants who answered the relevant questions, including those who did not supply full demographic information (compare **Figure 6**).

S2.8. Weighted views on a national data sharing consent system

Supplementary Figure 7 shows weighted views on a national data consent system (compare **Figure 7**).

S2.9. Free-text comments on the suggested consent form

Themes of free-text comments in relation to the proposed consent form (*n* = 3112 comments/respondents), with tallied topics within them (subthemes), included:

- Form design (*n* = 1312 respondents; note that subtheme counts within a theme can sum to more than the theme total because individual respondents sometimes commented across multiple subthemes):
  - *Too long/complex* (*n* = 732). The form was too long and/or complex, or the text (or aspects of the text) should be clearer or simpler, including statements that the reader found it OK but felt that others (not specified in detail) would not.
  - *Visual style* (*n* = 395). Regarding visual clarity and style (e.g. typography, layout, colours, and the spelling of 'organization' versus 'organisation').[35]
  - *Clear/good* (*n* = 159). The form was satisfactory, good, clear, and/or concise.
  - *Extra information or accessible versions* (*n* = 271). There should be accessible forms, easy-read versions, alternative language versions, or accompanying explanations (e.g. leaflet, illustrations, video, explanations of the benefits and risks of data sharing).
  - *Too brief* (*n* = 18). It was too brief, and/or required more detail or explanation (in general).
  - *Biased* (*n* = 16). It was biased, coercive, or misleading in its questions.
- Method of completion (*n* = 308):
  - *Digital* (*n* = 69). A digital or online version would be desirable or preferable (or in some cases that a paper copy would be desirable or necessary as well).
  - *Non-digital* (*n* = 26). A paper copy would be desirable or necessary, or that not everyone could use internet-based methods.
  - *One-to-one support* (*n* = 90). One-to-one support (e.g. face-to-face, e-mail, telephone) would be desirable or necessary (from a clinician or other unbiased person, including support for those who may lack capacity to decide, and for children).
  - *Copies* (*n* = 27). People should have copies of what they agreed to.
  - *Changing preferences* (*n* = 96). It should be easy to revisit and update one's preferences and/or contact details (and/or clarity on what would happen to data previously shared under such circumstances).
  - *Enough time* (*n* = 49). It is important people have time to complete the form and do not feel pressured to do so, and/or are not asked during times of personal medical crisis.
- Legal aspects (*n* = 58):
  - *Legal* (*n* = 58). That the Data Protection Act and/or European Union (EU) General Data Protection Regulation (GDPR), Access to Health Records Act, Information Commissioner's Office, NHS privacy documents (e.g. privacy impact assessments, Caldicott Guardian framework), or mental capacity frameworks (e.g. Mental Capacity Act, Lasting Power of Attorney) should be referenced explicitly, or

For peer review only - http://bmjopen.bmj.com/site/about/guidelines.xhtml

views that the proposed system/form would be (or that existing NHS information systems are) incompatible with one of these.

- Managing data related to consent (*n* = 83):
  - *Data for the consent process* (*n* = 83). Relating to self-identification when completing the form: that asking for an NHS number is problematic as people may not know this, that some identifiers (e.g. full name, address, e-mail address) are unnecessary or sometimes unnecessary, or that some (e.g. telephone number, previous names and addresses) should be added, or concerns about the management of identity information used for the consent form.
- Management of healthcare data (*n* = 1079):
  - *De-identification* (*n* = 164). That aspects of de-identification/anonymisation/pseudonymisation needed to be clearer, including that asking for identity data whilst recording preferences about de-identified data use was confusing, or that any form of pseudonymisation (rather than full anonymisation) is undesirable, or that examples should be given of identifiable versus de-identified data.
  - *Data security* (*n* = 594). That the security and privacy of healthcare data is of paramount importance, including that more detail should be provided about data security/sharing or privacy controls, or that the respondent did not trust the NHS or the UK to manage data security properly.
  - *Centralization* (*n* = 30). Greater centralisation of data increases concern and risks.
  - *Sharing desirable* (*n* = 290). Data sharing is desirable in general, for clinical or research purposes or not specified.
  - *Sharing undesirable* (*n* = 74). Data sharing is undesirable in general, e.g. without case-by-case specific consent (± except in emergencies).
  - *Opt-outs* (*n* = 134). That opt-outs should be more prominent, the default, that the system should be linked with the NHS National Data Opt-Out (directly or by implication), or that the relationship to NHS Act Section 251 approval was unclear.
- Categories of healthcare data (*n* = 77):
  - *Distinguish mental/physical health* (*n* = 45). Mental health and physical health data should be distinguished.
  - *Combine mental/physical health* (*n* < 10). Mental health and physical health data should not be distinguished.
  - *Distinguish health data in other ways* (*n* = 11). Further subtypes of health data (e.g. sexual health, drug/alcohol use) should be distinguished.
  - *Distinguish free text from structured data* (*n* = 23). Free/narrative text should be distinguished from structured data.
  - *Distinguish data by age* (*n* < 10). Data should be distinguished by its age (historical versus recent).
- Categories of use (*n* = 264):
  - *Clinical versus research* (*n* = 44). Clinical and research uses of data should be more clearly distinguished (e.g. not included on the same form).
  - *Transparency of use* (*n* = 34). That people should be informed about, or be able to inspect, or choose regarding, each individual use of their data, and/or the results of research involving their data.
  - *Profit-making* (*n* = 198). That profit-making use should not happen, or people should be paid directly for providing data used for profit, or that the NHS should ensure that it profits from such data use.
- Data or use/users, not distinguished (*n* = 74):
  - *More detail* (*n* = 74). More detailed options should be available (in general).
- End users (*n* = 674):
  - *Specify clinical users more* (*n* = 147). Permissions to share clinical data should not relate to the whole NHS, but be more detailed (e.g. local, regional, or regarding specific staff groups, or regarding private healthcare providers, or the relationship to social rather than health care).
  - *Consent not needed* (*n* < 10). That consent (or at least explicit consent) is not required for aspects of data sharing, or should not be required.
  - *Specify researchers more* (*n* = 268). That "NHS-approved researchers" is too vague, and that more detail about who such researchers are, or more detailed options to select from (up to and including case-by-case approval by the patient concerned), should be provided.

For peer review only - http://bmjopen.bmj.com/site/about/guidelines.xhtml

- *Third-party/private users* (*n* = 417). That healthcare data should not be available to private or third-party companies (or should require specific permission), including insurance companies and other profit-making organizations, or that later developments to provide them with data would be of concern.
- *Other state users* (*n* = 29). That use by other state/Government organizations would be of concern, or more detail would be required in that regard.
- *Overseas users* (*n* = 52). That use by overseas users (e.g. researchers) would be of concern, e.g. because other countries have different data regulations.

• Contact by researchers (*n* = 69):

- *Contact by researchers* (*n* = 69). That more detailed options should be available about consent for research, or that being contact directly would be of concern (including about frequency of contact, and preferences that contact is always via clinical teams rather than researchers being given identifiable information).

• Overall objectives (*n* = 144):

- *Good idea* (*n* = 128). The consent system or project was a good idea (in general).
- *Bad idea* (*n* = 16). The consent system or project was a bad idea, or unnecessary.

• Other (*n* = 242):

- We did not tally comments relating to other topics, such as the accuracy of health records themselves, patient access to health records (or holding the primary version of all their health data themselves), descriptions of an individual's care, details of how the respondent would themselves choose, comments on the relationship to consent for clinical treatment, expressed wishes that the research team be shot, comments that people never read forms anyway, views that an independent body should oversee such a process, or concerns about research bias relating to who would or would not consent to their data being used.

S2.10. Other free-text comments

Themes of the free-text comments sent to the research team separately included:

- That the respondent's preferences regarding mental versus physical health data had been driven by personal experience of information being misused (*n* < 10).
- That all research use of data should require informed consent, involving participants' knowledge of the nature of the research (*n* < 10).

For peer review only

S3. SUPPLEMENTARY DISCUSSION

S3.1. Under-represented groups

All surveys carry the risk of sample bias, even if participation is a legal obligation.[36,37] The use of an online survey biases against or precludes some groups (e.g. those without Internet access; those without appropriate assistive technology; those for whom our REDCap site was insufficiently accessible; those with reading difficulties; those not fluent in English). We had envisaged that recruitment via a large network of health professionals (such as those forming the NIHR CRN) would help to overcome these problems. This would have included NHS research support staff providing assistance in person, if requested, and the provision of NHS devices to complete the survey. Our ethics approvals and protocol covered this process, and such support may indeed have taken place very early in the study, before COVID-19. Use of face-to-face recruitment by healthcare teams was also expected to enrich the sample for current users of healthcare services, and thus indirectly for groups at higher risk of health conditions. Face-to-face recruitment was the favoured approach of several participating healthcare sites. We had also planned to hold events in selected towns across the UK, and to reach out to local community groups directly and via the participating/recruiting sites, to try to ensure representative coverage (including of minority groups).

However, soon after the study opened, face-to-face recruitment and engagement became impossible (prohibited) due to COVID-19. Thereafter, we had to rely on online methods of recruitment only. Even after a degree of national "unlocking", extra care was advised for groups vulnerable to COVID-19, to minimize unecessary face-to-face contact, and some restrictions remained. Direct healthcare was prioritized throughout and in-person NIHR/NHS support for research was restricted, primarily to studies relating directly to COVID-19 (e.g. studies of vaccinations and treatments). This enforced shift to online recruitment and lack of anticipated extra support will have contributed to some of the observed bias (**Supplementary Table 1**).

During this period, we observed from anonymous data that some groups (e.g. males, young people, those of minority ethnicity) were under-represented amongst respondents who reported demographic information. One of our approved online recruitment methods was via social media, and some social media sites permit targeted as well as untargeted advertising, but only for some demographic characteristics; observing this bias, we prioritized young people and males for some of our study advertising. Rather than relying only on static recruitment materials, we made an online video to try to boost recruitment from groups predisposed to this format. We did not measure the effects across time, but overall these measures were inadequate to address the bias. In the analytical phase, however, we confirmed that weighted (raked) analyses were not materially discrepant from unweighted analyses (**Supplementary Results**).

The problem of some groups being under-represented remains, internationally.[38,39] We are currently working with UK organizations and individuals representing minority groups and with patient/public involvement and engagement networks across the UK, including via the Health Data Research UK (HDR UK) mental health data research hub, DATAMIND[40] (MRC grant reference MR/W014386/1), to reach out to those whose views are insufficiently heard.

S3.2. UK legal basis for using NHS data for clinical or research purposes

We are not legal experts and this is not, and should not be taken as, legal advice. This topic is also undoubtedly complex. However, our understanding of the legal basis for the holding and use of NHS data in the UK is as follows, summarized in **Supplementary Figure 8**. We focus on legislation and guidance applicable to England, where we work, and where we discuss research, we focus on research by public authorities such as NHS organizations, universities, and research council institutes.[41]

Main UK data protection laws. The European Union (EU) General Data Protection Regulation (GDPR)[8] governs data protection within the EU. The UK implemented this in domestic law via the Data Protection Act 2018 (DPA).[7] When the UK left the EU in 2020, the GDPR remained in UK law as the "frozen GDPR" or "UK GDPR", by virtue of the European Union (Withdrawal Agreement) Act 2020 §3,[42] which enabled some legislation to be amended by ministerial direction following the UK's withdrawal from the EU, and the Data Protection, Privacy and Electronic Communications (Amendments etc[.]) (EU Exit) Regulations,[43] a statutory instrument (ministerial order) that amended relevant UK data protection legislation. We refer simply to the "GDPR" below. Data protection in the UK is overseen by the Information Commissioner's Office (ICO), the UK's independent authority for data protection.

The UK also has specific legislation that governs NHS data, including the NHS Act 2006[4] and the Health Service (Control of Patient Information) Regulations (CPIR).[9] The NHS Act has since been amended, including by the Health and Social Care Act 2012 (HSCA)[44] and the DPA, and is often referred to as the "NHS Act 2006 (as amended)" (NHSA) as a result. Additionally, there is a duty of confidentiality applicable to health care arising from common law (which is to say, case law rather than statute).[45] A duty to share information for health care, discussed below, was created by the Health and Social Care (Safety and Quality) Act 2015 (HSCSQA).[46]

For peer review only - http://bmjopen.bmj.com/site/about/guidelines.xhtml

Governance within NHS organizations. In addition to statutory and common-law obligations relating to privacy and data protection, NHS staff delivering health care operate under professional duties of confidentiality, overseen by regulators such as the UK General Medical Council (GMC) and the Nursing and Midwifery Council (NMC). They set out confidentiality requirements.[45,47] NHS organizations must appoint a Data Protection Officer (GDPR Article 37, page 55) and also a Caldicott Guardian, a senior professional responsible for safeguarding the confidentiality of patient information.[48] Many NHS Trusts are active in medical research, which has additional governance requirements, as outlined below.

Nature of the identifiable data. Data relating to an identifiable living person is "personal data" under GDPR Article 4(1) (p. 33), and DPA §3 (p. 2). Both the GDPR (Recital 27, p. 5) and the DPA (§3) exclude personal data relating to deceased persons, but the NHS continues to treat information about deceased people with respect for confidentiality, and a common law duty of confidentiality (or confidence) continues to apply to deceased people.[49] Health data is "sensitive personal data" or "special category" data or a topic of "sensitive processing" [GDPR Recital 10 (p. 2) and Article 9 (p. 38); DPA §35(8) (p. 22) and §86 (p. 51)]. The NHSA[4] refers to medical data, which may be de-identified, as "patient information" [NHSA §251(10), p. 266] and uses the term "confidential patient information" for identifiable medical data [NHSA §251(11), p. 266], making explicit reference also to the obligations of confidence. This obligation of confidence is expanded on further[49] by the ICO.

Clinical purposes: identifiable data. Though it is perhaps surprising at first, consent is not the main legal basis through which the NHS holds sensitive personal data about patients. Of course, patients often give consent, explicitly or implicitly. However, health care also involves patients who lack capacity to consent[50,51] or who explicitly refuse, whether with capacity or not.[52–54] There are other valid reasons for data processing without consent.[45] Accordingly, the legal basis for NHS organizations to hold identifiable patient data is not, or should not be, consent.[55,56] Instead, it is usually GDPR Article 6(1)(c) (p. 36), processing of personal data to comply with a legal obligation on the data controller; GDPR Article 6(1)(e), processing to perform a task in the public interest or in the exercise of official authority vested in the controller; GDPR Article 6(3), setting out that the meaning of 6(1)(c) and 6(1)(e) can be defined by EU or member state law; GDPR Article 9(2)(h) (p. 38), processing of special categories of personal data for the purposes of the provision of health care; GDPR Article 9(3), that use of Article 9(2)(h) requires obligations of professional secrecy; DPA §10(2) (p. 6), that processing of special categories of personal data fulfils the conditions of GDPR Article 9(2)(h) if it meets a condition in Part 1 of Schedule 1 of the DPA; and DPA Schedule 1 (Part 1) ¶2 (p. 131), health or social care purposes. Sometimes GDPR Article 9(2)(i), processing for reasons relating to public health, also applies.[55]

Clinical service evaluation and audit. NHS organizations routinely evaluate the performance of their own services, and may use identifiable or de-identified data to do so. If possible, the latter is clearly preferable from an information governance standpoint. There is a distinction between service evaluation, which NHS organizations conduct independently, and research (discussed next), which requires independent research ethics approvals and governance. The distinction between research and service evaluation may be less sharp for work involving only the secondary use of routinely collected data. The NHS Health Research Authority (HRA) provides classification guidance.[57] Legal support for service evaluation may include GDPR Article 6(1)(c), legal obligation; Article 6(1)(e), task in the public interest; Article 9(1)(h), which includes the "management of health or social care systems and services" as well as direct health care; and DPA sections as in the preceding paragraph.

Clinical purposes: data processors. While data controllers "own" data and are responsible for deciding how it is processed [e.g. GDPR Article 4(7)], data processors handle data on behalf of the controller and do what the controller instructs [e.g. GDPR Articles 4(8) and 29]. The contract between the two (e.g. GDPR Articles 28–30, pp. 49–51) is formalized via a data processing agreement. An NHS organization (data controller) will often engage commercial organizations as data processors to manage identifiable and sensitive patient data, such as when a software company provides an electronic health record system to the NHS; this situation is very widespread.[58]

Clinical purposes: sharing data between NHS organizations. The Caldicott principles include the duty to share relevant information for clinical care; the 2013 Caldicott review ("Caldicott 2") noted that this can be as important as the duty to protect patient confidentiality.[59] The HSCSQA 2015 created a legal duty for health or social care providers (or commissioners) to disclose information about an individual to another provider/commissioner where that disclosure would likely facilitate the provision of health or social care in England, is in the individual's best interests, and the individual does not object (or would be likely to object) to the disclosure, so long as there is not a conflict with data protection legislation or a common law duty of care or confidence (HSCSQA §3, p. 3, creating HSCA §251B).

The DPA has provision for joint data controllers (§58, p. 34), as does the GDPR [Article 4(7), p. 33]. Data sharing agreements (DSAs) formalize and support appropriate sharing.[59] NHSA §75 (pp. 113–115) makes provision for joint work between NHS bodies and Local Authorities for the provision of health-related functions. "Caldicott 2"[59] noted that NHSA §251 support (discussed further below) is not required for disclosure of personal confidential data by one health service body to another (p. 110), and that the NHSA §251 excludes processing "solely or principally for the purpose of determining the care and treatment to be given to particular individuals" [NHSA §251(6)]. Many parts of the NHS have, or are developing, "shared care record" systems (ShCRs) for direct patient care, in which identifiable information is shared for clinical purposes between different NHS organizations (such as primary care, secondary care, and ambulance

For peer review only - http://bmjopen.bmj.com/site/about/guidelines.xhtml

services), including without explicit consent. NHSX advises that this should be done using the joint data controller provisions of the DPA, alongside DSAs, data protection impact assessments (DPIAs, per GDPR Recital 84), and other agreements ([60], pp. 12–16). It notes that opt-outs are a matter of local policy, separate from any right to object under GDPR Article 21 [to data being processed under Article 6(1)(e) or 6(1)(f)] ([60], p. 31). It emphasizes that access to information should only be permitted in a proportionate way, to relevant data, and only if there is a legitimate relationship between the health professional concerned and the patient (p. 37).

Research purposes: identifiable data, with consent. Research involving NHS patients in the UK must be approved by the HRA and/or by one of its NHS Research Ethics Committees (RECs), in addition to approval by individual participating NHS organizations. Informed consent is a principle of participatory research,[61] and consent might be the basis of data management—such as GDPR Article 6(1)(a), consent; Article 9(2)(a), consent for special-category data; DPA §2(1)(a) (p. 2), consent as a principle; and DPA §84(2) (p. 50), defining consent. However, there are also additional provisions and exceptions in the GDPR and DPA relating to research, and legal reasons other than consent to hold data, such as GDPR Article 6(1)(e), performance of a task in the public interest (as above). The GDPR appears to refer to the possibility of multiple reasons in Articles 6(1) and perhaps 9(2), but the correct policy is likely to be "pick one".[62] So, consent might be the basis on which a subject joins a research study, but not the lawful basis for processing the data that result, which might instead be "task in the public interest" (see below); the lawful basis should be made explicit to the subject.[41] The ICO summarizes these research exceptions;[63] for example, personal data can be kept indefinitely for research purposes and the subject's right to object later may be limited if the data are being processed for research.

Research purposes: identifiable data, without consent. Under some circumstances, identifiable NHS data is used for research without consent. This requires a strong public interest justification, and approval under NHSA §251 (pp. 265–266; "section 251 approval"). NHSA §251 allows regulations to be made for the use of confidential patient information for medical purposes, which includes medical research [NHSA §251(12)]. These specific provisions of the NHS Act §251 are implemented in the CPIR's Regulation 5 and Schedule, which require that such medical research be approved both by the Secretary of State and by a REC. For the former, approval is sought via the UK Confidentiality Advisory Group (CAG). The CAG is part of the HRA, and replaced the National Information Governance Board for Health and Social Care referred to in the NHS Act 2006. For this purpose it advises (and in practice acts on behalf of) the Secretary of State for Health and Social Care. A well-known example of research conducted under §251 approval is the National Confidential Enquiry into Suicide and Safety in Mental Health (e.g. [64]), but there are many others; some data linkage work may also require the use of identifiers initially, even if these are then removed before researchers are granted access to the data.[65] Research without consent is provided for by GDPR Article 6(1)(e), tasks in the public interest; GDPR Article 9(2)(j), processing of sensitive personal data for research or statistical purposes; GDPR Article 89 (p. 84), relating to safeguards and derogations in member state law for the purposes of research; DPA §10(2), compatibility with those GDPR provisions if a condition in DPA Schedule 1, Part 1 is met; DPA Schedule 1 (Part 1) ¶4 (p. 132), research purposes; and DPA §19(4) (p. 12), regarding processing for research and defining "approved medical research" to include research approved by a REC recognized by the HRA. The NHSA [§251(4)], CPIR [Regulation 7], and the CAG[66,67] are clear that §251 is a last resort: if work can be conducted other than by using confidential patient information (without consent), those other methods should be used.

NHS National Data Opt-Out, relating to identifiable data without consent. The NHS National Data Opt-Out (NDO), created on the advice of the National Data Guardian (Caldicott) 2016 review,[68] applies to §251 work.[10,11,69,70] Specifically, it applies to personal confidential data (identifiable information), being used for "purposes beyond individual care", where consent has not been explicitly given, and NHS Act §251 support is used as the legal basis. It does not apply to anonymised data,[69] defined with reference to the ICO Code of Practice on anonymisation.[71] It is not an absolute opt-out with respect to identifiable data. For example, it does not apply to information flowing into NHS Digital (the operating name of the Health and Social Care Information Centre), unless those data flows rely on §251 support; nor to some other specific programmes including national cancer screening programmes; nor to data flows for the production of some disease registers, for some public health purposes, or for official statistics; nor when information is required to be disclosed by law.[68,69] Likewise, it does not apply when data controllers process information internally (within their "data controllership boundary") unless the purpose of use changes and §251 support is relied upon.[69] It continues to apply after death.[69] The phrasing of the choice offered to NHS patients, after some explanation is given, is: "I allow my confidential patient information to be used for research and planning".[69]

De-identification. We discuss forms of de-identified data next. There is a grey zone around de-identification (pseudonymisation and anonymisation), relating to what is obviously identifiable, what is identifiable if the reader has extra information, and whether the techniques for re-identification are simple or sophisticated. If all identifiers are replaced by a pseudonym (code), at face value the data may not be identifiable by a person who does not possess the mapping between pseudonym and identity. Of course, the data will be identifiable with little effort by a person or organization who does hold the mapping between pseudonym codes and identities. However, details in de-identified data may support re-identification by a malicious actor, e.g. via a so-called "jigsaw" attack.[72] We gave a simple example in our survey (**Appendix B**) in which de-identified free text might be susceptible to re-identification via a newspaper report about the same incident.

The 2012 ICO code of practice on anonymisation notes that "*[t]here is clear legal authority for the view that where an organisation converts personal data into an anonymised form and discloses it, this will not amount to a disclosure of*

For peer review only - http://bmjopen.bmj.com/site/about/guidelines.xhtml

personal data. This is the case even though the organisation disclosing the data still holds the other data that would allow re-identification to take place. This means that the DPA no longer applies to the disclosed data..." (p. 13). The code cites relevant case law.[73] However, it also stresses that "*the risk of re-identification through data linkage is essentially unpredictable because it can never be assessed with certainty what data is already available or what data may be released in the future*" (p. 18). It stresses, therefore, the importance of risk assessment regarding re-identification, and of the use of the minimum quantity of data necessary. It notes that "*[n]either the DPA nor the FOIA* [Freedom of Information Act[74]] *provide any practical assistance in terms of helping organisations to determine whether… the anonymised data they release is likely to result in the re-identification of an individual...*", but suggests the "motivated intruder" test—whether re-identification would be possible by someone starting without prior knowledge but with technical and investigative skill, who is motivated to discover the identity of someone in a data set.

What gives authority to de-identify data? Although the CPIR (Schedule, p.6) makes reference to de-identification of confidential patient information as one activity that may be conducted under CPIR Regulation 5 (relating to research under NHSA §251), it is also explicit that these regulations relate to the transfer of information between bodies, or disclosure [NHSA §251(2)(a, b); CPIR Regulation 6], covering situations such as transfer to another organization followed by de-identification. Our understanding is that if an organization (data controller) holds data legitimately, it may normally de-identify that data itself; this is made clear by the ICO code of practice on anonymisation[71] (p. 13, as above, and pp. 28–29, that consent is generally not required to anonymise data); but also by the CAG (e.g. that de-identification is an alternative to §251 approval and a preferable one);[66] the GDPR (Recitals 28–29, p. 5, e.g. that "to create incentives to apply pseudonymisation... measures of pseudonymisation should... be possible within the same controller"); and the DPA (§171, p. 99, regarding the offences of re-identifying de-identified data and the responsibilities of the controller, in addition to the provisions listed above/below regarding the processing of the data in the first place).

The ICO is consulting on new guidance in this area.[75] In its drafts, it is explicit that the same information can be personal data to one organisation but anonymous in the hands of another, so context is vital; that the act of anonymisation (or pseudonymisation) counts as "processing" so must have a valid legal basis, but that it is normally fair and lawful for this to be done by an organization holding personal data (see below); and that DPA references to "de-identified" information are to pseudonymised information, and pseudonymised information remains personal data.[76] It provides a flowchart for categorizing data legally along a spectrum of identifiability, from fully identifiable to truly anonymous.[77] It notes that scientific/historical research and statistical purposes are both purposes "compatible" with further processing [relating to GDPR Recital 50 and Article 5(1)(b)].[78]

Research purposes: pseudonymised data, with consent. Research involving pseudonymised data with explicit patient consent is likely to follow the same rules as for research using identifiable data with consent, as above.

Research purposes: pseudonymised data, without consent. In the GDPR (Recital 26, p. 5), pseudonymised data is considered personal data if it "could be attributed to a natural person by the use of additional information" (e.g. if the mapping is available). The DPA uses the term "de-identified" data to mean data that cannot be attributed to a specific person "without more" [DPA §171(2)(a), p. 99], which may be compatible with the interpretation that pseudonymised data can be identifiable if one holds the mapping keys and de-identified if not; however, the ICO provides conservative clarification (noting also that the risk of re-identification must be considered in the round), and the ICO is currently consulting on further guidance around anonymisation,[79] as above. Under the DPA, it is a criminal offence to re-identify de-identified data without the permission of the relevant data controller (DPA §171–172, pp. 98–100).

Research using de-identified (pseudonymised) data without consent is supported by GDPR Articles 6(1)(e), 9(2)(j), and 89, and DPA §10(2), Schedule 1 ¶4, and §19(4), as above (the research clauses). The GDPR also supports the principle of pseudonymisation (Recitals 28–29).

Research purposes: patient-level anonymised data. If the pseudonyms are removed, and a person cannot be identified from the data, then they do not represent "personal data". The GDPR is explicit that data protection principles do not apply to anonymous information, i.e. information from which a person is not identifiable (Recital 26). However, the same caveats about malicious re-identification through jigsaw attack apply, and the same criminal offences under the DPA regarding re-identification. In general, the richer the data, the easier it is to re-identify, and the more sensitive its content is, the greater the level of concern. The ICO say that if an individual can be re-identified by "any reasonably available means", then it is merely pseudonymised, not anonymised.[79] However, the amount of information required to perform a jigsaw attack can be extremely small;[72] we consider patient-level NHS data, even anonymised, to require significant safeguards.

Research purposes: aggregated anonymous health data. This does not fall under the scope of the DPA or GDPR; it is not "personal data" and may be published freely. However, the level of aggregation is also important: very small group numbers may also support jigsaw attack, so appropriate "statistical disclosure control" is vital as part of the aggregation process.[80]

Research purposes: opt-outs relating to de-identified data. The NDO does not apply to de-identified data, and fully anonymised data is not "personal data" and therefore not within the scope of the GDPR or DPA, as above. While consent for use is not legally required for the use of anonymised information, and opt-outs may not be legally required,

For peer review only - http://bmjopen.bmj.com/site/about/guidelines.xhtml

it is often considered good practice nevertheless to offer opt-outs with respect to the processing of de-identified data for research (mindful that this may create bias), and many NHS organizations do so (e.g. [81,82]).

Changes in relation to the COVID-19 pandemic. The CPIR rules were varied during the pandemic to require organizations providing health services to process confidential patient information for purposes relating to COVID-19, including direct patient care and research.[83] Research conducted on this basis included national linkage studies to examine risk factors for COVID-19 mortality.[84]

For peer review only

S4. SUPPLEMENTARY TABLES

Supplementary Table 1. Demographic dimensions and categories used for weighting, with UK population marginal proportions (*e*), observed marginal proportions amongst respondents (*o*, ignoring those who did not answer or answered via a category not acknowledged by UK population statistics), and marginal proportions after raking (*r*, likewise). Proportions are shown to 3 decimal places; VLP, $p<2{\cdot}2\times10^{-16}$.

**Variable (dimension)**	**Category**	**UK expected population proportion, *e***	**Reference for *e***	**Observed proportion (ignoring other/unknown), *o***	**Sample versus population proportions**	**Raked proportion (ignoring other/unknown), *r***
Age	Under 18	0·211	[85,86]	0·005	χ^2_8=8860, VLP	0·024
	18–24	0·085		0·050		0·082
	25–34	0·135		0·139		0·167
	35–44	0·126		0·163		0·159
	45–54	0·136		0·218		0·177
	55–64	0·122		0·228		0·160
	65–74	0·100		0·150		0·133
	75–84	0·060		0·043		0·078
	85+	0·025		0·004		0·019
Gender	Female	0·506	[85]	0·673	χ^2_1=2390, VLP	0·471
	Male	0·494		0·327		0·529
Ethnicity	White	0·860	[87] (data only available for England and Wales)	0·910	χ^2_1=442, VLP	0·861
	Other	0·140		0·090		0·139
Education	No formal	0·232	[88]; data for over-16s only; vocational data absent for Scotland (ignored in calculating proportions)	0·025	χ^2_4=13800, VLP	0·121
	Level 1 and Level 2 (approximating "secondary")	0·292		0·139		0·250
	Level 3 (approximating "A-Level/equivalent")	0·121		0·122		0·146
	Level 4+ (approximating undergraduate, postgraduate, and professional)	0·270		0·589		0·364
	Vocational	0·084		0·126		0·119
Religion	No religion or not stated	0·332	[89] (Table B; data only available for England and Wales)	0·618	χ^2_2=10900, VLP	0·337
	Christian	0·565		0·338		0·563
	Other	0·102		0·043		0·100
Nation	England	0·843	[85] (Channel Islands not included)	0·994	χ^2_3=3660, VLP	0·968
	Northern Ireland	0·028		0·0002		0·001
	Scotland	0·082		0·002		0·010
	Wales	0·047		0·004		0·020
Index of multiple deprivation	First (least deprived) quartile	0·25	By definition	0·334	χ^2_3=1620, VLP	0·247
	Second	0·25		0·317		0·195
	Third	0·25		0·233		0·279
	Fourth (most deprived)	0·25		0·116		0·279

For peer review only

S5. SUPPLEMENTARY REFERENCES

1 Aitken M, de St Jorre J, Pagliari C, Jepson R, Cunningham-Burley S. Public responses to the sharing and linkage of health data for research purposes: a systematic review and thematic synthesis of qualitative studies. *BMC Med Ethics* 2016; **17**: 73.

2 Grippo AJ, Johnson AK. Biological mechanisms in the relationship between depression and heart disease. *Neurosci Biobehav Rev* 2002; **26**: 941–62.

3 Royal College of Psychiatrists. CR183: Liaison psychiatry for every acute hospital. Integrated mental and physical healthcare. 2013. https://www.rcpsych.ac.uk/docs/default-source/members/faculties/liaison-psychiatry/cr183liaisonpsych-every-acute-hospital.pdf?sfvrsn=26c57d4_2.

4 UK. National Health Service Act 2006. 2006 https://www.legislation.gov.uk/ukpga/2006/41.

5 NHS England. The NHS Constitution for England. 2013; published online March 26. https://www.gov.uk/government/publications/the-nhs-constitution-for-england.

6 NHS England. The NHS Constitution for England. 2015; published online July 27. https://www.gov.uk/government/publications/the-nhs-constitution-for-england.

7 UK. Data Protection Act 2018. 2018 http://www.legislation.gov.uk/ukpga/2018/12/contents/enacted.

8 European Parliament and Council. Regulation (EU) 2016/679 (General Data Protection Regulation). *Off J Eur Union* 2016; **L119**: 1–88.

9 UK. The Health Service (Control of Patient Information) Regulations 2002. 2002 http://www.legislation.gov.uk/uksi/2002/1438/contents/made.

10 NHS. National Data Opt-out: Factsheet 2 – When it applies. 2018. https://digital.nhs.uk/binaries/content/assets/website-assets/services/national-data-opt-out-programme/guidance-for-health-and-care-staff/nhs-factsheet-2-23052018.pdf (accessed July 26, 2019).

11 NHS Digital. Understanding the national data opt-out. 2019. https://web.archive.org/web/20200804083843/https://digital.nhs.uk/services/national-data-opt-out/understanding-the-national-data-opt-out.

12 Office for National Statistics. Standard Occupational Classification 2010: Volume 3: The National Statistics Socio-economic Classification: (Rebased on the SOC2010) User Manual. 2010. https://www.ons.gov.uk/file?uri=/methodology/classificationsandstandards/standardoccupationalclassificationsoc/soc2010/soc2010volume3thenationalstatisticssocioeconomicclassificationnssecrebasedonsoc2010/soc2010vol31amendedjanuary2013tcm77179133.pdf (accessed Feb 13, 2020).

13 Office for National Statistics. Deriving the NS-SEC: self-coded method. 2012. https://www.ons.gov.uk/methodology/classificationsandstandards/standardoccupationalclassificationsoc/soc2010/soc2010volume3thenationalstatisticssocioeconomicclassificationnssecrebasedonsoc2010#deriving-the-ns-sec-self-coded-method (accessed Aug 23, 2019).

14 UK Ministry of Housing, Communities & Local Government. English indices of deprivation 2015. 2015. https://www.gov.uk/government/statistics/english-indices-of-deprivation-2015 (accessed May 15, 2018).

15 UK Office for National Statistics. Census geography: An overview of the various geographies used in the production of statistics collected via the UK census. 2011. https://www.ons.gov.uk/methodology/geography/ukgeographies/censusgeography (accessed May 15, 2018).

16 Abel GA, Barclay ME, Payne RA. Adjusted indices of multiple deprivation to enable comparisons within and between constituent countries of the UK including an illustration using mortality rates. *BMJ Open* 2016; **6**: e012750.

For peer review only

17 Abel GA, Payne RA, Barclay ME. UK Deprivation Indices. 2016; published online Nov 21. https://dx.doi.org/10.5523/bris.1ef3q32gybk001v77c1ifmty7x (accessed Feb 23, 2020).

18 Scottish Government. Data Zone Matching File (Area and Pop. based 2001 - 2011). 2015. https://www2.gov.scot/Resource/0048/00483037.xlsx.

19 Office for National Statistics. Lower layer Super Output Area population estimates (supporting information). 2019. https://www.ons.gov.uk/peoplepopulationandcommunity/populationandmigration/populationestimates/datasets/lowersuperoutputareamidyearpopulationestimates.

20 National Records of Scotland. Mid-2019 Small Area Population Estimates for 2011 Data Zones. 2020. https://www.nrscotland.gov.uk/statistics-and-data/statistics/statistics-by-theme/population/population-estimates/2011-based-special-area-population-estimates/small-area-population-estimates/mid-2019.

21 Northern Ireland Statistics and Research Agency. 2019 Mid Year Population Estimates for Northern Ireland. 2020. https://www.nisra.gov.uk/publications/2019-mid-year-population-estimates-northern-ireland.

22 Office for National Statistics. Local Authority Districts (April 2019) UK BFE. 2019. https://geoportal.statistics.gov.uk/datasets/b06ce126e1014409ac24c82739b2c130_0.

23 Office for National Statistics. ONS Postcode Directory (November 2019). 2019. https://geoportal.statistics.gov.uk/datasets/ons-postcode-directory-november-2019.

24 Kalton G, Flores-Cervantes I. Weighting methods. *J Off Stat* 2003; **19**: 81–97.

25 DeBell M, Krosnick JA. Computing Weights for American National Election Study Survey Data. Ann Arbor, MI; Palo Alto, CA: American National Election Studies, 2009 https://electionstudies.org/wp-content/uploads/2018/04/nes012427.pdf.

26 Pasek J. ANES weighting algorithm: a description. 2010; published online March 15. https://web.stanford.edu/group/iriss/cgi-bin/anesrake/resources/RakingDescription.pdf.

27 Pasek J. anesrake: ANES Raking Implementation. 2018. https://CRAN.R-project.org/package=anesrake (accessed June 4, 2021).

28 Office for National Statistics. 2011 Census Household Questionnaire. 2011. https://www.ons.gov.uk/file?uri=/census/censustransformationprogramme/consultations/the2021censusinitialviewoncontentforenglandandwales/2011censusquestionnaireenglandh1.pdf.

29 Satterthwaite FE. An approximate distribution of estimates of variance components. *Biometrics* 1946; **2**: 110–4.

30 Cardinal RN, Aitken MRF. ANOVA for the Behavioural Sciences Researcher. Mahwah, N.J: L. Erlbaum, 2006.

31 Braun V, Clarke V. Using thematic analysis in psychology. *Qual Res Psychol* 2006; **3**: 77–101.

32 MySQL AB. MySQL. 1995 http://www.mysql.com/.

33 Oracle Corporation. MySQL Workbench 8.0.20. 2020. https://www.mysql.com/products/workbench/ (accessed April 27, 2020).

34 Codd EF. A Relational Model of Data for Large Shared Data Banks. *Commun ACM* 1970; **13**: 377–87.

35 Oxford University Press. New Shorter Oxford English Dictionary. Oxford, UK: Oxford University Press, 1997.

36 UK. Census Act 1920. 1920 https://www.legislation.gov.uk/ukpga/Geo5/10-11/41/contents (accessed Feb 3, 2022).

37 Plewis I, Simpson L, Williamson P. Census 2011: Independent Review of Coverage Assessment, Adjustment and Quality Assurance. 2011. https://webarchive.nationalarchives.gov.uk/ukgwa/20160108085307mp_/http://www.ons.gov.uk/ons/guide-method/census/2011/the-2011-census/the-2011-census-project/independent-

For peer review only

assessments/independent-review-of-coverage-assessment--adjustment-and-quality-assurance/independent-review-final-report.pdf (accessed Feb 3, 2022).

38 Pierce M, McManus S, Jessop C, *et al.* Says who? The significance of sampling in mental health surveys during COVID-19. *Lancet Psychiatry* 2020; **7**: 567–8.

39 Yu Z, Kowalkowski J, Roll AE, Lor M. Engaging Underrepresented Communities in Health Research: Lessons Learned. *West J Nurs Res* 2021; **43**: 915–23.

40 HDR UK. DATAMIND - our Hub for Mental Health Informatics Research Development. 2021. https://www.hdruk.ac.uk/helping-with-health-data/health-data-research-hubs/datamind/ (accessed March 2, 2022).

41 NHS Health Research Authority. Legal basis for processing data. 2018; published online May 8. https://www.hra.nhs.uk/planning-and-improving-research/policies-standards-legislation/data-protection-and-information-governance/gdpr-detailed-guidance/legal-basis-processing-data/ (accessed Feb 7, 2022).

42 UK. European Union (Withdrawal Agreement) Act 2020. 2020 https://www.legislation.gov.uk/ukpga/2020/1/contents/enacted.

43 UK. The Data Protection, Privacy and Electronic Communications (Amendments etc) (EU Exit) Regulations 2019. 2019 https://www.legislation.gov.uk/ukdsi/2019/9780111177594/contents.

44 UK. Health and Social Care Act 2012. 2012 http://www.legislation.gov.uk/ukpga/2012/7/contents.

45 UK General Medical Council. Confidentiality: good practice in handling patient information. 2018 https://www.gmc-uk.org/ethical-guidance/ethical-guidance-for-doctors/confidentiality.

46 UK. Health and Social Care (Safety and Quality) Act 2015. 2015 http://www.legislation.gov.uk/ukpga/2015/28/contents.

47 Nursing and Midwifery Council. The Code: professional standards of practice and behaviour for nurses, midwives and nursing associates. 2018. https://www.nmc.org.uk/globalassets/sitedocuments/nmc-publications/nmc-code.pdf (accessed Feb 6, 2022).

48 UK Caldicott Guardian Council. A manual for Caldicott Guardians. 2017. https://assets.publishing.service.gov.uk/government/uploads/system/uploads/attachment_data/file/581213/cgmanual.pdf (accessed Feb 6, 2022).

49 UK Information Commissioner's Office. Information provided in confidence (section 41): Freedom of Information Act. 2017. https://ico.org.uk/media/for-organisations/documents/1432163/information-provided-in-confidence-section-41.pdf (accessed Feb 4, 2022).

50 The Lord Chancellor. Mental Capacity Act 2005 Code of Practice. UK Department for Constitutional Affairs, 2007 https://www.gov.uk/government/publications/mental-capacity-act-code-of-practice.

51 UK. Mental Capacity Act 2005. 2005 https://www.legislation.gov.uk/ukpga/2005/9/contents.

52 UK. Mental Health Act 1983. 1983 https://www.legislation.gov.uk/ukpga/1983/20/contents.

53 UK Department of Health. Mental Health Act 1983: Code of Practice. UK Department of Health, 2015 https://www.gov.uk/government/publications/code-of-practice-mental-health-act-1983.

54 UK. Mental Health Act 2007. 2007 https://www.legislation.gov.uk/ukpga/2007/12/contents.

55 NHSX. Consent and confidential patient information. 2022; published online Feb 2. https://www.nhsx.nhs.uk/information-governance/guidance/consent-and-confidential-patient-information/ (accessed Feb 4, 2022).

For peer review only

56 UK Information Commissioner's Office. When is consent appropriate? 2019. https://ico.org.uk/for-organisations/guide-to-data-protection/guide-to-the-general-data-protection-regulation-gdpr/consent/when-is-consent-appropriate/ (accessed Feb 4, 2022).

57 NHS Health Research Authority. Is my study research? 2020. http://www.hra-decisiontools.org.uk/research/.

58 Kontopantelis E, Stevens RJ, Helms PJ, Edwards D, Doran T, Ashcroft DM. Spatial distribution of clinical computer systems in primary care in England in 2016 and implications for primary care electronic medical record databases: a cross-sectional population study. *BMJ Open* 2018; **8**: e020738.

59 Caldicott F. Information: To share or not to share? The Information Governance Review. 2013; published online March. https://www.gov.uk/government/publications/the-information-governance-review.

60 NHSX. Information Governance Framework for Integrated Health and Care: Shared Care Records. 2021. https://www.nhsx.nhs.uk/media/documents/NHSX_IG_Framework_V6.pdf (accessed Feb 5, 2022).

61 World Medical Association. Declaration of Helsinki - ethical principles for medical research involving human subjects. 2013. https://www.wma.net/policies-post/wma-declaration-of-helsinki-ethical-principles-for-medical-research-involving-human-subjects/ (accessed March 22, 2021).

62 Townend D. GDPR Brief: "At Least One" Legal Basis for Processing Under the GDPR: Clarifying Article 6(1). 2020; published online Jan 6. https://www.ga4gh.org/news/gdpr-brief-at-least-one-legal-basis-for-processing-under-the-gdpr-clarifying-article-61/ (accessed Feb 7, 2022).

63 Information Commissioner's Office. Guide to the General Data Protection Regulation (GDPR). 2021. https://ico.org.uk/media/for-organisations/guide-to-data-protection/guide-to-the-general-data-protection-regulation-gdpr-1-1.pdf (accessed Feb 5, 2022).

64 Rodway C, Tham S-G, Ibrahim S, *et al.* Suicide in children and young people in England: a consecutive case series. *Lancet Psychiatry* 2016; **3**: 751–9.

65 Downs JM, Ford T, Stewart R, *et al.* An approach to linking education, social care and electronic health records for children and young people in South London: a linkage study of child and adolescent mental health service data. *BMJ Open* 2019; **9**: e024355.

66 NHS Health Research Authority. CAG pre-application checklist: Do I need to process identifiable information without consent? 2018. https://s3.eu-west-2.amazonaws.com/www.hra.nhs.uk/media/documents/CAG_pre-application_checklist.pdf (accessed Feb 5, 2022).

67 Cross L, Carson LE, Jewell A, *et al.* Guidance for researchers wanting to link NHS data using non-consent approaches: a thematic analysis of feedback from the Health Research Authority Confidentiality Advisory Group. *Int J Popul Data Sci* 2020; **5**: 1355.

68 National Data Guardian for Health and Care. Review of Data Security, Consent and Opt-Outs. 2016. https://www.gov.uk/government/uploads/system/uploads/attachment_data/file/535024/data-security-review.PDF.

69 NHS. National Data Opt-out Operational Policy Guidance Document, v4.0. 2020; published online Feb 21. https://web.archive.org/web/20210204203917/https://digital.nhs.uk/binaries/content/assets/website-assets/services/national-data-opt-out/guidance-for-health-and-care-staff/ndopnationaldataoptoutpolicy_v4.0.pdf.

70 NHS Digital. DCB3058 Compliance with National Data Opt-outs: Requirements Specification. 2019 https://digital.nhs.uk/data-and-information/information-standards/information-standards-and-data-collections-including-extractions/publications-and-notifications/standards-and-collections/dcb3058-compliance-with-national-data-opt-outs.

71 Information Commissioner's Office. Anonymisation: managing data protection risk: code of practice. UK, 2012 https://ico.org.uk/media/1061/anonymisation-code.pdf (accessed Sept 9, 2015).

72 Culnane C, Rubinstein BIP, Teague V. Health Data in an Open World. *ArXiv171205627 Cs* 2017; published online Dec 15. http://arxiv.org/abs/1712.05627.

For peer review only

73 Mr Justice Cranston. Department of Health, R (on the application of) v Information Commissioner [2011] EWHC 1430 (Admin). 2011 https://www.bailii.org/ew/cases/EWHC/Admin/2011/1430.html (accessed Feb 5, 2022).

74 UK. Freedom of Information Act 2000. 2000 https://www.legislation.gov.uk/ukpga/2000/36/contents.

75 Information Commissioner's Office. ICO call for views: Anonymisation, pseudonymisation and privacy enhancing technologies guidance. 2021; published online May 28. https://ico.org.uk/about-the-ico/ico-and-stakeholder-consultations/ico-call-for-views-anonymisation-pseudonymisation-and-privacy-enhancing-technologies-guidance/ (accessed Feb 13, 2022).

76 Information Commissioner's Office. Chapter 1: introduction to anonymisation [Draft anonymisation, pseudonymisation and privacy enhancing technologies guidance]. 2021; published online May. https://ico.org.uk/media/about-the-ico/consultations/2619862/anonymisation-intro-and-first-chapter.pdf (accessed Feb 13, 2022).

77 Information Commissioner's Office. Chapter 2: How do we ensure anonymisation is effective? [Draft anonymisation, pseudonymisation and privacy enhancing technologies guidance]. 2021; published online Oct. https://ico.org.uk/media/about-the-ico/consultations/2619862/anonymisation-intro-and-first-chapter.pdf (accessed Feb 13, 2022).

78 Information Commissioner's Office. Chapter 3: pseudonymisation [Draft anonymisation, pseudonymisation and privacy enhancing technologies guidance]. 2022; published online Feb. https://ico.org.uk/media/about-the-ico/consultations/4019579/chapter-3-anonymisation-guidance.pdf (accessed Feb 13, 2022).

79 UK Information Commissioner's Office. What is personal data? 2019. https://ico.org.uk/for-organisations/guide-to-data-protection/guide-to-the-general-data-protection-regulation-gdpr/what-is-personal-data/what-is-personal-data/.

80 Griffiths E, Greci C, Kotrotsios Y, *et al.* Handbook on Statistical Disclosure Control for Outputs. 2019; published online July. https://ukdataservice.ac.uk/media/622521/thf_datareport_aw_web.pdf.

81 Cardinal RN. Clinical records anonymisation and text extraction (CRATE): an open-source software system. *BMC Med Inform Decis Mak* 2017; **17**: 50.

82 Stewart R, Soremekun M, Perera G, *et al.* The South London and Maudsley NHS Foundation Trust Biomedical Research Centre (SLAM BRC) case register: development and descriptive data. *BMC Psychiatry* 2009; **9**: 51.

83 Department for Health and Social Care, Hancock M. Coronavirus (COVID-19): notification to organisations to share information. 2020; published online April 1. https://www.gov.uk/government/publications/coronavirus-covid-19-notification-of-data-controllers-to-share-information.

84 Williamson EJ, Walker AJ, Bhaskaran K, *et al.* Factors associated with COVID-19-related death using OpenSAFELY. *Nature* 2020; **584**: 430–6.

85 Office for National Statistics. Estimates of the population for the UK, England and Wales, Scotland and Northern Ireland: mid-2019. 2020. https://www.ons.gov.uk/peoplepopulationandcommunity/populationandmigration/populationestimates/datasets/populationestimatesforukenglandandwalesscotlandandnorthernireland.

86 Office for National Statistics. Population estimates for the UK, England and Wales, Scotland and Northern Ireland: mid-2019. 2020; published online June 24. https://www.ons.gov.uk/peoplepopulationandcommunity/populationandmigration/populationestimates/bulletins/annualmidyearpopulationestimates/mid2019estimates (accessed Nov 6, 2020).

87 Office for National Statistics. UK population by ethnicity. 2018; published online Aug 1. https://www.ethnicity-facts-figures.service.gov.uk/uk-population-by-ethnicity (accessed Nov 6, 2020).

88 UK Data Service. Age by Highest level of qualification 2011. 2011. http://dx.doi.org/10.5257/census/aggregate-2011-2 (accessed Jan 19, 2021).

For peer review only

89 Office for National Statistics. Population characteristics research tables [2016 estimates]. 2019; published online Dec 4. https://www.ons.gov.uk/peoplepopulationandcommunity/populationandmigration/populationestimates/datasets/populationcharacteristicsresearchtables (accessed Jan 19, 2021).

For peer review only

S6. LIST OF APPENDICES

Appendix A shows a screenshot of the full REDCap design for the survey.

Appendix B shows resulting screenshots of the survey running in a web browser. (Questions labelled 11, 12, and 13 internally, as per **Appendix A**, were the same question, but with differing accompanying framing statements; the "concern" framing is shown here. The last content page is shown twice, once in its starting state and once fully expanded after partial completion with hypothetical data.)

Appendix C is a STROBE statement for cross-sectional studies (page numbers for composite PDF including main manuscript and supplementary materials).

For peer review only

Supplementary Figure 1. Recruitment sources, participation over time (shading indicates weekends), and survey completion. The denominator for percentages is the total number of consented participants.

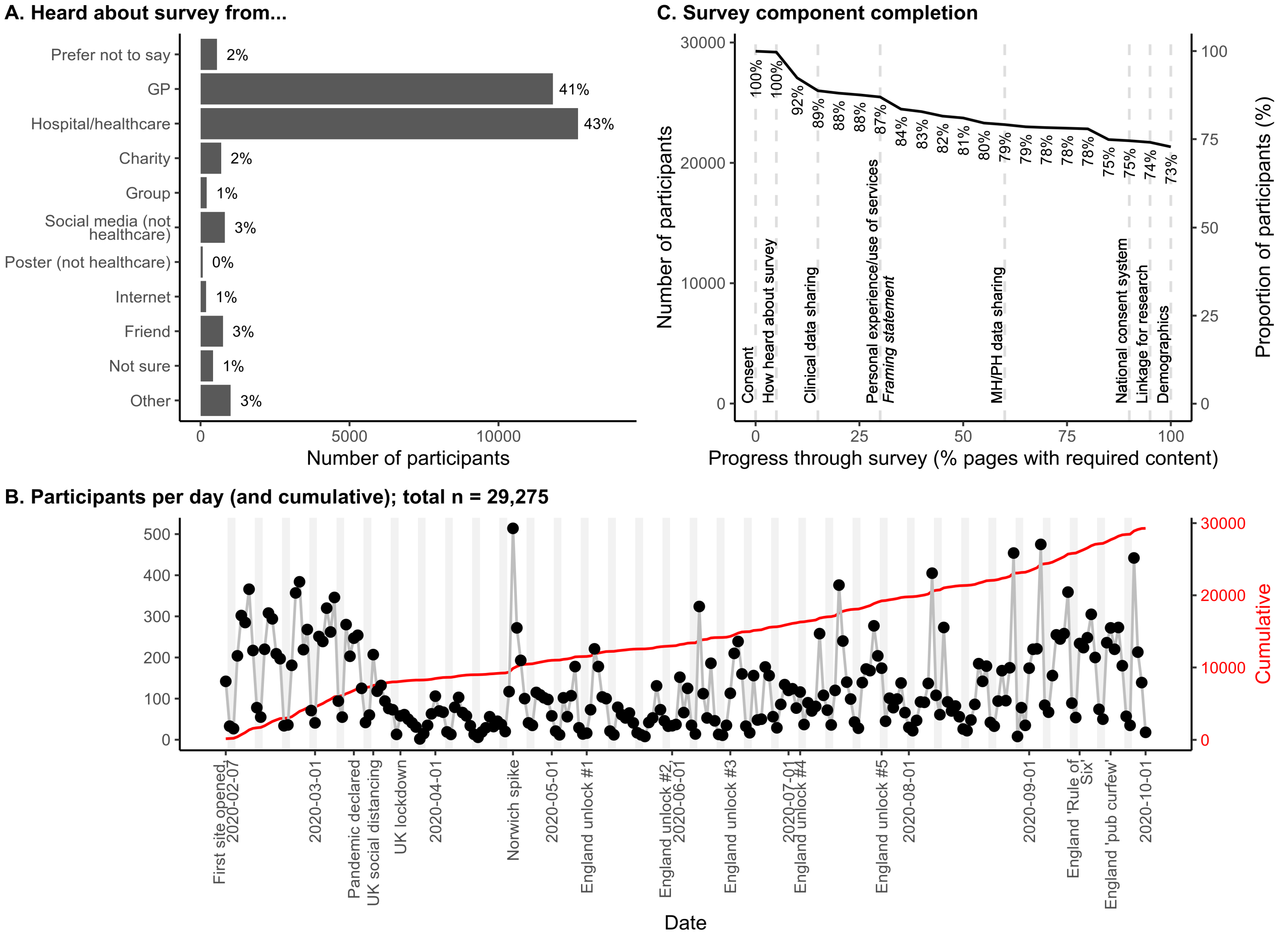

Supplementary Figure 2. Demographics of respondents. The denominator for percentages is the number of people who answered each question. For panels marked †, only participants who provided a postcode are included. ONS local authority map and deprivation centiles exclude the Channel Islands. See **Supplementary Methods S1.3** for details of geography and deprivation calculations.

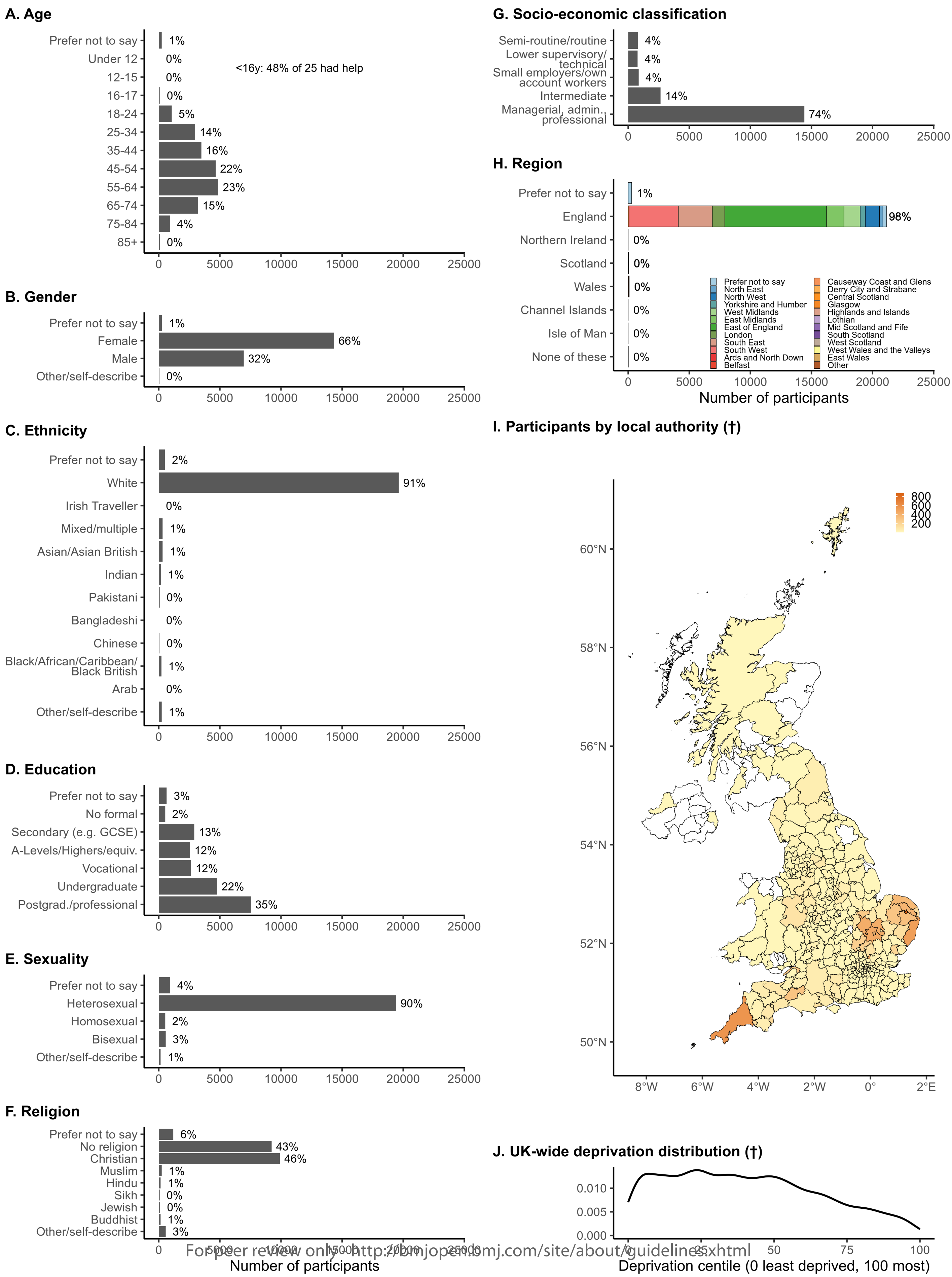

For peer review only - http://bmjopen.bmj.com/site/about/guidelines.xhtml

Supplementary Figure 3. Respondents' experience of mental health conditions/services and physical health services. The denominator for percentages is the number of people who answered each question.

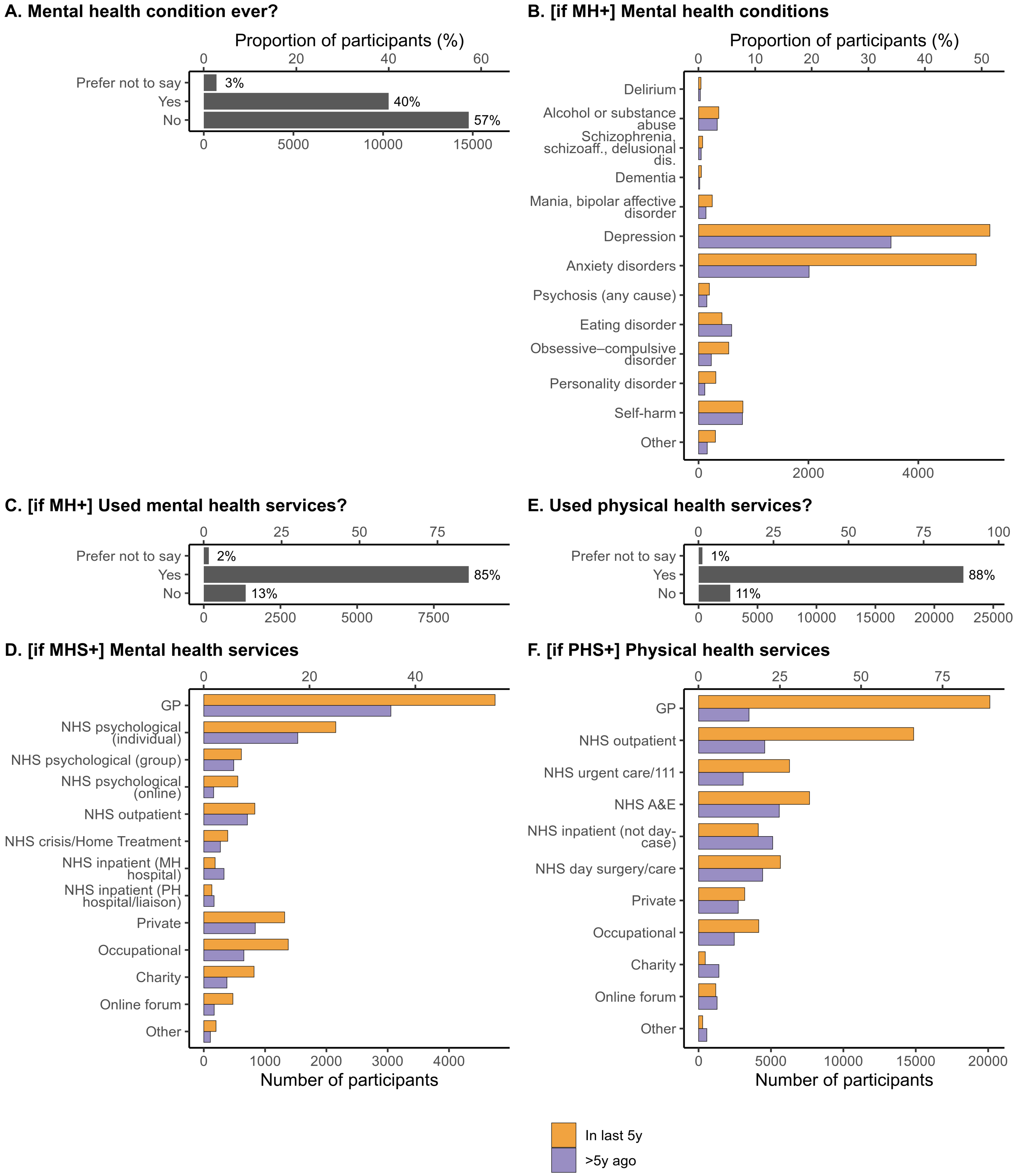

Supplementary Figure 4. Opinions about sharing mental and physical health data, weighted according to demographic variables. This is equivalent to **Figure 3** apart from the weighting (see **Supplementary Methods S1.4**), with identical conventions. “(W)”: weighted.

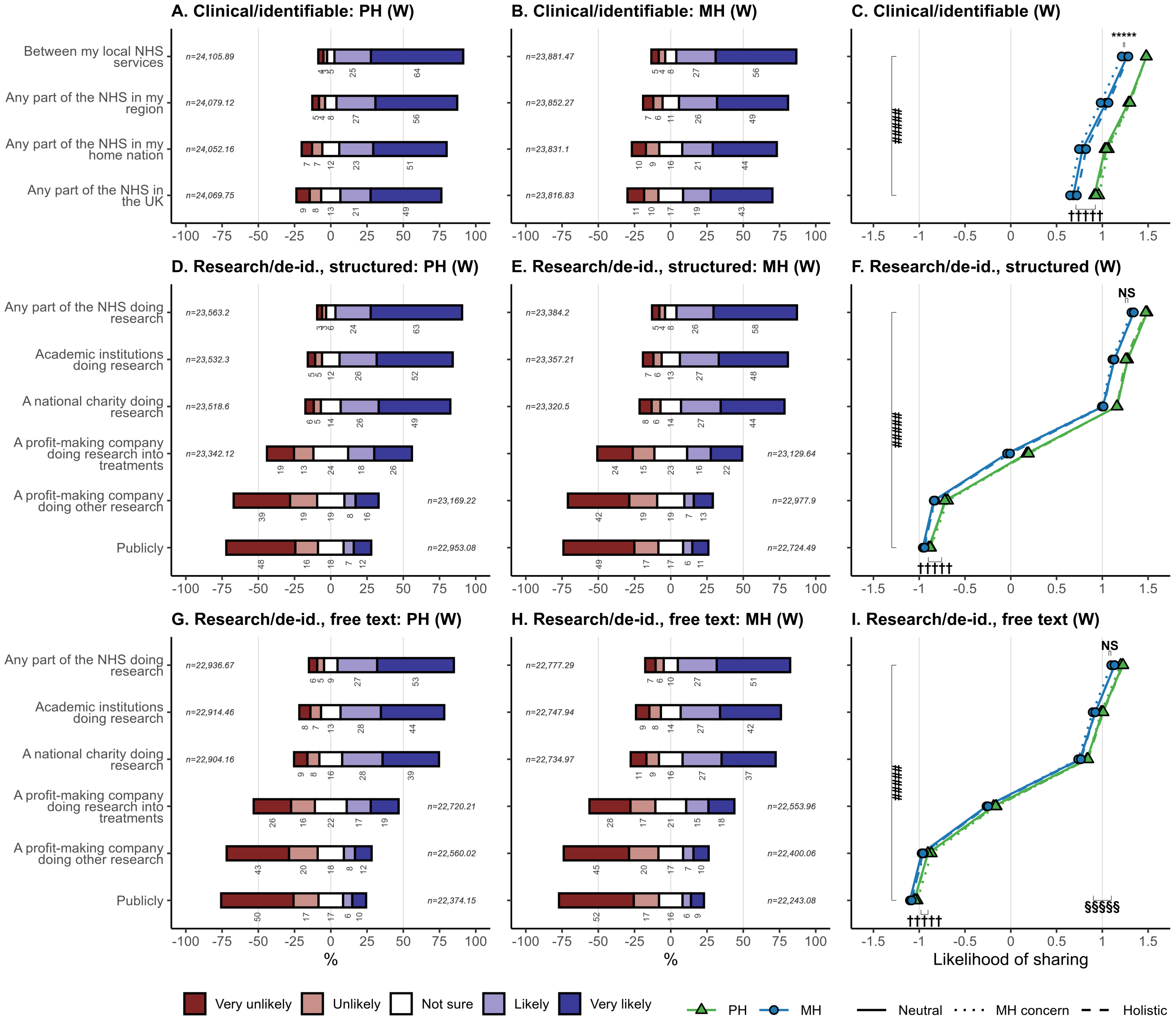

Supplementary Figure 5. Views on linkage to non-health data for research, weighted according to demographic variables. This is equivalent to **Figure 4** apart from the weighting (see **Supplementary Methods S1.4**). "(W)": weighted.

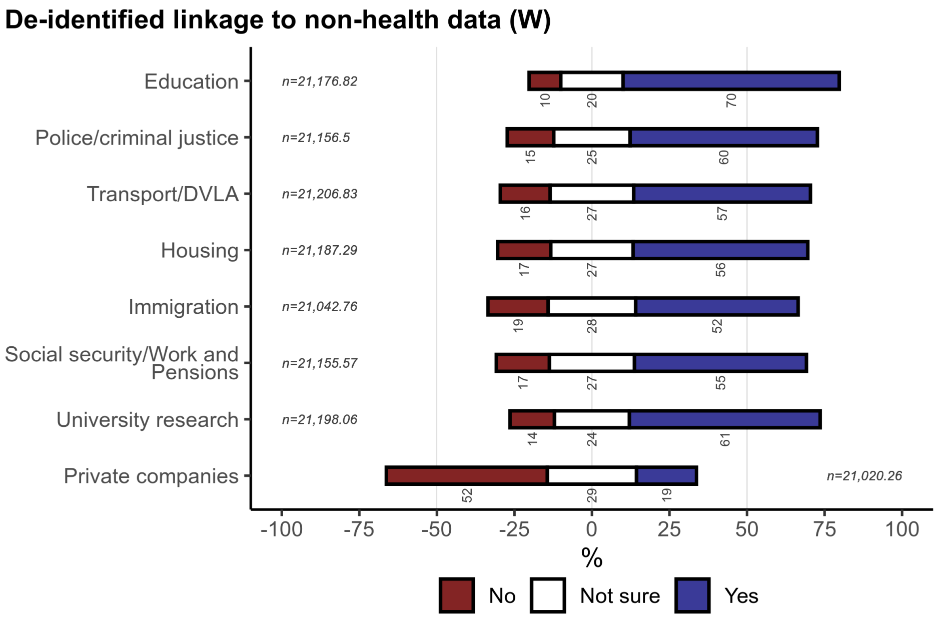

Supplementary Figure 6. Effect sizes for predictors of willingness to **(A)** share data for clinical purposes (compare **Figure 3C**), **(B)** share data for research purposes (compare **Figure 3F,H**), or **(C)** support linkage to non-health data for research (compare **Figure 4**). Analysis via models C1, R1, and L1 respectively. These models include all participants, including those not supplying full demographic details, and therefore do not include the demographic predictors; compare **Figure 6.** Conventions as for **Figure 6**.

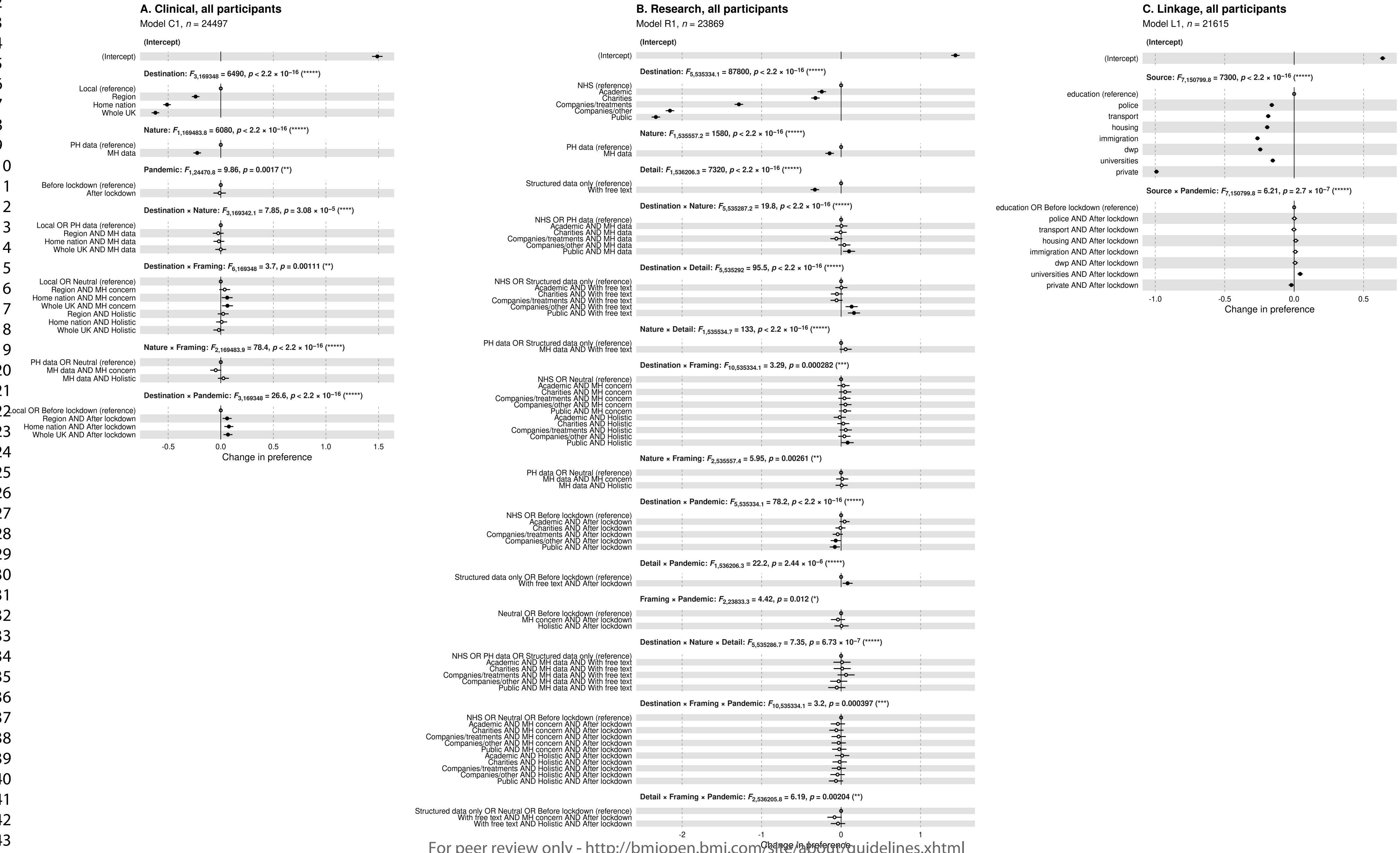

For peer review only - http://bmjopen.bmj.com/site/about/guidelines.xhtml

Supplementary Figure 7. Views on a national data sharing consent system, weighted according to demographic variables. This is equivalent to **Figure 7** apart from the weighting (see **Supplementary Methods S1.4**). "(W)": weighted.

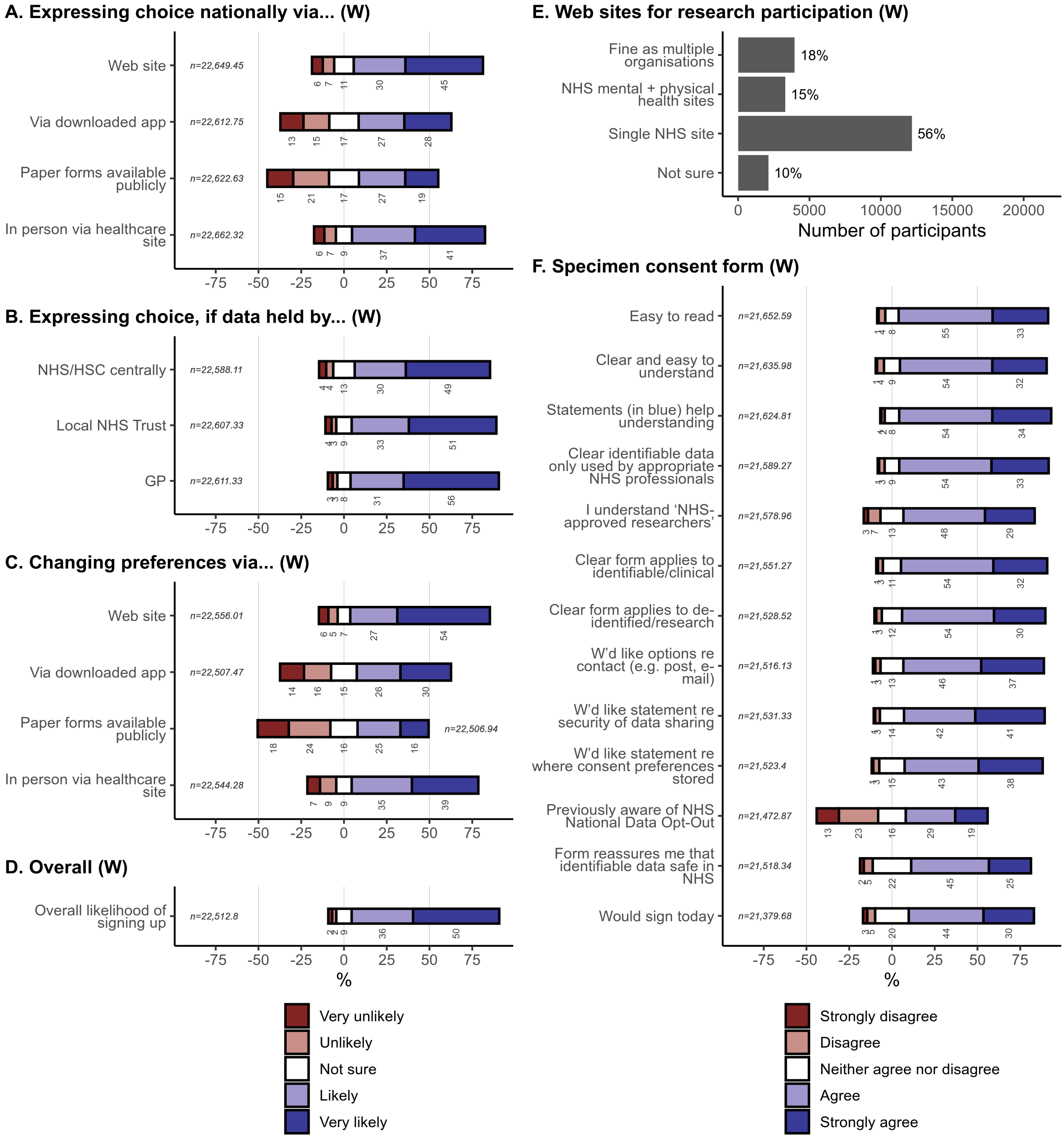

For peer review only - http://bmjopen.bmj.com/site/about/guidelines.xhtml

Supplementary Figure 8. Our understanding of UK data protection legislation relating to clinical and research uses of NHS data. We are not legal experts and this is not legal advice. Colours and data types correspond to **Figure 1.** All data are fictional. Not all aspects of the legal basis are shown and there are grey areas around the level of de-identification (see **Supplementary Discussion S3.2**). Abbreviations: Art., article; CAG, UK Confidentiality Advisory Group; CPIR, UK Control of Patient Information Regulations; DOB, date of birth; DPA, UK Data Protection Act 2018; GDPR, European Union General Data Protection Regulation; ID, identifier; HRA, NHS Health Research Authority; N/A, not applicable; NHSA, National Health Service Act 2006; REC, NHS research ethics committee; Reg., regulation; Sch., schedule; §, section; ¶, paragraph.

**Type of data**	**Confidential patient information**	**Pseudonymised health data**	**Patient-level anonymous health data**	**Aggregated anonymous health data**
**Example**	*John Smith, NHS# 1234567890<br>DOB 3 Jan 1948<br>Depression, heart failure, 84 kg<br><br>Rose Jones, NHS# 9876543210<br>DOB 21 Aug 2001<br>Epilepsy, diabetes, 52 kg*	*Research ID 840829<br>73 years old<br>Depression, heart failure, 84 kg<br><br>Research ID 451060<br>20 years old<br>Epilepsy, diabetes, 52 kg*	*73 years old<br>Depression, heart failure, 84 kg<br><br>20 years old<br>Epilepsy, diabetes, 52 kg*	*"52,000 people in this group have depression."<br>"15% of those with diabetes have depression."*
**Risk of identification**	Fully identifiable.	Re-identifiable via pseudonym map (e.g. NHS# ↔ research ID). De-identification might fail. Jigsaw attack possible.	Not "personal data", unless de-identification failure. However, jigsaw attack possible.	Very low risk after statistical disclosure control. Not "personal data". May be published freely.
**Legal basis (clinical)**	NHS clinical purpose.<br>► *GDPR Art. 6(1)(c,e), 9(2)(h)*<br>► *DPA §10(2), Sch.1 ¶2*	N/A	N/A	N/A
**Legal basis (research)**	(1) With consent:<br>► *GDPR Art. 6(1)(a), 9(2)(a); or as below*<br>► *DPA §2(1)(a), §84(2); or as below*<br>► *REC approval*<br><br>(2) Without consent: "Section 251" approval, with National Data Opt-Out.<br>► *NHSA §251*<br>► *CPIR Reg. 5*<br>► *GDPR Art. 6(1)(e), 9(2)(j), 89*<br>► *DPA §10(2), Sch.1 ¶4, §19(4)*<br>► *REC + CAG approval*	(1) With consent:<br>► *GDPR Art. 6(1)(a), 9(2)(a); or as below*<br>► *DPA §2(1)(a), §84(2); or as below*<br>► *REC approval*<br><br>(2) Without consent:<br>► *GDPR Art. 6(1)(e), 9(2)(j), 89*<br>► *DPA §10(2), Sch.1 ¶4, §19(4)*	Not "personal data" if de-identified. However, HRA ± REC approval advisable for sensitive data (the more information, the greater the potential for jigsaw attack).	No permissions needed.

For peer review only - http://bmjopen.bmj.com/site/about/guidelines.xhtml

University of Cambridge NHS Health Data Consent Survey

Project Home | Project Setup | Online Designer | Data Dictionary | Codebook

Create snapshot of instruments   VIDEO: How to use this page

Last snapshot: 28/08/2019 10:22am ?

This page allows you to build and customize your data collection instruments one field at a time. You may add new fields or edit existing ones. New fields may be added by clicking the Add Field buttons. You can begin editing an existing field by clicking on the **Edit** icon. If you decide that you do not want to keep a field, you can simply delete it by clicking on the **Delete** icon. To reorder the fields, simply **drag and drop** a field to a different position within the form below. NOTE: While in development status, all field changes will take effect immediately in real time. Are you using Action Tags yet? If not, learn about Action Tags here.

Return to list of instruments   Survey settings

Current instrument: **NHS Data Consent Survey**   Preview instrument

Variable: record_id   * This field will NOT be displayed on the survey page.

Record ID

NOTE: The field above is the record ID field and thus cannot be deleted or moved. It can only be edited.

Add Field   Add Matrix of Fields

Variable: random_no_gen

Random number generator   View equation

Add Field   Add Matrix of Fields

Variable: random_no_min

Minimum random number

Add Field   Add Matrix of Fields

Variable: random_no_max

Maximum random number

Add Field   Add Matrix of Fields

Variable: random_no

Random number   View equation

Add Field   Add Matrix of Fields

Variable: date_time_1

Now   D-M-Y H:M

Add Field   Add Matrix of Fields

Matrix group: consent

Consent Form

Variable: consent_1

Please tick ALL boxes

I confirm I am aged 16 or over, or I am under 16 years of age but have the consent of my parent/guardian.
* must provide value

Variable: consent_2

I confirm that I am a resident of the UK.
* must provide value

Variable: consent_3

I understand I may quit the survey at any time but any data I have provided may still be used.
* must provide value

Variable: consent_4

I understand that the answers I give will be used for analysis and publication.
* must provide value

Variable: consent_5

I understand my answers will be recorded in a way that means I cannot be identified.
* must provide value

Variable: consent_6

I understand that not everyone will see the same questions or definitions.
* must provide value

Variable: consent_7

If I have any problems or questions with the survey, I understand I can contact Linda Jones, e-mail: laj28@cam.ac.uk, telephone: 01223 764670
* must provide value

Variable: consent_8

If I choose to leave an email address, I understand it will be removed from the survey and only used to send me the results of the survey
* must provide value

Variable: consent_9

I consent to take part in this survey.
* must provide value

Add Field | Add Matrix of Fields

Variable: demo_advertising

We would like to know where you heard about this survey. It is especially important to know if you were given a leaflet (or letter) or saw a poster/text/social media from a 'healthcare provider' e.g. hospital, clinic, GP, ambulance service, healthcare community setting, or any other healthcare provider.

* must provide value

- Prefer not to say
- GP (via a poster, leaflet, from a staff member, via text, via GP Practice social media etc.)
- Hospital/Clinic/Ambulance/Healthcare community setting, other healthcare provider (via a poster, leaflet, from a staff member, text, healthcare provider's social media etc.)
- Group to which I belong
- Charity website/email/newsletter etc.
- Social media (other than from a healthcare provider)
- Poster (other than at a healthcare provider)
- Browsing the internet
- Friend
- Not sure
- Other

reset

Add Field | Add Matrix of Fields

Variable: healthcare_provider *Branching logic:* [demo_advertising] = '3'

Please could you tell us in which healthcare establishment you saw a poster or picked up/were given a leaflet? If you have the leaflet, the healthcare setting may be named on the back of it. This is really important to each healthcare provider. (Please type in a few letters and options will appear.)

* must provide value

Add Field | Add Matrix of Fields

Variable: gp *Branching logic:* [demo_advertising] = '2'

Please could you tell us in which GP Practice (surgery) you saw a poster or picked up/were given a leaflet? If you have the leaflet, the practice may be named on the back of it. This is really important to the GPs. (Please type in a few letters and options will appear.)

* must provide value

Add Field | Add Matrix of Fields

Before starting the survey please read the following definitions:

By "NHS" we mean all parts of the national health service in the UK: NHS England, NHS Scotland, NHS Wales, and Health and Social Care in Northern Ireland (HSC). We refer to all these as the NHS in this survey.

Health data means any information about a person's health such as might be collected by a nurse, doctor or other appropriate member of NHS staff. Health data includes things like your medical notes, blood test results, prescription information, scans, etc.

Identifiable health data means any health data (as described above) that can also identify a person -- such as any health data that also contains names, addresses, NHS numbers, etc.

Clinical care means providing health care directly to people -- for example, diagnosing illnesses or treating ill health.

Add Field | Add Matrix of Fields

Variable: understanding

We would like to know/gauge your understanding of how NHS clinical care providers currently share your identifiable health data for your clinical care, without asking you each time. If you are not sure, please have a "best guess" as to what you think might happen.

My understanding is that (please tick only one):

* must provide value

- My identifiable health data is currently shared freely between all NHS sites across the UK -- i.e. all acute hospital Trusts, my GP, all mental health Trusts, ambulance services, etc., in the whole UK.
- My identifiable health data is currently shared between all NHS sites but only in the home nation that I live in -- i.e. shared only in England, or Scotland, or Northern Ireland, or Wales, but not between them.
- My identifiable health data is currently only shared between NHS sites in my region (cluster of hospitals nearest to my local hospital).
- My identifiable health data is currently only shared between my GP, acute local hospital, local mental health Trust, and other local services.
- My identifiable health data is currently not shared between any NHS sites.

reset

Add Field | Add Matrix of Fields

MOST hospital Trusts, GPs, mental health Trusts are separate and currently DO NOT share identifiable health data without asking you, though they correspond and communicate with each other with your permission.

For peer review only - http://bmjopen.bmj.com/site/about/guidelines.xhtml

Add Field | Add Matrix of Fields

Variable: views_on_sharing

In view of the statement above, we would now like to ask you your views on sharing your own identifiable health data for YOUR CLINICAL CARE, without having to be asked each time (please tick only one):

* must provide value

- I would like my identifiable health data to be shared for clinical purposes with any part of the NHS in the UK, without asking me first.
- I would like my identifiable health data to be shared for clinical purposes with any part of the NHS in my home nation only (i.e. only in England, or only in Northern Ireland, or only in Scotland, or only in Wales) without asking me first.
- I would like my identifiable health data to be shared for clinical purposes with any part of my region (cluster of hospitals nearest to my local hospital), without asking me first.
- I would like my identifiable health data to be shared for clinical purposes only with my GP, local hospital, and local mental health services, without asking me first.
- No one in the NHS should share my identifiable health data without asking me first.
- Not sure.

reset

Add Field | Add Matrix of Fields

We would now like to ask you some questions about your own MENTAL and PHYSICAL health. You can "prefer not to say" if you wish.

Add Field | Add Matrix of Fields

Variable: mh_yes_or_no

We would like to ask if you have had a MENTAL health condition either recently or at some point in your life.

By mental health conditions we include things like delirium, self harm, substance abuse, dementia, mania, schizophrenia, depression, anxiety disorders, psychosis, eating disorders, OCD, personality disorders, etc.

* must provide value

- I prefer not to say.
- Yes, I have had a mental health condition either recently or at some point in my life.
- No, I have never had a mental health condition.

reset

Add Field | Add Matrix of Fields

Matrix group: mh_conditions

We would like to know if you would be willing to share what mental health condition you have had.

You may tick as many boxes as you wish.

If you prefer not to say, please use the "next page" button below to move on.

Variable: delirium *Branching logic:* [mh_yes_or_no] = '1'

	In the last 5 years	More than 5 years ago
**Delirium**	☐	☐

Variable: alcohol_substance *Branching logic:* [mh_yes_or_no] = '1'

**Alcohol or substance abuse**	☐	☐

Variable: schiz *Branching logic:* [mh_yes_or_no] = '1'

**Schizophrenia, schizoaffective disorder, or delusional disorder**	☐	☐

Variable: dementia *Branching logic:* [mh_yes_or_no] = '1'

**Dementia**	☐	☐

Variable: mania_bipolar *Branching logic:* [mh_yes_or_no] = '1'

Mania or bipolar affective disorder

Variable: depression *Branching logic:* [mh_yes_or_no] = '1'

Depression

Variable: anxiety *Branching logic:* [mh_yes_or_no] = '1'

Anxiety disorder (e.g. phobia, panic, generalized anxiety disorder, post-traumatic stress disorder)

Variable: psychosis *Branching logic:* [mh_yes_or_no] = '1'

Psychosis of any cause

Variable: eating_disorder *Branching logic:* [mh_yes_or_no] = '1'

Eating disorder (e.g. anorexia nervosa, bulimia)

Variable: ocd *Branching logic:* [mh_yes_or_no] = '1'

Obsessive-compulsive disorder

Variable: personality_disorder *Branching logic:* [mh_yes_or_no] = '1'

Personality disorder

Variable: self_harm *Branching logic:* [mh_yes_or_no] = '1'

Self harm

Variable: other_mh_illness *Branching logic:* [mh_yes_or_no] = '1'

Other

Add Field | Add Matrix of Fields

Variable: mh_other *Branching logic:* [other_mh_illness(1)] = '1' or [other_mh_illness(2)] = '1'

Other (please specify)

Add Field | Add Matrix of Fields

And also in relation to mental health:

Add Field | Add Matrix of Fields

Variable: mh_services_yes_no *Branching logic:* [mh_yes_or_no] = '1'

We would also like to know if you have accessed any mental health services for your own mental health condition.

These might include your GP, online services, mental health forums, psychological therapy services, crisis care, home treatments or visits, outpatient or inpatient care, services from the private sector, support from a mental health charity, etc.

* must provide value

- I prefer not to say.
- I have used mental health services.
- I have not used any mental health services (not even my GP or surgery) for my mental health condition.

reset

Add Field | Add Matrix of Fields

Matrix group: mh_services

Variable: mh_gp *Branching logic:* [mh_services_yes_no] = '1'

	In the last 5 years	More than 5 years ago
**Mental health support from your GP or surgery**	☐	☐

Variable: mh_iapt_individual *Branching logic:* [mh_services_yes_no] = '1'

An NHS psychological therapy service, e.g. IAPT or other therapy service (INDIVIDUAL session)

Variable: mh_iapt_group *Branching logic:* [mh_services_yes_no] = '1'

An NHS psychological therapy service, e.g. IAPT or other therapy service (GROUP session)

Variable: mh_iapt_online *Branching logic:* [mh_services_yes_no] = '1'

An NHS psychological therapy service, e.g. IAPT or other therapy service (ONLINE)

Variable: mh_op *Branching logic:* [mh_services_yes_no] = '1'

NHS outpatient or community mental health services, or key worker

Variable: mh_crisis *Branching logic:* [mh_services_yes_no] = '1'

NHS crisis care/Home Treatment teams

Variable: mh_ip_mh *Branching logic:* [mh_services_yes_no] = '1'

NHS inpatient mental health services in a mental health hospital

Variable: mh_ip_general *Branching logic:* [mh_services_yes_no] = '1'

NHS inpatient mental health services in a general hospital (liaison psychiatry)

Variable: mh_private *Branching logic:* [mh_services_yes_no] = '1'

Private services

Variable: mh_oh *Branching logic:* [mh_services_yes_no] = '1'

Services in the workplace e.g. Occupational Health

Variable: mh_charity *Branching logic:* [mh_services_yes_no] = '1'

Charity providing mental health support

Variable: mh_forum *Branching logic:* [mh_services_yes_no] = '1'

Online forum

Variable: mh_other_service *Branching logic:* [mh_services_yes_no] = '1'

Other

Add Field | Add Matrix of Fields

Variable: mh_service_other_detail *Branching logic:* [mh_other_service(1)] = '1' or [mh_other_service(2)] = '1'

Other (please specify)

Add Field | Add Matrix of Fields

We are also interested to know if you have accessed any services regarding your own PHYSICAL health.

This could be services like your GP, outpatient hospital appointments, inpatient services, online support, Accident and Emergency, 111 service, private services, charities providing physical health support, etc.

Add Field | Add Matrix of Fields

Variable: ph_services_yes_no

Have you ever used any of the following services for your own PHYSICAL health?

* must provide value

- I prefer not to say
- I have used physical health services (this includes using your GP)
- I have not used any physical health services

reset

Add Field | Add Matrix of Fields

Matrix group: ph_services

And finally in this section, we would like to know if you would be willing to share what physical health services you have used for your own physical health.

You may tick as many boxes as you wish.

Please use the 'next page' button if you prefer not to answer.

Variable	Branching logic	Item	In the last 5 years	More than 5 years ago
ph_gp	[ph_services_yes_no] = '1'	Physical health support from your GP or surgery	☐	☐
ph_op	[ph_services_yes_no] = '1'	NHS hospital outpatient services (outpatient clinics etc.)	☐	☐
ph_urgent_care	[ph_services_yes_no] = '1'	NHS Urgent Care Centre (not A&E) or NHS 111 helpline	☐	☐
ph_accident	[ph_services_yes_no] = '1'	NHS Accident and Emergency services (A&E)	☐	☐
ph_ip	[ph_services_yes_no] = '1'	NHS inpatient services (procedures not completed in 1 day)	☐	☐
ph_day	[ph_services_yes_no] = '1'	NHS Day Surgery/day care in a hospital	☐	☐
ph_private	[ph_services_yes_no] = '1'	Private services	☐	☐
ph_oh	[ph_services_yes_no] = '1'	Services in the workplace e.g. Occupational Health	☐	☐
ph_charity	[ph_services_yes_no] = '1'	Charity providing physical health support	☐	☐
ph_online	[ph_services_yes_no] = '1'	Online forum	☐	☐
ph_other_services	[ph_services_yes_no] = '1'	Other	☐	☐

Add Field | Add Matrix of Fields

Variable: ph_other *Branching logic:* [ph_other_services(1)] = '1' or [ph_other_services(2)] = '1'

Other (please specify)

Add Field | Add Matrix of Fields

Moving on...

Add Field | Add Matrix of Fields

Variable: identifiable_description

The table below shows what NHS identifiable health data may look like. This sort of data is used for health care by NHS staff. (Please note the examples shown are not real.)

NHS number	Title	Forename	Surname	Date of birth	Address	Diagnoses	Notes
9876543210	Mr	John	Smith	20 Jan 1950	1 The Street, Maidstone	pneumonia<br>high blood pressure	1 Apr 2019. Seen in clinic. Mr Smith has a fever and a cough. His chest sounds crackly. I think he has pneumonia. Sent to hospital.
8765432109	Miss	Alice	Jones	16 Jun 1994	15 The Road, Dundee	depression<br>carpal tunnel syndrome	2 Apr 2019. Alice rates her mood as 1/10 on average, she is more tired, and she is not looking forward to anything. Her depression has worsened. She wishes to restart citalopram.
7654321098	Mrs	Chloe	Williams	4 May 1930	5 Tree Close, Cardiff	broken humerus<br>anxiety	3 Apr 2019. Chloe fell after skidding on an oily patch while cycling and was hit by a car. Has an obvious fracture of her left upper arm. Needs X-ray.
6543210987	Mr	Pradeep	Agarwal	22 Sep 1973	27 The Mews, London	schizophrenia<br>diabetes	4 Apr 2019. Pradeep still worries that other people can hear his thoughts, but this is getting less common. He is attending cognitive–behavioural therapy sessions.

Add Field | Add Matrix of Fields

Matrix group: share_ph_1

Previous surveys have found that people have more concerns about the use of their identifiable health data relating to their mental health than other aspects of their physical health care.

Regardless of whether you have a PHYSICAL health condition or have used PHYSICAL health services:

How likely are you to agree to share your identifiable PHYSICAL health data for your clinical care with the following, without being asked every time?

Variable: share_ph_local_1 *Branching logic:* [random_no] = '1'

	Very likely	Likely	Not sure	Unlikely	Very unlikely	Prefer not to say
**Between my local NHS services -- i.e. between my local physical and mental health hospitals, my GP, etc.** * must provide value	○	○	○	○	○	○

reset

Variable: share_ph_region_1 *Branching logic:* [random_no] = '1'

**Any part of the NHS in my region (cluster of hospitals nearest to my local hospital)** * must provide value	○	○	○	○	○	○

reset

Variable: share_ph_nation_1 *Branching logic:* [random_no] = '1'

**Any part of the NHS in my home nation only (i.e. only in England, or only in Northern Ireland, or only in Scotland, or only in Wales)** * must provide value	○	○	○	○	○	○

reset

Variable: share_ph_uk_1 *Branching logic:* [random_no] = '1'

**Any part of the NHS in the UK** * must provide value	○	○	○	○	○	○

reset

Add Field Add Matrix of Fields

Matrix group: share_ph_2

We would like to find out your perspective on using information about your mental health and your physical health.

Regardless of whether you have a PHYSICAL health condition or have used PHYSICAL health services:

How likely are you to agree to share your identifiable PHYSICAL health data for your clinical care with the following, without being asked every time?

Variable: share_ph_local_2 *Branching logic:* [random_no] = '2'

	Very likely	Likely	Not sure	Unlikely	Very unlikely	Prefer not to say
**Between my local NHS services -- i.e. between my local physical and mental health hospitals, my GP, etc.** * must provide value	○	○	○	○	○	○

reset

Variable: share_ph_region_2 *Branching logic:* [random_no] = '2'

**Any part of the NHS in my region (cluster of hospitals nearest to my local hospital)** * must provide value	○	○	○	○	○	○

reset

Variable: share_ph_nation_2 *Branching logic:* [random_no] = '2'

**Any part of the NHS in my home nation only (i.e. only in England, or only in Northern Ireland, or only in Scotland, or only in Wales)** * must provide value	○	○	○	○	○	○

reset

Variable: share_ph_uk_2 *Branching logic:* [random_no] = '2'

**Any part of the NHS in the UK** * must provide value	○	○	○	○	○	○

reset

Add Field | Add Matrix of Fields

Matrix group: share_ph_3

Mental and physical illnesses overlap, so holistic health care is important. Mental health problems have physical consequences, and physical illnesses have important consequences for mental health.

Regardless of whether you have a PHYSICAL health condition or have used PHYSICAL health services:

How likely are you to agree to share your identifiable PHYSICAL health data for your clinical care with the following, without being asked every time?

Variable: share_ph_local_3 *Branching logic:* [random_no] = '3'

	Very likely	Likely	Not sure	Unlikely	Very unlikely	Prefer not to say
**Between my local NHS services -- i.e. between my local physical and mental health hospitals, my GP, etc.** * must provide value	◯	◯	◯	◯	◯	◯

reset

Variable: share_ph_region_3 *Branching logic:* [random_no] = '3'

**Any part of the NHS in my region (cluster of hospitals nearest to my local hospital)** * must provide value	◯	◯	◯	◯	◯	◯

reset

Variable: share_ph_nation_3 *Branching logic:* [random_no] = '3'

**Any part of the NHS in my home nation only (i.e. only in England, or only in Northern Ireland, or only in Scotland, or only in Wales)** * must provide value	◯	◯	◯	◯	◯	◯

reset

Variable: share_ph_uk_3 *Branching logic:* [random_no] = '3'

**Any part of the NHS in the UK** * must provide value	◯	◯	◯	◯	◯	◯

reset

Add Field | Add Matrix of Fields

Matrix group: share_mh

Regardless of whether you have a MENTAL health condition or have used MENTAL health services:

How likely are you to agree to share your identifiable MENTAL health data for your clinical care with the following, without being asked every time?

Variable: share_mh_local

	Very likely	Likely	Not sure	Unlikely	Very unlikely	Prefer not to say
**Between my local NHS services -- i.e. between my local physical and mental health hospitals, my GP, etc.** * must provide value	◯	◯	◯	◯	◯	◯

reset

Variable: share_mh_region

**Any part of the NHS in my region (cluster of hospitals nearest to my local hospital)** * must provide value	◯	◯	◯	◯	◯	◯

reset

Variable: share_mh_nation

Any part of the NHS in my home nation only (i.e. only in England, or only in Northern Ireland, or only in Scotland, or only in Wales)
* must provide value

reset

Variable: share_mh_uk

Any part of the NHS in the UK
* must provide value

reset

Add Field Add Matrix of Fields

In the next two questions we would like to ask you about sharing your NHS data for research purposes. Research is used to improve overall healthcare for everyone.

Add Field Add Matrix of Fields

Variable: deidentified_description

Firstly, please look at some of the NHS health care data that you saw previously, which has now been de-identified.

Research ID	Age	Sex	Region	Diagnoses
5X62V	69	M	Kent	pneumonia<br>high blood pressure
597PT	24	F	Dundee	depression<br>carpal tunnel syndrome
8HG7S	89	F	Glamorganshire	broken humerus<br>anxiety
BA6A9	45	M	London	schizophrenia<br>diabetes

Add Field Add Matrix of Fields

Matrix group: ph_non_ident_research

How likely would you be to share your de-identified PHYSICAL health data with the following, without giving consent every time?

Variable: ph_non_id_nhs

	Very likely	Likely	Not sure	Unlikely	Very unlikely	Prefer not to say
**Any part of the NHS doing research** * must provide value	○	○	○	○	○	○

reset

Variable: ph_non_id_academic

Academic institutions doing research (e.g. universities)
* must provide value

reset

Variable: ph_non_id_charity

A national charity doing research (e.g. MIND, Cancer Research UK, British Heart Foundation)
* must provide value

reset

Variable: ph_non_id_profit_tx

A profit-making company doing research into treatments (e.g. a pharmaceutical company, health technology company)
* must provide value

reset

Variable: ph_non_id_profit_other

A profit-making company doing other research (e.g. an insurance company, broadband provider)
* must provide value

reset

Variable: ph_non_id_public

Publicly
* must provide value

reset

Add Field | Add Matrix of Fields

Matrix group: mh_non_id_research

How likely would you be to share your de-identified MENTAL health data with the following, without giving consent every time:

Variable: mh_non_id_nhs

	Very likely	Likely	Not sure	Unlikely	Very unlikely	Prefer not to say
**Any part of the NHS doing research** * must provide value	○	○	○	○	○	○

reset

Variable: mh_non_id_academic

Academic institutions doing research (e.g. universities)
* must provide value

reset

Variable: mh_non_id_charity

A national charity doing research (e.g. MIND, Cancer Research UK, British Heart Foundation)
* must provide value

reset

Variable: mh_non_id_profit_tx

A profit-making company doing research into treatments (e.g. pharmaceutical company, health technology company)
* must provide value

reset

Variable: mh_non_id_profit_other

A profit-making company doing other research (e.g. insurance company, broadband provider)
* must provide value

reset

Variable: mh_non_id_public

Publicly
* must provide value

reset

Add Field | Add Matrix of Fields

The final question in this section relates to sharing de-identified "free text" data for research.

Add Field | Add Matrix of Fields

Variable: deidentified_ft_descript

Here is the table of de-identified health care data you saw earlier, but this time a column labelled "Notes" has been added. This is known as "free text" data.

Research ID	Age	Sex	Region	Diagnoses	Notes
5X62V	69	M	Kent	pneumonia<br>high blood pressure	1st April 2019. XXX has a fever and cough. His chest sounds crackly. I think he has pneumonia. Sent to hospital.
597PT	24	F	Dundee	depression<br>carpal tunnel syndrome	2nd April 2019. XXX rates her mood as 1/10 on average, she is more tired, and she is not looking forward to anything. Her depression has worsened. She wishes to restart citalopram.
8HG7S	89	F	Glamorganshire	broken humerus<br>anxiety	3rd April 2019. XXX fell after skidding on an oily patch while cycling and was hit by a car. She has an obvious fracture of her left upper arm. She needs an X-ray.
BA6A9	45	M	London	schizophrenia<br>diabetes	4th April 2019. XXX still worries that other people can hear his thoughts, but this is getting less common. He is attending cognitive-behavioural therapy sessions.

Like the last example the data remains de-identified but gives the researchers more information. As there is more information there is a slightly increased risk of someone being identified from the data.

For example, in the table above, an "89-year-old cyclist skidding on oil and being hit by a car" may be reported in a local newspaper. Whilst the lady is not named in the data above, she would almost certainly be named in the newspaper and it may therefore be possible for researchers to work out who she is.

Add Field | Add Matrix of Fields

Matrix group: ph_free_text

How likely would you be to share your FREE TEXT de-identified PHYSICAL health data with the following, without giving consent every time:

	Very likely	Likely	Not sure	Unlikely	Very unlikely	Prefer not to say
Variable: ph_ft_nhs<br>**Any part of the NHS doing research**<br>* must provide value	○	○	○	○	○	○
Variable: ph_ft_academic<br>**Academic institutions doing research (e.g. universities)**<br>* must provide value	○	○	○	○	○	○
Variable: ph_ft_charity<br>**A national charity doing research (e.g MIND, Cancer Research UK, British Heart Foundation)**<br>* must provide value	○	○	○	○	○	○
Variable: ph_ft_profit_tx<br>**A profit-making company doing research into treatments (e.g. pharmaceutical company, health technology company)**<br>* must provide value	○	○	○	○	○	○

reset

Variable: ph_ft_profit_other

A profit-making company doing other research (e.g. insurance company, broadband provider)
* must provide value

reset

Variable: ph_ft_public

Publicly
* must provide value

reset

Add Field | Add Matrix of Fields

Matrix group: mh_free_text

How likely would you be to share your FREE TEXT de-identified MENTAL health data with the following, without giving consent each time:

Variable: mh_ft_nhs

	Very likely	Likely	Not sure	Unlikely	Very unlikely	Prefer not to say
**Any part of the NHS doing research** * must provide value	○	○	○	○	○	○

reset

Variable: mh_ft_academic

Academic institutions doing research (e.g. universities)
* must provide value

reset

Variable: mh_ft_charity

A national charity doing research (e.g MIND, Cancer Research UK, British Heart Foundation)
* must provide value

reset

Variable: mh_ft_profit_tx

A profit-making company doing research into treatments (e.g. pharmaceutical company, health technology company)
* must provide value

reset

Variable: mh_ft_profit_other

A profit-making company doing other research (e.g. insurance company, broadband provider)
* must provide value

reset

Variable: mh_ft_public

Publicly
* must provide value

reset

Add Field | Add Matrix of Fields

Matrix group: consent_signup_place

On a slightly different topic...

Imagine that there was a single place where you could securely sign up to choose how your NHS identifiable health data is used. This would cover how your confidential patient information is managed across all UK NHS services, and how your NHS de-identified data could (or could not) be used for research.

It would be in the form of a standardised UK-wide CONSENT FORM that all NHS services could use.

How likely would you be to sign up if you were asked to sign:

Variable: signup_online

Variable: signup_app

Variable: signup_paper

Variable: signup_in_person

	Very likely	Likely	Not sure	Unlikely	Very unlikely	Prefer not to say
**Online (via a website)** * must provide value	○	○	○	○	○	○
**By downloading an app** * must provide value	○	○	○	○	○	○
**On paper forms available from pharmacies, the Post Office, etc. (and sent by post)** * must provide value	○	○	○	○	○	○
**In person (e.g. when attending your GP, at a hospital clinic, etc.)** * must provide value	○	○	○	○	○	○

reset

Add Field | Add Matrix of Fields

Matrix group: consent_stored

Would you be likely to sign up if your consent form were then stored and managed securely by the following institutions?

Variable: stored_centrally

Variable: stored_locally

Variable: stored_gp

	Very likely	Likely	Not sure	Unlikely	Very unlikely	Prefer not to say
**Centrally by NHS England/NHS Scotland/NHS Wales/HSC Northern Ireland** * must provide value	○	○	○	○	○	○
**Your local NHS Trust** * must provide value	○	○	○	○	○	○
**Your GP** * must provide value	○	○	○	○	○	○

reset

Add Field | Add Matrix of Fields

Matrix group: consent_change

If you wanted to change your preferences, how likely would you be to use the following methods?

Variable: change_online

Variable: change_app

	Very likely	Likely	Not sure	Unlikely	Very unlikely	Prefer not to say
**Online (via a website)** * must provide value	○	○	○	○	○	○
**By downloading an app** * must provide value	○	○	○	○	○	○

reset

For peer review only - http://bmjopen.bmj.com/site/about/guidelines.xhtml

Variable: change_paper

On paper forms available from pharmacies, Post Office, etc. (and sent by post)
* must provide value

reset

Variable: change_in_person

In person (e.g. when attending your GP, at a hospital clinic, etc.)
* must provide value

reset

Add Field | Add Matrix of Fields

Matrix group: likelihood_signup

Assume that you were able to sign up and change your choices in a way you preferred, and that the consent form was stored securely in a place where you felt comfortable.

How likely would you be to use this system to choose how your NHS health data is managed?

Variable: likelihood_signup

	Very likely	Likely	Not sure	Unlikely	Very unlikely	Prefer not to say
**Overall likelihood that you would sign up** * must provide value	○	○	○	○	○	○

reset

Add Field | Add Matrix of Fields

The last part of the survey (before we ask a few questions to ensure we have reached a broad section of the UK) examines what a consent form to share our health data could look like.

By completing one consent form it would then be possible for all UK NHS professionals to access their patient's identifiable health data for their clinical care, saving time for both patients and health care professionals.

Please take a look at the consent form below:

Add Field | Add Matrix of Fields

Variable: consent_form_descript

National NHS/HSC data sharing consent form

Full name ______________________
Date of birth ___________
NHS number ______________________
Address ________________________________
____
E-mail address ______________________

I confirm I have read the information sheet 'Sharing My Health Data' version XXX dated XXX. I have had the opportunity to consider the information and ask questions. I understand that my participation is voluntary, and that I am free to change or withdraw my consent at any time, without giving a reason and without my medical care or rights being affected. Yes [ ]

1. Providing health care to you

Confidential patient information is information that can identify you and that says something about your health care or treatment. Information about you might be held by several NHS organizations (such as GP surgeries and hospitals). May they share your information with each other when providing health care to you?

I agree that all NHS care providers and professionals may share my confidential patient information with each other for the purpose of my treatment and care. Yes [ ] No [ ]

2. Using your de-identified data to help others

The NHS promises to **anonymise** the information collected during the course of your treatment and use it to support research and improve care for others. Research is conducted by the NHS and by NHS-approved researchers in organizations such as universities. Strict security controls apply to the use of NHS data, even after information that might identify you (such as names and addresses) has been removed. All research involving NHS data must be approved by the NHS.

Saying no doesn't prevent all uses of your confidential information for research. To do that, use the national data opt-out at https://www.nhs.uk/your-nhs-data-matters.

I agree that all NHS care providers may share my confidential patient information with each other and de-identify it for the purpose of research. Yes [ ] No [ ]

3. Taking part in research

Some research involves **direct participation.** This ranges from questionnaires to trials of new treatments. The NHS promises to inform you of research studies in which you may be eligible to participate. There is never a commitment to take part.

I agree that NHS-approved researchers may learn my identity and contact me directly about research studies for which I may be eligible. Yes [ ] No [ ]

Signature: ______________________

Date: ___________

Add Field | Add Matrix of Fields

Matrix group: consent_form

Having looked at the consent form, please agree or disagree with the following statements.

(If you would like to take another look at the consent form at any time while answering this question, you can use the 'previous' button at the bottom of this page to look back. This will not affect answers already given.)

Item	Strongly agree	Agree	Neither agree nor disagree	Disagree	Strongly disagree	Prefer not to say
Variable: consent_read<br>**The consent form is easy to read.**<br>* must provide value<br>reset						
Variable: consent_clear<br>**The consent form is clear and easy to understand.**<br>* must provide value<br>reset						
Variable: consent_help<br>**The statements (in blue) help me to understand what I am consenting to.**<br>* must provide value<br>reset						
Variable: consent_professionals<br>**The consent form is clear that my identifiable health data would only be used by appropriate NHS health care professionals.**<br>* must provide value<br>reset						
Variable: consent_researchers<br>**I understand the term 'NHS-approved researchers' and which individuals this may refer to.**<br>* must provide value<br>reset						
Variable: consent_identifiable_care<br>**The consent form makes clear that it would be used for my preferences about sharing my IDENTIFIABLE health data within the NHS for my CLINICAL CARE.**<br>* must provide value<br>reset						
Variable: consent_non_id_research<br>**The consent form makes clear that it would also be used for my preferences about sharing my DE-IDENTIFIED health data for RESEARCH purposes.**<br>* must provide value<br>reset						
Variable: consent_add_contact<br>**I would like the consent form to give specific options about how researchers can contact me (e.g. by post only, e-mail).**<br>* must provide value<br>reset						
Variable: consent_add_security<br>**I would like to see a statement added about the security of data sharing.**<br>* must provide value<br>reset						
Variable: consent_add_storage<br>**I would like to see a statement added about where my consent preferences will be stored.**<br>* must provide value<br>reset						

Variable: consent_opt_out

Before seeing this consent form I was aware of the NHS National Data Opt-Out.
* must provide value

reset

Variable: consent_nhs_secure

The consent form reassures me that my identifiable health data is safe in the NHS.
* must provide value

reset

Variable: consent_sign

If this consent form were put in front of me today, I would sign it.
* must provide value

reset

Add Field | Add Matrix of Fields

Variable: consent_comments

If you would like to make any comments about the consent form, please do so here:

Expand

Add Field | Add Matrix of Fields

There are currently many individual websites where people can sign up to take part in research. Examples include charity websites, the national "Join Dementia Research" register, and the National Institute for Health Research (NIHR) "be part of research" campaign.

Add Field | Add Matrix of Fields

Variable: national_sign_up

An alternative might be a national sign-up portal, on a web site, where people could register their preferences about taking part in all NHS research.

Choose the statement you agree with most:

* must provide value

- Everything is fine as it is; leave it to individuals to sign up with the various organisations.
- Have two national NHS research websites, one where people can sign up for mental health research, and a second website where people can sign up for physical health research.
- Have a national NHS research website where people can sign up for all conditions.
- Not sure.

reset

Add Field | Add Matrix of Fields

Matrix group: linked_data

Finally, it can be very valuable for research to link health (NHS) data to other data sources.

For example, causes of death (from death certificates) are held by the UK Office of National Statistics (ONS), rather than the NHS -- so to find out more about the reasons people die, NHS and ONS data must be linked.

Usually, this is done as follows:
a) Research teams seek special permission for the process.
b) The relevant information from each organization, plus a small amount of identifiable information, is given to a "trusted third party" (e.g. an NHS Trust, the Office of National Statistics).
c) The trusted third party links the information, then removes any details that can identify anyone.
d) Researchers are then given access to the de-identified information only, under special controls.

We are interested to hear whether you would be happy for your health data to be linked, in this way, to:

Variable	Item	Yes	Not sure	No	Prefer not to say
Variable: link_education	**Educational data (e.g. to study impact of illness on education)** * must provide value	○	○	○	○
Variable: link_police	**Police/Criminal Justice data (e.g. to study health in the victims of crime)** * must provide value	○	○	○	○
Variable: link_transport	**Transport/DVLA data (e.g. to study health and pollution)** * must provide value	○	○	○	○
Variable: link_housing	**Housing data (e.g. impact of social housing on health)** * must provide value	○	○	○	○
Variable: link_immigration	**Immigration data (e.g. health in immigrants)** * must provide value	○	○	○	○
Variable: link_dwp	**Social security/Work and Pensions (e.g. health and financial insecurity)** * must provide value	○	○	○	○
Variable: link_universities	**Data held about you for research by universities (e.g. if you have volunteered for research studies)** * must provide value	○	○	○	○
Variable: link_private	**Data held about you by private companies** * must provide value	○	○	○	○

reset

Add Field | Add Matrix of Fields

And finally, to ensure we have surveyed a wide range of the population (please note, all questions have a "prefer not to say" option and have been written to apply to all of the home nations):

Add Field | Add Matrix of Fields

Variable: demo_gender

Do you consider yourself to be:

* must provide value

- Prefer not to say
- Female
- Male
- Prefer to self describe

reset

Add Field | Add Matrix of Fields

Variable: demo_gender_self *Branching logic:* [demo_gender] = '4'

Prefer to self describe (please specify)

Add Field | Add Matrix of Fields

Variable: demo_age

May we know which age range you fit in?

* must provide value

- Prefer not to say
- Under 12
- 12-15
- 16-17
- 18-24
- 25-34
- 35-44
- 45-54
- 55-64
- 65-74
- 75-84
- 85+

reset

Add Field | Add Matrix of Fields

Variable: under_16_help *Branching logic:* [demo_age] = '2' or [demo_age] = '3'

Please tick which of the two options applies:

* must provide value

- I completed the survey by myself
- I had help to complete the survey

reset

Add Field | Add Matrix of Fields

Variable: demo_ethnicity

What do you consider your ethnicity to be?

* must provide value

- Prefer not to say
- White
- Irish Traveller
- Mixed/Multiple ethnic groups
- Asian or Asian British
- Indian
- Pakistani
- Bangladeshi
- Chinese
- Black/African/Caribbean/Black British
- Arab
- Other or prefer to self describe

reset

Add Field | Add Matrix of Fields

Variable: demo_ethnicity_other *Branching logic:* [demo_ethnicity] = '12'

Other or prefer to self describe (please specify)

Add Field | Add Matrix of Fields

Variable: demo_education

What is the highest qualification you have achieved?

* must provide value

- Prefer not to say
- No formal qualifications
- Secondary school level qualifications e.g. CSE, GCSE, O-Levels, Nationals, or equivalent
- A-Levels, Highers, or equivalent
- Vocational qualification or equivalent
- Undergraduate degree or equivalent
- Postgraduate or professional qualification or equivalent

reset

Add Field | Add Matrix of Fields

Variable: demo_sexuality

Do you consider yourself to be

* must provide value

- Prefer not to say
- Heterosexual (straight)
- Homosexual (gay or lesbian)
- Bisexual
- Other or prefer to self describe

reset

Add Field Add Matrix of Fields

Variable: demo_sexuality_other *Branching logic:* [demo_sexuality] = '5'

Other or prefer to self describe (Please specify)

Add Field Add Matrix of Fields

Variable: demo_religion

What is your religion, if any?

* must provide value

- Prefer not to say
- No religion
- Christian
- Muslim
- Hindu
- Sikh
- Jewish
- Buddhist
- Other or prefer to self describe

reset

Add Field Add Matrix of Fields

Variable: demo_religion_other *Branching logic:* [demo_religion] = '9'

Other or prefer to self describe (please specify)

Add Field Add Matrix of Fields

Variable: demo_se_1

Thinking about your current (or last) main job or occupation. Do (did) you work as an employee or are (were) you self-employed?

* must provide value

- Prefer not to say
- Never worked
- Employee
- Self-employed with employees
- Self-employed/freelance without employees

reset

Add Field Add Matrix of Fields

Variable: demo_se_2 *Branching logic:* [demo_se_1] = '3'

How many people work (worked) for your employer at the place where you work (worked)?

- Prefer not to say
- 1-24
- 25 or more

reset

Add Field Add Matrix of Fields

Variable: demo_se_3 *Branching logic:* [demo_se_1] = '4'

How many people do (did) you employ?

- Prefer not to say
- 1-24
- 25 or more

reset

Add Field Add Matrix of Fields

Variable: demo_se_4 *Branching logic:* ([demo_se_1] = '3' and ([demo_se_2] = '2' or [demo_se_2] = '3'...

Do (did) you supervise any other employees on a day to day basis?

- Prefer not to say
- Yes
- No

reset

Add Field Add Matrix of Fields

Variable: demo_se_5 *Branching logic:* ([demo_se_1] = '3' and ([demo_se_2] = '2' or [demo_se_2] = '3'...

And which best describes the work you do (did)?

- Prefer not to say
- Modern professional occupations such as: teacher - nurse - physiotherapist - social worker - welfare officer - artist - musician - police office (sergeant or above) - software designer
- Clerical and intermediate occupations such as: secretary - personal assistant - clerical worker - office clerk - call centre agent - nursing auxiliary - nursery nurse
- Senior managers or administrators (responsible for planning, organising, and co-coordinating work and for finance) such as: finance manager - chief executive
- Technical and craft occupations such as: motor mechanic - fitter - inspector - plumber - printer - tool maker - electrician - gardener - train driver
- Semi-routine manual and service occupations such as: postal worker - machine operative - security guard - caretaker - farm worker - catering assistant - receptionist - sales assistant
- Routine manual and service occupations such as: HGV driver - van driver - cleaner - porter - packer - sewing machinist - messenger - labourer - waiter/waitress - bar staff
- Middle or junior managers such as: office manager - retail manager - bank manager - restaurant manager - warehouse manager - publican
- Traditional professional occupations such as: accountant - solicitor - medical practitioner - scientist - civil/mechanical engineer

reset

Add Field Add Matrix of Fields

Variable: demo_country

And where in the UK do you currently live?

* must provide value

- Prefer not to say
- England
- Northern Ireland
- Scotland
- Wales
- Channel Islands
- Isle of Man
- None of these

reset

Add Field | Add Matrix of Fields

Variable: demo_eng_region *Branching logic:* [demo_country] = '2'

Please specify the region you live in

* must provide value

- Prefer not to say
- North East
- North West
- Yorkshire and Humber
- West Midlands
- East Midlands
- East of England
- London
- South East
- South West
- Other

reset

Add Field | Add Matrix of Fields

Variable: demo_sco_region *Branching logic:* [demo_country] = '4'

Please specify the region you live in

* must provide value

- Prefer not to say
- Central Scotland
- Glasgow
- Highlands and Islands
- Lothian
- Mid Scotland and Fife
- North East Scotland
- South Scotland
- West Scotland
- Other

reset

Add Field | Add Matrix of Fields

Variable: demo_wales_region *Branching logic:* [demo_country] = '5'

Please specify the region you live in

* must provide value

- Prefer not to say
- West Wales and the Valleys
- East Wales
- Other

reset

Add Field | Add Matrix of Fields

Variable: demo_ni_region *Branching logic:* [demo_country] = '3'

Please specify the region you live in

* must provide value

- Prefer not to say
- Antrim and Newtownabbey
- Ards and North Down
- Armagh City, Banbridge and Craigavon
- Belfast
- Causeway Coast and Glens
- Derry City and Strabane
- Fermanagh and Omagh
- Lisburn and Castlereagh
- Mid and East Antrim
- Mid Ulster
- Newry Mourne and Down
- Other

reset

Add Field | Add Matrix of Fields

Variable: demo_sco_region_other *Branching logic:* [demo_sco_region] = '10'

Other (Please specify)

Add Field | Add Matrix of Fields

Variable: demo_eng_regio_other *Branching logic:* [demo_eng_region] = '11'

Other (please specify)

Add Field | Add Matrix of Fields

Variable: demo_wales_region_other *Branching logic:* [demo_wales_region] = '4'

Other (please specify)

Add Field | Add Matrix of Fields

Variable: demo_ni_region_other *Branching logic:* [demo_ni_region] = '13'

Other (please specify)

Add Field | Add Matrix of Fields

Variable: demo_postcode

We would like to know your postcode so that we can analyse our results by "geography", such as urban versus rural areas. You do not have to give this. However if you do, we will never disclose it or identify you from it.

* must provide value

- I prefer not to give my postcode
- I am happy to give my postcode

reset

For peer review only - http://bmjopen.bmj.com/site/about/guidelines.xhtml

Add Field | Add Matrix of Fields

Variable: demo_postcode_actual *Branching logic:* [demo_postcode] = '2'

Postcode

Add Field | Add Matrix of Fields

Variable: email

Before you submit your answers there is the option to leave an e-mail address should you wish to have the final report of the survey results personally e-mailed to you.

Please note: all e-mail addresses will be removed from the survey answers so that you cannot be identified, and will be held securely on password-protected computers at the University of Cambridge until the results are available, as described in the information sheet at the start of the survey.

If you wish to leave an e-mail address, please do so in the box below (and then submit your answers). Otherwise, please submit your answers now.

Optional

Add Field | Add Matrix of Fields

Resize font:

Returning?

NHS Data Consent Survey

What am I being invited to do?

You are being invited to take part in an online survey about the UK's views on sharing National Health Service (NHS) (or Health and Social Care in Northern Ireland) health data, for both clinical and research purposes. This survey is open to anyone living in the UK over 16 years of age (or under 16 with parental permission). If you are under 16, please show your parent/guardian this page and ask if they are happy for you to take this survey. You will need to confirm you have their permission before you start the survey.

What will taking part involve?

If you agree to take part, after filling in a consent form, you will be asked a series of questions about your views on sharing your NHS/HSC health data for both clinical and research purposes. By 'health data' we mean any information collected about a person's health including things like medical notes, blood test results, prescription information etc. collected by an appropriate health care professional. As well as asking about sharing health data, we will also ask you some questions about your physical and mental health and any health services you may have used, as well as some questions about you, for example your age and gender. The survey does not ask for any identifiable personal information, and at any time you may click the "prefer not to answer" box if you wish.

The survey has been tested and will take approximately 18-25 minutes to complete. The survey does not need to be completed at one sitting.

Your answers won't affect the way your own health information is managed.

What are the benefits or disadvantages of taking part in the survey?

There are no direct benefits or disadvantages to taking part in this survey nor are there any payments. However, the study team hopes the results of this survey can be used to inform and improve the way the NHS/HSC manage data for clinical and research purposes.

Do I have to take part?

No, taking part in this survey is entirely up to you. The consent form at the start of the survey asks for your permission to use your data, even if you decide not to complete all of the survey. Unfortunately, once data has been submitted it will not be possible for the study team to find and delete your answers as we are not asking for any personal details, making it impossible for us to retrieve and delete your individual answers.

How will the information I give be kept confidential? Where can I find out more about how your information is used?

No information that can identify you is being collected in this study. There is an option to leave an email address, should you wish to personally receive the results of the survey. Email addresses will be removed from the survey responses and stored on password protected computers at the University of Cambridge. Access to these email addresses will be by the study team only and will only be used to send you the final results of the survey. Once you have received the results you will be asked if you would like any further updates, for example any publications or posters that arise from the results of the study. If you decide you do not want any further information, we will delete your email address. If you would like further updates, we will keep your email address securely until we have sent you the final publications from the study, at which time point your email address will then be deleted from our records.

There is also an option to leave your postcode (or not) in the survey. The study team would like to look at whether there are any effects of geographical area on answers to the survey. Once the survey has closed the study team will convert all postcodes to a nationally available 'code' of the area you live in, and then delete your postcode. By doing this it will never be possible to identify you or the road that you live in, as one code is given to multiple postcodes in the same area. Whether or not you agree to give us your post code is entirely up to you.

Once the study team have finished using the de-identified data, they will make it freely available for public use via the University of Cambridge Research Data Repository (https://www.data.cam.ac.uk/repository). Please be assured that, as in the information above, any email addresses or postcodes given will have been deleted before this happens. There will be no way that anyone will be able to identify you from the data, but we hope that others may use the data to further understand the sharing of health data with the aim to improve mental and physical health.

You can find out more about how we use your information:

- at www.hra.nhs.uk/information-about-patients
- our leaflet from http://www.hra.nhs.uk/patientdataandresearch
- by sending an email to the University of Cambridge Data Protection officer at dpo@admin.cam.ac.uk
- by asking one of the research team

Who is conducting this survey?

This survey is being conducted by the CLIMB project study team based at the University of Cambridge in conjunction with a patient and public research advisory group. Funding for the study team is from the Medical Research Council's (MRC) Mental Health Data Pathfinder awards.

For peer review only - http://bmjopen.bmj.com/site/about/guidelines.xhtml

Who has reviewed this study?

This study has been reviewed and given a favourable opinion by members of the East of Scotland Research Ethics Service.

Where can I find the results of this survey?

Once the study has concluded and the results have been analysed, a report will be made available. If you have chosen to leave an email address this report will be sent directly to you. Results will also be published on the following websites: https://www.climbproject.org.uk/ and www.psychiatry.cam.ac.uk. You may like to note these down now before you move on or, alternatively, they will be displayed again at the end of the survey.

What if I have any concerns about this study?

The study team can be contacted in the following ways:

Mrs Linda Jones

Email: laj28@cam.ac.uk

Telephone (01223) 764670

Further contact details are available at: https://www.climbproject.org.uk/

Alternatively you may like to contact the CPFT's Patient Advice and Liaison Service (PALS) on 0800 376 0775 or email pals@cpft.nhs.uk. Please note PALS will not be able to give advice about technical queries with the study.

Thank you very much for reading the information sheet about the survey. If you would now like to take part in the survey, please read the following statements and tick the boxes to show that you agree. Please only take the survey one time (although you do not have to complete it in one go).

Page 1 of 29

Next Page >>

Save & Return Later

For peer review only - http://bmjopen.bmj.com/site/about/guidelines.xhtml

NHS Data Consent Survey

Resize font:

Page 2 of 29

Consent Form

Please tick ALL boxes

**I confirm I am aged 16 or over, or I am under 16 years of age but have the consent of my parent/guardian.** * must provide value	☑
**I confirm that I am a resident of the UK.** * must provide value	☑
**I understand I may quit the survey at any time but any data I have provided may still be used.** * must provide value	☑
**I understand that the answers I give will be used for analysis and publication.** * must provide value	☑
**I understand my answers will be recorded in a way that means I cannot be identified.** * must provide value	☑
**I understand that not everyone will see the same questions or definitions.** * must provide value	☑
**If I have any problems or questions with the survey, I understand I can contact Linda Jones, e-mail: laj28@cam.ac.uk, telephone: 01223 764670** * must provide value	☑
**If I choose to leave an email address, I understand it will be removed from the survey and only used to send me the results of the survey** * must provide value	☑
**I consent to take part in this survey.** * must provide value	☑

We would like to know where you heard about this survey. It is especially important to know if you were given a leaflet (or letter) or saw a poster/text/social media from a 'healthcare provider' e.g. hospital, clinic, GP, ambulance service, healthcare community setting, or any other healthcare provider.

* must provide value

- ○ Prefer not to say
- ○ GP (via a poster, leaflet, from a staff member, via text, via GP Practice social media etc.)
- ◉ Hospital/Clinic/Ambulance/Healthcare community setting, other healthcare provider (via a poster, leaflet, from a staff member, text, healthcare provider's social media etc.)
- ○ Group to which I belong
- ○ Charity website/email/newsletter etc.
- ○ Social media (other than from a healthcare provider)
- ○ Poster (other than at a healthcare provider)
- ○ Browsing the internet
- ○ Friend
- ○ Not sure
- ○ Other

reset

Please could you tell us in which healthcare establishment you saw a poster or picked up/were given a leaflet? If you have the leaflet, the healthcare setting may be named on the back of it. This is really important to each healthcare provider. (Please type in a few letters and options will appear.)

* must provide value

Cambridgeshire and Peterborough NHS Foundation Trust (CPFT)

<< Previous Page

Next Page >>

Save & Return Later

For peer review only

NHS Data Consent Survey

Resize font:

Page 3 of 29

Before starting the survey please read the following definitions:

By "NHS" we mean all parts of the national health service in the UK: NHS England, NHS Scotland, NHS Wales, and Health and Social Care in Northern Ireland (HSC). We refer to all these as the NHS in this survey.

Health data means any information about a person's health such as might be collected by a nurse, doctor or other appropriate member of NHS staff. Health data includes things like your medical notes, blood test results, prescription information, scans, etc.

Identifiable health data means any health data (as described above) that can also identify a person -- such as any health data that also contains names, addresses, NHS numbers, etc.

Clinical care means providing health care directly to people -- for example, diagnosing illnesses or treating ill health.

We would like to know/gauge your understanding of how NHS clinical care providers currently share your identifiable health data for your clinical care, without asking you each time. If you are not sure, please have a "best guess" as to what you think might happen.

My understanding is that (please tick only one):

* must provide value

- My identifiable health data is currently shared freely between all NHS sites across the UK -- i.e. all acute hospital Trusts, my GP, all mental health Trusts, ambulance services, etc., in the whole UK.
- My identifiable health data is currently shared between all NHS sites but only in the home nation that I live in -- i.e. shared only in England, or Scotland, or Northern Ireland, or Wales, but not between them.
- My identifiable health data is currently only shared between NHS sites in my region (cluster of hospitals nearest to my local hospital).
- My identifiable health data is currently only shared between my GP, acute local hospital, local mental health Trust, and other local services.
- My identifiable health data is currently not shared between any NHS sites.

reset

<< Previous Page

Next Page >>

Save & Return Later

For peer review only - http://bmjopen.bmj.com/site/about/guidelines.xhtml

NHS Data Consent Survey

Resize font:

Page 4 of 29

MOST hospital Trusts, GPs, mental health Trusts are separate and currently DO NOT share identifiable health data without asking you, though they correspond and communicate with each other with your permission.

In view of the statement above, we would now like to ask you your views on sharing your own identifiable health data for YOUR CLINICAL CARE, without having to be asked each time (please tick only one):

* must provide value

- I would like my identifiable health data to be shared for clinical purposes with any part of the NHS in the UK, without asking me first.
- I would like my identifiable health data to be shared for clinical purposes with any part of the NHS in my home nation only (i.e. only in England, or only in Northern Ireland, or only in Scotland, or only in Wales) without asking me first.
- I would like my identifiable health data to be shared for clinical purposes with any part of my region (cluster of hospitals nearest to my local hospital), without asking me first.
- I would like my identifiable health data to be shared for clinical purposes only with my GP, local hospital, and local mental health services, without asking me first.
- No one in the NHS should share my identifiable health data without asking me first.
- Not sure.

reset

<< Previous Page

Next Page >>

Save & Return Later

For peer review only - http://bmjopen.bmj.com/site/about/guidelines.xhtml

NHS Data Consent Survey

Resize font:

Page 5 of 29

We would now like to ask you some questions about your own MENTAL and PHYSICAL health. You can "prefer not to say" if you wish.

We would like to ask if you have had a MENTAL health condition either recently or at some point in your life.

By mental health conditions we include things like delirium, self harm, substance abuse, dementia, mania, schizophrenia, depression, anxiety disorders, psychosis, eating disorders, OCD, personality disorders, etc.

* must provide value

- I prefer not to say.
- Yes, I have had a mental health condition either recently or at some point in my life.
- No, I have never had a mental health condition.

reset

<< Previous Page

Next Page >>

Save & Return Later

For peer review only - http://bmjopen.bmj.com/site/about/guidelines.xhtml

NHS Data Consent Survey

Resize font:

Page 6 of 29

We would like to know if you would be willing to share what mental health condition you have had.

You may tick as many boxes as you wish.

If you prefer not to say, please use the "next page" button below to move on.

	In the last 5 years	More than 5 years ago
Delirium	☐	☐
Alcohol or substance abuse	☐	☐
Schizophrenia, schizoaffective disorder, or delusional disorder	☐	☐
Dementia	☐	☐
Mania or bipolar affective disorder	☐	☐
Depression	☐	☐
Anxiety disorder (e.g. phobia, panic, generalized anxiety disorder, post-traumatic stress disorder)	☐	☐
Psychosis of any cause	☐	☐
Eating disorder (e.g. anorexia nervosa, bulimia)	☐	☐
Obsessive-compulsive disorder	☐	☐
Personality disorder	☐	☐
Self harm	☐	☐
Other	☐	☐

<< Previous Page

Next Page >>

Save & Return Later

For peer review only - http://bmjopen.bmj.com/site/about/guidelines.xhtml

NHS Data Consent Survey

Resize font:

Page 7 of 29

And also in relation to mental health:

We would also like to know if you have accessed any mental health services for your own mental health condition.

These might include your GP, online services, mental health forums, psychological therapy services, crisis care, home treatments or visits, outpatient or inpatient care, services from the private sector, support from a mental health charity, etc.

* must provide value

- ○ I prefer not to say.
- ● I have used mental health services.
- ○ I have not used any mental health services (not even my GP or surgery) for my mental health condition.

reset

	In the last 5 years	More than 5 years ago
Mental health support from your GP or surgery	☐	☐
An NHS psychological therapy service, e.g. IAPT or other therapy service (INDIVIDUAL session)	☐	☐
An NHS psychological therapy service, e.g. IAPT or other therapy service (GROUP session)	☐	☐
An NHS psychological therapy service, e.g. IAPT or other therapy service (ONLINE)	☐	☐
NHS outpatient or community mental health services, or key worker	☐	☐
NHS crisis care/Home Treatment teams	☐	☐
NHS inpatient mental health services in a mental health hospital	☐	☐
NHS inpatient mental health services in a general hospital (liaison psychiatry)	☐	☐
Private services	☐	☐
Services in the workplace e.g. Occupational Health	☐	☐
Charity providing mental health support	☐	☐
Online forum	☐	☐
Other	☐	☐

<< Previous Page

Next Page >>

Save & Return Later

For peer review only - http://bmjopen.bmj.com/site/about/guidelines.xhtml

NHS Data Consent Survey

Resize font:

Page 8 of 29

We are also interested to know if you have accessed any services regarding your own PHYSICAL health.

This could be services like your GP, outpatient hospital appointments, inpatient services, online support, Accident and Emergency, 111 service, private services, charities providing physical health support, etc.

Have you ever used any of the following services for your own PHYSICAL health?

* must provide value

- ○ I prefer not to say
- ◉ I have used physical health services (this includes using your GP)
- ○ I have not used any physical health services

reset

<< Previous Page

Next Page >>

Save & Return Later

For peer review only - http://bmjopen.bmj.com/site/about/guidelines.xhtml

NHS Data Consent Survey

Resize font:

Page 9 of 29

And finally in this section, we would like to know if you would be willing to share what physical health services you have used for your own physical health.

You may tick as many boxes as you wish.

Please use the 'next page' button if you prefer not to answer.

	In the last 5 years	More than 5 years ago
Physical health support from your GP or surgery	☐	☐
NHS hospital outpatient services (outpatient clinics etc.)	☐	☐
NHS Urgent Care Centre (not A&E) or NHS 111 helpline	☐	☐
NHS Accident and Emergency services (A&E)	☐	☐
NHS inpatient services (procedures not completed in 1 day)	☐	☐
NHS Day Surgery/day care in a hospital	☐	☐
Private services	☐	☐
Services in the workplace e.g. Occupational Health	☐	☐
Charity providing physical health support	☐	☐
Online forum	☐	☐
Other	☐	☐

<< Previous Page

Next Page >>

Save & Return Later

For peer review only - http://bmjopen.bmj.com/site/about/guidelines.xhtml

NHS Data Consent Survey

Resize font:

Page 10 of 29

Moving on...

The table below shows what NHS identifiable health data may look like. This sort of data is used for health care by NHS staff. (Please note the examples shown are not real.)

NHS number	Title	Forename	Surname	Date of birth	Address	Diagnoses	Notes
9876543210	Mr	John	Smith	20 Jan 1950	1 The Street, Maidstone	pneumonia<br>high blood pressure	1 Apr 2019. Seen in clinic. Mr Smith has a fever and a cough. His chest sounds crackly. I think he has pneumonia. Sent to hospital.
8765432109	Miss	Alice	Jones	16 Jun 1994	15 The Road, Dundee	depression<br>carpal tunnel syndrome	2 Apr 2019. Alice rates her mood as 1/10 on average, she is more tired, and she is not looking forward to anything. Her depression has worsened. She wishes to restart citalopram.
7654321098	Mrs	Chloe	Williams	4 May 1930	5 Tree Close, Cardiff	broken humerus<br>anxiety	3 Apr 2019. Chloe fell after skidding on an oily patch while cycling and was hit by a car. Has an obvious fracture of her left upper arm. Needs X-ray.
6543210987	Mr	Pradeep	Agarwal	22 Sep 1973	27 The Mews, London	schizophrenia<br>diabetes	4 Apr 2019. Pradeep still worries that other people can hear his thoughts, but this is getting less common. He is attending cognitive–behavioural therapy sessions.

<< Previous Page

Next Page >>

Save & Return Later

For peer review only - http://bmjopen.bmj.com/site/about/guidelines.xhtml

NHS Data Consent Survey

Resize font:

Page 11 of 29

Previous surveys have found that people have more concerns about the use of their identifiable health data relating to their mental health than other aspects of their physical health care.

Regardless of whether you have a PHYSICAL health condition or have used PHYSICAL health services:

How likely are you to agree to share your identifiable PHYSICAL health data for your clinical care with the following, without being asked every time?

	Very likely	Likely	Not sure	Unlikely	Very unlikely	Prefer not to say
**Between my local NHS services -- i.e. between my local physical and mental health hospitals, my GP, etc.** * must provide value	○	○	○	○	○	○ reset
**Any part of the NHS in my region (cluster of hospitals nearest to my local hospital)** * must provide value	○	○	○	○	○	○ reset
**Any part of the NHS in my home nation only (i.e. only in England, or only in Northern Ireland, or only in Scotland, or only in Wales)** * must provide value	○	○	○	○	○	○ reset
**Any part of the NHS in the UK** * must provide value	○	○	○	○	○	○ reset

<< Previous Page

Next Page >>

Save & Return Later

For peer review only - http://bmjopen.bmj.com/site/about/guidelines.xhtml

NHS Data Consent Survey

Resize font:

Page 14 of 29

Regardless of whether you have a MENTAL health condition or have used MENTAL health services:

How likely are you to agree to share your identifiable MENTAL health data for your clinical care with the following, without being asked every time?

	Very likely	Likely	Not sure	Unlikely	Very unlikely	Prefer not to say
**Between my local NHS services -- i.e. between my local physical and mental health hospitals, my GP, etc.** * must provide value	○	○	○	○	○	○ reset
**Any part of the NHS in my region (cluster of hospitals nearest to my local hospital)** * must provide value	○	○	○	○	○	○ reset
**Any part of the NHS in my home nation only (i.e. only in England, or only in Northern Ireland, or only in Scotland, or only in Wales)** * must provide value	○	○	○	○	○	○ reset
**Any part of the NHS in the UK** * must provide value	○	○	○	○	○	○ reset

<< Previous Page

Next Page >>

Save & Return Later

For peer review only - http://bmjopen.bmj.com/site/about/guidelines.xhtml

NHS Data Consent Survey

Resize font:

Page 15 of 29

In the next two questions we would like to ask you about sharing your NHS data for research purposes. Research is used to improve overall healthcare for everyone.

Firstly, please look at some of the NHS health care data that you saw previously, which has now been de-identified.

Research ID	Age	Sex	Region	Diagnoses
5X62V	69	M	Kent	pneumonia<br>high blood pressure
597PT	24	F	Dundee	depression<br>carpal tunnel syndrome
8HG7S	89	F	Glamorganshire	broken humerus<br>anxiety
BA6A9	45	M	London	schizophrenia<br>diabetes

<< Previous Page

Next Page >>

Save & Return Later

For peer review only - http://bmjopen.bmj.com/site/about/guidelines.xhtml

NHS Data Consent Survey

Resize font:

Page 16 of 29

How likely would you be to share your de-identified PHYSICAL health data with the following, without giving consent every time?

	Very likely	Likely	Not sure	Unlikely	Very unlikely	Prefer not to say
**Any part of the NHS doing research** * must provide value	○	○	○	○	○	○ reset
**Academic institutions doing research (e.g. universities)** * must provide value	○	○	○	○	○	○ reset
**A national charity doing research (e.g. MIND, Cancer Research UK, British Heart Foundation)** * must provide value	○	○	○	○	○	○ reset
**A profit-making company doing research into treatments (e.g. a pharmaceutical company, health technology company)** * must provide value	○	○	○	○	○	○ reset
**A profit-making company doing other research (e.g. an insurance company, broadband provider)** * must provide value	○	○	○	○	○	○ reset
**Publicly** * must provide value	○	○	○	○	○	○ reset

<< Previous Page

Next Page >>

Save & Return Later

For peer review only - http://bmjopen.bmj.com/site/about/guidelines.xhtml

NHS Data Consent Survey

Resize font:

Page 17 of 29

How likely would you be to share your de-identified MENTAL health data with the following, without giving consent every time:

	Very likely	Likely	Not sure	Unlikely	Very unlikely	Prefer not to say
**Any part of the NHS doing research** * must provide value	○	○	○	○	○	○ reset
**Academic institutions doing research (e.g. universities)** * must provide value	○	○	○	○	○	○ reset
**A national charity doing research (e.g. MIND, Cancer Research UK, British Heart Foundation)** * must provide value	○	○	○	○	○	○ reset
**A profit-making company doing research into treatments (e.g. pharmaceutical company, health technology company)** * must provide value	○	○	○	○	○	○ reset
**A profit-making company doing other research (e.g. insurance company, broadband provider)** * must provide value	○	○	○	○	○	○ reset
**Publicly** * must provide value	○	○	○	○	○	○ reset

<< Previous Page

Next Page >>

Save & Return Later

For peer review only - http://bmjopen.bmj.com/site/about/guidelines.xhtml

NHS Data Consent Survey

Resize font:

Page 18 of 29

The final question in this section relates to sharing de-identified "free text" data for research.

Here is the table of de-identified health care data you saw earlier, but this time a column labelled "Notes" has been added. This is known as "free text" data.

Research ID	Age	Sex	Region	Diagnoses	Notes
5X62V	69	M	Kent	pneumonia<br>high blood pressure	1st April 2019. XXX has a fever and cough. His chest sounds crackly. I think he has pneumonia. Sent to hospital.
597PT	24	F	Dundee	depression<br>carpal tunnel syndrome	2nd April 2019. XXX rates her mood as 1/10 on average, she is more tired, and she is not looking forward to anything. Her depression has worsened. She wishes to restart citalopram.
8HG7S	89	F	Glamorganshire	broken humerus<br>anxiety	3rd April 2019. XXX fell after skidding on an oily patch while cycling and was hit by a car. She has an obvious fracture of her left upper arm. She needs an X-ray.
BA6A9	45	M	London	schizophrenia<br>diabetes	4th April 2019. XXX still worries that other people can hear his thoughts, but this is getting less common. He is attending cognitive-behavioural therapy sessions.

Like the last example the data remains de-identified but gives the researchers more information. As there is more information there is a slightly increased risk of someone being identified from the data.

For example, in the table above, an "89-year-old cyclist skidding on oil and being hit by a car" may be reported in a local newspaper. Whilst the lady is not named in the data above, she would almost certainly be named in the newspaper and it may therefore be possible for researchers to work out who she is.

<< Previous Page

Next Page >>

Save & Return Later

For peer review only - http://bmjopen.bmj.com/site/about/guidelines.xhtml

NHS Data Consent Survey

Resize font:

Page 19 of 29

How likely would you be to share your FREE TEXT de-identified PHYSICAL health data with the following, without giving consent every time:

	Very likely	Likely	Not sure	Unlikely	Very unlikely	Prefer not to say
**Any part of the NHS doing research** * must provide value	○	○	○	○	●	○
**Academic institutions doing research (e.g. universities)** * must provide value	○	○	○	○	●	○
**A national charity doing research (e.g MIND, Cancer Research UK, British Heart Foundation)** * must provide value	○	○	○	○	●	○
**A profit-making company doing research into treatments (e.g. pharmaceutical company, health technology company)** * must provide value	○	○	○	○	●	○
**A profit-making company doing other research (e.g. insurance company, broadband provider)** * must provide value	○	○	○	○	●	○
**Publicly** * must provide value	○	○	○	○	●	○

(Each row: reset)

<< Previous Page

Next Page >>

Save & Return Later

For peer review only - http://bmjopen.bmj.com/site/about/guidelines.xhtml

NHS Data Consent Survey

Resize font:

Page 20 of 29

How likely would you be to share your FREE TEXT de-identified MENTAL health data with the following, without giving consent each time:

	Very likely	Likely	Not sure	Unlikely	Very unlikely	Prefer not to say
**Any part of the NHS doing research** * must provide value	○	○	○	○	○	○ reset
**Academic institutions doing research (e.g. universities)** * must provide value	○	○	○	○	○	○ reset
**A national charity doing research (e.g MIND, Cancer Research UK, British Heart Foundation)** * must provide value	○	○	○	○	○	○ reset
**A profit-making company doing research into treatments (e.g. pharmaceutical company, health technology company)** * must provide value	○	○	○	○	○	○ reset
**A profit-making company doing other research (e.g. insurance company, broadband provider)** * must provide value	○	○	○	○	○	○ reset
**Publicly** * must provide value	○	○	○	○	○	○ reset

<< Previous Page

Next Page >>

Save & Return Later

For peer review only - http://bmjopen.bmj.com/site/about/guidelines.xhtml

NHS Data Consent Survey

Resize font:

Page 21 of 29

On a slightly different topic...

Imagine that there was a single place where you could securely sign up to choose how your NHS identifiable health data is used. This would cover how your confidential patient information is managed across all UK NHS services, and how your NHS de-identified data could (or could not) be used for research.

It would be in the form of a standardised UK-wide CONSENT FORM that all NHS services could use.

How likely would you be to sign up if you were asked to sign:

	Very likely	Likely	Not sure	Unlikely	Very unlikely	Prefer not to say
**Online (via a website)** * must provide value	○	○	○	○	○	○ reset
**By downloading an app** * must provide value	○	○	○	○	○	○ reset
**On paper forms available from pharmacies, the Post Office, etc. (and sent by post)** * must provide value	○	○	○	○	○	○ reset
**In person (e.g. when attending your GP, at a hospital clinic, etc.)** * must provide value	○	○	○	○	○	○ reset

<< Previous Page

Next Page >>

Save & Return Later

For peer review only - http://bmjopen.bmj.com/site/about/guidelines.xhtml

NHS Data Consent Survey

Resize font:

Page 22 of 29

Would you be likely to sign up if your consent form were then stored and managed securely by the following institutions?

	Very likely	Likely	Not sure	Unlikely	Very unlikely	Prefer not to say
**Centrally by NHS England/NHS Scotland/NHS Wales/HSC Northern Ireland** * must provide value	○	○	○	○	○	○
**Your local NHS Trust** * must provide value	○	○	○	○	○	○
**Your GP** * must provide value	○	○	○	○	○	○

reset

<< Previous Page

Next Page >>

Save & Return Later

For peer review only - http://bmjopen.bmj.com/site/about/guidelines.xhtml

NHS Data Consent Survey

Resize font:

Page 23 of 29

If you wanted to change your preferences, how likely would you be to use the following methods?

	Very likely	Likely	Not sure	Unlikely	Very unlikely	Prefer not to say
**Online (via a website)** * must provide value	○	○	○	○	○	○ reset
**By downloading an app** * must provide value	○	○	○	○	○	○ reset
**On paper forms available from pharmacies, Post Office, etc. (and sent by post)** * must provide value	○	○	○	○	○	○ reset
**In person (e.g. when attending your GP, at a hospital clinic, etc.)** * must provide value	○	○	○	○	○	○ reset

<< Previous Page

Next Page >>

Save & Return Later

For peer review only - http://bmjopen.bmj.com/site/about/guidelines.xhtml

Resize font:

NHS Data Consent Survey

Assume that you were able to sign up and change your choices in a way you preferred, and that the consent form was stored securely in a place where you felt comfortable.

How likely would you be to use this system to choose how your NHS health data is managed?

	Very likely	Likely	Not sure	Unlikely	Very unlikely	Prefer not to say
**Overall likelihood that you would sign up** * must provide value	○	○	○	○	○	○

reset

<< Previous Page

Next Page >>

Save & Return Later

For peer review only - http://bmjopen.bmj.com/site/about/guidelines.xhtml

NHS Data Consent Survey

Resize font:

Page 25 of 29

The last part of the survey (before we ask a few questions to ensure we have reached a broad section of the UK) examines what a consent form to share our health data could look like.

By completing one consent form it would then be possible for all UK NHS professionals to access their patient's identifiable health data for their clinical care, saving time for both patients and health care professionals.

Please take a look at the consent form below:

National NHS/HSC data sharing consent form

Full name ______________________
Date of birth ___________
NHS number ______________________
Address ________________________________
____
E-mail address ______________________

I confirm I have read the information sheet 'Sharing My Health Data' version XXX dated XXX. I have had the opportunity to consider the information and ask questions. I understand that my participation is voluntary, and that I am free to change or withdraw my consent at any time, without giving a reason and without my medical care or rights being affected. Yes [ ]

1. Providing health care to you

Confidential patient information is information that can identify you and that says something about your health care or treatment. Information about you might be held by several NHS organizations (such as GP surgeries and hospitals). May they share your information with each other when providing health care to you?

I agree that all NHS care providers and professionals may share my confidential patient information with each other for the purpose of my treatment and care. Yes [ ] No [ ]

2. Using your de-identified data to help others

The NHS promises to **anonymise** the information collected during the course of your treatment and use it to support research and improve care for others. Research is conducted by the NHS and by NHS-approved researchers in organizations such as universities. Strict security controls apply to the use of NHS data, even after information that might identify you (such as names and addresses) has been removed. All research involving NHS data must be approved by the NHS.

Saying no doesn't prevent all uses of your confidential information for research. To do that, use the national data opt-out at https://www.nhs.uk/your-nhs-data-matters.

I agree that all NHS care providers may share my confidential patient information with each other and de-identify it for the purpose of research. Yes [ ] No [ ]

3. Taking part in research

Some research involves **direct participation.** This ranges from questionnaires to trials of new treatments. The NHS promises to inform you of research studies in which you may be eligible to participate. There is never a commitment to take part.

I agree that NHS-approved researchers may learn my identity and contact me directly about research studies for which I may be eligible. Yes [ ] No [ ]

Signature: ______________________

Date: ___________

<< Previous Page

Next Page >>

Save & Return Later

For peer review only

NHS Data Consent Survey

Resize font:

Page 26 of 29

Having looked at the consent form, please agree or disagree with the following statements.

(If you would like to take another look at the consent form at any time while answering this question, you can use the 'previous' button at the bottom of this page to look back. This will not affect answers already given.)

	Strongly agree	Agree	Neither agree nor disagree	Disagree	Strongly disagree	Prefer not to say
The consent form is easy to read. * must provide value	○	○	○	○	○	○ reset
The consent form is clear and easy to understand. * must provide value	○	○	○	○	○	○ reset
The statements (in blue) help me to understand what I am consenting to. * must provide value	○	○	○	○	○	○ reset
The consent form is clear that my identifiable health data would only be used by appropriate NHS health care professionals. * must provide value	○	○	○	○	○	○ reset
I understand the term 'NHS-approved researchers' and which individuals this may refer to. * must provide value	○	○	○	○	○	○ reset
The consent form makes clear that it would be used for my preferences about sharing my IDENTIFIABLE health data within the NHS for my CLINICAL CARE. * must provide value	○	○	○	○	○	○ reset
The consent form makes clear that it would also be used for my preferences about sharing my DE-IDENTIFIED health data for RESEARCH purposes. * must provide value	○	○	○	○	○	○ reset
I would like the consent form to give specific options about how researchers can contact me (e.g. by post only, e-mail). * must provide value	○	○	○	○	○	○ reset
I would like to see a statement added about the security of data sharing. * must provide value	○	○	○	○	○	○ reset
I would like to see a statement added about where my consent preferences will be stored. * must provide value	○	○	○	○	○	○ reset
Before seeing this consent form I was aware of the NHS National Data Opt-Out. * must provide value	○	○	○	○	○	○ reset
The consent form reassures me that my identifiable health data is safe in the NHS. * must provide value	○	○	○	○	○	○ reset
If this consent form were put in front of me today, I would sign it. * must provide value	○	○	○	○	○	○ reset

If you would like to make any comments about the consent form, please do so here:

Expand

<< Previous Page

Next Page >>

Save & Return Later

For peer review only

For peer review only - http://bmjopen.bmj.com/site/about/guidelines.xhtml

NHS Data Consent Survey

Resize font:

Page 27 of 29

There are currently many individual websites where people can sign up to take part in research. Examples include charity websites, the national "Join Dementia Research" register, and the National Institute for Health Research (NIHR) "be part of research" campaign.

An alternative might be a national sign-up portal, on a web site, where people could register their preferences about taking part in all NHS research.

Choose the statement you agree with most:

* must provide value

- Everything is fine as it is; leave it to individuals to sign up with the various organisations.
- Have two national NHS research websites, one where people can sign up for mental health research, and a second website where people can sign up for physical health research.
- Have a national NHS research website where people can sign up for all conditions.
- Not sure.

reset

<< Previous Page

Next Page >>

Save & Return Later

For peer review only - http://bmjopen.bmj.com/site/about/guidelines.xhtml

NHS Data Consent Survey

Resize font:

Page 28 of 29

Finally, it can be very valuable for research to link health (NHS) data to other data sources.

For example, causes of death (from death certificates) are held by the UK Office of National Statistics (ONS), rather than the NHS -- so to find out more about the reasons people die, NHS and ONS data must be linked.

Usually, this is done as follows:
a) Research teams seek special permission for the process.
b) The relevant information from each organization, plus a small amount of identifiable information, is given to a "trusted third party" (e.g. an NHS Trust, the Office of National Statistics).
c) The trusted third party links the information, then removes any details that can identify anyone.
d) Researchers are then given access to the de-identified information only, under special controls.

We are interested to hear whether you would be happy for your health data to be linked, in this way, to:

	Yes	Not sure	No	Prefer not to say
Educational data (e.g. to study impact of illness on education) * must provide value	○	○	○	○ reset
Police/Criminal Justice data (e.g. to study health in the victims of crime) * must provide value	○	○	○	○ reset
Transport/DVLA data (e.g. to study health and pollution) * must provide value	○	○	○	○ reset
Housing data (e.g. impact of social housing on health) * must provide value	○	○	○	○ reset
Immigration data (e.g. health in immigrants) * must provide value	○	○	○	○ reset
Social security/Work and Pensions (e.g. health and financial insecurity) * must provide value	○	○	○	○ reset
Data held about you for research by universities (e.g. if you have volunteered for research studies) * must provide value	○	○	○	○ reset
Data held about you by private companies * must provide value	○	○	○	○ reset

<< Previous Page

Next Page >>

Save & Return Later

For peer review only - http://bmjopen.bmj.com/site/about/guidelines.xhtml

NHS Data Consent Survey

Resize font:

Page 29 of 29

And finally, to ensure we have surveyed a wide range of the population (please note, all questions have a "prefer not to say" option and have been written to apply to all of the home nations):

Do you consider yourself to be:

* must provide value

- Prefer not to say
- Female
- Male
- Prefer to self describe

reset

May we know which age range you fit in?

* must provide value

- Prefer not to say
- Under 12
- 12-15
- 16-17
- 18-24
- 25-34
- 35-44
- 45-54
- 55-64
- 65-74
- 75-84
- 85+

reset

What do you consider your ethnicity to be?

* must provide value

- Prefer not to say
- White
- Irish Traveller
- Mixed/Multiple ethnic groups
- Asian or Asian British
- Indian
- Pakistani
- Bangladeshi
- Chinese
- Black/African/Caribbean/Black British
- Arab
- Other or prefer to self describe

reset

For peer review only - http://bmjopen.bmj.com/site/about/guidelines.xhtml

What is the highest qualification you have achieved?

* must provide value

- Prefer not to say
- No formal qualifications
- Secondary school level qualifications e.g. CSE, GCSE, O-Levels, Nationals, or equivalent
- A-Levels, Highers, or equivalent
- Vocational qualification or equivalent
- Undergraduate degree or equivalent
- Postgraduate or professional qualification or equivalent

reset

Do you consider yourself to be

* must provide value

- Prefer not to say
- Heterosexual (straight)
- Homosexual (gay or lesbian)
- Bisexual
- Other or prefer to self describe

reset

What is your religion, if any?

* must provide value

- Prefer not to say
- No religion
- Christian
- Muslim
- Hindu
- Sikh
- Jewish
- Buddhist
- Other or prefer to self describe

reset

Thinking about your current (or last) main job or occupation. Do (did) you work as an employee or are (were) you self-employed?

* must provide value

- Prefer not to say
- Never worked
- Employee
- Self-employed with employees
- Self-employed/freelance without employees

reset

And where in the UK do you currently live?

* must provide value

- Prefer not to say
- England
- Northern Ireland
- Scotland
- Wales
- Channel Islands
- Isle of Man
- None of these

reset

For peer review only - http://bmjopen.bmj.com/site/about/guidelines.xhtml

We would like to know your postcode so that we can analyse our results by "geography", such as urban versus rural areas. You do not have to give this. However if you do, we will never disclose it or identify you from it.

* must provide value

- ◯ I prefer not to give my postcode
- ◯ I am happy to give my postcode

reset

Before you submit your answers there is the option to leave an e-mail address should you wish to have the final report of the survey results personally e-mailed to you.

Please note: all e-mail addresses will be removed from the survey answers so that you cannot be identified, and will be held securely on password-protected computers at the University of Cambridge until the results are available, as described in the information sheet at the start of the survey.

If you wish to leave an e-mail address, please do so in the box below (and then submit your answers). Otherwise, please submit your answers now.

Optional

[ ]

<< Previous Page

Submit

Save & Return Later

r review only

For peer review only - http://bmjopen.bmj.com/site/about/guidelines.xhtml

NHS Data Consent Survey

Resize font:

Page 29 of 29

And finally, to ensure we have surveyed a wide range of the population (please note, all questions have a "prefer not to say" option and have been written to apply to all of the home nations):

Do you consider yourself to be:

* must provide value

- ( ) Prefer not to say
- ( ) Female
- ( ) Male
- (•) Prefer to self describe

reset

Prefer to self describe (please specify)

May we know which age range you fit in?

* must provide value

- ( ) Prefer not to say
- (•) Under 12
- ( ) 12-15
- ( ) 16-17
- ( ) 18-24
- ( ) 25-34
- ( ) 35-44
- ( ) 45-54
- ( ) 55-64
- ( ) 65-74
- ( ) 75-84
- ( ) 85+

reset

Please tick which of the two options applies:

* must provide value

- ( ) I completed the survey by myself
- ( ) I had help to complete the survey

reset

What do you consider your ethnicity to be?

* must provide value

- ( ) Prefer not to say
- ( ) White
- ( ) Irish Traveller
- ( ) Mixed/Multiple ethnic groups
- ( ) Asian or Asian British
- ( ) Indian
- ( ) Pakistani
- ( ) Bangladeshi
- ( ) Chinese
- ( ) Black/African/Caribbean/Black British
- ( ) Arab
- (•) Other or prefer to self describe

reset

Other or prefer to self describe (please specify)

What is the highest qualification you have achieved?

* must provide value

- Prefer not to say
- No formal qualifications
- Secondary school level qualifications e.g. CSE, GCSE, O-Levels, Nationals, or equivalent
- A-Levels, Highers, or equivalent
- Vocational qualification or equivalent
- Undergraduate degree or equivalent
- Postgraduate or professional qualification or equivalent

reset

Do you consider yourself to be

* must provide value

- Prefer not to say
- Heterosexual (straight)
- Homosexual (gay or lesbian)
- Bisexual
- Other or prefer to self describe

reset

Other or prefer to self describe (Please specify)

What is your religion, if any?

* must provide value

- Prefer not to say
- No religion
- Christian
- Muslim
- Hindu
- Sikh
- Jewish
- Buddhist
- Other or prefer to self describe

reset

Other or prefer to self describe (please specify)

Thinking about your current (or last) main job or occupation. Do (did) you work as an employee or are (were) you self-employed?

* must provide value

- Prefer not to say
- Never worked
- Employee
- Self-employed with employees
- Self-employed/freelance without employees

reset

How many people work (worked) for your employer at the place where you work (worked)?

- ( ) Prefer not to say
- ( ) 1-24
- (•) 25 or more

reset

Do (did) you supervise any other employees on a day to day basis?

- ( ) Prefer not to say
- (•) Yes
- ( ) No

reset

And which best describes the work you do (did)?

- ( ) Prefer not to say
- ( ) Modern professional occupations such as: teacher - nurse - physiotherapist - social worker - welfare officer - artist - musician - police office (sergeant or above) - software designer
- ( ) Clerical and intermediate occupations such as: secretary - personal assistant - clerical worker - office clerk - call centre agent - nursing auxiliary - nursery nurse
- ( ) Senior managers or administrators (responsible for planning, organising, and co-coordinating work and for finance) such as: finance manager - chief executive
- ( ) Technical and craft occupations such as: motor mechanic - fitter - inspector - plumber - printer - tool maker - electrician - gardener - train driver
- ( ) Semi-routine manual and service occupations such as: postal worker - machine operative - security guard - caretaker - farm worker - catering assistant - receptionist - sales assistant
- ( ) Routine manual and service occupations such as: HGV driver - van driver - cleaner - porter - packer - sewing machinist - messenger - labourer - waiter/waitress - bar staff
- ( ) Middle or junior managers such as: office manager - retail manager - bank manager - restaurant manager - warehouse manager - publican
- ( ) Traditional professional occupations such as: accountant - solicitor - medical practitioner - scientist - civil/mechanical engineer

reset

And where in the UK do you currently live?

* must provide value

- ( ) Prefer not to say
- (•) England
- ( ) Northern Ireland
- ( ) Scotland
- ( ) Wales
- ( ) Channel Islands
- ( ) Isle of Man
- ( ) None of these

reset

For peer review only - http://bmjopen.bmj.com/site/about/guidelines.xhtml

Please specify the region you live in

* must provide value

- ○ Prefer not to say
- ○ North East
- ○ North West
- ○ Yorkshire and Humber
- ○ West Midlands
- ○ East Midlands
- ○ East of England
- ○ London
- ○ South East
- ○ South West
- ◉ Other

reset

Other (please specify)

We would like to know your postcode so that we can analyse our results by "geography", such as urban versus rural areas. You do not have to give this. However if you do, we will never disclose it or identify you from it.

* must provide value

- ○ I prefer not to give my postcode
- ◉ I am happy to give my postcode

reset

Postcode

Before you submit your answers there is the option to leave an e-mail address should you wish to have the final report of the survey results personally e-mailed to you.

Please note: all e-mail addresses will be removed from the survey answers so that you cannot be identified, and will be held securely on password-protected computers at the University of Cambridge until the results are available, as described in the information sheet at the start of the survey.

If you wish to leave an e-mail address, please do so in the box below (and then submit your answers). Otherwise, please submit your answers now.

Optional

<< Previous Page

Submit

Save & Return Later

For peer review only - http://bmjopen.bmj.com/site/about/guidelines.xhtml

STROBE Statement—Checklist of items that should be included in reports of ***cross-sectional studies***

Jones et al.: Public opinion on sharing data from UK health services for clinical and research purposes without explicit consent, 2021

	Item No	Recommendation	Section (§, main text section; S, supplementary; F, figure; T, table)	Page number in submitted manuscript (S, supplementary)
**Title and abstract**	1	(*a*) Indicate the study's design with a commonly used term in the title or the abstract	§1	2
		(*b*) Provide in the abstract an informative and balanced summary of what was done and what was found	§1	2
**Introduction**				
Background/ rationale	2	Explain the scientific background and rationale for the investigation being reported	§3	3
Objectives	3	State specific objectives, including any prespecified hypotheses	§3	3
**Methods**				
Study design	4	Present key elements of study design early in the paper	§3, §4	3–4
Setting	5	Describe the setting, locations, and relevant dates, including periods of recruitment, exposure, follow-up, and data collection	§4.3–4.5	4
Participants	6	(*a*) Give the eligibility criteria, and the sources and methods of selection of participants	§4.3	4
Variables	7	Clearly define all outcomes, exposures, predictors, potential confounders, and effect modifiers. Give diagnostic criteria, if applicable	§4.5–4.9, S1.2	4–5, S2–S4
Data sources/ measurement	8*	For each variable of interest, give sources of data and details of methods of assessment (measurement). Describe comparability of assessment methods if there is more than one group	§4.5	5–6
Bias	9	Describe any efforts to address potential sources of bias	§4.9, S1.3	5, S4
Study size	10	Explain how the study size was arrived at	§4.3	4
Quantitative	11	Explain how quantitative variables were handled	§4.9, S1.2–S1.4	5–6, S2–S5

For peer review only - http://bmjopen.bmj.com/site/about/guidelines.xhtml

variables		in the analyses. If applicable, describe which groupings were chosen and why		
Statistical methods	12	(*a*) Describe all statistical methods, including those used to control for confounding	§4.9, S1.3–S1.4	5–6, S4–S5
		(*b*) Describe any methods used to examine subgroups and interactions	§4.9, S1.4	5–6, S5
		(*c*) Explain how missing data were addressed	§4.9	5–6
		(*d*) If applicable, describe analytical methods taking account of sampling strategy	§4.8–4.9, S1.3–S1.4	5–6, S4–S5
		(*e*) Describe any sensitivity analyses	S1.3, ST1	S4, S15
**Results**				
Participants	13*	(a) Report numbers of individuals at each stage of study—eg numbers potentially eligible, examined for eligibility, confirmed eligible, included in the study, completing follow-up, and analysed	§5.1, S2.5, SF1	6, S6–S7, S23
		(b) Give reasons for non-participation at each stage	SF1	S23
		(c) Consider use of a flow diagram	SF1	S23
Descriptive data	14*	(a) Give characteristics of study participants (eg demographic, clinical, social) and information on exposures and potential confounders	§5.1, ST1, SF2, SF3	6, S15, S24, S25
		(b) Indicate number of participants with missing data for each variable of interest	F2–F4, F6–F7, SF1–SF7	Figures (as left); S23–S29
Outcome data	15*	Report numbers of outcome events or summary measures	N/A	N/A
Main results	16	(*a*) Give unadjusted estimates and, if applicable, confounder-adjusted estimates and their precision (eg, 95% confidence interval). Make clear which confounders were adjusted for and why they were included	F2–F7, SF4–SF7	Figures (as left); S26–S29
		(*b*) Report category boundaries when continuous variables were categorized	S1.2	S2–S4
		(*c*) If relevant, consider translating estimates of relative risk into absolute risk for a meaningful time period	N/A	N/A
Other analyses	17	Report other analyses done—eg analyses of subgroups and interactions, and sensitivity	§4.9, §5.4–5.7, S1.4, S2.2–S2.10,	5–8, S5, S6–S9,

For peer review only - http://bmjopen.bmj.com/site/about/guidelines.xhtml

		analyses	ST1, SF4–SF7	S15, S26–S29
**Discussion**				
Key results	18	Summarise key results with reference to study objectives	§6.1	9–10
Limitations	19	Discuss limitations of the study, taking into account sources of potential bias or imprecision. Discuss both direction and magnitude of any potential bias	§6.2	10
Interpretation	20	Give a cautious overall interpretation of results considering objectives, limitations, multiplicity of analyses, results from similar studies, and other relevant evidence	§6.3–6.6, §S3.1–§S3.2	10–13, S10–S14
Generalisability	21	Discuss the generalisability (external validity) of the study results	§6.2	10–11
**Other information**				
Funding	22	Give the source of funding and the role of the funders for the present study and, if applicable, for the original study on which the present article is based	§10	14

*Give information separately for exposed and unexposed groups.

Note: An Explanation and Elaboration article discusses each checklist item and gives methodological background and published examples of transparent reporting. The STROBE checklist is best used in conjunction with this article (freely available on the Web sites of PLoS Medicine at http://www.plosmedicine.org/, Annals of Internal Medicine at http://www.annals.org/, and Epidemiology at http://www.epidem.com/). Information on the STROBE Initiative is available at www.strobe-statement.org.

For peer review only
